# Transcriptomic Analysis in Multiple Myeloma and Primary Plasma Cell Leukemia with t(11;14) Reveals Different Expression Patterns with Biological Implications in Venetoclax Sensitivity

**DOI:** 10.3390/cancers13194898

**Published:** 2021-09-29

**Authors:** Katia Todoerti, Elisa Taiana, Noemi Puccio, Vanessa Favasuli, Marta Lionetti, Ilaria Silvestris, Massimo Gentile, Pellegrino Musto, Fortunato Morabito, Umberto Gianelli, Niccolò Bolli, Luca Baldini, Antonino Neri, Domenica Ronchetti

**Affiliations:** 1 Department of Oncology and Hemato-Oncology, University of Milan, 20122 Milan, Italy; katia.todoerti@studenti.unimi.it (K.T.); elisa.taiana@unimi.it (E.T.); noemi.puccio@studenti.unimi.it (N.P.); vanessa.favasuli@unimi.it (V.F.); marta.lionetti@unimi.it (M.L.); ilaria.silvestris@unimi.it (I.S.); niccolo.bolli@unimi.it (N.B.); luca.baldini@unimi.it (L.B.); 2 Hematology, Fondazione Cà Granda IRCCS Policlinico, 20122 Milan, Italy; 3 Hematology Unit, “Annunziata” Hospital of Cosenza, 87100 Cosenza, Italy; massimogentile@virgilio.it; 4 Department of Emergency and Organ Transplantation, “Aldo Moro” University School of Medicine, 70124 Bari, Italy; pellegrino.musto@uniba.it; 5 Unit of Hematology and Stem Cell Transplantation, AOUC Policlinico, 70124 Bari, Italy; 6 Hematology and Bone Marrow Transplant Unit, Hemato-Oncology Department, Augusta Victoria Hospital, East Jerusalem 91191, Israel; fmorabito@avh.org; 7 Biotechnology Research Unit, Azienda Ospedaliera di Cosenza, 87100 Cosenza, Italy; 8 Department of Pathophysiology and Transplantation, University of Milan, 20122 Milan, Italy; Umberto.Gianelli@unimi.it; 9 Division of Pathology, Fondazione Cà Granda IRCCS Policlinico, 20122 Milan, Italy

**Keywords:** t(11;14), multiple myeloma, Plasma Cell Leukemia, venetoclax, lncRNA, SNHG6

## Abstract

**Simple Summary:**

The growing interest in BCL2 inhibitors for the treatment of multiple myeloma (MM) has led to the need for biomarkers that are able to predict patient’s sensitivity to the drug. The presence of the chromosomal translocation t(11;14) in MM is mainly associated with sensitivity to venetoclax and good prognosis. The incidence of t(11;14) largely increases in primary Plasma Cell Leukemia (pPCL) in association with an unfavorable outcome. Currently, data concerning pPCL sensitivity to venetoclax are virtually absent. In this context, we investigated the transcriptome of MM and pPCL with t(11;14), evidencing that the two clinical entities are likely responsive to venetoclax based on different molecular programs, thus prompting further studies to elucidate better novel potential predictive biomarkers.

**Abstract:**

Mechanisms underlying the pathophysiology of primary Plasma Cell Leukemia (pPCL) and intramedullary multiple myeloma (MM) need to be further elucidated, being potentially relevant for improving therapeutic approaches. In such a context, the MM and pPCL subgroups characterized by t(11;14) deserve a focused investigation, as the presence of the translocation is mainly associated with sensitivity to venetoclax. Herein, we investigated a proprietary cohort of MM and pPCL patients, focusing on the transcriptional signature of samples carrying t(11;14), whose incidence increases in pPCL in association with an unfavorable outcome. In addition, we evaluated the expression levels of the BCL2-gene family members and of a panel of B-cell genes recently reported to be associated with sensitivity to venetoclax in MM. Moreover, transcriptional analysis of lncRNAs in the two clinical settings led to the identification of several differentially expressed transcripts, among which the SNGH6 deregulated lncRNA might be relevant in the pathogenesis and prognosis of pPCL with t(11;14). Overall, our data suggest that MMs and pPCLs with t(11;14) might be responsive to venetoclax based on different molecular programs, prompting further studies to elucidate better novel potential predictive biomarkers.

## 1. Introduction

Multiple myeloma (MM) is a malignant proliferation of antibody-secreting bone-marrow plasma cells (PCs) that is characterized by a highly heterogeneous genetic background with structural chromosomal aberrations and specific gene mutations [1]. MM has different clinical courses, ranging from the asymptomatic clonal proliferation of pre-malignant plasma cells, i.e., monoclonal gammopathy of undetermined significance (MGUS) and smoldering MM (sMM), to truly overt and symptomatic MM, and extra-medullary myeloma/Plasma Cell Leukemia (PCL). PCL can be subdivided clinically into primary and secondary types. Primary PCL (pPCL) presents de novo in the leukemic phase, while secondary PCL (sPCL) arises in the context of a preexisting MM [2]. Despite the advances in MM therapy, patients with pPCL continue to have dismal survival, suggesting that MM and pPCL largely differ in underlying biology [3,4,5].

Several studies investigating the gene mutation patterns [6] and differential gene and miRNAs expression profiles [7,8,9,10,11,12,13], compared to pPCL and MM at molecular and biological levels. Overall, pPCLs have elevated genomic instability, especially for karyotypic complexity, and a higher prevalence of 17p13 deletions and 1q gains compared to MMs. In addition, among translocations involving the immunoglobulin heavy chain locus (IGH), the t(11;14) and t(14;16) are more frequent in pPCL than in MM [14,15,16]; in particular, up to 60% of pPCL patients present with the translocation t(11;14) [17]. However, in the context of pPCLs, those harboring the t(11;14) do not show a better clinical outcome, as generally observed in the MM setting. Recently, it has been reported that enrichment of complex structural changes and high-risk mutational patterns is associated with pPCL patients, particularly with those carrying the t(11;14) [10]. Therefore, mechanisms underlying pPCL pathophysiology compared to intramedullary MM need to be further elucidated, being potentially relevant for improving therapeutic approaches. In such a context, the MM and pPCL subgroups characterized by t(11;14) deserve a focused investigation as the presence of the translocation is mainly associated with sensitivity to venetoclax, a highly potent selective BCL2 inhibitor capable of inducing apoptosis in cells dependent upon BCL2 for survival [18].

In the present study, we investigated the transcriptomic profiles of MM and pPCL samples carrying t(11;14) in a proprietary gene expression profile dataset (GSE116294). In addition, given the increasing importance of BCL2 inhibitors in the treatment of MM, both MMs and pPCLs stratified for the presence of t(11;14) were evaluated for the expression levels of the BCL2 gene family and of a panel of B-cell genes recently reported as associated with venetoclax sensitivity in MM [19]. Our analyses also focused on lncRNAs, leading to the identification of SNGH6 deregulated lncRNA, the expression level of which we found to have clinical relevance in MM.

## 2. Materials and Methods

Full details of multi-omics data in CoMMpass study and statistical analysis are provided in the Supplementary Methods.

### 2.1. Samples

We investigated, by gene expression array, a proprietary cohort of 50 MM patients and 15 primary PCLs (pPCL) that was purified and characterized as previously described [20]. This cohort also included 4 bone-marrow PCs samples purified from normal donors (N) and purchased from Voden, Medical Instruments IT. We investigated by qRT-PCR 11 MM and 5 pPCL samples belonging to the previously described cohort, along with an independent dataset of 8 MM cases, 4 pPCL cases, and 6 normal controls.

### 2.2. Gene Expression Profiling

Fifty MMs, 15 PCLs, and 4 N samples were profiled on GeneChip^®^ Human Gene 2.0 ST arrays (Thermo Scientific, Wilmington, DE, USA), thus obtaining the expression level of 18,642 protein coding transcripts and 10,138 unique lncRNAs, as previously described [20]. The list of differentially expressed genes or lncRNAs between MM and pPCL was gathered by Significant Analysis of Microarrays v5.00, using the tool provided for the shiny package in R software (https://github.com/MikeJSeo/SAM, accessed on 12 May 2021), as previously described [21]. Microarray data were globally analyzed by Gene Set Enrichment Analysis (GSEA, software v.4.0.2) (see Supplementary Materials) [22]. Gene sets were considered significant with a nominal *p*-value < 0.05. All the data are available in the NCBI Gene Expression Omnibus database (GEO; http//www.ncbi.nlm.nih.gov/geo, accessed on 26 March 2021) and are accessible under accession #GSE116294 [23].

### 2.3. Quantitative Real-Rime PCR (qRT-PCR)

We performed qRT-PCR as previously described [24]. Primers used for the analyses are provided in the Supplementary Materials.

## 3. Results

### 3.1. Distinct Transcriptomic Signature of MM and pPCL Samples with t(11;14)

At first, we analyzed the transcriptome of MM and pPCL carrying the t(11;14) translocation by investigating the expression profiling of a cohort of 50 newly diagnosed MM patients representative of the major molecular types of the disease, 15 pPCLs, and four normal controls (Table 1).

**Figure 1:**
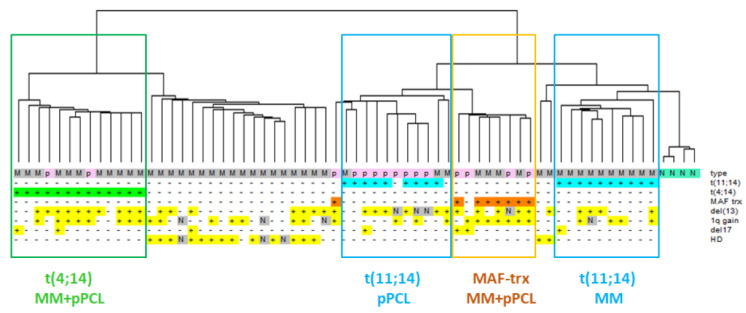
Hierarchical clustering analysis of gene-expression profiles of 50 MM (grey), 15 pPCL (pink) and 4 N samples (green). Samples are grouped according to the expression levels of the 2402 most variable transcripts (varying at least 2-fold in expression levels from the mean across the dataset). Main molecular alterations are shown; N indicates data not available. The specific types (N = normal control, M = MM, *p* = pPCL) are enriched by colored sub-branches, also highlighted by the appropriately colored box (see text).

We took advantage of the GeneChip^®^ Human Gene 2.0 ST array, which is able to detect 35.458 transcripts, including 18.642 protein coding (53%) and 10.138 lncRNAs (29%), upon annotation on unambiguous entries in GENCODE encyclopedia (V25) [20]. Hierarchical clustering of the 2402 most variable transcripts in the dataset showed that the grouping was mainly driven by the IGH translocations; in fact, t(14;16)/t(14;20) or t(4;14) cases clustered together independently of belonging to MM or pPCL groups, suggesting that such translocation events had consequences on the transcriptional fingerprint stronger than those due to leukemic phenotype. However, MMs and pPCLs with t(11;14) were split into two subclusters: MM cases were in the one grouping of normal controls, whereas pPCLs were grouped closer to samples with MAF translocation (MAF-trx) (Figure 1).

The 2402 most variable transcripts driving the clustering are mainly enriched in protein coding genes (66%) but also include lncRNAs (15%), as well as a heterogeneous group of transcripts among which immunoglobulin genes, small nucleolar RNAs, miRNAs, and pseudogenes. Based on these considerations, we focused our further studies on protein-coding genes and lncRNAs.

### 3.2. Protein-Coding Genes: Molecular Pathways and Gene Sets Modulated in MM and pPCL in Association with t(11;14)

To identify the genes that specifically distinguished the two clinical entities in the context of t(11;14), we performed a supervised analysis between the seven pPCL and the 12 MM cases carrying the translocation. A comprehensive list of 2416 differentially expressed (DE) protein-coding genes (i.e., 1021 upregulated and 1395 downregulated genes in pPCL samples) was obtained at a low stringency cutoff (FDR < 10%) (Table S1). In the attempt to focus on DE transcripts more specifically related to t(11;14), we excluded from the 2416 DE list the transcripts in common with those specifically differentiating the 50 MM from the 15 pPCL patients (Table S2). Based on this approach, we obtained a signature of 628 transcripts (specifically, 165 upregulated and 463 downregulated genes in pPCL samples; Table S3). Interestingly, the functional annotation analysis of this signature, aimed at identifying highly significant represented categories, revealed the enrichment in eight biological processes among which the regulation of transcription through DNA binding, the regulation of cytokine biosynthetic process, and the positive regulation of intracellular protein transport (Table S4).

In addition, to define those molecular pathways differently modulated in relation to MM and pPCL with t(11;14), a Gene Set Enrichment Analysis (GSEA) was performed on the list of 628 DE protein-coding genes that were ranked based on fold-change values (Table S3). Remarkably, the enrichment map on the top GSEA gene sets, based on Gene Ontology (GO) Biological Process (BP) terms, revealed a network of connected functional modules concerning downregulated genes mainly involved in the immune system process and its positive regulation, the positive regulation of cell population proliferation, the cell surface receptor signaling pathway, the signal transduction by protein phosphorylation, the MAPK cascade and its regulation, and the developmental processes (Figure S1).

**Figure 2:**
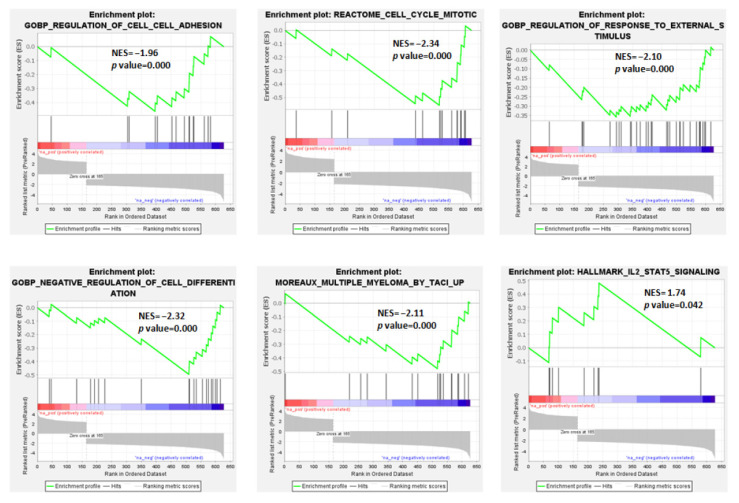
Enrichment plots of 6 selected gene sets detected by GSEA. The green curves show the enrichment score and reflect the degree to which each gene (black vertical lines) is represented in the ranked gene list. NES: Normalized Enrichment Score.

In detail, among the significantly enriched gene sets (Table S5), we evidenced that the t(11;14) pPCL group was negatively associated with the regulation of cell–cell adhesion, response to external stimuli, and the negative regulation of cell differentiation. GSEA also demonstrated for the t(11;14) pPCL cases a negative association with the signature defined by high TACI expression level in MM by Moreaux et al. [25] (Figure 2 and Table S6). On the contrary, they were significantly enriched in genes involved in IL2-STAT5 signaling.

### 3.3. Differential Expression of BCL2 Gene Family Members in Association with t(11;14)

**Figure 3:**
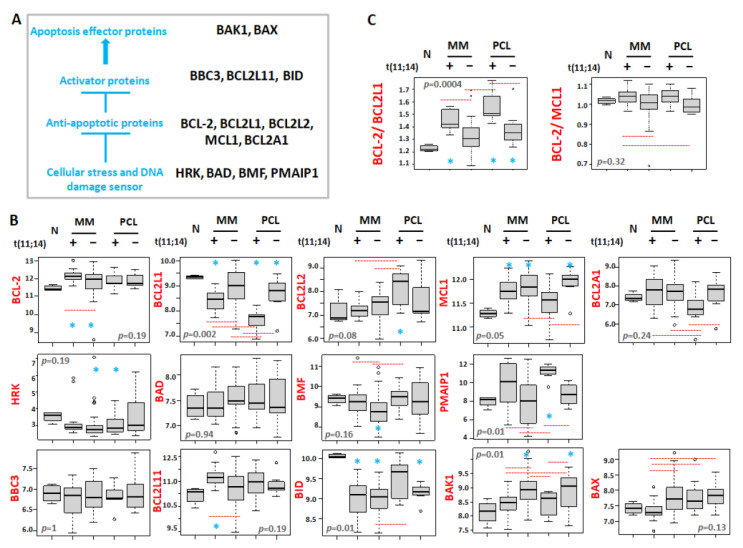
(**A**) Scheme of the BCL2 family protein interactions. (**B**) Box plot representation of the mRNA expression of BCL2 family genes in 4 normal controls (N), 12 MM patients carrying t(11;14), 38 MMs without t(11;14), 7 pPCL patients carrying t(11;14), and 8 pPCLs without t(11;14) evaluated by GeneChip^®^ Human Gene 2.0 ST array (GSE116294). (**C**) Box plot representation of the ratio BCL2/BCL2L1 and BCL2/MCL1 expression in each group. In each panel, red dashed lines below/above two groups indicate significant differences in their gene expression level; blue asterisks indicate significant differences between the indicated group and N (Dunn’s test *p*-values are reported in Figure S2). BH adjusted Kruskal–Wallis test *p*-value is reported for each panel.

Given the increasing importance of BCL2 inhibitors in the treatment of MM and the enrichment of the responders in patients with t(11;14), we investigated the expression levels of all the BCL2 gene family members in both MM and pPCL samples stratified for the presence or absence of the t(11;14), and normal controls (Figure 3A,B and Figure S2).

Concerning the anti-apoptotic proteins, a significant overexpression of BCL2 was found in MM patients with t(11;14) compared to the normal controls, MM cases not carrying the translocation and pPCL samples, irrespective of the presence of the t(11;14). On the other hand, we observed a significant downregulation of BCL2L1 (known to encode for BCL-XL) in all pathological samples compared to normal controls, and in particular in patients carrying t(11;14), markedly in pPCL. Therefore, when considering the BCL2/BCL2L1 ratio, known as an essential response predictor to venetoclax [26,27], it was significantly higher in both MM and pPCL with t(11;14) (Figure 3C). Furthermore, pPCLs with t(11;14) displayed BCL2L2 expression levels significantly higher than those in normal samples and MMs, regardless of the presence of t(11;14); conversely, pPCLs with the t(11;14) showed the lowest BCL2A1 expression levels compared to all the other pathological groups. Concerning MCL1, when compared to normal controls, it was significantly overexpressed in all pathological groups but t(11;14) pPCLs. However, MCL1 was significantly downregulated in t(11;14) pPCLs if compared to pPCLs without t(11;14), likely due to the enrichment of patients carrying 1q gain in the negative t(11;14) pPCLs (see Figure 1). As a result, a higher ratio of BCL2/MCL1 was observed in MMs and pPCLs with t(11;14) (Figure 3C), although not reaching statistical significance in pPCL (Figure S2).

Among genes encoding sensors for cellular stress and DNA damage, higher PMAIP1 expression levels were associated with the presence of t(11;14) in both MM and pPCL, although PMAIP1 overexpression in t(11;14) MMs did not reach significance compared to normal controls. Conversely, a lower BMF expression was significantly associated with the absence of t(11;14) only in the MM group.

Regarding apoptosis activator proteins, higher BCL2L11 expression levels were associated with t(11;14) in MM. Finally, focusing on apoptosis effector proteins, we found that, in both MMs and pPCLs, BAK1 overexpression was significantly associated with the absence of t(11;14), being also significant if compared to normal controls; in addition, the median lowest BAX expression was observed in t(11;14) MMs, although not reaching significance when compared to N controls.

The expression levels of BCL2, BCL2L1, and MCL1 were validated by qRT-PCR in 11 MM and five pPCL samples profiled by gene expression arrays for whom RNA material was available (Pearson analyses in Figure S3a). Furthermore, we investigated by qRT-PCR an independent panel of 18 samples, including six normal controls, eight MMs, and four pPCLs. Overall, the global cohort included six normal controls, nine MM cases, and three pPCL cases with t(11;14), and 10 MM and six pPCL samples without this translocation. For all the three genes, our qRT-PCR data confirmed the pattern of expression levels between the four different molecular and clinical groups found by gene expression arrays; in particular, a higher ratio of BCL2/BCL2L1 and BCL2/MCL1 was confirmed in MM and pPCL with t(11;14) (Figure S3b).

Finally, by taking advantage of the large public Multiple Myeloma Research Foundation (MMRF) CoMMpass dataset including MM samples characterized for genome abnormalities, transcriptome, and clinical outcome, we confirmed that BCL2, BMF, PMAIP1, and BCL2L11 expression levels were higher in MMs with t(11;14) than in MMs without t(11;14). In addition, we validated the lower expression levels of BCL2L1, BAK1, and BAX in MMs carrying t(11;14) compared to the other MM group. Moreover, similarly to our proprietary database, in the CoMMpass cohort no differences in the expression of MCL1, BCL2A1, BBC3, and BAD were detectable in MMs with or without the t(11;14). Concerning BCL2L2, HRK, and BID, whose expression levels did not reach any relevant difference between the two MM groups in our database, probably due to the limited number of patients, in the CoMMpass cohort their expression was significantly higher in MMs with t(11;14) (Figure S4).

### 3.4. Differential Expression Patterns of B-Cell-Associated Genes

**Figure 4:**
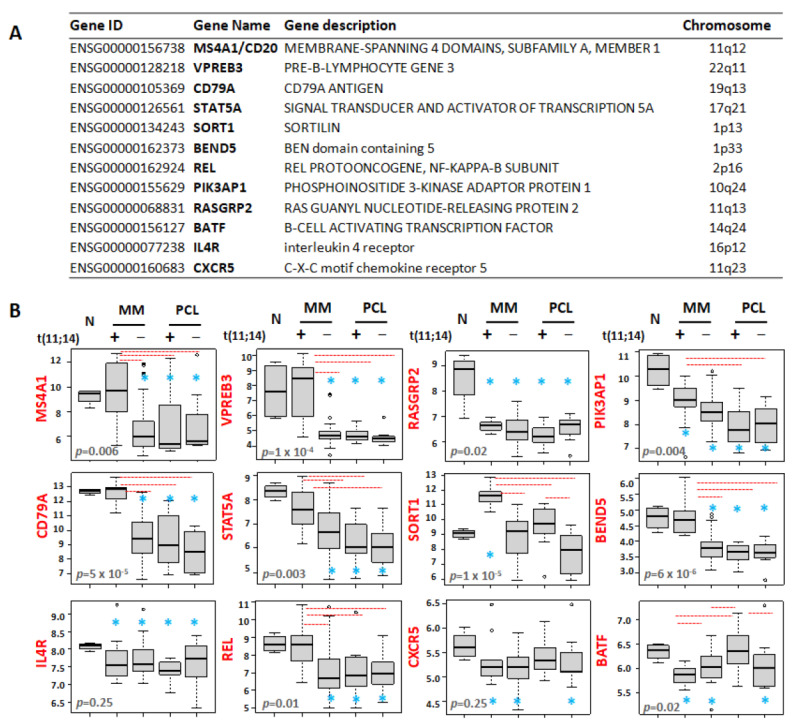
(**A**) Summary information on the 12 B-cell-related genes (Figure 4 in Gupta et al. [19]). (**B**) Box plot representation of the expression of B-cell genes from the signature of venetoclax sensitive MM, in 4 normal controls (N), 12 MM patients carrying t(11;14), 38 MMs without t(11;14), 7 pPCL patients carrying t(11;14), and 8 pPCLs without t(11;14) evaluated by GeneChip^®^ Human Gene 2.0 ST array (GSE116294). In each panel, red dashed lines below/above two groups indicate significant differences in their gene-expression level; blue asterisks indicate significant differences between the indicated group and N (Dunn’s test *p*-values are reported in Figure S5). BH adjusted Kruskal–Wallis test *p*-value is reported for each panel.

Recent data described a gene expression signature enriched for B-cell-associated genes in MM cell lines and MM patients sensitive to venetoclax [19]. Given the enrichment of venetoclax responsive patients in the MM group with t(11;14), we wished to investigate the expression levels of these putative biomarkers in both MM and pPCL samples stratified for the presence of t(11;14) (Figure 4A,B and Figure S5). In particular, we focused on the twelve B-cell-associated genes all reported to be upregulated in venetoclax sensitive MMs [19]. Among them, there are MS4A1, VPREB3, and SORT1 which are involved in immune system process and cell surface receptor signaling pathway (Figure S1), and belong to the signature discriminating MMs and pPCLs with t(11;14) reported here (see Table S3). Interestingly, MS4A1 and VPREB3 along with CD79A, STAT5A, BEND5, and REL genes maintained expression levels similar to normal samples only in t(11;14) MMs, whereas their expression levels were significantly lower in MMs without t(11;14) and in both pPCL groups. Otherwise, SORT1 was specifically upregulated in MMs with the t(11;14) compared to normal controls and all the other pathological subgroups. With regard to the PIK3AP1 and RASGRP2 genes, all pathological subgroups showed significantly lower expression levels than normal controls, markedly in pPCL patients as far as PIK3AP1 is concerned. On the contrary, BATF maintained expression levels similar to the normal controls only in t(11;14) pPCLs, whereas its expression level was significantly lower in all the other pathological groups, reaching the lowest median level in t(11;14) MM.

As for BCL2 family genes, MS4A1, CD79A, SORT1, and BEND5 expression levels were validated by qRT-PCR in 16 samples profiled by gene expression arrays for whom RNA material was available (Pearson analyses in Figure S6a) and in an independent panel of 18 samples (see above). Overall, the qRT-PCR analysis of six normal controls and 28 samples stratified for the presence of t(11;14) confirmed the expression patterns between the different groups found by arrays for the four genes (Figure S6b).

Finally, the significant differences found in our cohort of MM stratified for the presence of t(11;14) were confirmed in the CoMMpass dataset. In detail, VPREB3, MS4A1, CD79A, STAT5A, SORT1, BEND5, and REL genes were all significantly upregulated in MMs with t(11;14) compared to the other MM subgroup. Moreover, BATF expression level was significantly lower in the MM group with t(11;14), whereas no differences in the expression of the RASGRP2 gene were detectable between the two MM groups in CoMMpass cohort. Concerning CXCR5, PIK3AP1, and IL4R, for which differences in expression levels between the two groups in our database did not reach significance, probably due to the limited number of patients, in the CoMMpass cohort, their expression was significantly higher in MMs with t(11;14) (Figure S7).

### 3.5. Differential Expression Patterns of lncRNAs Associated with t(11;14)

**Figure 5:**
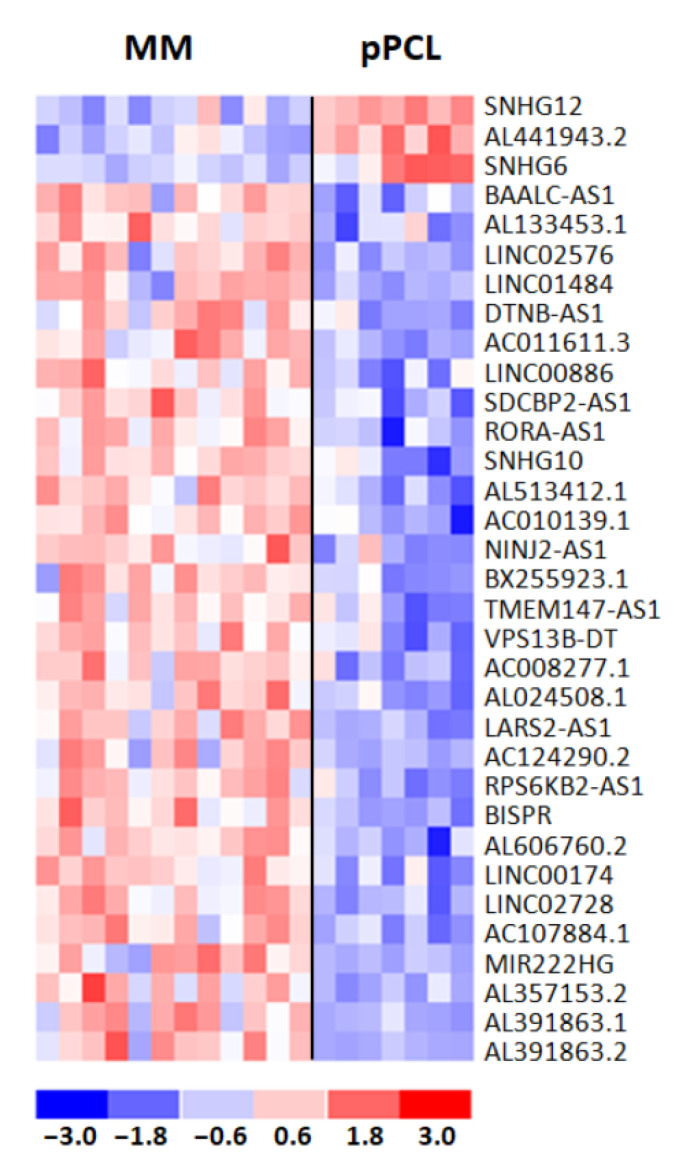
Heatmap of the 33 differentially expressed lncRNAs in 12 MM and 7 pPCL patients with t(11;14) chromosomal translocation. The colored scaled bar represents standardized rows by subtracting the mean and divided by the standard deviation.

Next, we investigated lncRNA transcriptional patterns specifically differentiating pPCL and MM cases carrying t(11;14). In detail, we performed a supervised analysis between the 7 pPCLs and the 12 MM cases carrying such a translocation, detecting 38 DE lncRNAs (i.e., three upregulated and 35 downregulated lncRNAs in pPCL samples). As for protein-coding genes, we excluded from further analyses the five lncRNAs in common with the analysis comparing all 15 pPCL and 50 MM cases (Table S7). Therefore, we obtained a distinctive list of 33 lncRNAs specifically deregulated in the t(11;14) context (Figure 5), a majority of which (30 out of 33) were downregulated in pPCL vs. MM cases and were mainly represented by lncRNAs antisense to coding-genes (42%); the remaining cases included eight novel transcripts, five long intergenic non-protein-coding RNAs, one miRNA host gene, three small nucleolar RNA host genes, and two divergent transcripts (Table S8).

Based on the recurrent evidence that the transcription of mRNAs and lncRNAs appears to be closely regulated, leading to a cis-regulatory relationship between the two transcripts [28,29,30], we investigated the levels of expression of overlapping or nearby transcripts localized in close proximity to the 33 lncRNAs (up to 100 kb window). Therefore, we considered 145 transcript–lncRNA pairs and analyzed the correlation between their expression levels across the entire dataset of 774 MM cases profiled by RNA-seq in the CoMMpass cohort (Table S9). As BISPR lncRNA and several transcripts were not annotated in the CoMMpass matrix, we could evaluate 123 out of the 145 transcript–lncRNA pairs. For 16 of 33 DE lncRNAs, all downregulated in pPCLs but SNHG12, a significant Pearson’s correlation (r > 0.5, *q*-value < 0.001) was observed for 26 lncRNA-gene pairs; among them, we identified 10 lncRNA-coding genes couples (Table 2) and 10 lncRNA–lncRNA pairs (Table S9).

### 3.6. Clinical Relevance of lncRNAs

To gain insight into the clinical relevance of the expression level of each of the 33 DE lncRNAs, we took advantage of the CoMMpass database. Specifically, clinical and outcome data concerning Overall Survival (OS) and Progression-Free Survival (PFS), freely accessible from MMRF Study, were analyzed in 767 MM cases with available RNA-seq data. In detail, for each lncRNA we defined two MM patient groups based on their median lncRNA expression level (see Supplementary Materials). Kaplan–Meier curves indicated the group with lower expression levels associated with a shorter PFS for Linc00886, NINJ2-AS1, AL513412.1, and Linc02728 out of all the 33 tested lncRNAs (Figure S8). At the same time, Linc00886 and NINJ2-AS1 resulted as unfavorable prognostic factors even in association with OS. As expected, we found the expression levels of these four lncRNAs significantly downregulated in pPCL compared to MM samples. Conversely, a higher expression level of SNHG6 was associated with significantly inferior OS. Accordingly, SNHG6 was upregulated in pPCL patients.

The clinical impact of these five lncRNAs was further investigated by Cox regression univariate analysis. For three lncRNAs, their lower expression level was associated with a significantly higher risk in PFS, and for one of them (Linc00886) also in OS (Table 3). Conversely, higher SNHG6 expression level was associated with a significantly higher risk in OS. Cox univariate analysis indicated a significantly increased risk in terms of PFS and OS for cases with ISS stage III, 13q deletion, TP53 alterations associated with 1q gain, DIS3 mutations, t(4;14), the occurrence of 1q gain/amp, and MAF translocation, while ISS stage I and the presence of HD reduced such risks. Moreover, 1p deletion and MYC translocation showed to be significantly associated with an increased risk in terms of OS and PFS, respectively (Table 3).

Next, we constructed a series of Cox multivariate analyses to assess the independent prognostic power for PFS and OS of the four lncRNAs (Table S10). Every single lncRNA was introduced into the corresponding model together with ISS and all genetic/molecular variables significantly associated with PFS and OS at univariate analyses. Notably, SNHG6 was the only one whose higher expression levels retained an independent significant prognostic power for OS when tested in Cox regression multivariate analysis (Table S10). Furthermore, we analyzed the association of SNHG6 expression level with the major MM oncogenic lesions (Table S11) and found that among the main IgH translocations, the t(4;14) was significantly associated with low SNHG6 expression level (*p* = 0.037). Concerning the copy number alterations (CNAs) commonly found in MM, 1q gain/amp occurred significantly more frequently in patients with low SNHG6 expression levels (*p* = 0.0074). Finally, the SNHG6 expression level was not correlated with the presence of mutations affecting K-RAS, N-RAS, BRAF, FAM46C, TP53, and DIS3 genes (Table S11). Overall, these data reinforce the clinical relevance of SNHG6 expression level in MM.

## 4. Discussion

We investigated a proprietary cohort of MM and pPCL patients, focusing on the transcriptome of samples carrying t(11;14), whose incidence increases in pPCL in association with an unfavorable outcome. These selection criteria allowed us to investigate possible molecular mechanisms other than those associated with this chromosomal abnormality that could better characterize the differences between these two clinical and pathological forms associated with the t(11;14). We found that the two clinical entities are distinguishable at the transcriptomic level despite sharing the same t(11;14) genetic background. In particular, we defined the specific signatures of coding genes and lncRNAs that discriminate MM and pPCL samples with t(11;14) by refining from the lists those transcripts that significantly differentiate the two clinical settings in general.

Our data showed that the pPCL group with t(11;14) was particularly enriched in genes involved in IL2-STAT5 signaling (Figure 2), which is one of the most rapidly activated signaling pathways following stimulation by IL-2 family cytokines. This pathway, presumably in concert with others, allows an efficient cellular reaction in response to the micro-environmental changes in vivo; in addition, this system plays roles in lymphoid development, as well as in modulation of T-cell and NK-cell immune responses [31]. Our results also demonstrated in pPCLs with t(11;14) a negative association with the signature defined by high TACI expression level in MM, as reported by Moreaux et al. [25]. Interestingly, according to this signature, MM cells with high TACI expression levels displayed a mature PC gene signature, indicating dependence on the bone marrow (BM) microenvironment; in contrast, the low-TACI-expressing group had a gene signature of plasma blasts, suggesting a decreased need for BM environment support.

The interest in BCL2 inhibitors in MM treatment is continuously increasing, especially for venetoclax that is capable of inducing apoptosis in cells dependent upon BCL2 for survival. Most myelomas are MCL1 dependent; however, a subset of MM enriched for t(11;14) is co-dependent on BCL2 and thus sensitive to venetoclax. Indeed, in numerous pre-clinical and clinical studies venetoclax has been reported to be consistently more effective in MM harboring the t(11;14) translocation [32,33,34]. However, even amongst t(11;14) MM patients, the response rate is only 40–60% [32]; this suggests that the biology underlying the heterogeneity of drug response remains poorly understood. Furthermore, a recent study indicated that remnants of B-cell biology are associated with BCL2 dependency. Indeed, despite possessing the hallmarks of terminally differentiated PCs, venetoclax sensitive MMs retain or aberrantly reactivate aspects of the B-cell program, including BCL2 dependence, along with other specific patterns of B-cell gene expression and chromatin accessibility, and point to novel biomarkers of venetoclax sensitive MM independent of t(11;14) [19]. Currently, there are very little data from case reports on the efficacy of venetoclax in pPCL with t(11;14) [26,35,36,37], and defining a specific predictor of venetoclax sensitivity is challenging. Our study focused on the expression levels of the BCL2 gene family and of a panel of B-cell genes associated with venetoclax sensitive MM [19] in both MMs and pPCLs stratified for the presence of t(11;14).

In detail, we found that MM and pPCL cases with t(11;14) showed a distinctive expression pattern of the BCL2 family genes (Figure 3), although, globally, both clinical entities maintained similar BCL2/BCL2L1 and BCL2/MCL1 ratios, which are parameters that have been reported to correlate with venetoclax sensitivity [27,32,38,39]. Given the BCL2 family proteins redundant interactions, a detailed portrait of their expression could explain the heterogeneity of BCL2 dependence in MM, since t(11;14) subtypes enrich for venetoclax sensitive patients; however, not all t(11;14) patients respond, and some t(11;14) negative patients do respond. Concerning pPCL, our data urge the need both for their additional validation in a larger cohort of patients and for further clinical studies on venetoclax efficacy in this clinical entity.

Besides the distinct expression pattern of BCL2 family genes, MM and pPCL cases with t(11;14) largely differed for the expression of B-cell genes associated with venetoclax sensitive MM [19]. Indeed, with respect to previous reported data, our results indicated that MMs with t(11;14) had an expression profile more similar to venetoclax sensitive MMs than t(11;14) pPCLs, which showed a significant downregulation of B-cell-associated genes, such as CD79A, MS4A1, VPREB3, STAT5A, SORT1, BEND5, and REL transcripts. However, t(11;14) pPCLs exhibited significantly higher levels of BATF expression, a transcription factor specifically expressed in activated B cells. Notably, it has been demonstrated that increased BATF expression improves sensitivity to venetoclax in HMCLs without changes in expression of BIM, BCL2, BCL2L1, or MCL1, thus suggesting that a BATF mediated transcriptional program may contribute to venetoclax response in MM [19]. To note, BATF expression levels were significantly lower in t(11;14) MMs compared to negative MM patients.

Overall, these data suggest that MMs and pPCLs may be sensitive to venetoclax based on different molecular programs, implying that specific biomarkers should be investigated in the two clinical entities as the best predictor of venetoclax sensitivity.

Concerning lncRNAs, our data indicated that MMs and pPCLs with t(11;14) scarcely differed from each other based on lncRNA expression. However, some of the lncRNAs found in the signature are suggestive for pathogenic implication in pPCL and warrant further investigation. Indeed, AC107884.1, whose expression levels in pPCLs are significantly lower than in MM, is highly correlated with the OLFML1 gene (Table 2), whose activity is fundamental for osteoblast mineralization [40]. Very interesting are also AL391863.1 and AL391863.2 lncRNAs that are both highly correlated with SNX9 gene (Table 2), a member of a large family of proteins involved in endocytosis and intracellular trafficking [41], which has been recently described as significantly overexpressed in venetoclax sensitive MMs [19]. To note, Linc02728 is located sense between the asparaginyl-tRNA synthetase 2 (NARS2) and GRB2-associated binding protein 2 (GAB2), whose phosphorylation is essential in IL-6-induced proliferation and survival of MM cells [42]. Finally, we identified SNHG6 as significantly upregulated in pPCLs with t(11;14). In recent years, accumulating evidence have revealed that SNHG6 was aberrantly expressed in various cancers and was significantly correlated with clinical stage and prognosis [43,44,45]. Our investigation in the CoMMpass dataset showed that its overexpression retains an independent significant prognostic power for OS in MM, thus conferring, for the first time, clinical relevance to SNHG6 expression level in the MM context. Although already highly expressed in MMs, SNHG6 expression levels in pPCLs with t(11;14) are significantly higher than those in MMs irrespective of the presence of t(11;14) and pPCLs without t(11;14) (Figure S9). Overall, these data prompted further studies investigating the biological role and activity of SNHG6 in the pathobiology of pPCL with t(11;14).

## 5. Conclusions

Transcriptomic analyses of MMs and pPCLs with t(11;14) indicated that the two clinical entities might be responsive to venetoclax based on different molecular programs, prompting further study to identify biomarkers of venetoclax sensitivity specific for MM or pPCL. Our results also suggested that SNHG6 deregulation might be relevant in the pathogenesis and prognosis of pPCLs with t(11;14).

## Figures and Tables

**Table 1:** Molecular characteristics of 50 MM and 15 pPCL patients. NA = not available.

Parameter	MM			pPCL		
Median age, years (range)	67 (39–78)			67 (39–78)		
Male gender (%)	23 (46%)			5 (33%)		
ISS, I–II–III	13–28–9					
**Sample Features**	**Positive**	**Negative**	**NA**	**Positive**	**Negative**	**NA**
HD	15	32	3	0	15	0
t(11;14)	12	38	0	7	8	0
t(4;14)	12	38	0	2	13	0
MAF-trx	5	45	0	4	11	0
none	3	47		2	13	
del(17p)	4	46	0	3	12	0
del(13q)	27	23	0	9	2	4
1q gain	25	21	4	7	8	0

**Table 2:** List of 10 lncRNAs with a significant Pearson’s correlation with protein-coding genes.

lncRNA Gene ID	lncRNA Gene Name	Overlapping/Nearby Gene ID	Overlapping/Nearby Gene Name	Pearson’s Correlation
ENSG00000223642	AC008277.1	ENSG00000123636	BAZ2B	r = 0.53, *q*-value = 1.4 × 10^−57^
ENSG00000224220	DTNB-AS1	ENSG00000138101	DTNB	r = 0.89, *q*-value = 4.3 × 10^−272^
ENSG00000229502	AL391863.1	ENSG00000130340	SNX9	r = 0.65, *q*-value = 3.5 × 10^−95^
ENSG00000234361	AL391863.2	ENSG00000130340	SNX9	r = 0.58, *q*-value = 2.4 × 10^−71^
ENSG00000234684	SDCBP2-AS1	ENSG00000088832	FKBP1A	r = 0.56, *q*-value = 1.8 × 10^−64^
ENSG00000245534	RORA-AS1	ENSG00000128915	ICE2	r = 0.51, *q*-value = 1.5 × 10^−38^
ENSG00000247081	BAALC-AS1	ENSG00000164930	FZD6	r = 0.84, *q*-value = 1.5 × 10^−209^
ENSG00000251364	AC107884.1	ENSG00000183801	OLFML1	r = 0.61, *q*-value = 2.2 × 10^−79^
ENSG00000257453	AC011611.3	ENSG00000139289	PHLDA1	r = 0.87, *q*-value = 1.2 × 10^−233^
ENSG00000272189	AL024508.1	ENSG00000135525	MAP7	r = 0.55, *q*-value = 1.6 × 10^−63^

**Table 3:** Results of Cox regression univariate analysis for 5 lncRNAs with an unfavorable clinical outcome by Kaplan–Meier analysis. Higher expression level based on median cutoff in 497 MM dataset was tested in both OS and PFS. Significant *p*-values are marked in bold. Number (N) and fraction of MM patients at higher expression levels are indicated for each lncRNA. Clinical and molecular variables important for MM were also tested.

		OS Univariate Cox Analysis		PFS Univariate Cox Analysis	
Molecular Lesion	N (%) High Level	HR (95% CI)	*p*-Value	HR (95% CI)	*p*-Value
Linc00886.median	240 (48%)	0.65 (0.45–0.94)	**0.0213**	0.73 (0.57–0.95)	**0.017**
NINJ2-AS1.median	246 (49%)	0.71 (0.49–1.03)	0.0691	0.76 (0.59–0.99)	**0.0386**
AL513412.1.median	233 (47%)	0.77 (0.53–1.12)	0.173	0.78 (0.60–1.01)	0.0641
Linc02728.median	263 (53%)	0.79 (0.55–1.13)	0.195	0.76 (0.59–0.98)	**0.0343**
SNHG6.median	262 (53%)	1.45 (1.00–2.09)	**0.0478**	1.11 (0.86–1.43)	0.437
del1p.CDKN2C	143 (29%)	1.61 (1.10–2.35)	**0.0148**	1.30 (0.98–1.71)	0.0646
del13q.RB1	258 (52%)	2.11 (1.44–3.09)	**0.000119**	1.71 (1.32–2.21)	**4.71 × 10^−5^**
HD	281 (57%)	0.64 (0.44–0.92)	**0.0149**	0.67 (0.52–0.86)	**0.00166**
TP53.alt.1q.gain.amp	19 (4%)	3.63 (1.89–6.97)	**0.000103**	2.46 (1.38–4.42)	**0.00243**
TP53.alt	40 (8%)	1.05 (0.53–2.07)	0.892	0.85 (0.52–1.39)	0.511
gain.amp.1q	164 (33%)	1.68 (1.17–2.43)	**0.0051**	1.52 (1.17–1.98)	**0.00151**
DIS3mut	50 (10%)	1.63 (1.06–2.50)	**0.0264**	1.62 (1.18–2.22)	**0.00308**
NRASmut	117 (24%)	0.90 (0.62–1.32)	0.596	1.01 (0.79–1.29)	0.924
KRASmut	121 (24%)	1.07 (0.77–1.49)	0.694	1.03 (0.80–1.31)	0.827
BRAF.mut	36 (7%)	1.18 (0.60–2.31)	0.628	0.88 (0.52–1.48)	0.626
FAM46C.mut	49 (10%)	0.78 (0.42–1.43)	0.419	1.07 (0.74–1.53)	0.728
TRAF3.mut	38 (8%)	0.42 (0.17–1.06)	0.0668	0.80 (0.52–1.22)	0.297
t11.14	102 (21%)	0.92 (0.58–1.46)	0.726	0.94 (0.68–1.30)	0.694
t4.14	69 (14%)	1.63 (1.04–2.55)	**0.0335**	1.60 (1.14–2.23)	**0.00612**
trx.MAF	33 (7%)	1.96 (1.08–3.57)	**0.0276**	1.63 (1.03–2.57)	**0.0376**
trx.MYC	20 (4%)	1.92 (0.94–3.94)	0.0748	1.94 (1.13–3.34)	**0.0163**
ISS I	187 (38%)	0.31 (0.19–0.50)	**1.86 × 10^−6^**	0.46 (0.34–0.61)	**1.20 × 10^−7^**
ISS II	171 (34%)	1.13 (0.78–1.64)	0.524	1.18 (0.91–1.54)	0.213
ISS III	139 (28%)	2.45 (1.70–3.53)	**1.62 × 10^−6^**	1.92 (1.48–2.50)	**1.14 × 10^−6^**

## Data Availability

All the data are available in the NCBI Gene Expression Omnibus database (GEO; https://www.ncbi.nlm.nih.gov/geo, 26 March 2021) and are accessible under accession #GSE116294. MMRF CoMMpass data are available at https://research.themmrf.org/and retrieved from the Interim Analysis 15a (MMRF_CoMMpass_IA15a, 16 October 2020).

## Supplementary Materials

The following are available online at https://www.mdpi.com/article/10.3390/cancers13194898/s1. Supplementary Methods. Supplementary Figure S1: Enrichment map on top 8 GSEA gene sets based on GO-BP terms, performed by Cluster Profiler analysis on DE global protein-coding gene list. B-cell-related genes upregulated in venetoclax-sensitive MMs are marked by a red box. Supplementary Figure S2: Results of Dunn’s test evaluating the differences in the expression of BCL2 gene family in MMs and PCLs stratified by the presence of t(11;14), and in normal controls (N). Significant *p*-value < 0.05 is marked in red/bold. Expression levels are plotted in Figure S3B,C. Supplementary Figure S3: Quantitative RT-PCR validation of BCL2, BCL2L1, and MCL1 expression levels. (**A**) Pearson’s correlation coefficient was calculated between GEP data and quantitative RT-PCR results expressed as 2^−ΔCt^ in 16 samples. (**B**) Box plot representation of the gene expression levels in 34 samples, including 6 normal controls (N), 9 MM patients carrying t(11;14), 10 MMs without t(11;14), 3 pPCL patients carrying t(11;14), and 6 pPCLs without t(11;14) evaluated by Kruskal–Wallis test (*p*-values are shown for each panel). The expression levels are represented as 2^−ΔCt^. In each panel, red dashed lines above two groups indicate significant differences in their gene expression level; blue asterisks indicate significant differences between the indicated group and N, evaluated by Dunn’s test (table under the corresponding box plot, significant *p*-value < 0.05 is marked in red/bold). Supplementary Figure S4: Box plot representation of the mRNA expression of BCL2 family genes in 156 MMs with t(11;14) and 618 MMs without t(11;14) from the CoMMpass database. For each plot, BH adjusted *p*-value is reported. Supplementary Figure S5: Results of Dunn’s test evaluating the differences in the expression of B-cell genes from the signature of venetoclax sensitive myeloma in MMs and PCLs stratified by the presence of t(11;14), and in normal controls (N). Significant *p*-value < 0.05 is marked in red/bold. Expression levels are plotted in Figure 4. Supplementary Figure S6: Quantitative RT-PCR validation of BEND5, SORT1, CD79A, and MS4A1 expression levels. (**A**) Pearson’s correlation coefficient was calculated between GEP data and quantitative RT-PCR results expressed as 2^−ΔCt^ in 16 samples. (**B**) Box plot representation of the gene expression levels in 34 samples, including 6 normal controls (N), 9 MM patients carrying t(11;14), 10 MMs without t(11;14), 3 pPCL patients carrying t(11;14), and 6 pPCLs without t(11;14) evaluated by Kruskal–Wallis test (*p*-values are shown for each panel). The expression levels are represented as 2^−ΔCt^. In each panel, red dashed lines above two groups indicate significant differences in their gene expression level; blue asterisks indicate significant differences between the indicated group and N, evaluated by Dunn’s test (table under the corresponding box plot, significant *p*-value < 0.05 is marked in red/bold). Supplementary Figure S7: Box plot representation of the mRNA expression of the signature of venetoclax sensitive myeloma in 156 MMs with t(11;14) and 618 MMs without t(11;14) from the CoMMpass database. For each plot, BH adjusted *p*-value is reported. Supplementary Figure S8: Kaplan–Meier survival curves in 767 BM_1 MM cases stratified according to the expression level of the specified lncRNAs with respect to PFS (**A**) and OS (**B**). Supplementary Figure S9: Box plot representation of SNHG6 gene expression in 4 normal controls (N), 12 MM patients carrying t(11;14), 38 MMs without t(11;14), 7 pPCL patients carrying t(11;14), and 8 pPCLs without t(11;14) evaluated by GeneChip® Human Gene 2.0 ST array (GSE116294). Red dashed lines below/above two groups indicate significant differences in their gene expression level (Dunn’s test, p-value < 0.05 reported in the table on the right). Supplementary Table S1: List of 2416 differentially expressed coding genes in 7 t(11;14)-pPCL compared to 12 t(11;14)-MM cases, by SAM analysis (FDR < 10%). Transcripts are ordered according to SAM (d) score. Supplementary Table S2: List of 4847 differentially expressed coding genes in 15 pPCL compared to 50 MM cases by SAM analysis (FDR < 10%). Transcripts are ordered according to SAM (d) score. Supplementary Table S3: List of 628 specific differentially expressed coding genes in 7 t(11;14)-pPCL compared to 12 t(11;14)-MM cases. Transcripts are ordered according to SAM (d) score. Supplementary Table S4: List of the significantly enriched Annotation Clusters for the 628 differentially expressed protein-coding genes (Enrichment Score > 1.3) by DAVID 6.8 Bioinformatics tool. Supplementary Table S5: List of positively (red) or negatively (blue) enriched gene sets in pPCLs with t(11;14) compared to MM cases with t(11;14), by GSEA analysis on pre-ranked 628 protein-coding gene list. Gene sets (version 7.1) are selected according to nominal *p*-value (<0.05), and ordered according to Normalized Enrichment Score (NES) in each gene set collection. Size, NES, Nominal *p*-value, and FDR *q*-value are reported for each gene set. Supplementary Table S6: Selected gene sets (shown in Figure 2) obtained by using Gene Set Enrichment Analysis on the list of 628 DE protein-coding genes that were ranked based on fold change values (NES > 1.5 or < −1.5; *p*-value < 0.05, NES: Normalized Enrichment Score). Supplementary Table S7: List of 31 differentially expressed lncRNAs in 15 pPCLs compared to 50 MM cases by SAM analysis, at *q*-value = 0. Transcripts are ordered according to SAM (d) score. Supplementary Table S8: List of 33 differentially expressed lncRNAs in 7 t(11;14)-pPCLs compared to 12 t(11;14)-MM cases by SAM analysis, at *q*-value = 0. Transcripts are ordered according to SAM (d) score. Supplementary Table S9: Results of Pearson’s correlation between the expression levels of the 33 lncRNAs and the overlapping/nearby transcripts globally profiled in 774 MM CoMMpass cohort. In bold, those lncRNA-gene pairs with at least 0.5 significant Pearson’s correlation. BH adjustment was applied. Supplementary Table S10: Results of Cox regression multivariate analysis for selected lncRNAs in high versus low expression group, together with significant clinical and molecular variables respectively in PFS and OS, in 497 MM patients of CoMMpass cohort. The number (N) and percentage of MM cases at higher expression levels are reported for each lncRNA. Hazard Ratio (HR) and 95% Confidence Interval (95% CI) are reported for each variable. Global *p*-value from Log-Rank test is also indicated for each analysis. Supplementary Table S11: Fisher’s exact test measuring the correlation between low and high SNHG6 expression level and the occurrence of main IgH translocations (trx), copy number alterations (CNAs), and non-synonymous somatic mutations in recurrently mutated genes in CoMMpass MM cases. del(13q) refers to 13q14, 13q34 cytobands or RB1 locus; 1q gain to 1q21 cytoband; del(1p) to 1p22 or CDKN2C locus; del(17p) to 17p13 or TP53 locus; HD to hyperdiploid condition. The total number of MM samples analyzed for each kind of data is reported. Significant adjusted *p*-values (<0.05) are in bold.
