# Supplementary material for: Transcriptomic Analysis in Multiple Myeloma and Primary Plasma Cell Leukemia with t(11;14) Reveals Different Expression Patterns with Biological Implications in Venetoclax Sensitivity
Source: Cancers (Basel). 2021 Sep 29;13(19):4898. doi: 10.3390/cancers13194898 (PMC8508148; doi:10.3390/cancers13194898)
Supplement: Supplementary file 1 [file cancers-13-04898-s001.zip › cancers-1395098-supplementary.pdf]

SUPPLEMENTARY METHODS

Quantitative real-rime PCR (qRT-PCR)

qRT-PCR was performed as previously described [1]. Primers used for the analyses are as follow.

Primer name	Sequence (5'-3')
BCL2 forward	5' – TTGTAATTCATCTGCCGCCG – 3'
BCL2 reverse	5' – CAATGAATCAGGAGTCGCGG – 3'
BCL2L1 forward	5' – CCCCTTAGCCTCCCTGAAAA – 3'
BCL2L1 reverse	5' – GAGTCCTGGTCCTTGCATCT – 3'
MCL1 forward	5' – CACCACCACCAAAACCAGTT – 3'
MCL1 reverse	5' – GTCCTCCTCCATAGCTTCCC – 3'
CD79A forward	5' – GCAACGAGTCATACCAGCAG – 3'
CD79A reverse	5' – TATTCATCCCCGGCATCCAA – 3'
MS4A1 forward	5' – AGGATGTCTTCACTGGTGGG – 3'
MS4A1 reverse	5' – ACACAGTCACACAGATGGGT – 3'
BEND5 forward	5' – CATCTGGGAAGCGGGATTTG – 3'
BEND5 reverse	5' – GAGAGGGGTGGTTTAGGGAC – 3'
SORT1 forward	5' – GGTGACTGACTGTTCCCTGA – 3'
SORT1 reverse	5' – ACCACATGCTCAGAAAGGGA – 3'
GAPDH forward	5' – ACAGTCAGCCGCATCTTCTT – 3'
GAPDH reverse	5' – AATGAAGGGGTCATTGATGG – 3'

Multi-Omics Data in CoMMpass Study

Multi-omics data about bone marrow MM samples at baseline (BM_1) were freely accessible from MMRF CoMMpass Study (https://research.themmrf.org/) and retrieved from the Interim Analysis 15a (MMRF_CoMMpass_IA15a).

Information about t(11;14) occurrence was inferred from CCND1 spike expression estimates from RNA-sequencing (RNA-seq) data (MMRF_CoMMpass_IA15a_RNAseq_Canonical_Ig_Translocations) and Transcript per Million (TPM) reads values of the investigated transcripts were obtained by Salmon gene expression quantification (MMRF_CoMMpass_IA15a_E74GTF_Salmon_V7.2_Filtered_Gene_TPM) in 774 BM_1 MM patients. Clinical data regarding Overall Survival (OS) and Progression free Survival (PFS) were considered in 767 MM patients for which both RNA-seq expression and survival data were available. Non-synonymous (NS) somatic mutation variants and counts data were obtained

from whole exome sequencing (WES) analyses, main IgH translocations were inferred from RNA-seq spike expression estimates of known target genes and Copy Number Alteration (CNA) data were retrieved by means of Next generation Sequencing (NGS)-based fluorescence in situ hybridization (FISH) [2] in 497 MM cases for which all data were available [3].

The presence of a specific CNA was considered when occurring in at least one of the investigated cytoband at a 20 percent cut-off for each considered chromosomal aberration, as previously reported [3].

Survival and statistical analyses

Survival analyses were performed using survival and survminer packages in R Bioconductor (version 4.0.0). Kaplan-Meier analysis was applied on OS and PFS data in 767 MM cohort stratified in high/low expression group according to median cut-off on lncRNAs expression levels. Log-Rank test p-value was calculated to measure the global difference between survival curves. The number of samples at risk in each group across time was calculated.

Cox proportional hazards model was applied as univariate analysis on single molecular variables and International Staging System (ISS) groups in relation to OS and PFS data, in 497 MM cases for which all information were accessible and significant features with p-value < 0.05 were tested by Cox regression multivariate analysis.

Wilcoxon and Kruskal-Wallis tests were applied to assess the differences in gene expression levels between two or more groups. Benjamini-Hochberg correction was applied to global p-values. Dunn's test was used to assess pairwise comparisons between multiple groups. Fisher's exact test was performed to verify the association of high/low lncRNA expression groups with main molecular aberrations investigated in CoMMpass cohort.

Functional annotation enrichment analysis

Gene Set Enrichment Analysis (GSEA) [4] was performed under default conditions on pre-ranked protein coding gene list based on fold-change values. Hallmark, Reactome, KEGG and C2-Gene Perturbations, filtered for Multiple Myeloma term and Gene Ontology (GO) gene sets (version 7.1), were selected on the base of nominal p-value < 0.05.

Cluster Profiler analysis [5] was performed in R environment on DE global protein coding gene list and the top (BH adjusted pvalue) GSEA gene sets, based on Gene-Ontology (GO) Biological Process (BP) terms, were selected. Cnetplot function was applied to depict the linkages of genes and biological GO terms as a network.

Functional enrichment analysis by means of the database for annotation, visualization and integrated discovery (DAVID) 6.8 [6], which can be freely accessed at http://david.abcc.ncifcrf.gov/, was performed at high stringency by means of functional annotation clustering on GO terms. Significant annotation clusters were selected on the base of Enrichment Score >1.3 and representative GO annotation terms were reported for each cluster.

1. Ronchetti D, Todoerti K, Vinci C, Favasuli V, Agnelli L, Manzoni M, Pelizzoni F, Chiaramonte R, Platonova N, Giuliani N, Tassone P, Amodio N, Neri A, Taiana E. Expression Pattern and Biological Significance of the lncRNA ST3GAL6-AS1 in Multiple Myeloma. Cancers (Basel). 2020 Mar 25;12(4):782. doi: 10.3390/cancers12040782.
2. Miller, C. et al. A comparison of clinical FISH and sequencing based FISH estimates in multiple myeloma: An MMRF CoMMpass analysis. In: Hematology TASo, editor. The American Society of Hematology; 2016: Blood; 2016. p. 374.
3. Todoerti K, Ronchetti D, Favasuli V, Maura F, Morabito F, Bolli N, Taiana E, Neri A. DIS3 mutations in multiple myeloma impact the transcriptional signature and clinical outcome. Haematologica. 2021
4. Subramanian A, Tamayo P, Mootha VK, et al. Gene set enrichment analysis: a knowledge-based approach for interpreting genome-wide expression profiles. PNAS. 2005; 102(43):15545-50.
5. Yu G, Wang L, Han Y, He Q (2012). “clusterProfiler: an R package for comparing biological themes among gene clusters.” OMICS: A Journal of Integrative Biology, 16(5), 284-287.
6. Huang DW, Sherman BT, Lempicki RA. Systematic and integrative analysis of large gene lists using DAVID Bioinformatics Resources. Nature Protoc. 2009;4(1):44-57.

Supplementary Table S1. List of 2416 differentially expressed coding genes in 7 t(11;14)-pPCL compared to 12 t(11;14)-MM cases, by SAM analysis at FDR<10%. Transcripts are ordered according to SAM (d) score.

ensembl gene	gene symbol	Score(d)	Fold Change
ENSG00000110321	EIF4G2	8,09	2,05
ENSG00000257103	LSM14A	6,51	3,16
ENSG00000101182	PSMA7	6,31	3,54
ENSG00000159459	UBR1	6,20	2,26
ENSG00000105849	TWISTNB	5,67	3,06
ENSG00000108107	RPL28	5,63	2,21
ENSG00000131711	MAP1B	5,60	15,76
ENSG00000101391	CDK5RAP1	5,46	1,85
ENSG00000137055	PLAA	5,43	2,80
ENSG00000183484	GPR132	5,34	2,80
ENSG00000161057	PSMC2	5,13	1,91
ENSG00000115053	NCL	5,11	1,88
ENSG00000101367	MAPRE1	5,10	1,69
ENSG00000164211	STARD4	5,10	2,14
ENSG00000198522	GPN1	5,07	2,05
ENSG00000141385	AFG3L2	5,02	1,92
ENSG00000137955	RABGGTB	5,01	1,90
ENSG00000185246	PRPF39	4,93	1,81
ENSG00000080824	HSP90AA1	4,93	2,94
ENSG00000181061	HIGD1A	4,91	2,56
ENSG00000101695	RNF125	4,91	5,94
ENSG00000164219	PGGT1B	4,86	2,22
ENSG00000177917	ARL6IP6	4,78	1,99
ENSG00000163714	U2SURP	4,76	1,75
ENSG00000155959	VBP1	4,71	2,03
ENSG00000112306	RPS12	4,71	4,02
ENSG00000211460	TSN	4,70	1,85
ENSG00000198677	TTC37	4,69	2,23
ENSG00000108055	SMC3	4,68	1,64
ENSG00000174839	DENND6A	4,67	2,04
ENSG00000133142	TCEAL4	4,66	2,14
ENSG00000173545	ZNF622	4,65	2,34
ENSG00000123136	DDX39A	4,63	2,33
ENSG00000085274	MYNN	4,62	1,76
ENSG00000143344	RGL1	4,61	5,56
ENSG00000105379	ETFB	4,55	2,15
ENSG00000008018	PSMB1	4,53	1,64
ENSG00000115541	HSPE1	4,49	4,20
ENSG00000072609	CHFR	4,49	1,63
ENSG00000072864	NDE1	4,48	2,00
ENSG00000188886	ASTL	4,45	1,83
ENSG00000152332	UHMK1	4,44	1,92
ENSG00000130935	NOL11	4,44	2,00
ENSG00000169251	NMD3	4,43	1,79
ENSG00000156515	HK1	4,43	2,87

ensembl gene	gene symbol	Score(d)	Fold Change
ENSG00000154723	ATP5J	4,41	2,42
ENSG00000198951	NAGA	4,41	2,21
ENSG00000241685	ARPC1A	4,41	2,21
ENSG00000078043	PIAS2	4,40	1,98
ENSG00000131746	TNS4	4,39	2,64
ENSG00000100473	COCH	4,38	4,15
ENSG00000035681	NSMAF	4,37	1,75
ENSG00000114115	RBP1	4,32	5,63
ENSG00000180957	PITPNB	4,32	2,41
ENSG00000170759	KIF5B	4,32	2,10
ENSG00000173486	FKBP2	4,31	3,09
ENSG00000060762	MPC1	4,28	1,81
ENSG00000120437	ACAT2	4,26	2,32
ENSG00000092439	TRPM7	4,26	1,65
ENSG00000152133	GPATCH11	4,25	1,70
ENSG00000156976	EIF4A2	4,24	3,99
ENSG00000165280	VCP	4,21	1,44
ENSG00000005075	POLR2J	4,20	1,94
ENSG00000077147	TM9SF3	4,18	1,37
ENSG00000138231	DBR1	4,18	1,72
ENSG00000137776	SLTM	4,18	1,76
ENSG00000183340	JRKL	4,17	1,79
ENSG00000114062	UBE3A	4,17	1,52
ENSG00000164091	WDR82	4,15	1,60
ENSG00000105329	TGFB1	4,15	2,24
ENSG00000085978	ATG16L1	4,12	1,94
ENSG00000114416	FXR1	4,12	1,69
ENSG00000256053	APOPT1	4,11	2,23
ENSG00000101928	MOSPD1	4,09	1,63
ENSG00000274523	RCC1L	4,09	1,41
ENSG00000244624	KRTAP20-1	4,08	3,16
ENSG00000134697	GNL2	4,08	2,03
ENSG00000147251	DOCK11	4,07	2,58
ENSG00000083168	KAT6A	4,04	1,45
ENSG00000099783	HNRNPM	4,04	1,83
ENSG00000165219	GAPVD1	4,03	1,53
ENSG00000147155	EBP	4,02	1,88
ENSG00000134313	KIDINS220	4,02	2,23
ENSG00000170242	USP47	4,01	2,36
ENSG00000198836	OPA1	4,01	1,79
ENSG00000165644	COMTD1	4,00	1,72
ENSG00000158669	GPAT4	4,00	1,65
ENSG00000176485	PLA2G16	4,00	3,22
ENSG00000092201	SUPT16H	3,99	1,60
ENSG00000165650	PDZD8	3,98	1,94
ENSG00000260286	C6orf229	3,98	1,44
ENSG00000140829	DHX38	3,98	1,47
ENSG00000125352	RNF113A	3,95	1,61
ENSG00000196141	SPATS2L	3,94	3,48

ensembl gene	gene symbol	Score(d)	Fold Change
ENSG00000064419	TNPO3	3,94	1,51
ENSG00000236334	PPIAL4G	3,93	1,94
ENSG00000130489	SCO2	3,93	1,59
ENSG00000144567	FAM134A	3,92	1,92
ENSG00000196705	ZNF431	3,92	1,72
ENSG00000182552	RWDD4	3,90	3,10
ENSG00000204659	CBY3	3,89	1,74
ENSG00000067167	TRAM1	3,89	1,39
ENSG00000167553	TUBA1C	3,88	1,46
ENSG00000101146	RAE1	3,88	1,62
ENSG00000180185	FAHD1	3,88	1,50
ENSG00000236637	IFNA4	3,87	1,84
ENSG00000173598	NUDT4	3,86	2,22
ENSG00000183648	NDUFB1	3,86	3,77
ENSG00000185627	PSMD13	3,85	2,13
ENSG00000150779	TIMM8B	3,85	1,97
ENSG00000113141	IK	3,85	1,89
ENSG00000147140	NONO	3,83	1,71
ENSG00000072182	ASIC4	3,83	1,38
ENSG00000128534	LSM8	3,83	1,79
ENSG00000109445	ZNF330	3,82	1,95
ENSG00000118242	MREG	3,82	2,65
ENSG00000095485	CWF19L1	3,81	1,58
ENSG00000137404	NRM	3,81	1,82
ENSG00000197323	TRIM33	3,80	1,59
ENSG00000088320	REM1	3,80	1,31
ENSG00000105639	JAK3	3,79	2,31
ENSG00000105409	ATP1A3	3,78	2,32
ENSG00000130827	PLXNA3	3,78	2,14
ENSG00000166200	COPS2	3,78	1,68
ENSG00000007168	PAFAH1B1	3,77	1,67
ENSG00000164944	KIAA1429	3,76	1,81
ENSG00000137154	RPS6	3,76	2,04
ENSG00000103342	GSPT1	3,76	2,30
ENSG00000116704	SLC35D1	3,75	1,85
ENSG00000236543	RP11-98L5.5	3,74	1,74
ENSG00000115963	RND3	3,74	7,35
ENSG00000125726	CD70	3,74	1,60
ENSG00000163041	H3F3A	3,73	2,08
ENSG00000196550	FAM72A	3,73	1,65
ENSG00000115816	CEBPZ	3,73	1,94
ENSG00000102001	CACNA1F	3,73	2,11
ENSG00000118804	STBD1	3,72	1,84
ENSG00000165194	PCDH19	3,72	2,03
ENSG00000163618	CADPS	3,71	5,38
ENSG00000100243	CYB5R3	3,71	1,53
ENSG00000112081	SRSF3	3,71	1,34
ENSG00000196950	SLC39A10	3,69	1,89
ENSG00000137073	UBAP2	3,69	1,48

ensembl gene	gene symbol	Score(d)	Fold Change
ENSG00000214309	MBLAC1	3,69	2,49
ENSG00000167720	SRR	3,68	1,84
ENSG00000007520	TSR3	3,68	1,90
ENSG00000163162	RNF149	3,67	2,37
ENSG00000173692	PSMD1	3,67	1,47
ENSG00000132823	OSER1	3,65	2,13
ENSG00000240065	PSMB9	3,65	2,40
ENSG00000101844	ATG4A	3,65	1,69
ENSG00000163584	RPL22L1	3,64	3,82
ENSG00000105258	POLR2I	3,64	1,70
ENSG00000204388	HSPA1B	3,64	5,03
ENSG00000197540	GZMM	3,64	1,40
ENSG00000155380	SLC16A1	3,63	2,24
ENSG00000119041	GTF3C3	3,62	1,93
ENSG00000144741	SLC25A26	3,62	1,59
ENSG00000187170	LCE4A	3,62	1,42
ENSG00000128191	DGCR8	3,61	1,79
ENSG00000068366	ACSL4	3,61	2,64
ENSG00000110660	SLC35F2	3,61	2,21
ENSG00000125944	HNRNPR	3,61	1,80
ENSG00000136718	IMP4	3,61	1,75
ENSG00000197647	ZNF433	3,61	4,22
ENSG00000133328	HRASLS2	3,61	3,31
ENSG00000136930	PSMB7	3,60	1,68
ENSG00000166226	CCT2	3,60	2,19
ENSG00000042286	AIFM2	3,59	1,53
ENSG00000162645	GBP2	3,58	7,63
ENSG00000143774	GUK1	3,58	1,46
ENSG00000158526	TSR2	3,57	1,60
ENSG00000120438	TCP1	3,57	1,50
ENSG00000175110	MRPS22	3,57	1,65
ENSG00000187189	TSPYL4	3,57	1,37
ENSG00000121691	CAT	3,56	2,03
ENSG00000151917	BEND6	3,55	1,90
ENSG00000135269	TES	3,55	2,24
ENSG00000152936	LMNTD1	3,55	1,79
ENSG00000110628	SLC22A18	3,55	1,77
ENSG00000037474	NSUN2	3,55	1,80
ENSG00000214706	IFRD2	3,55	1,66
ENSG00000118454	ANKRD13C	3,54	1,63
ENSG00000171102	OBP2B	3,54	2,23
ENSG00000163904	SENP2	3,54	1,77
ENSG00000171960	PPIH	3,54	1,63
ENSG00000125454	SLC25A19	3,54	1,98
ENSG00000177432	NAP1L5	3,53	1,44
ENSG00000166479	TMX3	3,53	1,69
ENSG00000166311	SMPD1	3,53	2,04
ENSG00000186792	HYAL3	3,53	1,65
ENSG00000177370	TIMM22	3,53	1,66

ensembl gene	gene symbol	Score(d)	Fold Change
ENSG00000174749	C4orf32	3,53	2,22
ENSG00000198231	DDX42	3,53	1,68
ENSG00000151470	C4orf33	3,52	2,85
ENSG00000185947	ZNF267	3,52	2,71
ENSG00000241258	CRCP	3,52	1,90
ENSG00000162433	AK4	3,50	2,00
ENSG00000111142	METAP2	3,50	1,58
ENSG00000198034	RPS4X	3,50	1,47
ENSG00000134186	PRPF38B	3,50	1,76
ENSG00000068903	SIRT2	3,49	2,17
ENSG00000275700	AATF	3,49	2,09
ENSG00000265241	RBM8A	3,49	1,97
ENSG00000086061	DNAJA1	3,49	2,05
ENSG00000164615	CAMLG	3,49	1,50
ENSG00000013364	MVP	3,49	3,30
ENSG00000179950	PUF60	3,48	1,61
ENSG00000204843	DCTN1	3,48	1,60
ENSG00000145022	TCTA	3,47	1,69
ENSG00000186577	C6orf1	3,47	1,60
ENSG00000149136	SSRP1	3,46	2,38
ENSG00000178287	SPAG11A	3,46	2,18
ENSG00000103257	SLC7A5	3,45	2,53
ENSG00000183527	PSMG1	3,45	1,92
ENSG00000196072	BLOC1S2	3,45	2,03
ENSG00000082153	BZW1	3,44	1,91
ENSG00000175416	CLTB	3,44	1,74
ENSG00000129083	COPB1	3,44	3,51
ENSG00000171566	PLRG1	3,43	2,02
ENSG00000237190	CDKN2AIPNL	3,43	1,70
ENSG00000087510	TFAP2C	3,43	1,57
ENSG00000175105	ZNF654	3,43	1,69
ENSG00000163518	FCRL4	3,43	1,87
ENSG00000162368	CMPK1	3,41	1,45
ENSG00000176531	PHLDB3	3,41	1,66
ENSG00000099624	ATP5D	3,41	1,59
ENSG00000171497	PPID	3,41	1,98
ENSG00000166710	B2M	3,41	1,52
ENSG00000069509	FUNDC1	3,39	1,61
ENSG00000109586	GALNT7	3,39	1,84
ENSG00000179059	ZFP42	3,39	1,52
ENSG00000151576	QTRT2	3,39	1,65
ENSG00000107581	EIF3A	3,39	1,40
ENSG00000197106	SLC6A17	3,39	1,65
ENSG00000155622	XAGE2	3,39	1,36
ENSG00000185633	NDUFA4L2	3,38	1,91
ENSG00000277791	PSMB3	3,37	1,70
ENSG00000123892	RAB38	3,37	1,67
ENSG00000273540	AGBL1	3,37	1,50
ENSG00000198695	MT-ND6	3,37	2,07

ensembl gene	gene symbol	Score(d)	Fold Change
ENSG00000162783	IER5	3,36	2,24
ENSG00000204264	PSMB8	3,36	3,38
ENSG00000165521	EML5	3,34	2,49
ENSG00000116852	KIF21B	3,33	1,89
ENSG00000196924	FLNA	3,33	2,72
ENSG00000151778	SERP2	3,32	1,72
ENSG00000272899	RP11-309L24.4	3,32	1,73
ENSG00000134802	SLC43A3	3,32	2,64
ENSG00000154518	ATP5G3	3,31	1,76
ENSG00000169436	COL22A1	3,31	1,83
ENSG00000105851	PIK3CG	3,30	2,26
ENSG00000110075	PPP6R3	3,30	1,38
ENSG00000067533	RRP15	3,30	1,81
ENSG00000164209	SLC25A46	3,30	1,56
ENSG00000196214	ZNF766	3,30	1,96
ENSG00000013588	GPRC5A	3,30	2,31
ENSG00000142227	EMP3	3,29	1,70
ENSG00000100796	PPP4R3A	3,29	1,65
ENSG00000147381	MAGEA4	3,29	1,65
ENSG00000221989	OR2A2	3,29	1,68
ENSG00000160799	CCDC12	3,29	1,75
ENSG00000100867	DHRS2	3,29	1,31
ENSG00000173432	SAA1	3,29	1,81
ENSG00000228607	CLDN25	3,28	1,32
ENSG00000278540	ACACA	3,28	1,72
ENSG00000168454	TXNDC2	3,28	1,42
ENSG00000113575	PPP2CA	3,28	2,00
ENSG00000127328	RAB3IP	3,28	1,69
ENSG00000103489	XYLT1	3,28	4,55
ENSG00000092199	HNRNPC	3,27	1,30
ENSG00000137251	TINAG	3,27	1,62
ENSG00000204370	SDHD	3,27	2,09
ENSG00000274068	RP11-475E11.9	3,27	1,64
ENSG00000099800	TIMM13	3,27	2,60
ENSG00000197969	VPS13A	3,27	1,62
ENSG00000138495	COX17	3,26	2,54
ENSG00000078596	ITM2A	3,26	2,02
ENSG00000100056	DGCR14	3,26	1,43
ENSG00000205189	ZBTB10	3,26	3,05
ENSG00000166888	STAT6	3,24	1,65
ENSG00000178586	OR6B3	3,24	1,44
ENSG00000255302	EID1	3,24	1,57
ENSG00000213231	TCL1B	3,24	3,46
ENSG00000115840	SLC25A12	3,24	1,80
ENSG00000169567	HINT1	3,23	1,50
ENSG00000175868	CALCB	3,23	2,09
ENSG00000170791	CHCHD7	3,23	1,95
ENSG00000126460	PRRG2	3,23	1,31
ENSG00000085788	DDHD2	3,23	1,47

ensembl gene	gene symbol	Score(d)	Fold Change
ENSG00000266412	NCOA4	3,22	2,15
ENSG00000172893	DHCR7	3,22	1,74
ENSG00000104894	CD37	3,22	2,87
ENSG00000129473	BCL2L2	3,22	1,90
ENSG00000177684	DEFB114	3,22	1,46
ENSG00000100316	RPL3	3,22	1,63
ENSG00000176920	FUT2	3,21	1,42
ENSG00000181744	C3orf58	3,21	2,25
ENSG00000189046	ALKBH2	3,20	2,08
ENSG00000023228	NDUFS1	3,20	1,55
ENSG00000005243	COPZ2	3,20	2,59
ENSG00000116183	PAPPA2	3,19	1,32
ENSG00000128641	MYO1B	3,19	3,34
ENSG00000214194	LINC00998	3,19	1,82
ENSG00000100297	MCM5	3,19	1,60
ENSG00000136754	ABI1	3,19	1,52
ENSG00000102271	KLHL4	3,19	5,79
ENSG00000136630	HLX	3,18	2,18
ENSG00000056097	ZFR	3,18	1,64
ENSG00000204314	PRRT1	3,18	1,58
ENSG00000108671	PSMD11	3,17	1,35
ENSG00000107593	PKD2L1	3,17	1,49
ENSG00000174780	SRP72	3,16	1,35
ENSG00000135404	CD63	3,16	2,02
ENSG00000138785	INTS12	3,16	1,52
ENSG00000171931	FBXW10	3,15	1,39
ENSG00000214954	LRRC69	3,15	1,59
ENSG00000238205	MPC1L	3,14	1,27
ENSG00000123144	C19orf43	3,14	1,70
ENSG00000160185	UBASH3A	3,14	1,22
ENSG00000165637	VDAC2	3,14	1,34
ENSG00000169126	ARMC4	3,14	2,63
ENSG00000213638	ADAT3	3,14	1,70
ENSG00000119285	HEATR1	3,14	1,97
ENSG00000104885	DOT1L	3,14	1,39
ENSG00000057757	PITHD1	3,14	1,48
ENSG00000125492	BARHL1	3,13	1,37
ENSG00000018510	AGPS	3,13	1,60
ENSG00000144681	STAC	3,13	1,69
ENSG00000172175	MALT1	3,13	1,82
ENSG00000136536	42801	3,13	1,68
ENSG00000143815	LBR	3,13	1,90
ENSG00000124191	TOX2	3,12	2,22
ENSG00000137876	RSL24D1	3,12	1,87
ENSG00000147724	FAM135B	3,12	1,63
ENSG00000131668	BARX1	3,12	1,31
ENSG00000046774	MAGEC2	3,12	1,53
ENSG00000204387	C6orf48	3,11	1,85
ENSG00000115761	NOL10	3,11	1,59

ensembl gene	gene symbol	Score(d)	Fold Change
ENSG00000159961	OR3A3	3,11	1,56
ENSG00000196131	VN1R2	3,11	1,84
ENSG00000134160	TRPM1	3,11	1,28
ENSG00000153574	RPIA	3,11	1,57
ENSG00000129910	CDH15	3,11	1,51
ENSG00000172262	ZNF131	3,10	1,47
ENSG00000183166	CALN1	3,10	1,44
ENSG00000157322	CLEC18A	3,10	1,36
ENSG00000105671	DDX49	3,09	1,56
ENSG00000110243	APOA5	3,09	1,27
ENSG00000041802	LSG1	3,09	1,58
ENSG00000055044	NOP58	3,09	1,55
ENSG00000115170	ACVR1	3,08	1,80
ENSG00000168209	DDIT4	3,08	2,76
ENSG00000112972	HMGCS1	3,08	1,68
ENSG00000023191	RNH1	3,08	1,88
ENSG00000100097	LGALS1	3,07	2,05
ENSG00000125457	MIF4GD	3,07	1,75
ENSG00000163636	PSMD6	3,07	1,56
ENSG00000132872	SYT4	3,07	1,48
ENSG00000031823	RANBP3	3,07	1,24
ENSG00000060982	BCAT1	3,07	4,43
ENSG00000108061	SHOC2	3,07	1,43
ENSG00000141543	EIF4A3	3,07	1,93
ENSG00000114784	EIF1B	3,07	2,09
ENSG00000179010	MRFAP1	3,06	1,89
ENSG00000139977	NAA30	3,06	1,56
ENSG00000125970	RALY	3,05	1,93
ENSG00000119541	VPS4B	3,05	1,66
ENSG00000145365	TIFA	3,04	1,81
ENSG00000113810	SMC4	3,04	1,63
ENSG00000196660	SLC30A10	3,04	1,25
ENSG00000095574	IKZF5	3,04	1,64
ENSG00000130254	SAFB2	3,04	1,84
ENSG00000176410	DNAJC30	3,03	1,32
ENSG00000153879	CEBPG	3,03	1,69
ENSG00000130703	OSBPL2	3,03	1,38
ENSG00000105699	LSR	3,03	1,72
ENSG00000087266	SH3BP2	3,03	2,00
ENSG00000198271	KRTAP4-5	3,03	1,47
ENSG00000112031	MTRF1L	3,03	1,88
ENSG00000113758	DBN1	3,03	2,01
ENSG00000100629	CEP128	3,03	2,14
ENSG00000115267	IFIH1	3,02	1,83
ENSG00000269556	TMEM185A	3,02	1,50
ENSG00000089234	BRAP	3,02	1,51
ENSG00000111727	HCFC2	3,02	2,50
ENSG00000163900	TMEM41A	3,02	1,80
ENSG00000165733	BMS1	3,02	1,52

ensembl gene	gene symbol	Score(d)	Fold Change
ENSG00000138794	CASP6	3,01	1,78
ENSG00000215301	DDX3X	3,01	1,61
ENSG00000048544	MRPS10	3,01	1,57
ENSG00000139697	SBNO1	3,01	1,52
ENSG00000102241	HTATSF1	3,01	1,60
ENSG00000170881	RNF139	3,00	1,48
ENSG00000118513	MYB	3,00	2,29
ENSG00000101670	LIPG	3,00	1,80
ENSG00000068308	OTUD5	3,00	1,44
ENSG00000134987	WDR36	3,00	1,59
ENSG00000087191	PSMC5	3,00	2,02
ENSG00000141076	UTP4	3,00	1,99
ENSG00000150676	CCDC83	2,99	1,46
ENSG00000186868	MAPT	2,99	1,35
ENSG00000107164	FUBP3	2,99	1,34
ENSG00000101972	STAG2	2,99	1,56
ENSG00000091436	AC013461.1	2,99	2,21
ENSG00000184575	XPOT	2,98	2,03
ENSG00000164175	SLC45A2	2,98	1,74
ENSG00000147526	TACC1	2,98	3,09
ENSG00000075975	MKRN2	2,98	1,46
ENSG00000168539	CHRM1	2,98	1,28
ENSG00000183747	ACSM2A	2,97	1,61
ENSG00000183520	UTP11	2,97	1,66
ENSG00000079134	THOC1	2,97	1,47
ENSG00000163541	SUCLG1	2,97	1,62
ENSG00000171987	C11orf40	2,97	1,22
ENSG00000196361	ELAVL3	2,96	1,71
ENSG00000151729	SLC25A4	2,96	2,23
ENSG00000170619	COMMD5	2,96	1,35
ENSG00000144895	EIF2A	2,96	1,60
ENSG00000103266	STUB1	2,95	1,60
ENSG00000197885	NKIRAS1	2,95	2,41
ENSG00000181826	RELL1	2,95	2,04
ENSG00000186509	OR9Q1	2,95	1,51
ENSG00000158715	SLC45A3	2,95	1,40
ENSG00000169488	OR4K15	2,95	1,29
ENSG00000103978	TMEM87A	2,95	1,55
ENSG00000065978	YBX1	2,95	1,58
ENSG00000106397	PLOD3	2,95	2,04
ENSG00000266338	NBPF15	2,94	1,72
ENSG00000109193	SULT1E1	2,94	1,39
ENSG00000076003	MCM6	2,94	1,95
ENSG00000157045	NTAN1	2,94	2,10
ENSG00000133321	RARRES3	2,94	3,33
ENSG00000165006	UBAP1	2,93	1,68
ENSG00000164172	MOCS2	2,93	1,99
ENSG00000161203	AP2M1	2,93	1,81
ENSG00000170088	TMEM192	2,93	1,49

ensembl gene	gene symbol	Score(d)	Fold Change
ENSG00000071537	SEL1L	2,93	1,63
ENSG00000206557	TRIM71	2,93	1,32
ENSG00000107960	OBFC1	2,93	1,63
ENSG00000101057	MYBL2	2,93	1,60
ENSG00000105193	RPS16	2,93	2,79
ENSG00000100003	SEC14L2	2,93	2,72
ENSG00000168438	CDC40	2,93	1,59
ENSG00000144029	MRPS5	2,93	1,44
ENSG00000158615	PPP1R15B	2,93	1,50
ENSG00000179826	MRGPRX3	2,93	1,49
ENSG00000269404	SPIB	2,93	2,10
ENSG00000181264	TMEM136	2,92	2,60
ENSG00000197125	OR8B8	2,92	1,36
ENSG00000115339	GALNT3	2,92	4,58
ENSG00000166012	TAF1D	2,92	2,02
ENSG00000137100	DCTN3	2,92	1,82
ENSG00000187231	SESTD1	2,92	4,43
ENSG00000162520	SYNC	2,92	1,36
ENSG00000184162	NR2C2AP	2,92	1,50
ENSG00000186480	INSIG1	2,92	2,06
ENSG00000140105	WARS	2,92	1,98
ENSG00000188372	ZP3	2,91	2,26
ENSG00000130766	SESN2	2,91	2,59
ENSG00000188295	ZNF669	2,91	1,39
ENSG00000163319	MRPS18C	2,91	1,48
ENSG00000171858	RPS21	2,91	2,50
ENSG00000163898	LIPH	2,91	3,62
ENSG00000102119	EMD	2,90	1,59
ENSG00000205186	FABP9	2,90	1,27
ENSG00000006007	GDE1	2,90	1,51
ENSG00000162909	CAPN2	2,90	2,51
ENSG00000172020	GAP43	2,90	1,38
ENSG00000197343	ZNF655	2,90	1,64
ENSG00000073584	SMARCE1	2,89	1,62
ENSG00000124134	KCNS1	2,89	1,23
ENSG00000061794	MRPS35	2,89	2,22
ENSG00000156697	UTP14A	2,89	1,40
ENSG00000007237	GAS7	2,89	2,63
ENSG00000206026	SMIM21	2,89	1,44
ENSG00000134198	TSPAN2	2,89	3,60
ENSG00000147274	RBMX	2,88	1,58
ENSG00000183891	TTC32	2,88	2,28
ENSG00000107625	DDX50	2,88	1,76
ENSG00000179051	RCC2	2,88	1,55
ENSG00000241123	KRTAP10-5	2,88	1,45
ENSG00000080709	KCNN2	2,87	1,50
ENSG00000138182	KIF20B	2,87	1,94
ENSG00000128609	NDUFA5	2,87	1,57
ENSG00000106245	BUD31	2,87	1,79

ensembl gene	gene symbol	Score(d)	Fold Change
ENSG00000158411	MITD1	2,87	1,45
ENSG00000031698	SARS	2,87	1,87
ENSG00000163743	RCHY1	2,86	1,66
ENSG00000109111	SUPT6H	2,86	1,49
ENSG00000261341	CTD-2568A17.1	2,86	1,44
ENSG00000123983	ACSL3	2,86	1,69
ENSG00000137818	RPLP1	2,86	1,72
ENSG00000171130	ATP6V0E2	2,86	1,51
ENSG00000115944	COX7A2L	2,85	1,91
ENSG00000134265	NAPG	2,85	1,42
ENSG00000170185	USP38	2,85	1,58
ENSG00000188725	SMIM15	2,85	1,27
ENSG00000206073	SERPINB4	2,85	1,41
ENSG00000176294	OR4N2	2,85	2,07
ENSG00000129654	FOXJ1	2,85	1,46
ENSG00000134597	RBMX2	2,85	1,58
ENSG00000101911	PRPS2	2,84	1,91
ENSG00000204161	C10orf128	2,84	1,78
ENSG00000105321	CCDC9	2,84	1,77
ENSG00000145632	PLK2	2,84	3,39
ENSG00000198431	TXNRD1	2,84	1,48
ENSG00000139343	SNRPF	2,84	1,73
ENSG00000144747	TMF1	2,84	1,65
ENSG00000130023	ERMARD	2,84	1,48
ENSG00000132792	CTNNBL1	2,84	1,55
ENSG00000213341	CHUK	2,83	1,77
ENSG00000075413	MARK3	2,83	1,31
ENSG00000183379	SYNDIG1L	2,83	1,39
ENSG00000112651	MRPL2	2,83	1,63
ENSG00000102195	GPR50	2,83	1,30
ENSG00000178498	DTX3	2,83	1,77
ENSG00000135249	RINT1	2,82	1,44
ENSG00000182698	RESP18	2,82	1,29
ENSG00000174775	HRAS	2,82	1,24
ENSG00000183287	CCBE1	2,82	1,41
ENSG00000142871	CYR61	2,82	1,51
ENSG00000215375	MYL5	2,82	1,39
ENSG00000101222	SPEF1	2,82	1,29
ENSG00000142544	CTU1	2,81	1,35
ENSG00000091039	OSBPL8	2,81	1,44
ENSG00000159259	CHAF1B	2,81	1,97
ENSG00000147874	HAUS6	2,81	1,61
ENSG00000158639	PAGE5	2,81	1,84
ENSG00000186838	SELV	2,81	1,22
ENSG00000170775	GPR37	2,81	2,20
ENSG00000105668	UPK1A	2,81	1,26
ENSG00000183378	OVCH2	2,80	1,33
ENSG00000259075	POC1B-GALNT4	2,80	2,10
ENSG00000198848	CES1	2,80	1,49

ensembl gene	gene symbol	Score(d)	Fold Change
ENSG00000130544	ZNF557	2,80	1,45
ENSG00000186806	VSIG10L	2,80	1,45
ENSG00000114209	PDCD10	2,79	1,66
ENSG00000176783	RUFY1	2,79	1,79
ENSG00000187824	TMEM220	2,79	1,89
ENSG00000168724	DNAJC21	2,79	1,33
ENSG00000155729	KCTD18	2,79	1,47
ENSG00000204642	HLA-F	2,79	1,39
ENSG00000159261	CLDN14	2,79	2,06
ENSG00000105507	CABP5	2,79	1,32
ENSG00000058729	RIOK2	2,79	1,50
ENSG00000160310	PRMT2	2,78	1,54
ENSG00000081014	AP4E1	2,78	1,42
ENSG00000147180	ZNF711	2,78	2,00
ENSG00000101474	APMAP	2,78	1,48
ENSG00000006125	AP2B1	2,78	1,66
ENSG00000171314	PGAM1	2,78	1,85
ENSG00000186795	KCNK18	2,78	1,24
ENSG00000074755	ZZEF1	2,78	1,51
ENSG00000182557	SPNS3	2,78	1,62
ENSG00000151552	QDPR	2,78	1,55
ENSG00000123728	RAP2C	2,77	1,48
ENSG00000155330	C16orf87	2,77	2,02
ENSG00000178965	ERICH3	2,77	1,70
ENSG00000197928	ZNF677	2,77	2,49
ENSG00000156127	BATF	2,77	1,44
ENSG00000171425	ZNF581	2,77	1,64
ENSG00000117984	CTSD	2,77	1,41
ENSG00000091140	DLD	2,77	1,78
ENSG00000177971	IMP3	2,77	1,42
ENSG00000184945	AQP12A	2,77	1,38
ENSG00000137824	RMDN3	2,77	1,31
ENSG00000115241	PPM1G	2,76	1,38
ENSG00000156261	CCT8	2,76	1,88
ENSG00000140374	ETFA	2,76	1,75
ENSG00000140612	SEC11A	2,76	1,92
ENSG00000123505	AMD1	2,76	1,52
ENSG00000108094	CUL2	2,76	1,42
ENSG00000166292	TMEM100	2,76	1,32
ENSG00000168890	TMEM150A	2,76	1,74
ENSG00000243910	TUBA4B	2,75	1,34
ENSG00000065621	GSTO2	2,75	1,95
ENSG00000123143	PKN1	2,75	1,67
ENSG00000017260	ATP2C1	2,75	1,56
ENSG00000176225	RTTN	2,74	2,41
ENSG00000010810	FYN	2,74	2,53
ENSG00000119718	EIF2B2	2,74	1,65
ENSG00000122565	CBX3	2,74	1,72
ENSG00000141437	SLC25A52	2,74	1,34

ensembl gene	gene symbol	Score(d)	Fold Change
ENSG00000104142	VPS18	2,74	1,39
ENSG00000214642	DEFB113	2,74	1,42
ENSG00000145912	NHP2	2,73	1,74
ENSG00000162607	USP1	2,73	1,64
ENSG00000088986	DYNLL1	2,73	2,80
ENSG00000197019	SERTAD1	2,73	1,68
ENSG00000132436	FIGNL1	2,73	2,19
ENSG00000127603	MACF1	2,73	1,54
ENSG00000062524	LTK	2,72	1,91
ENSG00000187612	OR5W2	2,72	1,26
ENSG00000156232	WHAMM	2,72	1,85
ENSG00000165164	CFAP47	2,72	1,77
ENSG00000134077	THUMPD3	2,72	1,39
ENSG00000112130	RNF8	2,72	1,68
ENSG00000163634	THOC7	2,72	1,92
ENSG00000171295	ZNF440	2,72	1,42
ENSG00000155438	NIFK	2,72	1,75
ENSG00000108651	UTP6	2,72	1,60
ENSG00000180745	CLRN3	2,71	1,27
ENSG00000197417	SHPK	2,71	1,97
ENSG00000187516	HYPM	2,71	1,39
ENSG00000172421	EFCAB3	2,71	1,33
ENSG00000083937	CHMP2B	2,71	1,52
ENSG00000121680	PEX16	2,71	1,28
ENSG00000100941	PNN	2,71	1,71
ENSG00000138068	SULT6B1	2,71	1,48
ENSG00000157870	FAM213B	2,70	1,45
ENSG00000130707	ASS1	2,70	3,85
ENSG00000131203	IDO1	2,70	1,55
ENSG00000122971	ACADS	2,70	1,23
ENSG00000206172	HBA1	2,70	1,69
ENSG00000183735	TBK1	2,70	1,67
ENSG00000163754	GYG1	2,69	1,47
ENSG00000102034	ELF4	2,69	2,37
ENSG00000278053	DDX52	2,69	1,55
ENSG00000173660	UQCRH	2,69	2,05
ENSG00000114942	EEF1B2	2,69	1,87
ENSG00000159579	RSPRY1	2,69	1,58
ENSG00000283526	RP11-40A7.2	2,69	1,31
ENSG00000105617	LENG1	2,68	1,35
ENSG00000197386	HTT	2,68	1,58
ENSG00000151012	SLC7A11	2,68	4,53
ENSG00000166441	RPL27A	2,68	1,77
ENSG00000151338	MIPOL1	2,68	1,28
ENSG00000197790	OR52M1	2,68	1,33
ENSG00000123159	GIPC1	2,67	1,47
ENSG00000073067	CYP2W1	2,67	1,22
ENSG00000170802	FOXN2	2,67	1,61
ENSG00000111087	GLI1	2,67	1,28

ensembl gene	gene symbol	Score(d)	Fold Change
ENSG00000106459	NRF1	2,67	1,64
ENSG00000143878	RHOB	2,66	2,33
ENSG00000198898	CAPZA2	2,66	1,61
ENSG00000168297	PXK	2,66	2,33
ENSG00000132259	CNGA4	2,66	1,31
ENSG00000164647	STEAP1	2,66	1,64
ENSG00000145975	FAM217A	2,66	1,55
ENSG00000130772	MED18	2,66	1,32
ENSG00000164038	SLC9B2	2,66	1,63
ENSG00000140284	SLC27A2	2,66	1,90
ENSG00000023909	GCLM	2,65	1,72
ENSG00000114854	TNNC1	2,65	1,43
ENSG00000155744	FAM126B	2,65	1,56
ENSG00000003096	KLHL13	2,65	1,54
ENSG00000168488	ATXN2L	2,65	1,47
ENSG00000160948	VPS28	2,65	1,47
ENSG00000136710	CCDC115	2,65	1,28
ENSG00000173264	GPR137	2,65	1,40
ENSG00000076043	REXO2	2,64	2,35
ENSG00000148335	NTMT1	2,64	1,43
ENSG00000127334	DYRK2	2,64	2,39
ENSG00000021355	SERPINB1	2,64	1,53
ENSG00000160161	CILP2	2,64	1,37
ENSG00000129951	PLPPR3	2,64	1,23
ENSG00000186281	GPAT2	2,64	2,00
ENSG00000178952	TUFM	2,64	1,77
ENSG00000126698	DNAJC8	2,64	1,43
ENSG00000204520	MICA	2,64	1,56
ENSG00000033050	ABCF2	2,64	1,43
ENSG00000151923	TIAL1	2,64	1,24
ENSG00000154529	CNTNAP3B	2,64	1,37
ENSG00000171202	TMEM126A	2,64	1,97
ENSG00000157800	SLC37A3	2,63	2,05
ENSG00000101782	RIOK3	2,63	1,64
ENSG00000160445	ZER1	2,63	1,37
ENSG00000065883	CDK13	2,63	1,36
ENSG00000164128	NPY1R	2,63	1,53
ENSG00000185085	INTS5	2,63	1,48
ENSG00000147432	CHRNB3	2,63	1,32
ENSG00000060971	ACAA1	2,63	1,53
ENSG00000214367	HAUS3	2,63	2,16
ENSG00000249751	ECSCR	2,63	1,32
ENSG00000150456	EEF1AKMT1	2,63	1,34
ENSG00000204946	ZNF783	2,63	1,53
ENSG00000144802	NFKBIZ	2,63	2,55
ENSG00000163098	BIRC8	2,62	1,48
ENSG00000167461	RAB8A	2,62	1,58
ENSG00000138028	CGREF1	2,62	1,36
ENSG00000169155	ZBTB43	2,62	1,64

ensembl gene	gene symbol	Score(d)	Fold Change
ENSG00000105290	APLP1	2,62	1,23
ENSG00000132286	TIMM10B	2,62	1,47
ENSG00000222047	C10orf55	2,62	1,37
ENSG00000146757	ZNF92	2,62	1,76
ENSG00000145337	PYURF	2,62	1,51
ENSG00000033030	ZCCHC8	2,62	1,49
ENSG00000204887	KRTAP1-4	2,62	1,54
ENSG00000127125	PPCS	2,62	1,72
ENSG00000116473	RAP1A	2,62	1,64
ENSG00000116171	SCP2	2,61	1,75
ENSG00000074695	LMAN1	2,61	1,48
ENSG00000183569	SERHL2	2,61	1,89
ENSG00000164758	MED30	2,61	1,57
ENSG00000261857	MIA	2,61	2,93
ENSG00000222014	RAB6C	2,61	1,47
ENSG00000134982	APC	2,61	1,58
ENSG00000185730	ZNF696	2,61	1,33
ENSG00000152223	EPG5	2,61	1,31
ENSG00000136717	BIN1	2,61	1,75
ENSG00000179055	OR13D1	2,60	1,40
ENSG00000183048	SLC25A10	2,60	1,30
ENSG00000180573	HIST1H2AC	2,60	2,31
ENSG00000067900	ROCK1	2,60	1,41
ENSG00000160932	LY6E	2,60	3,05
ENSG00000074211	PPP2R2C	2,60	1,35
ENSG00000081307	UBA5	2,60	1,34
ENSG00000160993	ALKBH4	2,60	1,34
ENSG00000031081	ARHGAP31	2,59	1,89
ENSG00000133020	MYH8	2,59	1,39
ENSG00000172538	FAM170B	2,59	1,34
ENSG00000138802	SEC24B	2,59	1,36
ENSG00000112249	ASCC3	2,59	1,67
ENSG00000196420	S100A5	2,59	1,27
ENSG00000115758	ODC1	2,59	1,41
ENSG00000109832	DDX25	2,59	1,31
ENSG00000170369	CST2	2,59	1,34
ENSG00000188672	RHCE	2,58	1,53
ENSG00000167384	ZNF180	2,58	1,67
ENSG00000140320	BAHD1	2,58	1,53
ENSG00000105619	TFPT	2,58	1,48
ENSG00000239900	ADSL	2,58	1,75
ENSG00000162877	PM20D1	2,58	2,13
ENSG00000138685	FGF2	2,58	3,77
ENSG00000055332	EIF2AK2	2,58	1,55
ENSG00000015479	MATR3	2,58	1,56
ENSG00000141161	UNC45B	2,58	1,17
ENSG00000150787	PTS	2,58	1,42
ENSG00000135976	ANKRD36	2,58	1,96
ENSG00000221823	PPP3R1	2,58	1,41

ensembl gene	gene symbol	Score(d)	Fold Change
ENSG00000205856	C22orf42	2,57	1,30
ENSG00000178473	UCN3	2,57	1,37
ENSG00000088247	KHSRP	2,57	1,35
ENSG00000135655	USP15	2,57	1,41
ENSG00000155368	DBI	2,57	1,67
ENSG00000130598	TNNI2	2,57	1,34
ENSG00000114520	SNX4	2,57	1,80
ENSG00000147872	PLIN2	2,56	1,38
ENSG00000136960	ENPP2	2,56	4,23
ENSG00000075223	SEMA3C	2,56	1,64
ENSG00000100028	SNRPD3	2,56	1,72
ENSG00000130720	FIBCD1	2,56	1,46
ENSG00000090266	NDUFB2	2,56	1,43
ENSG00000130755	GMFG	2,56	1,75
ENSG00000131437	KIF3A	2,56	1,85
ENSG00000143971	ETAA1	2,56	1,66
ENSG00000093000	NUP50	2,56	1,66
ENSG00000111445	RFC5	2,56	1,54
ENSG00000113739	STC2	2,56	2,57
ENSG00000099785	MARCHF2	2,55	1,36
ENSG00000166780	C16orf45	2,55	1,46
ENSG00000139350	NEDD1	2,55	1,48
ENSG00000137831	UACA	2,55	1,64
ENSG00000176903	PNMA1	2,55	1,91
ENSG00000123737	EXOSC9	2,55	1,33
ENSG00000130764	LRRC47	2,55	1,43
ENSG00000197061	HIST1H4C	2,55	1,62
ENSG00000111450	STX2	2,55	1,88
ENSG00000239810	PRAMEF11	2,54	1,39
ENSG00000096384	HSP90AB1	2,54	1,51
ENSG00000107807	TLX1	2,54	1,53
ENSG00000049541	RFC2	2,54	1,57
ENSG00000132467	UTP3	2,54	1,64
ENSG00000033867	SLC4A7	2,54	1,73
ENSG00000162654	GBP4	2,54	4,46
ENSG00000078808	SDF4	2,54	1,40
ENSG00000174137	FAM53A	2,54	1,27
ENSG00000128040	SPINK2	2,54	1,37
ENSG00000147869	CER1	2,54	1,26
ENSG00000166869	CHP2	2,54	1,28
ENSG00000048162	NOP16	2,54	1,98
ENSG00000170653	ATF7	2,53	1,91
ENSG00000196376	SLC35F1	2,53	3,00
ENSG00000134962	KLB	2,53	1,27
ENSG00000006659	LGALS14	2,53	7,29
ENSG00000082515	MRPL22	2,53	1,61
ENSG00000070669	ASNS	2,53	2,14
ENSG00000113013	HSPA9	2,53	1,46
ENSG00000186335	SLC36A2	2,53	1,38

ensembl gene	gene symbol	Score(d)	Fold Change
ENSG00000176853	FAM91A1	2,53	1,35
ENSG00000187608	ISG15	2,53	2,14
ENSG00000127774	EMC6	2,52	1,38
ENSG00000139194	RBP5	2,52	1,27
ENSG00000121064	SCPEP1	2,52	2,08
ENSG00000158786	PLA2G2F	2,52	1,29
ENSG00000206549	PRSS50	2,52	1,19
ENSG00000221878	PSG7	2,52	1,31
ENSG00000107282	APBA1	2,52	1,63
ENSG00000101132	PFDN4	2,52	1,45
ENSG00000013441	CLK1	2,52	1,58
ENSG00000112208	BAG2	2,51	1,69
ENSG00000119787	ATL2	2,51	1,59
ENSG00000165688	PMPCA	2,51	1,60
ENSG00000169967	MAP3K2	2,51	1,55
ENSG00000148835	TAF5	2,51	1,44
ENSG00000167210	LOXHD1	2,51	1,20
ENSG00000163069	SGCB	2,51	3,64
ENSG00000082438	COBLL1	2,51	1,85
ENSG00000127580	WDR24	2,51	1,26
ENSG00000085721	RRN3	2,50	1,66
ENSG00000215906	LACTBL1	2,50	1,20
ENSG00000205220	PSMB10	2,50	1,56
ENSG00000183644	C11orf88	2,50	1,28
ENSG00000165732	DDX21	2,50	1,72
ENSG00000117620	SLC35A3	2,50	1,38
ENSG00000008277	ADAM22	2,50	2,28
ENSG00000242110	AMACR	2,49	1,45
ENSG00000104818	CGB2	2,49	1,33
ENSG00000138750	NUP54	2,49	1,34
ENSG00000174886	NDUFA11	2,49	1,43
ENSG00000127530	OR7C1	2,49	1,36
ENSG00000107819	SFXN3	2,49	1,48
ENSG00000135842	FAM129A	2,49	2,99
ENSG00000119820	YIPF4	2,49	1,63
ENSG00000181191	PJA1	2,49	1,86
ENSG00000069345	DNAJA2	2,49	1,58
ENSG00000109787	KLF3	2,49	1,46
ENSG00000165259	HDX	2,49	1,57
ENSG00000163071	SPATA18	2,49	1,41
ENSG00000085449	WDFY1	2,49	1,73
ENSG00000162687	KCNT2	2,49	2,80
ENSG00000182957	SPATA13	2,49	3,06
ENSG00000085741	WNT11	2,48	1,46
ENSG00000163510	CWC22	2,48	1,47
ENSG00000168259	DNAJC7	2,48	1,37
ENSG00000137574	TGS1	2,48	1,46
ENSG00000107771	CCSER2	2,48	1,47
ENSG00000124659	TBCC	2,48	1,44

ensembl gene	gene symbol	Score(d)	Fold Change
ENSG00000197930	ERO1A	2,48	1,38
ENSG00000171954	CYP4F22	2,48	1,18
ENSG00000177854	TMEM187	2,48	1,75
ENSG00000086288	NME8	2,48	1,65
ENSG00000116783	TNNI3K	2,47	1,84
ENSG00000102858	MGRN1	2,47	1,43
ENSG00000176624	MEX3C	2,47	1,34
ENSG00000181499	OR6T1	2,47	1,36
ENSG00000144635	DYNC1LI1	2,47	1,46
ENSG00000170486	KRT72	2,47	1,55
ENSG00000171262	FAM98B	2,46	1,66
ENSG00000072786	STK10	2,46	1,55
ENSG00000026025	VIM	2,46	1,70
ENSG00000141391	PRELID3A	2,46	1,34
ENSG00000165264	NDUFB6	2,46	1,64
ENSG00000181523	SGSH	2,46	1,49
ENSG00000169297	NR0B1	2,46	1,30
ENSG00000159202	UBE2Z	2,46	1,60
ENSG00000009954	BAZ1B	2,46	1,40
ENSG00000197780	TAF13	2,45	1,59
ENSG00000171492	LRRC8D	2,45	1,58
ENSG00000128805	ARHGAP22	2,45	1,87
ENSG00000103507	BCKDK	2,45	1,69
ENSG00000043591	ADRB1	2,45	1,34
ENSG00000120215	MLANA	2,45	1,21
ENSG00000213213	CCDC183	2,45	1,66
ENSG00000056558	TRAF1	2,45	1,48
ENSG00000196151	WDSUB1	2,45	1,58
ENSG00000002919	SNX11	2,45	1,46
ENSG00000067829	IDH3G	2,45	1,40
ENSG00000111046	MYF6	2,45	2,31
ENSG00000178105	DDX10	2,45	2,02
ENSG00000237515	SHISA9	2,45	1,30
ENSG00000146232	NFKBIE	2,44	2,02
ENSG00000244165	P2RY11	2,44	1,67
ENSG00000214860	EVPLL	2,44	1,54
ENSG00000149488	TMC2	2,44	1,29
ENSG00000067248	DHX29	2,44	1,38
ENSG00000139620	KANSL2	2,44	1,29
ENSG00000135972	MRPS9	2,44	1,50
ENSG00000105982	RNF32	2,44	1,71
ENSG00000181218	HIST3H2A	2,44	2,22
ENSG00000204308	RNF5	2,44	1,51
ENSG00000167112	TRUB2	2,44	1,54
ENSG00000258713	C20orf141	2,43	1,34
ENSG00000118707	TGIF2	2,43	1,60
ENSG00000144736	SHQ1	2,43	1,77
ENSG00000108588	CCDC47	2,43	1,45
ENSG00000132846	ZBED3	2,43	1,57

ensembl gene	gene symbol	Score(d)	Fold Change
ENSG00000142279	WTIP	2,43	1,23
ENSG00000146872	TLK2	2,43	1,34
ENSG00000092148	HECTD1	2,43	1,25
ENSG00000142230	SAE1	2,43	1,44
ENSG00000075188	NUP37	2,43	1,64
ENSG00000173464	RNASE11	2,43	1,96
ENSG00000165916	PSMC3	2,43	1,54
ENSG00000108443	RPS6KB1	2,43	1,49
ENSG00000108582	CPD	2,43	2,00
ENSG00000205730	ITPRIPL2	2,43	1,42
ENSG00000163946	FAM208A	2,43	1,51
ENSG00000170128	GPR25	2,43	1,28
ENSG00000069424	KCNAB2	2,43	1,41
ENSG00000146755	TRIM50	2,42	1,39
ENSG00000169594	BNC1	2,42	1,19
ENSG00000067064	IDI1	2,42	1,46
ENSG00000185883	ATP6V0C	2,42	1,26
ENSG00000236279	CLEC2L	2,42	1,44
ENSG00000283697	RP5-937E21.8	2,42	1,32
ENSG00000181215	C4orf50	2,42	1,20
ENSG00000163749	CCDC158	2,41	1,82
ENSG00000213973	ZNF99	2,41	1,29
ENSG00000175324	LSM1	2,41	1,43
ENSG00000124783	SSR1	2,41	1,34
ENSG00000188761	BCL2L15	2,41	1,62
ENSG00000197603	C5orf42	2,41	1,57
ENSG00000120725	SIL1	2,41	1,59
ENSG00000171100	MTM1	2,41	1,47
ENSG00000090013	BLVRB	2,41	1,89
ENSG00000183798	EMILIN3	2,41	1,24
ENSG00000158315	RHBDL2	2,41	1,28
ENSG00000006042	TMEM98	2,41	1,43
ENSG00000130045	NXNL2	2,41	1,42
ENSG00000178338	ZNF354B	2,40	1,35
ENSG00000205277	MUC12	2,40	1,25
ENSG00000240857	RDH14	2,40	1,33
ENSG00000186141	POLR3C	2,40	1,58
ENSG00000113318	MSH3	2,40	1,32
ENSG00000196368	NUDT11	2,40	1,63
ENSG00000212710	CTAGE1	2,40	1,37
ENSG00000180988	OR52N2	2,40	1,39
ENSG00000126247	CAPNS1	2,40	1,56
ENSG00000118620	ZNF430	2,40	2,12
ENSG00000160410	SHKBP1	2,39	1,35
ENSG00000171824	EXOSC10	2,39	1,27
ENSG00000085433	WDR47	2,39	1,70
ENSG00000100749	VRK1	2,39	1,94
ENSG00000166743	ACSM1	2,39	1,43
ENSG00000166557	TMED3	2,39	1,69

ensembl gene	gene symbol	Score(d)	Fold Change
ENSG00000155868	MED7	2,39	1,31
ENSG00000140471	LINS1	2,39	1,48
ENSG00000100083	GGA1	2,39	1,40
ENSG00000174963	ZIC4	2,39	1,24
ENSG00000166788	SAAL1	2,38	1,46
ENSG00000006062	MAP3K14	2,38	1,76
ENSG00000119185	ITGB1BP1	2,38	1,60
ENSG00000129675	ARHGEF6	2,38	2,10
ENSG00000213160	KLHL23	2,38	1,96
ENSG00000149050	ZNF214	2,38	1,27
ENSG00000175550	DRAP1	2,38	1,79
ENSG00000197410	DCHS2	2,38	1,27
ENSG00000029725	RABEP1	2,38	1,63
ENSG00000099995	SF3A1	2,38	1,44
ENSG00000154451	GBP5	2,38	2,72
ENSG00000113407	TARS	2,38	1,69
ENSG00000214842	RAD51AP2	2,38	1,14
ENSG00000183549	ACSM5	2,38	1,46
ENSG00000129219	PLD2	2,38	1,72
ENSG00000131871	VIMP	2,38	1,51
ENSG00000105085	MED26	2,38	1,67
ENSG00000174748	RPL15	2,37	1,48
ENSG00000082516	GEMIN5	2,37	1,70
ENSG00000174227	PIGG	2,37	1,72
ENSG00000164284	GRPEL2	2,37	1,66
ENSG00000086289	EPDR1	2,37	2,01
ENSG00000113580	NR3C1	2,37	1,54
ENSG00000015171	ZMYND11	2,37	1,34
ENSG00000138315	OIT3	2,37	1,30
ENSG00000145113	MUC4	2,37	1,60
ENSG00000196365	LONP1	2,37	1,67
ENSG00000133641	C12orf29	2,37	1,40
ENSG00000125841	NRSN2	2,36	1,59
ENSG00000178913	TAF7	2,36	1,48
ENSG00000123349	PFDN5	2,36	1,44
ENSG00000171503	ETFDH	2,36	1,56
ENSG00000176406	RIMS2	2,36	3,40
ENSG00000126895	AVPR2	2,36	1,20
ENSG00000111877	MCM9	2,36	1,35
ENSG00000241241	KRTAP4-16	2,36	1,54
ENSG00000138796	HADH	2,36	2,28
ENSG00000104549	SQLE	2,36	1,63
ENSG00000121905	HPCA	2,36	1,47
ENSG00000178226	PRSS36	2,36	1,53
ENSG00000186575	NF2	2,36	1,65
ENSG00000120709	FAM53C	2,36	1,43
ENSG00000100079	LGALS2	2,36	1,40
ENSG00000241128	OR14A2	2,36	1,46
ENSG00000158710	TAGLN2	2,36	1,83

ensembl gene	gene symbol	Score(d)	Fold Change
ENSG00000165449	SLC16A9	2,35	1,39
ENSG00000101200	AVP	2,35	1,29
ENSG00000062650	WAPL	2,35	1,41
ENSG00000006634	DBF4	2,35	1,82
ENSG00000149150	SLC43A1	2,35	2,03
ENSG00000186329	TMEM212	2,35	1,46
ENSG00000187581	COX8C	2,35	1,20
ENSG00000166582	CENPV	2,35	1,70
ENSG00000166411	IDH3A	2,35	1,35
ENSG00000173769	TOPAZ1	2,35	1,16
ENSG00000117682	DHDDS	2,35	1,59
ENSG00000124216	SNAI1	2,35	1,43
ENSG00000137713	PPP2R1B	2,34	1,53
ENSG00000196890	HIST3H2BB	2,34	1,68
ENSG00000109805	NCAPG	2,34	2,12
ENSG00000173335	CST9	2,34	1,21
ENSG00000159884	CCDC107	2,34	1,27
ENSG00000179262	RAD23A	2,34	1,34
ENSG00000077454	LRCH4	2,34	1,52
ENSG00000204315	FKBPL	2,34	1,25
ENSG00000156269	NAA11	2,34	1,69
ENSG00000168615	ADAM9	2,34	2,46
ENSG00000069764	PLA2G10	2,33	2,10
ENSG00000255713	OR4D2	2,33	1,55
ENSG00000078668	VDAC3	2,33	1,78
ENSG00000006652	IFRD1	2,33	1,68
ENSG00000168143	FAM83B	2,33	1,23
ENSG00000143977	SNRPG	2,33	1,52
ENSG00000227500	SCAMP4	2,33	1,48
ENSG00000164045	CDC25A	2,33	1,37
ENSG00000152463	OLAH	2,33	3,57
ENSG00000103067	ESRP2	2,33	1,52
ENSG00000108479	GALK1	2,32	1,21
ENSG00000108854	SMURF2	2,32	1,46
ENSG00000133055	MYBPH	2,32	1,19
ENSG00000179331	RAB39A	2,32	1,95
ENSG00000172031	EPHX4	2,32	1,43
ENSG00000095917	TPSD1	2,32	1,38
ENSG00000132530	XAF1	2,32	2,17
ENSG00000103174	NAGPA	2,32	1,68
ENSG00000169856	ONECUT1	2,31	1,28
ENSG00000102007	PLP2	2,31	1,40
ENSG00000160087	UBE2J2	2,31	1,51
ENSG00000177733	HNRNPA0	2,31	1,38
ENSG00000102683	SGCG	2,31	1,32
ENSG00000184305	CCSER1	-2,14	0,40
ENSG00000165457	FOLR2	-2,14	0,65
ENSG00000126773	PCNX4	-2,14	0,59
ENSG00000004975	DVL2	-2,14	0,75

ensembl gene	gene symbol	Score(d)	Fold Change
ENSG00000118096	IFT46	-2,14	0,60
ENSG00000140678	ITGAX	-2,14	0,61
ENSG00000104643	MTMR9	-2,15	0,69
ENSG00000115884	SDC1	-2,15	0,66
ENSG00000170549	IRX1	-2,15	0,77
ENSG00000196557	CACNA1H	-2,15	0,70
ENSG00000105479	CCDC114	-2,15	0,75
ENSG00000197584	KCNMB2	-2,15	0,36
ENSG00000160055	TMEM234	-2,15	0,76
ENSG00000135439	AGAP2	-2,15	0,62
ENSG00000100429	HDAC10	-2,15	0,56
ENSG00000149932	TMEM219	-2,15	0,73
ENSG00000092009	CMA1	-2,15	0,86
ENSG00000256436	TAS2R31	-2,15	0,55
ENSG00000071909	MYO3B	-2,15	0,38
ENSG00000111536	IL26	-2,15	0,63
ENSG00000196505	GDAP2	-2,15	0,71
ENSG00000116690	PRG4	-2,15	0,72
ENSG00000179902	C1orf194	-2,15	0,83
ENSG00000170989	S1PR1	-2,15	0,41
ENSG00000181751	C5orf30	-2,16	0,64
ENSG00000198894	CIPC	-2,16	0,73
ENSG00000112033	PPARD	-2,16	0,74
ENSG00000103769	RAB11A	-2,16	0,73
ENSG00000184992	BRI3BP	-2,16	0,65
ENSG00000143353	LYPLAL1	-2,16	0,62
ENSG00000140400	MAN2C1	-2,16	0,68
ENSG00000105829	BET1	-2,16	0,50
ENSG00000183091	NEB	-2,16	0,25
ENSG00000140398	NEIL1	-2,16	0,51
ENSG00000075420	FNDC3B	-2,16	0,66
ENSG00000215343	ZNF705D	-2,16	0,80
ENSG00000152767	FARP1	-2,16	0,46
ENSG00000126456	IRF3	-2,16	0,69
ENSG00000153982	GDPD1	-2,16	0,45
ENSG00000132688	NES	-2,16	0,43
ENSG00000155506	LARP1	-2,17	0,77
ENSG00000078269	SYNJ2	-2,17	0,75
ENSG00000196372	ASB13	-2,17	0,70
ENSG00000197879	MYO1C	-2,17	0,65
ENSG00000132424	PNISR	-2,17	0,77
ENSG00000135097	MSI1	-2,17	0,81
ENSG00000168772	CXXC4	-2,17	0,40
ENSG00000168447	SCNN1B	-2,17	0,42
ENSG00000105204	DYRK1B	-2,17	0,71
ENSG00000204186	ZDBF2	-2,17	0,62
ENSG00000131788	PIAS3	-2,17	0,74
ENSG00000115652	UXS1	-2,17	0,66
ENSG00000257335	MGAM	-2,17	0,48

ensembl gene	gene symbol	Score(d)	Fold Change
ENSG00000001629	ANKIB1	-2,17	0,67
ENSG00000178852	EFCAB13	-2,17	0,63
ENSG00000197428	OR51D1	-2,17	0,72
ENSG00000150967	ABCB9	-2,17	0,60
ENSG00000082482	KCNK2	-2,17	0,81
ENSG00000100292	HMOX1	-2,18	0,51
ENSG00000256294	ZNF225	-2,18	0,68
ENSG00000184384	MAML2	-2,18	0,57
ENSG00000149243	KLHL35	-2,18	0,68
ENSG00000148655	C10orf11	-2,18	0,48
ENSG00000100246	DNAL4	-2,18	0,65
ENSG00000156738	MS4A1	-2,18	0,15
ENSG00000112624	GLTSCR1L	-2,18	0,69
ENSG00000170734	POLH	-2,18	0,48
ENSG00000170323	FABP4	-2,18	0,44
ENSG00000164086	DUSP7	-2,18	0,68
ENSG00000100330	MTMR3	-2,18	0,64
ENSG00000151117	TMEM86A	-2,18	0,68
ENSG00000163832	ELP6	-2,18	0,70
ENSG00000174989	FBXW8	-2,19	0,59
ENSG00000162819	BROX	-2,19	0,76
ENSG00000159189	C1QC	-2,19	0,48
ENSG00000169683	LRRC45	-2,19	0,71
ENSG00000152207	CYSLTR2	-2,19	0,42
ENSG00000136929	HEMGN	-2,19	0,68
ENSG00000105997	HOXA3	-2,19	0,78
ENSG00000168813	ZNF507	-2,19	0,66
ENSG00000177479	ARIH2	-2,19	0,78
ENSG00000046651	OFD1	-2,19	0,72
ENSG00000165959	CLMN	-2,19	0,61
ENSG00000141905	NFIC	-2,19	0,77
ENSG00000108091	CCDC6	-2,19	0,48
ENSG00000215612	HMX1	-2,19	0,83
ENSG00000103150	MLYCD	-2,19	0,71
ENSG00000197165	SULT1A2	-2,19	0,75
ENSG00000143776	CDC42BPA	-2,19	0,70
ENSG00000135093	USP30	-2,19	0,70
ENSG00000263465	SRSF8	-2,20	0,68
ENSG00000132470	ITGB4	-2,20	0,76
ENSG00000139624	CERS5	-2,20	0,71
ENSG00000158717	RNF166	-2,20	0,64
ENSG00000171044	XKR6	-2,20	0,41
ENSG00000145414	NAF1	-2,20	0,72
ENSG00000152213	ARL11	-2,20	0,66
ENSG00000169223	LMAN2	-2,20	0,67
ENSG00000123411	IKZF4	-2,20	0,65
ENSG00000183337	BCOR	-2,20	0,54
ENSG00000136371	MTHFS	-2,20	0,69
ENSG00000116157	GPX7	-2,20	0,67

ensembl gene	gene symbol	Score(d)	Fold Change
ENSG00000064961	HMG20B	-2,20	0,71
ENSG00000171649	ZIK1	-2,20	0,57
ENSG00000166987	MBD6	-2,20	0,67
ENSG00000268089	GABRQ	-2,21	0,84
ENSG00000169905	TOR1AIP2	-2,21	0,75
ENSG00000197561	ELANE	-2,21	0,52
ENSG00000154478	GPR26	-2,21	0,84
ENSG00000166167	BTRC	-2,21	0,68
ENSG00000145979	TBC1D7	-2,21	0,62
ENSG00000101040	ZMYND8	-2,21	0,59
ENSG00000178741	COX5A	-2,21	0,68
ENSG00000251369	ZNF550	-2,21	0,64
ENSG00000178764	ZHX2	-2,21	0,72
ENSG00000186399	GOLGA8R	-2,21	0,57
ENSG00000182551	ADI1	-2,21	0,75
ENSG00000139651	ZNF740	-2,21	0,67
ENSG00000033170	FUT8	-2,21	0,29
ENSG00000169047	IRS1	-2,21	0,45
ENSG00000198832	SELM	-2,22	0,55
ENSG00000154589	LY96	-2,22	0,44
ENSG00000100023	PPIL2	-2,22	0,73
ENSG00000145779	TNFAIP8	-2,22	0,57
ENSG00000119801	YPEL5	-2,22	0,73
ENSG00000147905	ZCCHC7	-2,22	0,64
ENSG00000166435	XRRA1	-2,22	0,48
ENSG00000275074	NUDT18	-2,22	0,77
ENSG00000136895	GARNL3	-2,22	0,64
ENSG00000113441	LNPEP	-2,22	0,54
ENSG00000148180	GSN	-2,23	0,59
ENSG00000189362	NEMP2	-2,23	0,64
ENSG00000130382	MLLT1	-2,23	0,66
ENSG00000074370	ATP2A3	-2,23	0,60
ENSG00000186889	TMEM17	-2,23	0,60
ENSG00000072736	NFATC3	-2,23	0,65
ENSG00000081791	KIAA0141	-2,23	0,69
ENSG00000132716	DCAF8	-2,23	0,65
ENSG00000162980	ARL5A	-2,23	0,55
ENSG00000172780	RAB43	-2,23	0,58
ENSG00000112787	FBRSL1	-2,23	0,79
ENSG00000171840	NINJ2	-2,23	0,68
ENSG00000196576	PLXNB2	-2,23	0,57
ENSG00000168286	THAP11	-2,23	0,76
ENSG00000165282	PIGO	-2,23	0,64
ENSG00000170345	FOS	-2,24	0,46
ENSG00000156398	SFXN2	-2,24	0,69
ENSG00000145982	FARS2	-2,24	0,63
ENSG00000059804	SLC2A3	-2,24	0,49
ENSG00000157985	AGAP1	-2,24	0,50
ENSG00000151623	NR3C2	-2,24	0,61

ensembl gene	gene symbol	Score(d)	Fold Change
ENSG00000168404	MLKL	-2,24	0,47
ENSG00000115042	FAHD2A	-2,24	0,58
ENSG00000187193	MT1X	-2,24	0,40
ENSG00000163563	MNDA	-2,24	0,42
ENSG00000188124	OR2AG2	-2,24	0,60
ENSG00000140443	IGF1R	-2,24	0,55
ENSG00000178719	GRINA	-2,24	0,67
ENSG00000178917	ZNF852	-2,24	0,71
ENSG00000270024	C8orf44-SGK3	-2,24	0,49
ENSG00000166825	ANPEP	-2,24	0,59
ENSG00000249948	GBA3	-2,24	0,47
ENSG00000100228	RAB36	-2,24	0,48
ENSG00000137040	RANBP6	-2,24	0,61
ENSG00000188573	FBLL1	-2,24	0,81
ENSG00000140262	TCF12	-2,24	0,76
ENSG00000176994	SMCR8	-2,24	0,73
ENSG00000142494	SLC47A1	-2,24	0,21
ENSG00000177830	CHID1	-2,25	0,67
ENSG00000166669	ATF7IP2	-2,25	0,40
ENSG00000166503	RP11-382A20.3	-2,25	0,81
ENSG00000186487	MYT1L	-2,25	0,70
ENSG00000152455	SUV39H2	-2,25	0,58
ENSG00000182896	TMEM95	-2,25	0,82
ENSG00000117691	NENF	-2,25	0,67
ENSG00000102053	ZC3H12B	-2,25	0,48
ENSG00000168872	DDX19A	-2,25	0,69
ENSG00000197782	ZNF780A	-2,25	0,64
ENSG00000132612	VPS4A	-2,25	0,62
ENSG00000188086	PRSS45	-2,25	0,71
ENSG00000125245	GPR18	-2,25	0,75
ENSG00000110057	UNC93B1	-2,25	0,67
ENSG00000139874	SSTR1	-2,25	0,79
ENSG00000164597	COG5	-2,25	0,67
ENSG00000187118	CMC1	-2,25	0,61
ENSG00000144120	TMEM177	-2,25	0,57
ENSG00000205209	SCGB2B2	-2,25	0,71
ENSG00000113328	CCNG1	-2,25	0,74
ENSG00000043039	BARX2	-2,25	0,57
ENSG00000157216	SSBP3	-2,26	0,70
ENSG00000168310	IRF2	-2,26	0,52
ENSG00000143811	PYCR2	-2,26	0,59
ENSG00000116962	NID1	-2,26	0,77
ENSG00000035403	VCL	-2,26	0,62
ENSG00000108784	NAGLU	-2,26	0,45
ENSG00000159618	ADGRG5	-2,26	0,43
ENSG00000182183	FAM159A	-2,26	0,53
ENSG00000108306	FBXL20	-2,26	0,64
ENSG00000156575	PRG3	-2,26	0,62
ENSG00000136295	TTYH3	-2,26	0,71

ensembl gene	gene symbol	Score(d)	Fold Change
ENSG00000183808	RBM12B	-2,27	0,63
ENSG00000156463	SH3RF2	-2,27	0,60
ENSG00000213626	LBH	-2,27	0,68
ENSG00000241399	CD302	-2,27	0,57
ENSG00000158435	CNOT11	-2,27	0,81
ENSG00000175592	FOSL1	-2,27	0,79
ENSG00000174579	MSL2	-2,27	0,65
ENSG00000107829	FBXW4	-2,27	0,62
ENSG00000168243	GNG4	-2,27	0,72
ENSG00000077157	PPP1R12B	-2,27	0,58
ENSG00000173409	ARV1	-2,27	0,62
ENSG00000171604	CXXC5	-2,27	0,60
ENSG00000176182	MYPOP	-2,27	0,76
ENSG00000125637	PSD4	-2,27	0,75
ENSG00000156869	FRRS1	-2,27	0,60
ENSG00000138944	KIAA1644	-2,27	0,76
ENSG00000173210	ABLIM3	-2,27	0,78
ENSG00000169435	RASSF6	-2,27	0,35
ENSG00000169764	UGP2	-2,28	0,67
ENSG00000175536	LIPT2	-2,28	0,74
ENSG00000111348	ARHGDIB	-2,28	0,73
ENSG00000122728	TAF1L	-2,28	0,79
ENSG00000137393	RNF144B	-2,28	0,31
ENSG00000132640	BTBD3	-2,28	0,31
ENSG00000078967	UBE2D4	-2,28	0,68
ENSG00000131368	MRPS25	-2,28	0,75
ENSG00000164035	EMCN	-2,28	0,68
ENSG00000167861	HID1	-2,28	0,59
ENSG00000197774	EME2	-2,28	0,78
ENSG00000163875	MEAF6	-2,29	0,74
ENSG00000175567	UCP2	-2,29	0,51
ENSG00000258223	PRSS58	-2,29	0,83
ENSG00000171843	MLLT3	-2,29	0,58
ENSG00000164576	SAP30L	-2,29	0,66
ENSG00000007129	CEACAM21	-2,29	0,65
ENSG00000198728	LDB1	-2,29	0,65
ENSG00000154945	ANKRD40	-2,29	0,74
ENSG00000073598	FNDC8	-2,29	0,88
ENSG00000135423	GLS2	-2,29	0,75
ENSG00000127920	GNG11	-2,29	0,57
ENSG00000264364	DYNLL2	-2,29	0,66
ENSG00000102837	OLFM4	-2,29	0,35
ENSG00000165682	CLEC1B	-2,29	0,74
ENSG00000168646	AXIN2	-2,29	0,57
ENSG00000079841	RIMS1	-2,30	0,39
ENSG00000100600	LGMN	-2,30	0,41
ENSG00000179776	CDH5	-2,30	0,78
ENSG00000137210	TMEM14B	-2,30	0,70
ENSG00000110074	FOXRED1	-2,30	0,71

ensembl gene	gene symbol	Score(d)	Fold Change
ENSG00000132313	MRPL35	-2,30	0,61
ENSG00000134452	FBXO18	-2,30	0,62
ENSG00000119335	SET	-2,30	0,64
ENSG00000162148	PPP1R32	-2,30	0,81
ENSG00000157637	SLC38A10	-2,30	0,63
ENSG00000100504	PYGL	-2,31	0,48
ENSG00000162722	TRIM58	-2,31	0,78
ENSG00000177354	C10orf71	-2,31	0,69
ENSG00000163545	NUAK2	-2,31	0,52
ENSG00000147573	TRIM55	-2,31	0,42
ENSG00000166432	ZMAT1	-2,31	0,46
ENSG00000158201	ABHD3	-2,31	0,68
ENSG00000099814	CEP170B	-2,31	0,69
ENSG00000103037	SETD6	-2,31	0,68
ENSG00000175857	GAPT	-2,31	0,64
ENSG00000119899	SLC17A5	-2,31	0,66
ENSG00000112679	DUSP22	-2,31	0,60
ENSG00000100218	RSPH14	-2,31	0,66
ENSG00000182087	TMEM259	-2,31	0,64
ENSG00000115020	PIKFYVE	-2,31	0,65
ENSG00000115446	UNC50	-2,31	0,73
ENSG00000081052	COL4A4	-2,32	0,70
ENSG00000122188	LAX1	-2,32	0,42
ENSG00000073169	SELO	-2,32	0,76
ENSG00000154930	ACSS1	-2,32	0,51
ENSG00000185669	SNAI3	-2,32	0,75
ENSG00000160688	FLAD1	-2,32	0,60
ENSG00000104967	NOVA2	-2,32	0,62
ENSG00000143621	ILF2	-2,32	0,79
ENSG00000122641	INHBA	-2,32	0,78
ENSG00000143457	GOLPH3L	-2,33	0,47
ENSG00000105650	PDE4C	-2,33	0,78
ENSG00000205364	MT1M	-2,33	0,68
ENSG00000173890	GPR160	-2,33	0,53
ENSG00000149346	SLX4IP	-2,33	0,58
ENSG00000065526	SPEN	-2,33	0,67
ENSG00000103043	VAC14	-2,33	0,68
ENSG00000267561	RP5-1052I5.2	-2,33	0,58
ENSG00000099834	CDHR5	-2,33	0,80
ENSG00000130024	PHF10	-2,33	0,71
ENSG00000100985	MMP9	-2,34	0,37
ENSG00000106823	ECM2	-2,34	0,68
ENSG00000128052	KDR	-2,34	0,74
ENSG00000179832	MROH1	-2,34	0,77
ENSG00000131779	PEX11B	-2,34	0,58
ENSG00000169439	SDC2	-2,34	0,26
ENSG00000125835	SNRPB	-2,34	0,62
ENSG00000182022	CHST15	-2,34	0,59
ENSG00000066382	MPPED2	-2,35	0,33

ensembl gene	gene symbol	Score(d)	Fold Change
ENSG00000154429	CCSAP	-2,35	0,50
ENSG00000137801	THBS1	-2,35	0,36
ENSG00000240184	PCDHGC3	-2,35	0,50
ENSG00000163877	SNIP1	-2,35	0,62
ENSG00000101307	SIRPB1	-2,35	0,59
ENSG00000089063	TMEM230	-2,35	0,76
ENSG00000163138	PACRGL	-2,35	0,46
ENSG00000198833	UBE2J1	-2,35	0,81
ENSG00000179085	DPM3	-2,35	0,63
ENSG00000206102	KRTAP19-8	-2,35	0,38
ENSG00000164830	OXR1	-2,35	0,65
ENSG00000197530	MIB2	-2,35	0,76
ENSG00000164654	MIOS	-2,35	0,57
ENSG00000112232	KHDRBS2	-2,35	0,39
ENSG00000180447	GAS1	-2,35	0,67
ENSG00000204231	RXRB	-2,35	0,76
ENSG00000237172	B3GNT9	-2,36	0,60
ENSG00000177138	FAM9B	-2,36	0,77
ENSG00000013016	EHD3	-2,36	0,40
ENSG00000119508	NR4A3	-2,36	0,45
ENSG00000088179	PTPN4	-2,36	0,66
ENSG00000196126	HLA-DRB1	-2,36	0,34
ENSG00000174943	KCTD13	-2,36	0,78
ENSG00000127837	AAMP	-2,36	0,74
ENSG00000188938	FAM120AOS	-2,36	0,71
ENSG00000113595	TRIM23	-2,36	0,68
ENSG00000167523	SPATA33	-2,36	0,54
ENSG00000180658	OR2A4	-2,36	0,75
ENSG00000090581	GNPTG	-2,36	0,53
ENSG00000113194	FAF2	-2,36	0,76
ENSG00000106077	ABHD11	-2,36	0,67
ENSG00000153707	PTPRD	-2,36	0,45
ENSG00000162511	LAPTM5	-2,36	0,28
ENSG00000255823	MTRNR2L8	-2,36	0,77
ENSG00000187650	VMAC	-2,36	0,72
ENSG00000204531	POU5F1	-2,36	0,43
ENSG00000129657	SEC14L1	-2,37	0,62
ENSG00000116497	S100PBP	-2,37	0,61
ENSG00000177570	SAMD12	-2,37	0,44
ENSG00000135407	AVIL	-2,37	0,67
ENSG00000182870	GALNT9	-2,37	0,59
ENSG00000187147	RNF220	-2,37	0,75
ENSG00000114902	SPCS1	-2,37	0,78
ENSG00000150977	RILPL2	-2,37	0,54
ENSG00000087460	GNAS	-2,37	0,78
ENSG00000072657	TRHDE	-2,37	0,63
ENSG00000039600	SOX30	-2,37	0,74
ENSG00000086506	HBQ1	-2,37	0,77
ENSG00000163083	INHBB	-2,37	0,83

ensembl gene	gene symbol	Score(d)	Fold Change
ENSG00000116017	ARID3A	-2,38	0,69
ENSG00000090402	SI	-2,38	0,59
ENSG00000135686	KLHL36	-2,38	0,51
ENSG00000063322	MED29	-2,38	0,80
ENSG00000122254	HS3ST2	-2,38	0,62
ENSG00000197594	ENPP1	-2,38	0,28
ENSG00000178665	ZNF713	-2,38	0,46
ENSG00000128383	APOBEC3A	-2,38	0,53
ENSG00000243317	C7orf73	-2,38	0,70
ENSG00000002745	WNT16	-2,38	0,61
ENSG00000149357	LAMTOR1	-2,38	0,62
ENSG00000163463	KRTCAP2	-2,39	0,71
ENSG00000215883	CYB5RL	-2,39	0,73
ENSG00000078177	N4BP2	-2,39	0,63
ENSG00000154822	PLCL2	-2,39	0,50
ENSG00000160796	NBEAL2	-2,39	0,49
ENSG00000174500	GCSAM	-2,39	0,28
ENSG00000159899	NPR2	-2,39	0,65
ENSG00000213347	MXD3	-2,39	0,64
ENSG00000225973	PIGBOS1	-2,39	0,76
ENSG00000203710	CR1	-2,39	0,49
ENSG00000204815	TTC25	-2,39	0,78
ENSG00000136100	VPS36	-2,39	0,65
ENSG00000125409	TEKT3	-2,39	0,67
ENSG00000260314	MRC1	-2,39	0,70
ENSG00000040487	PQLC2	-2,39	0,68
ENSG00000130208	APOC1	-2,40	0,58
ENSG00000186132	C2orf76	-2,40	0,61
ENSG00000130312	MRPL34	-2,40	0,57
ENSG00000165209	STRBP	-2,40	0,58
ENSG00000182158	CREB3L2	-2,40	0,65
ENSG00000103313	MEFV	-2,40	0,62
ENSG00000228439	TSTD3	-2,40	0,63
ENSG00000172466	ZNF24	-2,40	0,72
ENSG00000133740	E2F5	-2,40	0,62
ENSG00000115866	DARS	-2,41	0,61
ENSG00000110719	TCIRG1	-2,41	0,68
ENSG00000185112	FAM43A	-2,41	0,54
ENSG00000141627	DYM	-2,41	0,75
ENSG00000167419	LPO	-2,41	0,63
ENSG00000178585	CTNNBIP1	-2,41	0,82
ENSG00000197070	ARRDC1	-2,41	0,69
ENSG00000162729	IGSF8	-2,41	0,67
ENSG00000171443	ZNF524	-2,42	0,80
ENSG00000165997	ARL5B	-2,42	0,64
ENSG00000112303	VNN2	-2,42	0,38
ENSG00000196290	NIF3L1	-2,42	0,62
ENSG00000161664	ASB16	-2,42	0,72
ENSG00000185278	ZBTB37	-2,42	0,67

ensembl gene	gene symbol	Score(d)	Fold Change
ENSG00000172086	KRCC1	-2,42	0,59
ENSG00000164080	RAD54L2	-2,42	0,72
ENSG00000089022	MAPKAPK5	-2,43	0,76
ENSG00000157741	UBN2	-2,43	0,65
ENSG00000164587	RPS14	-2,43	0,66
ENSG00000134744	ZCCHC11	-2,43	0,64
ENSG00000196329	GIMAP5	-2,43	0,38
ENSG00000105793	GTPBP10	-2,43	0,62
ENSG00000130748	TMEM160	-2,43	0,53
ENSG00000134326	CMPK2	-2,43	0,37
ENSG00000196569	LAMA2	-2,43	0,28
ENSG00000198108	CHSY3	-2,43	0,37
ENSG00000167635	ZNF146	-2,44	0,63
ENSG00000187474	FPR3	-2,44	0,64
ENSG00000048462	TNFRSF17	-2,44	0,44
ENSG00000213015	ZNF580	-2,44	0,82
ENSG00000182919	C11orf54	-2,44	0,64
ENSG00000205038	PKHD1L1	-2,44	0,52
ENSG00000186529	CYP4F3	-2,44	0,50
ENSG00000160917	CPSF4	-2,45	0,61
ENSG00000161980	POLR3K	-2,45	0,63
ENSG00000100578	KIAA0586	-2,45	0,64
ENSG00000112936	C7	-2,45	0,57
ENSG00000221869	CEBPD	-2,45	0,71
ENSG00000124256	ZBP1	-2,45	0,55
ENSG00000091106	NLRC4	-2,45	0,79
ENSG00000133878	DUSP26	-2,45	0,43
ENSG00000134574	DDB2	-2,45	0,61
ENSG00000154229	PRKCA	-2,45	0,30
ENSG00000187815	ZFP69	-2,45	0,65
ENSG00000119725	ZNF410	-2,45	0,43
ENSG00000173391	OLR1	-2,45	0,51
ENSG00000050820	BCAR1	-2,45	0,60
ENSG00000116525	TRIM62	-2,45	0,64
ENSG00000091490	SEL1L3	-2,45	0,56
ENSG00000148943	LIN7C	-2,46	0,64
ENSG00000169962	TAS1R3	-2,46	0,62
ENSG00000010626	LRRC23	-2,46	0,68
ENSG00000147799	ARHGAP39	-2,46	0,61
ENSG00000102221	JADE3	-2,46	0,62
ENSG00000176209	SMIM19	-2,46	0,52
ENSG00000173065	FAM222B	-2,46	0,68
ENSG00000138175	ARL3	-2,46	0,64
ENSG00000119912	IDE	-2,46	0,70
ENSG00000176444	CLK2	-2,46	0,60
ENSG00000136731	UGGT1	-2,46	0,76
ENSG00000106868	SUSD1	-2,47	0,42
ENSG00000129151	BBOX1	-2,47	0,58
ENSG00000163795	ZNF513	-2,47	0,77

ensembl gene	gene symbol	Score(d)	Fold Change
ENSG00000187742	SECISBP2	-2,47	0,75
ENSG00000185900	POMK	-2,47	0,54
ENSG00000151612	ZNF827	-2,47	0,61
ENSG00000126261	UBA2	-2,47	0,68
ENSG00000051620	HEBP2	-2,47	0,61
ENSG00000160058	BSDC1	-2,47	0,66
ENSG00000107147	KCNT1	-2,47	0,67
ENSG00000225698	IGHV3-72	-2,47	0,42
ENSG00000149212	SESN3	-2,47	0,66
ENSG00000116918	TSNAX	-2,47	0,36
ENSG00000151893	CACUL1	-2,47	0,67
ENSG00000104154	SLC30A4	-2,47	0,61
ENSG00000180340	FZD2	-2,47	0,78
ENSG00000104765	BNIP3L	-2,48	0,71
ENSG00000007541	PIGQ	-2,48	0,61
ENSG00000167346	MMP26	-2,48	0,77
ENSG00000152443	ZNF776	-2,48	0,60
ENSG00000106638	TBL2	-2,48	0,67
ENSG00000158195	WASF2	-2,48	0,68
ENSG00000188021	UBQLN2	-2,48	0,65
ENSG00000215455	KRTAP10-1	-2,48	0,63
ENSG00000121053	EPX	-2,48	0,38
ENSG00000102763	VWA8	-2,48	0,60
ENSG00000188033	ZNF490	-2,48	0,71
ENSG00000162739	SLAMF6	-2,48	0,20
ENSG00000136156	ITM2B	-2,48	0,74
ENSG00000265190	ANXA8	-2,49	0,72
ENSG00000118260	CREB1	-2,49	0,70
ENSG00000198881	ASB12	-2,49	0,77
ENSG00000168036	CTNNB1	-2,49	0,57
ENSG00000179151	EDC3	-2,49	0,55
ENSG00000111652	COPS7A	-2,49	0,65
ENSG00000100299	ARSA	-2,49	0,59
ENSG00000145012	LPP	-2,49	0,57
ENSG00000169519	METTL15	-2,49	0,50
ENSG00000103363	TCEB2	-2,49	0,65
ENSG00000168763	CNNM3	-2,49	0,73
ENSG00000188554	NBR1	-2,49	0,62
ENSG00000230510	PPP5D1	-2,49	0,72
ENSG00000046647	GEMIN8	-2,50	0,65
ENSG00000123096	SSPN	-2,50	0,51
ENSG00000133812	SBF2	-2,50	0,35
ENSG00000147654	EBAG9	-2,50	0,74
ENSG00000159199	ATP5G1	-2,50	0,41
ENSG00000105323	HNRNPUL1	-2,50	0,75
ENSG00000133818	RRAS2	-2,50	0,20
ENSG00000141458	NPC1	-2,50	0,54
ENSG00000196812	ZSCAN16	-2,51	0,65
ENSG00000053770	AP5M1	-2,51	0,71

ensembl gene	gene symbol	Score(d)	Fold Change
ENSG00000051523	CYBA	-2,51	0,64
ENSG00000167964	RAB26	-2,51	0,69
ENSG00000152291	TGOLN2	-2,51	0,73
ENSG00000168530	MYL1	-2,51	0,57
ENSG00000146083	RNF44	-2,51	0,66
ENSG00000164142	FAM160A1	-2,51	0,27
ENSG00000162735	PEX19	-2,51	0,69
ENSG00000171236	LRG1	-2,51	0,61
ENSG00000113448	PDE4D	-2,51	0,22
ENSG00000102760	RGCC	-2,51	0,25
ENSG00000108950	FAM20A	-2,51	0,71
ENSG00000170142	UBE2E1	-2,51	0,58
ENSG00000155275	TRMT44	-2,52	0,71
ENSG00000143252	SDHC	-2,52	0,62
ENSG00000177380	PPFIA3	-2,52	0,65
ENSG00000109220	CHIC2	-2,52	0,61
ENSG00000138061	CYP1B1	-2,52	0,64
ENSG00000169116	PARM1	-2,52	0,48
ENSG00000121316	PLBD1	-2,52	0,37
ENSG00000109083	IFT20	-2,52	0,65
ENSG00000147454	SLC25A37	-2,52	0,64
ENSG00000109133	TMEM33	-2,52	0,71
ENSG00000132906	CASP9	-2,52	0,77
ENSG00000120279	MYCT1	-2,52	0,71
ENSG00000077044	DGKD	-2,52	0,53
ENSG00000282218	RP1-179P9.3	-2,52	0,51
ENSG00000169877	AHSP	-2,53	0,42
ENSG00000168556	ING2	-2,53	0,73
ENSG00000188167	TMPPE	-2,53	0,59
ENSG00000171388	APLN	-2,53	0,74
ENSG00000109436	TBC1D9	-2,53	0,58
ENSG00000079150	FKBP7	-2,53	0,51
ENSG00000177469	PTRF	-2,53	0,77
ENSG00000171310	CHST11	-2,53	0,41
ENSG00000176422	SPRYD4	-2,53	0,56
ENSG00000162736	NCSTN	-2,53	0,69
ENSG00000152464	RPP38	-2,53	0,68
ENSG00000173372	C1QA	-2,54	0,53
ENSG00000084693	AGBL5	-2,54	0,64
ENSG00000085224	ATRX	-2,54	0,63
ENSG00000136059	VILL	-2,54	0,76
ENSG00000166352	C11orf74	-2,54	0,51
ENSG00000135070	ISCA1	-2,54	0,76
ENSG00000189056	RELN	-2,54	0,28
ENSG00000138443	ABI2	-2,54	0,57
ENSG00000144677	CTDSPL	-2,54	0,64
ENSG00000213865	C8orf44	-2,54	0,56
ENSG00000170634	ACYP2	-2,54	0,75
ENSG00000213859	KCTD11	-2,54	0,68

ensembl gene	gene symbol	Score(d)	Fold Change
ENSG00000090975	PITPNM2	-2,54	0,70
ENSG00000133114	GPALPP1	-2,55	0,68
ENSG00000163884	KLF15	-2,55	0,65
ENSG00000178562	CD28	-2,55	0,22
ENSG00000137504	CREBZF	-2,55	0,69
ENSG00000125734	GPR108	-2,55	0,63
ENSG00000110435	PDHX	-2,55	0,64
ENSG00000175137	SH3BP5L	-2,56	0,57
ENSG00000118520	ARG1	-2,56	0,56
ENSG00000100234	TIMP3	-2,56	0,71
ENSG00000087253	LPCAT2	-2,56	0,37
ENSG00000120913	PDLIM2	-2,56	0,69
ENSG00000137726	FXYD6	-2,56	0,60
ENSG00000138593	SECISBP2L	-2,56	0,56
ENSG00000111653	ING4	-2,56	0,59
ENSG00000126351	THRA	-2,56	0,60
ENSG00000075914	EXOSC7	-2,56	0,69
ENSG00000094916	CBX5	-2,56	0,56
ENSG00000237541	HLA-DQA2	-2,56	0,75
ENSG00000198018	ENTPD7	-2,56	0,75
ENSG00000135605	TEC	-2,56	0,55
ENSG00000155659	VSIG4	-2,56	0,63
ENSG00000137101	CD72	-2,56	0,66
ENSG00000166471	TMEM41B	-2,56	0,68
ENSG00000043143	JADE2	-2,56	0,62
ENSG00000173757	STAT5B	-2,56	0,46
ENSG00000166927	MS4A7	-2,56	0,37
ENSG00000213619	NDUFS3	-2,57	0,66
ENSG00000103222	ABCC1	-2,57	0,43
ENSG00000011405	PIK3C2A	-2,57	0,63
ENSG00000105738	SIPA1L3	-2,57	0,59
ENSG00000196865	NHLRC2	-2,57	0,59
ENSG00000167363	FN3K	-2,57	0,29
ENSG00000109099	PMP22	-2,57	0,60
ENSG00000149634	SPATA25	-2,57	0,66
ENSG00000169871	TRIM56	-2,57	0,52
ENSG00000155957	TMBIM4	-2,57	0,70
ENSG00000149262	INTS4	-2,57	0,59
ENSG00000087095	NLK	-2,57	0,47
ENSG00000166233	ARIH1	-2,57	0,65
ENSG00000233493	TMEM238	-2,57	0,56
ENSG00000101439	CST3	-2,57	0,35
ENSG00000123384	LRP1	-2,57	0,42
ENSG00000178104	PDE4DIP	-2,58	0,51
ENSG00000184716	SERINC4	-2,58	0,59
ENSG00000215454	KRTAP10-4	-2,58	0,71
ENSG00000105205	CLC	-2,58	0,27
ENSG00000172375	C2CD2L	-2,58	0,64
ENSG00000185305	ARL15	-2,58	0,58

ensembl gene	gene symbol	Score(d)	Fold Change
ENSG00000107719	PALD1	-2,58	0,52
ENSG00000120594	PLXDC2	-2,58	0,47
ENSG00000168229	PTGDR	-2,58	0,53
ENSG00000168300	PCMTD1	-2,58	0,61
ENSG00000135525	MAP7	-2,59	0,41
ENSG00000125107	CNOT1	-2,59	0,54
ENSG00000112237	CCNC	-2,59	0,62
ENSG00000167642	SPINT2	-2,59	0,28
ENSG00000257365	FNTB	-2,59	0,56
ENSG00000163633	C4orf36	-2,59	0,69
ENSG00000125571	IL37	-2,59	0,68
ENSG00000166211	SPIC	-2,59	0,32
ENSG00000139197	PEX5	-2,59	0,66
ENSG00000073417	PDE8A	-2,59	0,73
ENSG00000203943	SAMD13	-2,60	0,54
ENSG00000112378	PERP	-2,60	0,56
ENSG00000144455	SUMF1	-2,60	0,60
ENSG00000104324	CPQ	-2,60	0,36
ENSG00000003400	CASP10	-2,60	0,64
ENSG00000133048	CHI3L1	-2,60	0,47
ENSG00000197943	PLCG2	-2,60	0,59
ENSG00000121741	ZMYM2	-2,60	0,58
ENSG00000119535	CSF3R	-2,60	0,41
ENSG00000072756	TRNT1	-2,60	0,64
ENSG00000167644	C19orf33	-2,60	0,64
ENSG00000088812	ATRN	-2,61	0,63
ENSG00000100105	PATZ1	-2,61	0,67
ENSG00000103064	SLC7A6	-2,61	0,54
ENSG00000165632	TAF3	-2,61	0,71
ENSG00000174695	TMEM167A	-2,61	0,71
ENSG00000112658	SRF	-2,61	0,67
ENSG00000120088	CRHR1	-2,61	0,57
ENSG00000178409	BEND3	-2,61	0,66
ENSG00000093009	CDC45	-2,61	0,62
ENSG00000135473	PAN2	-2,62	0,51
ENSG00000204713	TRIM27	-2,62	0,70
ENSG00000089775	ZBTB25	-2,62	0,72
ENSG00000125337	KIF25	-2,62	0,78
ENSG00000163528	CHCHD4	-2,62	0,74
ENSG00000197629	MPEG1	-2,62	0,37
ENSG00000179941	BBS10	-2,62	0,63
ENSG00000123095	BHLHE41	-2,62	0,25
ENSG00000009413	REV3L	-2,62	0,63
ENSG00000164048	ZNF589	-2,62	0,61
ENSG00000172264	MACROD2	-2,62	0,58
ENSG00000213416	KRTAP4-12	-2,62	0,72
ENSG00000070269	TMEM260	-2,62	0,60
ENSG00000166428	PLD4	-2,62	0,28
ENSG00000184867	ARMCX2	-2,62	0,49

ensembl gene	gene symbol	Score(d)	Fold Change
ENSG00000177238	TRIM72	-2,62	0,74
ENSG00000117335	CD46	-2,62	0,58
ENSG00000137502	RAB30	-2,62	0,57
ENSG00000039319	ZFYVE16	-2,62	0,53
ENSG00000280165	PCDH20	-2,63	0,50
ENSG00000118496	FBXO30	-2,63	0,64
ENSG00000114503	NCBP2	-2,63	0,70
ENSG00000160679	CHTOP	-2,63	0,59
ENSG00000052126	PLEKHA5	-2,63	0,38
ENSG00000131067	GGT7	-2,63	0,59
ENSG00000143183	TMCO1	-2,63	0,68
ENSG00000175390	EIF3F	-2,63	0,78
ENSG00000126759	CFP	-2,63	0,74
ENSG00000013392	RWDD2A	-2,63	0,67
ENSG00000250254	PTTG2	-2,64	0,66
ENSG00000133275	CSNK1G2	-2,64	0,52
ENSG00000170456	DENND5B	-2,64	0,63
ENSG00000156928	MALSU1	-2,64	0,61
ENSG00000155465	SLC7A7	-2,64	0,49
ENSG00000143297	FCRL5	-2,64	0,32
ENSG00000076928	ARHGEF1	-2,64	0,61
ENSG00000165238	WNK2	-2,64	0,53
ENSG00000104450	SPAG1	-2,64	0,57
ENSG00000116194	ANGPTL1	-2,65	0,59
ENSG00000152240	HAUS1	-2,65	0,53
ENSG00000187486	KCNJ11	-2,65	0,72
ENSG00000148400	NOTCH1	-2,65	0,69
ENSG00000167548	KMT2D	-2,65	0,60
ENSG00000185608	MRPL40	-2,65	0,57
ENSG00000197536	C5orf56	-2,65	0,61
ENSG00000106080	FKBP14	-2,65	0,62
ENSG00000047597	XK	-2,65	0,63
ENSG00000022267	FHL1	-2,65	0,17
ENSG00000182489	XKRX	-2,65	0,32
ENSG00000275385	CCL18	-2,66	0,65
ENSG00000150681	RGS18	-2,66	0,51
ENSG00000180660	MAB21L1	-2,66	0,50
ENSG00000139629	GALNT6	-2,66	0,61
ENSG00000130368	MAS1	-2,66	0,71
ENSG00000186166	CCDC84	-2,66	0,58
ENSG00000157350	ST3GAL2	-2,66	0,58
ENSG00000168026	TTC21A	-2,66	0,61
ENSG00000118523	CTGF	-2,66	0,33
ENSG00000196715	VKORC1L1	-2,67	0,61
ENSG00000143614	GATAD2B	-2,67	0,73
ENSG00000148730	EIF4EBP2	-2,67	0,69
ENSG00000158711	ELK4	-2,67	0,76
ENSG00000204560	DHX16	-2,67	0,73
ENSG00000137509	PRCP	-2,67	0,60

ensembl gene	gene symbol	Score(d)	Fold Change
ENSG00000156467	UQCRB	-2,67	0,70
ENSG00000144401	METTL21A	-2,67	0,54
ENSG00000111666	CHPT1	-2,67	0,45
ENSG00000185245	GP1BA	-2,68	0,70
ENSG00000175727	MLXIP	-2,68	0,65
ENSG00000172139	SLC9C1	-2,68	0,57
ENSG00000153048	CARHSP1	-2,68	0,67
ENSG00000198856	OSTC	-2,68	0,71
ENSG00000240764	PCDHGC5	-2,68	0,54
ENSG00000165494	PCF11	-2,68	0,64
ENSG00000178922	HYI	-2,68	0,59
ENSG00000088053	GP6	-2,68	0,73
ENSG00000184640	SEPTIN9	-2,68	0,56
ENSG00000197646	PDCD1LG2	-2,68	0,44
ENSG00000186591	UBE2H	-2,68	0,68
ENSG00000158042	MRPL17	-2,68	0,53
ENSG00000188211	NCR3LG1	-2,68	0,47
ENSG00000164548	TRA2A	-2,68	0,75
ENSG00000187239	FNBP1	-2,68	0,17
ENSG00000108773	KAT2A	-2,68	0,54
ENSG00000171606	ZNF274	-2,68	0,65
ENSG00000086598	TMED2	-2,68	0,77
ENSG00000177076	ACER2	-2,69	0,53
ENSG00000239264	TXNDC5	-2,69	0,44
ENSG00000144445	KANSL1L	-2,69	0,44
ENSG00000107341	UBE2R2	-2,69	0,66
ENSG00000040633	PHF23	-2,69	0,66
ENSG00000174238	PITPNA	-2,69	0,63
ENSG00000101938	CHRDL1	-2,69	0,61
ENSG00000038427	VCAN	-2,69	0,57
ENSG00000144840	RABL3	-2,69	0,61
ENSG00000072042	RDH11	-2,69	0,65
ENSG00000101624	CEP76	-2,69	0,62
ENSG00000165490	DDIAS	-2,70	0,41
ENSG00000144792	ZNF660	-2,70	0,32
ENSG00000135678	CPM	-2,70	0,34
ENSG00000169031	COL4A3	-2,70	0,47
ENSG00000102780	DGKH	-2,70	0,65
ENSG00000175899	A2M	-2,70	0,41
ENSG00000115828	QPCT	-2,70	0,13
ENSG00000179909	ZNF154	-2,70	0,50
ENSG00000165185	KIAA1958	-2,70	0,65
ENSG00000103381	CPPED1	-2,70	0,35
ENSG00000175482	POLD4	-2,70	0,56
ENSG00000118922	KLF12	-2,70	0,45
ENSG00000196220	SRGAP3	-2,70	0,62
ENSG00000135974	C2orf49	-2,70	0,66
ENSG00000185697	MYBL1	-2,70	0,28
ENSG00000170615	SLC26A5	-2,70	0,19

ensembl gene	gene symbol	Score(d)	Fold Change
ENSG00000033627	ATP6V0A1	-2,71	0,44
ENSG00000068784	SRBD1	-2,71	0,58
ENSG00000258644	SYNJ2BP-COX16	-2,71	0,51
ENSG00000134121	CHL1	-2,71	0,61
ENSG00000090659	CD209	-2,71	0,55
ENSG00000145087	STXBP5L	-2,71	0,27
ENSG00000144959	NCEH1	-2,71	0,63
ENSG00000137074	APTX	-2,71	0,69
ENSG00000138777	PPA2	-2,71	0,56
ENSG00000136868	SLC31A1	-2,71	0,65
ENSG00000196092	PAX5	-2,71	0,51
ENSG00000134717	BTF3L4	-2,72	0,63
ENSG00000150907	FOXO1	-2,72	0,50
ENSG00000188916	FAM196A	-2,72	0,83
ENSG00000067141	NEO1	-2,72	0,56
ENSG00000140416	TPM1	-2,72	0,51
ENSG00000134007	ADAM20	-2,72	0,65
ENSG00000113300	CNOT6	-2,72	0,69
ENSG00000011600	TYROBP	-2,72	0,54
ENSG00000108846	ABCC3	-2,72	0,67
ENSG00000197233	OR1J2	-2,73	0,59
ENSG00000099797	TECR	-2,73	0,64
ENSG00000197385	ZNF860	-2,73	0,38
ENSG00000103365	GGA2	-2,73	0,56
ENSG00000181481	RNF135	-2,73	0,64
ENSG00000127526	SLC35E1	-2,73	0,68
ENSG00000111254	AKAP3	-2,73	0,65
ENSG00000172380	GNG12	-2,73	0,60
ENSG00000108559	NUP88	-2,73	0,63
ENSG00000185920	PTCH1	-2,74	0,36
ENSG00000235750	KIAA0040	-2,74	0,60
ENSG00000138119	MYOF	-2,74	0,40
ENSG00000185442	FAM174B	-2,74	0,50
ENSG00000100711	ZFYVE21	-2,74	0,59
ENSG00000095059	DHPS	-2,74	0,58
ENSG00000111537	IFNG	-2,74	0,50
ENSG00000111642	CHD4	-2,74	0,77
ENSG00000132199	ENOSF1	-2,74	0,55
ENSG00000109743	BST1	-2,74	0,57
ENSG00000112394	SLC16A10	-2,74	0,59
ENSG00000158869	FCER1G	-2,74	0,38
ENSG00000182255	KCNA4	-2,74	0,59
ENSG00000106686	SPATA6L	-2,75	0,56
ENSG00000149418	ST14	-2,75	0,31
ENSG00000169908	TM4SF1	-2,75	0,50
ENSG00000049618	ARID1B	-2,75	0,66
ENSG00000073849	ST6GAL1	-2,75	0,53
ENSG00000143258	USP21	-2,75	0,65
ENSG00000170545	SMAGP	-2,75	0,50

ensembl gene	gene symbol	Score(d)	Fold Change
ENSG00000149292	TTC12	-2,75	0,61
ENSG00000138606	SHF	-2,75	0,58
ENSG00000185015	CA13	-2,75	0,49
ENSG00000198520	C1orf228	-2,75	0,55
ENSG00000163626	COX18	-2,76	0,62
ENSG00000143442	POGZ	-2,76	0,66
ENSG00000117394	SLC2A1	-2,76	0,57
ENSG00000242616	GNG10	-2,76	0,48
ENSG00000100632	ERH	-2,76	0,53
ENSG00000185101	ANO9	-2,76	0,58
ENSG00000121897	LIAS	-2,76	0,63
ENSG00000004468	CD38	-2,77	0,68
ENSG00000167468	GPX4	-2,77	0,64
ENSG00000142599	RERE	-2,77	0,49
ENSG00000141759	TXNL4A	-2,77	0,63
ENSG00000215251	FASTKD5	-2,77	0,62
ENSG00000205593	DENND6B	-2,77	0,43
ENSG00000136709	WDR33	-2,78	0,64
ENSG00000196998	WDR45	-2,78	0,69
ENSG00000116285	ERRFI1	-2,78	0,44
ENSG00000139971	C14orf37	-2,78	0,58
ENSG00000174175	SELP	-2,78	0,49
ENSG00000187210	GCNT1	-2,78	0,55
ENSG00000139133	ALG10	-2,78	0,59
ENSG00000134375	TIMM17A	-2,78	0,54
ENSG00000130787	HIP1R	-2,79	0,69
ENSG00000115275	MOGS	-2,79	0,66
ENSG00000180979	LRRC57	-2,79	0,64
ENSG00000055118	KCNH2	-2,79	0,48
ENSG00000181817	LSM10	-2,79	0,62
ENSG00000169403	PTAFR	-2,79	0,64
ENSG00000127720	METTL25	-2,79	0,59
ENSG00000105662	CRTC1	-2,79	0,65
ENSG00000138722	MMRN1	-2,80	0,41
ENSG00000104093	DMXL2	-2,80	0,46
ENSG00000095397	WHRN	-2,80	0,40
ENSG00000163171	CDC42EP3	-2,80	0,41
ENSG00000170271	FAXDC2	-2,80	0,51
ENSG00000164040	PGRMC2	-2,80	0,67
ENSG00000166833	NAV2	-2,80	0,43
ENSG00000135338	LCA5	-2,81	0,39
ENSG00000162777	DENND2D	-2,81	0,59
ENSG00000154144	TBRG1	-2,81	0,54
ENSG00000158604	TMED4	-2,81	0,55
ENSG00000105698	USF2	-2,81	0,71
ENSG00000143479	DYRK3	-2,82	0,59
ENSG00000128563	PRKRIP1	-2,82	0,68
ENSG00000165338	HECTD2	-2,82	0,44
ENSG00000178057	NDUFAF3	-2,82	0,61

ensembl gene	gene symbol	Score(d)	Fold Change
ENSG00000113811	SELK	-2,82	0,61
ENSG00000172590	MRPL52	-2,83	0,65
ENSG00000117533	VAMP4	-2,83	0,57
ENSG00000127364	TAS2R4	-2,83	0,68
ENSG00000137944	KYAT3	-2,83	0,63
ENSG00000172927	MYEOV	-2,83	0,30
ENSG00000197136	PCNX3	-2,83	0,73
ENSG00000223865	HLA-DPB1	-2,83	0,50
ENSG00000170962	PDGFD	-2,83	0,53
ENSG00000140396	NCOA2	-2,83	0,57
ENSG00000119929	CUTC	-2,83	0,78
ENSG00000203737	GPR52	-2,83	0,48
ENSG00000267508	ZNF285	-2,83	0,68
ENSG00000111554	MDM1	-2,84	0,48
ENSG00000185477	GPRIN3	-2,84	0,26
ENSG00000124491	F13A1	-2,84	0,33
ENSG00000136161	RCBTB2	-2,84	0,49
ENSG00000123352	SPATS2	-2,84	0,67
ENSG00000149636	DSN1	-2,84	0,65
ENSG00000124733	MEA1	-2,84	0,58
ENSG00000160345	C9orf116	-2,84	0,44
ENSG00000129625	REEP5	-2,84	0,76
ENSG00000143013	LMO4	-2,85	0,63
ENSG00000164920	OSR2	-2,85	0,60
ENSG00000182185	RAD51B	-2,85	0,50
ENSG00000104691	UBXN8	-2,86	0,49
ENSG00000108312	UBTF	-2,86	0,58
ENSG00000187676	B3GLCT	-2,86	0,47
ENSG00000122786	CALD1	-2,86	0,51
ENSG00000129472	RAB2B	-2,87	0,65
ENSG00000187266	EPOR	-2,87	0,60
ENSG00000005381	MPO	-2,87	0,22
ENSG00000128881	TTBK2	-2,87	0,69
ENSG00000130749	ZC3H4	-2,87	0,52
ENSG00000133704	IPO8	-2,87	0,47
ENSG00000135048	TMEM2	-2,87	0,29
ENSG00000135144	DTX1	-2,88	0,38
ENSG00000041353	RAB27B	-2,88	0,64
ENSG00000186951	PPARA	-2,88	0,64
ENSG00000169814	BTD	-2,88	0,64
ENSG00000153250	RBMS1	-2,88	0,52
ENSG00000116584	ARHGEF2	-2,88	0,39
ENSG00000161921	CXCL16	-2,88	0,38
ENSG00000114544	SLC41A3	-2,88	0,67
ENSG00000128567	PODXL	-2,88	0,73
ENSG00000160551	TAOK1	-2,88	0,66
ENSG00000186812	ZNF397	-2,88	0,50
ENSG00000276234	TADA2A	-2,88	0,51
ENSG00000141956	PRDM15	-2,88	0,31

ensembl gene	gene symbol	Score(d)	Fold Change
ENSG00000131094	C1QL1	-2,88	0,57
ENSG00000124181	PLCG1	-2,89	0,73
ENSG00000130818	ZNF426	-2,89	0,47
ENSG00000078140	UBE2K	-2,89	0,65
ENSG00000197249	SERPINA1	-2,89	0,47
ENSG00000177628	GBA	-2,89	0,55
ENSG00000250719	RP11-322N21.2	-2,89	0,65
ENSG00000124772	CPNE5	-2,89	0,43
ENSG00000179397	C1orf101	-2,89	0,62
ENSG00000144021	CIAO1	-2,89	0,71
ENSG00000165215	CLDN3	-2,89	0,73
ENSG00000100321	SYNGR1	-2,90	0,41
ENSG00000108691	CCL2	-2,90	0,19
ENSG00000269028	MTRNR2L12	-2,90	0,38
ENSG00000021776	AQR	-2,90	0,65
ENSG00000143458	GABPB2	-2,90	0,50
ENSG00000183778	B3GALT5	-2,90	0,61
ENSG00000181555	SETD2	-2,90	0,62
ENSG00000137642	SORL1	-2,90	0,31
ENSG00000114487	MORC1	-2,91	0,36
ENSG00000060237	WNK1	-2,91	0,51
ENSG00000168038	ULK4	-2,91	0,51
ENSG00000163539	CLASP2	-2,91	0,62
ENSG00000126561	STAT5A	-2,91	0,39
ENSG00000153815	CMIP	-2,91	0,49
ENSG00000089006	SNX5	-2,91	0,69
ENSG00000100445	SDR39U1	-2,91	0,70
ENSG00000185219	ZNF445	-2,91	0,62
ENSG00000224877	C17orf89	-2,92	0,69
ENSG00000118113	MMP8	-2,92	0,14
ENSG00000129245	FXR2	-2,92	0,49
ENSG00000179979	CRIPAK	-2,92	0,60
ENSG00000006740	ARHGAP44	-2,92	0,29
ENSG00000118518	RNF146	-2,92	0,66
ENSG00000153339	TRAPPC8	-2,92	0,44
ENSG00000122783	C7orf49	-2,92	0,68
ENSG00000088970	KIZ	-2,92	0,62
ENSG00000147144	CCDC120	-2,92	0,51
ENSG00000096006	CRISP3	-2,92	0,25
ENSG00000154639	CXADR	-2,92	0,36
ENSG00000182504	CEP97	-2,93	0,45
ENSG00000116539	ASH1L	-2,93	0,51
ENSG00000171757	LRRC34	-2,93	0,45
ENSG00000106066	CPVL	-2,93	0,21
ENSG00000118690	ARMC2	-2,93	0,25
ENSG00000175470	PPP2R2D	-2,93	0,67
ENSG00000139974	SLC38A6	-2,93	0,44
ENSG00000174206	C12orf66	-2,93	0,71
ENSG00000196562	SULF2	-2,94	0,27

ensembl gene	gene symbol	Score(d)	Fold Change
ENSG00000129911	KLF16	-2,94	0,59
ENSG00000189241	TSPYL1	-2,95	0,71
ENSG00000196405	EVL	-2,95	0,51
ENSG00000160991	ORAI2	-2,95	0,66
ENSG00000147316	MCPH1	-2,95	0,66
ENSG00000177732	SOX12	-2,95	0,74
ENSG00000104774	MAN2B1	-2,95	0,58
ENSG00000204060	FOXO6	-2,95	0,68
ENSG00000242114	MTFP1	-2,95	0,55
ENSG00000151746	BICD1	-2,95	0,53
ENSG00000101384	JAG1	-2,96	0,42
ENSG00000111679	PTPN6	-2,96	0,47
ENSG00000146021	KLHL3	-2,96	0,53
ENSG00000184905	TCEAL2	-2,96	0,69
ENSG00000125952	MAX	-2,96	0,68
ENSG00000163932	PRKCD	-2,96	0,56
ENSG00000147408	CSGALNACT1	-2,96	0,37
ENSG00000138670	RASGEF1B	-2,96	0,71
ENSG00000148290	SURF1	-2,97	0,63
ENSG00000145781	COMMD10	-2,97	0,65
ENSG00000167165	UGT1A6	-2,97	0,62
ENSG00000102710	SUPT20H	-2,97	0,57
ENSG00000090316	MAEA	-2,97	0,73
ENSG00000170525	PFKFB3	-2,97	0,36
ENSG00000049323	LTBP1	-2,97	0,36
ENSG00000173120	KDM2A	-2,97	0,59
ENSG00000118579	MED28	-2,98	0,62
ENSG00000157554	ERG	-2,98	0,72
ENSG00000060749	QSER1	-2,98	0,71
ENSG00000145335	SNCA	-2,98	0,51
ENSG00000104880	ARHGEF18	-2,98	0,36
ENSG00000074201	CLNS1A	-2,99	0,46
ENSG00000079215	SLC1A3	-2,99	0,63
ENSG00000116005	PCYOX1	-2,99	0,46
ENSG00000124557	BTN1A1	-2,99	0,38
ENSG00000168283	BMI1	-2,99	0,51
ENSG00000206530	CFAP44	-2,99	0,19
ENSG00000178568	ERBB4	-2,99	0,26
ENSG00000132953	XPO4	-2,99	0,60
ENSG00000119685	TTLL5	-3,00	0,61
ENSG00000163645	ERICH6	-3,00	0,73
ENSG00000243927	MRPS6	-3,00	0,60
ENSG00000164002	EXO5	-3,00	0,57
ENSG00000169499	PLEKHA2	-3,00	0,54
ENSG00000173369	C1QB	-3,00	0,34
ENSG00000163348	PYGO2	-3,00	0,28
ENSG00000152904	GGPS1	-3,00	0,61
ENSG00000148737	TCF7L2	-3,01	0,43
ENSG00000158161	EYA3	-3,01	0,55

ensembl gene	gene symbol	Score(d)	Fold Change
ENSG00000185591	SP1	-3,01	0,56
ENSG00000136193	SCRN1	-3,01	0,48
ENSG00000142507	PSMB6	-3,01	0,50
ENSG00000241233	KRTAP5-8	-3,01	0,69
ENSG00000198270	TMEM116	-3,02	0,56
ENSG00000160856	FCRL3	-3,02	0,36
ENSG00000164603	BMT2	-3,02	0,53
ENSG00000127507	ADGRE2	-3,02	0,52
ENSG00000106609	TMEM248	-3,03	0,74
ENSG00000141556	TBCD	-3,03	0,56
ENSG00000116574	RHOU	-3,03	0,63
ENSG00000174851	YIF1A	-3,03	0,51
ENSG00000137845	ADAM10	-3,04	0,55
ENSG00000119042	SATB2	-3,04	0,52
ENSG00000182578	CSF1R	-3,04	0,52
ENSG00000114446	IFT57	-3,04	0,60
ENSG00000102471	NDFIP2	-3,04	0,34
ENSG00000175471	MCTP1	-3,04	0,54
ENSG00000120833	SOCS2	-3,04	0,57
ENSG00000152582	SPEF2	-3,04	0,55
ENSG00000156642	NPTN	-3,05	0,53
ENSG00000144560	VGLL4	-3,05	0,51
ENSG00000109208	SMR3A	-3,05	0,65
ENSG00000141441	GAREM1	-3,05	0,22
ENSG00000173209	AHSA2	-3,05	0,67
ENSG00000198105	ZNF248	-3,05	0,51
ENSG00000186088	GSAP	-3,06	0,64
ENSG00000124767	GLO1	-3,06	0,68
ENSG00000100307	CBX7	-3,06	0,39
ENSG00000104341	LAPTM4B	-3,06	0,16
ENSG00000000419	DPM1	-3,07	0,53
ENSG00000143603	KCNN3	-3,07	0,43
ENSG00000271605	MILR1	-3,07	0,29
ENSG00000005020	SKAP2	-3,07	0,34
ENSG00000185565	LSAMP	-3,07	0,18
ENSG00000127415	IDUA	-3,07	0,65
ENSG00000181027	FKRP	-3,07	0,62
ENSG00000168916	ZNF608	-3,07	0,30
ENSG00000106344	RBM28	-3,08	0,51
ENSG00000136643	RPS6KC1	-3,09	0,45
ENSG00000178252	WDR6	-3,09	0,50
ENSG00000075568	TMEM131	-3,10	0,58
ENSG00000128594	LRRC4	-3,10	0,69
ENSG00000113068	PFDN1	-3,10	0,43
ENSG00000170296	GABARAP	-3,10	0,69
ENSG00000104824	HNRNPL	-3,10	0,57
ENSG00000169213	RAB3B	-3,10	0,33
ENSG00000254999	BRK1	-3,10	0,53
ENSG00000143315	PIGM	-3,10	0,50

ensembl gene	gene symbol	Score(d)	Fold Change
ENSG00000065970	FOXJ2	-3,10	0,60
ENSG00000133678	TMEM254	-3,10	0,55
ENSG00000168081	PNOC	-3,11	0,47
ENSG00000183291	SELENOF	-3,11	0,71
ENSG00000221916	C19orf73	-3,11	0,56
ENSG00000149084	HSD17B12	-3,11	0,65
ENSG00000145246	ATP10D	-3,11	0,47
ENSG00000185989	RASA3	-3,11	0,44
ENSG00000059145	UNKL	-3,12	0,73
ENSG00000159063	ALG8	-3,12	0,54
ENSG00000170949	ZNF160	-3,13	0,66
ENSG00000173875	ZNF791	-3,13	0,66
ENSG00000163527	STT3B	-3,13	0,65
ENSG00000274641	HIST1H2BO	-3,13	0,37
ENSG00000171634	BPTF	-3,13	0,60
ENSG00000196781	TLE1	-3,13	0,25
ENSG00000181408	UTS2R	-3,13	0,49
ENSG00000168056	LTBP3	-3,13	0,54
ENSG00000115607	IL18RAP	-3,13	0,39
ENSG00000101224	CDC25B	-3,14	0,53
ENSG00000154760	SLFN13	-3,14	0,40
ENSG00000114423	CBLB	-3,14	0,51
ENSG00000011295	TTC19	-3,14	0,52
ENSG00000110077	MS4A6A	-3,14	0,29
ENSG00000197776	KLHDC1	-3,15	0,38
ENSG00000123358	NR4A1	-3,15	0,37
ENSG00000137486	ARRB1	-3,15	0,42
ENSG00000125753	VASP	-3,16	0,56
ENSG00000184363	PKP3	-3,16	0,66
ENSG00000132535	DLG4	-3,16	0,31
ENSG00000213593	TMX2	-3,16	0,50
ENSG00000104957	CCDC130	-3,17	0,63
ENSG00000223501	VPS52	-3,17	0,62
ENSG00000148057	IDNK	-3,17	0,59
ENSG00000226761	TAS2R46	-3,18	0,33
ENSG00000164889	SLC4A2	-3,18	0,58
ENSG00000176261	ZBTB8OS	-3,18	0,48
ENSG00000073754	CD5L	-3,19	0,47
ENSG00000120675	DNAJC15	-3,19	0,51
ENSG00000262180	OCLM	-3,19	0,59
ENSG00000170166	HOXD4	-3,19	0,64
ENSG00000183401	CCDC159	-3,19	0,49
ENSG00000166341	DCHS1	-3,19	0,74
ENSG00000174574	AKIRIN1	-3,20	0,69
ENSG00000071655	MBD3	-3,20	0,55
ENSG00000104529	EEF1D	-3,20	0,70
ENSG00000131196	NFATC1	-3,20	0,49
ENSG00000140525	FANCI	-3,20	0,56
ENSG00000154309	DISP1	-3,20	0,50

ensembl gene	gene symbol	Score(d)	Fold Change
ENSG00000175106	TVP23C	-3,20	0,48
ENSG00000170571	EMB	-3,21	0,68
ENSG00000111328	CDK2AP1	-3,21	0,42
ENSG00000146701	MDH2	-3,21	0,59
ENSG00000155903	RASA2	-3,21	0,51
ENSG00000070087	PFN2	-3,21	0,37
ENSG00000158473	CD1D	-3,22	0,30
ENSG00000198146	ZNF770	-3,22	0,57
ENSG00000104671	DCTN6	-3,22	0,69
ENSG00000160255	ITGB2	-3,22	0,53
ENSG00000099326	MZF1	-3,22	0,62
ENSG00000012061	ERCC1	-3,22	0,63
ENSG00000124588	NQO2	-3,23	0,57
ENSG00000213024	NUP62	-3,23	0,38
ENSG00000163221	S100A12	-3,23	0,14
ENSG00000146963	LUC7L2	-3,23	0,64
ENSG00000250722	SEPP1	-3,23	0,35
ENSG00000140749	IGSF6	-3,23	0,35
ENSG00000185344	ATP6V0A2	-3,23	0,73
ENSG00000166068	SPRED1	-3,23	0,22
ENSG00000157014	TATDN2	-3,23	0,42
ENSG00000101290	CDS2	-3,23	0,44
ENSG00000110079	MS4A4A	-3,23	0,33
ENSG00000135824	RGS8	-3,23	0,64
ENSG00000088387	DOCK9	-3,24	0,48
ENSG00000180386	KRTAP9-7	-3,24	0,69
ENSG00000122417	ODF2L	-3,24	0,45
ENSG00000091879	ANGPT2	-3,25	0,76
ENSG00000118363	SPCS2	-3,25	0,28
ENSG00000114529	C3orf52	-3,25	0,50
ENSG00000165140	FBP1	-3,25	0,22
ENSG00000175634	RPS6KB2	-3,25	0,55
ENSG00000125818	PSMF1	-3,25	0,73
ENSG00000111325	OGFOD2	-3,26	0,58
ENSG00000139289	PHLDA1	-3,26	0,26
ENSG00000187116	LILRA5	-3,27	0,56
ENSG00000137494	ANKRD42	-3,27	0,46
ENSG00000176142	TMEM39A	-3,27	0,71
ENSG00000171552	BCL2L1	-3,27	0,59
ENSG00000160007	ARHGAP35	-3,28	0,64
ENSG00000171766	GATM	-3,28	0,22
ENSG00000197734	C14orf178	-3,28	0,77
ENSG00000073605	GSDMB	-3,28	0,49
ENSG00000112893	MAN2A1	-3,28	0,42
ENSG00000204420	C6orf25	-3,28	0,64
ENSG00000153071	DAB2	-3,29	0,33
ENSG00000184831	APOO	-3,29	0,32
ENSG00000172366	MCRIP2	-3,29	0,42
ENSG00000243789	JMJD7	-3,30	0,47

ensembl gene	gene symbol	Score(d)	Fold Change
ENSG00000198799	LRIG2	-3,30	0,63
ENSG00000159958	TNFRSF13C	-3,30	0,32
ENSG00000072134	EPN2	-3,30	0,53
ENSG00000172663	TMEM134	-3,31	0,70
ENSG00000198088	NUP62CL	-3,31	0,47
ENSG00000213020	ZNF611	-3,31	0,47
ENSG00000198081	ZBTB14	-3,32	0,51
ENSG00000181722	ZBTB20	-3,32	0,53
ENSG00000196712	NF1	-3,32	0,51
ENSG00000115756	HPCAL1	-3,32	0,64
ENSG00000106723	SPIN1	-3,33	0,48
ENSG00000196236	XPNPEP3	-3,33	0,61
ENSG00000126461	SCAF1	-3,33	0,72
ENSG00000105723	GSK3A	-3,33	0,75
ENSG00000168491	CCDC110	-3,34	0,34
ENSG00000075651	PLD1	-3,34	0,51
ENSG00000173166	RAPH1	-3,34	0,18
ENSG00000109079	TNFAIP1	-3,35	0,64
ENSG00000149177	PTPRJ	-3,35	0,28
ENSG00000103018	CYB5B	-3,35	0,42
ENSG00000125703	ATG4C	-3,35	0,43
ENSG00000129538	RNASE1	-3,35	0,27
ENSG00000140022	STON2	-3,35	0,38
ENSG00000154146	NRGN	-3,36	0,58
ENSG00000139687	RB1	-3,36	0,39
ENSG00000126464	PRR12	-3,36	0,66
ENSG00000073756	PTGS2	-3,36	0,27
ENSG00000100320	RBFOX2	-3,37	0,45
ENSG00000138336	TET1	-3,38	0,37
ENSG00000219626	FAM228B	-3,38	0,58
ENSG00000116729	WLS	-3,38	0,44
ENSG00000068971	PPP2R5B	-3,38	0,62
ENSG00000130234	ACE2	-3,38	0,59
ENSG00000198837	DENND4B	-3,38	0,57
ENSG00000178229	ZNF543	-3,38	0,59
ENSG00000204424	LY6G6F	-3,39	0,58
ENSG00000160588	MPZL3	-3,40	0,43
ENSG00000172716	SLFN11	-3,41	0,27
ENSG00000130723	PRRC2B	-3,41	0,74
ENSG00000205497	OR51A4	-3,42	0,52
ENSG00000149516	MS4A3	-3,42	0,21
ENSG00000116668	SWT1	-3,43	0,48
ENSG00000120333	MRPS14	-3,46	0,36
ENSG00000111906	HDDC2	-3,46	0,56
ENSG00000279342	AP000866.1	-3,46	0,37
ENSG00000212128	TAS2R13	-3,46	0,42
ENSG00000178115	GOLGA8Q	-3,47	0,36
ENSG00000101162	TUBB1	-3,48	0,41
ENSG00000063854	HAGH	-3,48	0,43

ensembl gene	gene symbol	Score(d)	Fold Change
ENSG00000172775	FAM192A	-3,48	0,61
ENSG00000105538	RASIP1	-3,49	0,75
ENSG00000166145	SPINT1	-3,49	0,39
ENSG00000100564	PIGH	-3,49	0,65
ENSG00000133983	COX16	-3,49	0,58
ENSG00000212126	TAS2R50	-3,50	0,37
ENSG00000204977	TRIM13	-3,51	0,43
ENSG00000153936	HS2ST1	-3,51	0,47
ENSG00000132003	ZSWIM4	-3,52	0,57
ENSG00000095303	PTGS1	-3,52	0,52
ENSG00000165028	NIPSNAP3B	-3,52	0,33
ENSG00000211584	SLC48A1	-3,52	0,59
ENSG00000181631	P2RY13	-3,52	0,39
ENSG00000283199	ABC13-47488600E17.1	-3,52	0,55
ENSG00000168298	HIST1H1E	-3,53	0,50
ENSG00000136869	TLR4	-3,53	0,49
ENSG00000107669	ATE1	-3,53	0,66
ENSG00000198879	SFMBT2	-3,54	0,45
ENSG00000018280	SLC11A1	-3,54	0,46
ENSG00000079931	MOXD1	-3,54	0,20
ENSG00000177463	NR2C2	-3,55	0,56
ENSG00000156858	PRR14	-3,55	0,56
ENSG00000181381	DDX60L	-3,55	0,46
ENSG00000069966	GNB5	-3,56	0,41
ENSG00000149131	SERPING1	-3,57	0,31
ENSG00000122592	HOXA7	-3,57	0,68
ENSG00000139174	PRICKLE1	-3,57	0,41
ENSG00000138639	ARHGAP24	-3,58	0,34
ENSG00000157869	RAB28	-3,58	0,55
ENSG00000124177	CHD6	-3,58	0,48
ENSG00000179833	SERTAD2	-3,59	0,36
ENSG00000111707	SUDS3	-3,59	0,62
ENSG00000108557	RAI1	-3,60	0,66
ENSG00000117020	AKT3	-3,61	0,21
ENSG00000128272	ATF4	-3,61	0,72
ENSG00000157379	DHRS1	-3,61	0,52
ENSG00000134243	SORT1	-3,62	0,24
ENSG00000114125	RNF7	-3,62	0,33
ENSG00000244734	HBB	-3,62	0,14
ENSG00000233822	HIST1H2BN	-3,62	0,53
ENSG00000168389	MFSD2A	-3,63	0,35
ENSG00000204482	LST1	-3,63	0,47
ENSG00000198369	SPRED2	-3,63	0,44
ENSG00000185267	CDNF	-3,63	0,55
ENSG00000169385	RNASE2	-3,63	0,15
ENSG00000154237	LRRK1	-3,64	0,32
ENSG00000149547	EI24	-3,65	0,47
ENSG00000166405	RIC3	-3,65	0,44
ENSG00000111269	CREBL2	-3,66	0,50

ensembl gene	gene symbol	Score(d)	Fold Change
ENSG00000212124	TAS2R19	-3,66	0,28
ENSG00000105352	CEACAM4	-3,67	0,58
ENSG00000144711	IQSEC1	-3,67	0,47
ENSG00000139193	CD27	-3,69	0,08
ENSG00000158773	USF1	-3,69	0,51
ENSG00000155749	ALS2CR12	-3,69	0,45
ENSG00000196911	KPNA5	-3,70	0,60
ENSG00000169696	ASPSCR1	-3,70	0,61
ENSG00000078699	CBFA2T2	-3,70	0,46
ENSG00000167766	ZNF83	-3,71	0,28
ENSG00000173376	NDNF	-3,71	0,20
ENSG00000205659	LIN52	-3,72	0,53
ENSG00000231389	HLA-DPA1	-3,72	0,31
ENSG00000136144	RCBTB1	-3,72	0,48
ENSG00000136104	RNASEH2B	-3,73	0,51
ENSG00000111271	ACAD10	-3,73	0,49
ENSG00000146674	IGFBP3	-3,74	0,52
ENSG00000108840	HDAC5	-3,74	0,55
ENSG00000021461	CYP3A43	-3,74	0,37
ENSG00000172765	TMCC1	-3,74	0,62
ENSG00000133800	LYVE1	-3,75	0,22
ENSG00000169397	RNASE3	-3,76	0,11
ENSG00000160094	ZNF362	-3,76	0,66
ENSG00000186815	TPCN1	-3,76	0,37
ENSG00000132704	FCRL2	-3,76	0,21
ENSG00000072121	ZFYVE26	-3,76	0,65
ENSG00000160213	CSTB	-3,77	0,59
ENSG00000064115	TM7SF3	-3,77	0,40
ENSG00000153291	SLC25A27	-3,78	0,15
ENSG00000119616	FCF1	-3,79	0,63
ENSG00000107185	RGP1	-3,79	0,70
ENSG00000137513	NARS2	-3,79	0,50
ENSG00000119638	NEK9	-3,79	0,56
ENSG00000057935	MTA3	-3,81	0,49
ENSG00000177666	PNPLA2	-3,81	0,52
ENSG00000147535	PLPP5	-3,82	0,67
ENSG00000130702	LAMA5	-3,82	0,45
ENSG00000091972	CD200	-3,82	0,20
ENSG00000128218	VPREB3	-3,83	0,12
ENSG00000254598	CSNK2A3	-3,83	0,55
ENSG00000135046	ANXA1	-3,84	0,13
ENSG00000130347	RTN4IP1	-3,85	0,44
ENSG00000086200	IPO11	-3,86	0,42
ENSG00000079691	LRRC16A	-3,86	0,41
ENSG00000135185	TMEM243	-3,87	0,49
ENSG00000101138	CSTF1	-3,88	0,63
ENSG00000090382	LYZ	-3,89	0,26
ENSG00000157429	ZNF19	-3,90	0,60
ENSG00000241058	NSUN6	-3,90	0,51

ensembl gene	gene symbol	Score(d)	Fold Change
ENSG00000197054	ZNF763	-3,90	0,42
ENSG00000113638	TTC33	-3,90	0,44
ENSG00000110717	NDUFS8	-3,91	0,44
ENSG00000107249	GLIS3	-3,91	0,15
ENSG00000082074	FYB	-3,91	0,32
ENSG00000105771	SMG9	-3,91	0,61
ENSG00000008226	DLEC1	-3,92	0,51
ENSG00000198900	TOP1	-3,92	0,59
ENSG00000198121	LPAR1	-3,93	0,57
ENSG00000110713	NUP98	-3,94	0,54
ENSG00000168288	MMADHC	-3,95	0,40
ENSG00000144791	LIMD1	-3,96	0,43
ENSG00000076108	BAZ2A	-3,96	0,51
ENSG00000160209	PDXK	-3,96	0,51
ENSG00000136715	SAP130	-3,97	0,46
ENSG00000106603	COA1	-3,99	0,54
ENSG00000176102	CSTF3	-4,00	0,49
ENSG00000078747	ITCH	-4,01	0,78
ENSG00000174282	ZBTB4	-4,01	0,62
ENSG00000182986	ZNF320	-4,01	0,41
ENSG00000196227	FAM217B	-4,02	0,44
ENSG00000171735	CAMTA1	-4,02	0,64
ENSG00000099250	NRP1	-4,02	0,18
ENSG00000188732	FAM221A	-4,02	0,36
ENSG00000241106	HLA-DOB	-4,03	0,38
ENSG00000121481	RNF2	-4,03	0,56
ENSG00000154016	GRAP	-4,03	0,48
ENSG00000214078	CPNE1	-4,05	0,49
ENSG00000179104	TMTC2	-4,06	0,48
ENSG00000034713	GABARAPL2	-4,07	0,45
ENSG00000196476	C20orf96	-4,09	0,51
ENSG00000274750	HIST1H3E	-4,10	0,27
ENSG00000198417	MT1F	-4,11	0,19
ENSG00000167280	ENGASE	-4,12	0,53
ENSG00000139352	ASCL1	-4,13	0,60
ENSG00000148358	GPR107	-4,14	0,61
ENSG00000107562	CXCL12	-4,16	0,17
ENSG00000170348	TMED10	-4,17	0,55
ENSG00000212127	TAS2R14	-4,17	0,30
ENSG00000161911	TREML1	-4,18	0,36
ENSG00000131100	ATP6V1E1	-4,19	0,52
ENSG00000130227	XPO7	-4,19	0,62
ENSG00000111676	ATN1	-4,19	0,50
ENSG00000180357	ZNF609	-4,21	0,66
ENSG00000164406	LEAP2	-4,22	0,58
ENSG00000196735	HLA-DQA1	-4,22	0,26
ENSG00000120727	PAIP2	-4,22	0,53
ENSG00000134369	NAV1	-4,24	0,46
ENSG00000165702	GFI1B	-4,25	0,68

ensembl gene	gene symbol	Score(d)	Fold Change
ENSG00000197980	LEKR1	-4,25	0,54
ENSG00000114956	DGUOK	-4,25	0,58
ENSG00000136205	TNS3	-4,27	0,30
ENSG00000259207	ITGB3	-4,31	0,21
ENSG00000117009	KMO	-4,31	0,09
ENSG00000064225	ST3GAL6	-4,32	0,48
ENSG00000100934	SEC23A	-4,33	0,50
ENSG00000255837	TAS2R20	-4,33	0,27
ENSG00000118689	FOXO3	-4,33	0,29
ENSG00000139190	VAMP1	-4,34	0,32
ENSG00000116750	UCHL5	-4,34	0,47
ENSG00000107984	DKK1	-4,35	0,19
ENSG00000104866	PPP1R37	-4,36	0,43
ENSG00000172915	NBEA	-4,37	0,29
ENSG00000162373	BEND5	-4,38	0,46
ENSG00000214413	BBIP1	-4,40	0,48
ENSG00000139318	DUSP6	-4,45	0,27
ENSG00000006468	ETV1	-4,46	0,07
ENSG00000139737	SLAIN1	-4,46	0,40
ENSG00000161267	BDH1	-4,47	0,27
ENSG00000106415	GLCCI1	-4,48	0,47
ENSG00000188215	DCUN1D3	-4,48	0,41
ENSG00000135503	ACVR1B	-4,48	0,38
ENSG00000163785	RYK	-4,52	0,53
ENSG00000100647	SUSD6	-4,52	0,38
ENSG00000102897	LYRM1	-4,53	0,51
ENSG00000112977	DAP	-4,54	0,51
ENSG00000163513	TGFBR2	-4,55	0,34
ENSG00000172728	FUT10	-4,55	0,50
ENSG00000137814	HAUS2	-4,59	0,42
ENSG00000119688	ABCD4	-4,60	0,58
ENSG00000099364	FBXL19	-4,63	0,59
ENSG00000197959	DNM3	-4,72	0,51
ENSG00000237441	RGL2	-4,73	0,42
ENSG00000119669	IRF2BPL	-4,73	0,55
ENSG00000143222	UFC1	-4,74	0,55
ENSG00000111341	MGP	-4,77	0,38
ENSG00000140382	HMG20A	-4,79	0,46
ENSG00000119711	ALDH6A1	-4,84	0,39
ENSG00000138738	PRDM5	-4,85	0,22
ENSG00000119650	IFT43	-4,85	0,55
ENSG00000154553	PDLIM3	-4,87	0,18
ENSG00000004534	RBM6	-4,88	0,49
ENSG00000186918	ZNF395	-4,89	0,40
ENSG00000105369	CD79A	-4,89	0,11
ENSG00000136267	DGKB	-4,89	0,54
ENSG00000134215	VAV3	-4,94	0,23
ENSG00000065457	ADAT1	-4,94	0,31
ENSG00000177045	SIX5	-4,94	0,56

ensembl gene	gene symbol	Score(d)	Fold Change
ENSG00000121318	TAS2R10	-4,96	0,29
ENSG00000135218	CD36	-4,97	0,24
ENSG00000196628	TCF4	-5,01	0,12
ENSG00000160999	SH2B2	-5,06	0,24
ENSG00000165685	TMEM52B	-5,10	0,21
ENSG00000163736	PPBP	-5,14	0,11
ENSG00000213999	MEF2B	-5,17	0,59
ENSG00000182985	CADM1	-5,18	0,04
ENSG00000184445	KNTC1	-5,21	0,39
ENSG00000005961	ITGA2B	-5,23	0,18
ENSG00000139132	FGD4	-5,26	0,19
ENSG00000120805	ARL1	-5,35	0,41
ENSG00000244405	ETV5	-5,41	0,17
ENSG00000144645	OSBPL10	-5,43	0,05
ENSG00000219545	UMAD1	-5,58	0,42
ENSG00000152818	UTRN	-5,64	0,25
ENSG00000070610	GBA2	-5,89	0,45
ENSG00000180953	ST20	-6,55	0,34
ENSG00000176788	BASP1	-7,06	0,05

Supplementary Table S2. List of 4847 differentially expressed coding genes in 15 pPCL compared to 50 MM cases, by SAM analysis at FDR<10%. Transcripts are ordered according to SAM (d) score.

ensembl gene	gene symbol	Score(d)	Fold Change
ENSG00000101182	PSMA7	7,75	2,54
ENSG00000211460	TSN	7,59	1,85
ENSG00000198522	GPN1	7,10	1,77
ENSG00000120437	ACAT2	6,94	2,27
ENSG00000110321	EIF4G2	6,92	1,62
ENSG00000180957	PITPNB	6,72	2,15
ENSG00000165650	PDZD8	6,60	1,78
ENSG00000257103	LSM14A	6,54	2,38
ENSG00000100281	HMGXB4	6,30	1,89
ENSG00000170881	RNF139	6,27	1,80
ENSG00000105849	TWISTNB	6,25	2,41
ENSG00000173486	FKBP2	6,20	2,57
ENSG00000152332	UHMK1	6,07	1,92
ENSG00000007520	TSR3	6,05	1,82
ENSG00000177917	ARL6IP6	6,00	1,81
ENSG00000131746	TNS4	5,97	2,11
ENSG00000170759	KIF5B	5,85	1,88
ENSG00000128191	DGCR8	5,75	1,92
ENSG00000174839	DENND6A	5,75	1,86
ENSG00000100221	JOSD1	5,70	1,85
ENSG00000101367	MAPRE1	5,70	1,51
ENSG00000198951	NAGA	5,69	2,23
ENSG00000185947	ZNF267	5,68	3,20
ENSG00000100243	CYB5R3	5,65	1,62
ENSG00000139977	NAA30	5,65	1,87
ENSG00000162783	IER5	5,63	2,70
ENSG00000244624	KRTAP20-1	5,55	2,09
ENSG00000162433	AK4	5,52	1,75
ENSG00000275700	AATF	5,49	1,75
ENSG00000125944	HNRNPR	5,49	1,64
ENSG00000132823	OSER1	5,48	2,38
ENSG00000183891	TTC32	5,48	2,28
ENSG00000274523	RCC1L	5,48	1,40
ENSG00000125457	MIF4GD	5,45	1,73
ENSG00000124191	TOX2	5,45	2,16
ENSG00000118804	STBD1	5,44	1,88
ENSG00000256053	APOPT1	5,42	1,83
ENSG00000013588	GPRC5A	5,41	2,25
ENSG00000085274	MYNN	5,37	1,59
ENSG00000181061	HIGD1A	5,37	2,10
ENSG00000156976	EIF4A2	5,34	3,11
ENSG00000107960	OBFC1	5,34	1,58
ENSG00000154723	ATP5J	5,32	1,91
ENSG00000164091	WDR82	5,31	1,56
ENSG00000100028	SNRPD3	5,29	1,79

ensembl gene	gene symbol	Score(d)	Fold Change
ENSG00000106245	BUD31	5,28	1,75
ENSG00000056097	ZFR	5,27	1,54
ENSG00000115053	NCL	5,24	1,55
ENSG00000168488	ATXN2L	5,21	1,48
ENSG00000114209	PDCD10	5,19	1,69
ENSG00000196141	SPATS2L	5,17	2,44
ENSG00000185627	PSMD13	5,16	1,73
ENSG00000126247	CAPNS1	5,14	1,55
ENSG00000164022	AIMP1	5,14	1,40
ENSG00000138071	ACTR2	5,13	1,38
ENSG00000163162	RNF149	5,13	2,39
ENSG00000169251	NMD3	5,13	1,47
ENSG00000173432	SAA1	5,12	1,59
ENSG00000136718	IMP4	5,12	1,64
ENSG00000141385	AFG3L2	5,09	1,76
ENSG00000091140	DLD	5,08	1,81
ENSG00000134077	THUMPD3	5,07	1,41
ENSG00000182552	RWDD4	5,07	2,25
ENSG00000047410	TPR	5,07	1,64
ENSG00000083937	CHMP2B	5,07	1,63
ENSG00000023191	RNH1	5,05	1,82
ENSG00000126945	HNRNPH2	5,05	1,80
ENSG00000204843	DCTN1	5,05	1,41
ENSG00000183648	NDUFB1	5,04	2,51
ENSG00000071127	WDR1	5,03	1,69
ENSG00000116473	RAP1A	5,03	2,04
ENSG00000131981	LGALS3	5,03	3,02
ENSG00000101844	ATG4A	5,00	1,54
ENSG00000163714	U2SURP	4,99	1,52
ENSG00000099783	HNRNPM	4,99	1,55
ENSG00000163636	PSMD6	4,99	1,57
ENSG00000105321	CCDC9	4,98	1,97
ENSG00000108055	SMC3	4,98	1,47
ENSG00000176225	RTTN	4,98	2,57
ENSG00000164713	BRI3	4,97	1,62
ENSG00000185730	ZNF696	4,95	1,33
ENSG00000205937	RNPS1	4,93	1,59
ENSG00000138279	ANXA7	4,90	1,69
ENSG00000147155	EBP	4,88	1,67
ENSG00000138495	COX17	4,88	2,35
ENSG00000166224	SGPL1	4,88	1,66
ENSG00000139350	NEDD1	4,87	1,68
ENSG00000135269	TES	4,87	1,87
ENSG00000143977	SNRPG	4,86	1,71
ENSG00000196924	FLNA	4,86	2,42
ENSG00000101391	CDK5RAP1	4,85	1,59
ENSG00000107625	DDX50	4,85	1,58
ENSG00000241685	ARPC1A	4,85	1,77
ENSG00000082153	BZW1	4,84	1,75

ensembl gene	gene symbol	Score(d)	Fold Change
ENSG00000176903	PNMA1	4,84	2,11
ENSG00000075975	MKRN2	4,84	1,43
ENSG00000146232	NFKBIE	4,83	2,10
ENSG00000061794	MRPS35	4,83	2,00
ENSG00000173598	NUDT4	4,82	1,92
ENSG00000085978	ATG16L1	4,82	1,56
ENSG00000158710	TAGLN2	4,81	2,40
ENSG00000158526	TSR2	4,80	1,59
ENSG00000108107	RPL28	4,78	1,61
ENSG00000074800	ENO1	4,77	1,65
ENSG00000055044	NOP58	4,77	1,53
ENSG00000198231	DDX42	4,77	1,53
ENSG00000102241	HTATSF1	4,76	1,61
ENSG00000183520	UTP11	4,76	1,60
ENSG00000171566	PLRG1	4,75	1,88
ENSG00000171262	FAM98B	4,74	1,70
ENSG00000151012	SLC7A11	4,74	4,03
ENSG00000089048	ESF1	4,74	1,94
ENSG00000186577	C6orf1	4,73	1,44
ENSG00000162645	GBP2	4,73	4,57
ENSG00000068366	ACSL4	4,72	1,90
ENSG00000179010	MRFAP1	4,72	1,67
ENSG00000023909	GCLM	4,71	1,83
ENSG00000103174	NAGPA	4,69	1,86
ENSG00000042286	AIFM2	4,68	1,39
ENSG00000159202	UBE2Z	4,67	1,59
ENSG00000113739	STC2	4,66	2,41
ENSG00000165733	BMS1	4,66	1,50
ENSG00000125352	RNF113A	4,65	1,39
ENSG00000174744	BRMS1	4,64	1,41
ENSG00000144567	FAM134A	4,63	1,51
ENSG00000113141	IK	4,63	1,61
ENSG00000183340	JRKL	4,62	1,45
ENSG00000126698	DNAJC8	4,62	1,43
ENSG00000154001	PPP2R5E	4,61	1,70
ENSG00000013364	MVP	4,61	2,14
ENSG00000143774	GUK1	4,60	1,43
ENSG00000183527	PSMG1	4,60	1,83
ENSG00000119787	ATL2	4,60	1,61
ENSG00000130956	HABP4	4,60	2,30
ENSG00000186792	HYAL3	4,59	1,50
ENSG00000163319	MRPS18C	4,58	1,52
ENSG00000007168	PAFAH1B1	4,58	1,54
ENSG00000130772	MED18	4,57	1,42
ENSG00000076003	MCM6	4,56	1,89
ENSG00000164219	PGGT1B	4,56	1,59
ENSG00000006125	AP2B1	4,55	1,48
ENSG00000139343	SNRPF	4,55	1,62
ENSG00000175104	TRAF6	4,54	1,95

ensembl gene	gene symbol	Score(d)	Fold Change
ENSG00000116874	WARS2	4,54	1,84
ENSG00000132872	SYT4	4,53	1,45
ENSG00000103342	GSPT1	4,53	1,69
ENSG00000158480	SPATA2	4,52	1,83
ENSG00000188886	ASTL	4,52	1,42
ENSG00000100796	PPP4R3A	4,51	1,65
ENSG00000114115	RBP1	4,51	2,95
ENSG00000114784	EIF1B	4,51	2,13
ENSG00000160087	UBE2J2	4,50	1,49
ENSG00000153879	CEBPG	4,50	1,88
ENSG00000133142	TCEAL4	4,48	1,83
ENSG00000123728	RAP2C	4,48	1,50
ENSG00000128534	LSM8	4,47	1,66
ENSG00000158615	PPP1R15B	4,47	1,60
ENSG00000068308	OTUD5	4,47	1,38
ENSG00000179331	RAB39A	4,46	1,68
ENSG00000120438	TCP1	4,46	1,44
ENSG00000173269	MMRN2	4,45	1,35
ENSG00000007237	GAS7	4,44	1,92
ENSG00000112972	HMGCS1	4,44	1,65
ENSG00000117228	GBP1	4,44	4,28
ENSG00000166226	CCT2	4,43	1,81
ENSG00000123685	BATF3	4,43	2,06
ENSG00000196072	BLOC1S2	4,42	1,97
ENSG00000109047	RCVRN	4,42	1,47
ENSG00000144802	NFKBIZ	4,41	2,36
ENSG00000235169	SMIM1	4,41	1,34
ENSG00000179950	PUF60	4,41	1,44
ENSG00000005075	POLR2J	4,41	1,58
ENSG00000118454	ANKRD13C	4,41	1,68
ENSG00000091039	OSBPL8	4,40	1,57
ENSG00000176485	PLA2G16	4,39	2,11
ENSG00000151651	ADAM8	4,39	1,66
ENSG00000185883	ATP6V0C	4,38	1,29
ENSG00000100100	PIK3IP1	4,38	2,05
ENSG00000056558	TRAF1	4,37	1,48
ENSG00000274068	RP11-475E11.9	4,37	1,52
ENSG00000178913	TAF7	4,37	1,50
ENSG00000125726	CD70	4,36	1,51
ENSG00000125356	NDUFA1	4,35	1,46
ENSG00000173175	ADCY5	4,35	1,62
ENSG00000155959	VBP1	4,34	1,63
ENSG00000196151	WDSUB1	4,34	1,60
ENSG00000178952	TUFM	4,34	1,60
ENSG00000183484	GPR132	4,34	1,73
ENSG00000073584	SMARCE1	4,33	1,70
ENSG00000100106	TRIOBP	4,33	1,69
ENSG00000077147	TM9SF3	4,33	1,30
ENSG00000100316	RPL3	4,33	1,62

ensembl gene	gene symbol	Score(d)	Fold Change
ENSG00000142227	EMP3	4,33	1,44
ENSG00000198677	TTC37	4,32	1,60
ENSG00000185246	PRPF39	4,32	1,74
ENSG00000130935	NOL11	4,31	1,78
ENSG00000198783	ZNF830	4,31	1,66
ENSG00000147119	CHST7	4,30	1,78
ENSG00000006634	DBF4	4,30	1,70
ENSG00000204314	PRRT1	4,30	1,36
ENSG00000126353	CCR7	4,30	2,55
ENSG00000154359	LONRF1	4,30	2,35
ENSG00000171612	SLC25A33	4,29	1,79
ENSG00000156261	CCT8	4,29	1,75
ENSG00000100941	PNN	4,29	1,75
ENSG00000170242	USP47	4,29	1,83
ENSG00000143344	RGL1	4,28	3,56
ENSG00000133641	C12orf29	4,28	1,52
ENSG00000106459	NRF1	4,28	1,64
ENSG00000077549	CAPZB	4,27	1,37
ENSG00000164430	MB21D1	4,27	1,83
ENSG00000138785	INTS12	4,27	1,46
ENSG00000130703	OSBPL2	4,27	1,44
ENSG00000091317	CMTM6	4,26	1,68
ENSG00000136758	YME1L1	4,26	1,40
ENSG00000115541	HSPE1	4,26	2,93
ENSG00000173848	NET1	4,26	2,37
ENSG00000182698	RESP18	4,26	1,22
ENSG00000005844	ITGAL	4,25	2,28
ENSG00000269404	SPIB	4,25	1,81
ENSG00000162607	USP1	4,25	1,70
ENSG00000155380	SLC16A1	4,24	1,85
ENSG00000174109	C16orf91	4,24	1,37
ENSG00000054118	THRAP3	4,24	1,47
ENSG00000110660	SLC35F2	4,24	2,11
ENSG00000163719	MTMR14	4,23	1,61
ENSG00000167553	TUBA1C	4,23	1,33
ENSG00000147140	NONO	4,22	1,41
ENSG00000100603	SNW1	4,22	1,51
ENSG00000186806	VSIG10L	4,22	1,34
ENSG00000101695	RNF125	4,22	3,24
ENSG00000004961	HCCS	4,22	1,57
ENSG00000123136	DDX39A	4,22	1,60
ENSG00000187170	LCE4A	4,22	1,28
ENSG00000239900	ADSL	4,22	1,72
ENSG00000145022	TCTA	4,21	1,58
ENSG00000113575	PPP2CA	4,21	1,68
ENSG00000085721	RRN3	4,21	1,58
ENSG00000107581	EIF3A	4,21	1,28
ENSG00000165732	DDX21	4,20	1,61
ENSG00000115816	CEBPZ	4,20	1,65

ensembl gene	gene symbol	Score(d)	Fold Change
ENSG00000255112	CHMP1B	4,18	1,50
ENSG00000134265	NAPG	4,18	1,42
ENSG00000137404	NRM	4,17	1,69
ENSG00000120063	GNA13	4,17	1,52
ENSG00000147526	TACC1	4,17	2,47
ENSG00000181191	PJA1	4,17	1,57
ENSG00000236543	RP11-98L5.5	4,16	1,38
ENSG00000006652	IFRD1	4,16	1,86
ENSG00000116852	KIF21B	4,16	1,65
ENSG00000095485	CWF19L1	4,15	1,50
ENSG00000184575	XPOT	4,15	1,74
ENSG00000146757	ZNF92	4,15	1,59
ENSG00000165195	PIGA	4,14	2,02
ENSG00000067900	ROCK1	4,14	1,50
ENSG00000155729	KCTD18	4,13	1,38
ENSG00000236334	PPIAL4G	4,13	1,53
ENSG00000112031	MTRF1L	4,13	1,77
ENSG00000196214	ZNF766	4,13	1,61
ENSG00000075089	ACTR6	4,13	1,77
ENSG00000106153	CHCHD2	4,13	1,72
ENSG00000277791	PSMB3	4,12	1,49
ENSG00000171497	PPID	4,12	1,81
ENSG00000169967	MAP3K2	4,11	1,61
ENSG00000164758	MED30	4,11	1,69
ENSG00000107537	PHYH	4,11	1,64
ENSG00000109445	ZNF330	4,10	1,57
ENSG00000116809	ZBTB17	4,10	1,34
ENSG00000168297	PXK	4,10	1,92
ENSG00000134186	PRPF38B	4,09	1,61
ENSG00000168884	TNIP2	4,08	1,52
ENSG00000075223	SEMA3C	4,08	1,87
ENSG00000127125	PPCS	4,08	1,63
ENSG00000171202	TMEM126A	4,07	1,68
ENSG00000170088	TMEM192	4,07	1,42
ENSG00000189319	FAM53B	4,07	2,07
ENSG00000147251	DOCK11	4,07	2,21
ENSG00000066427	ATXN3	4,06	1,59
ENSG00000132436	FIGNL1	4,06	1,95
ENSG00000119403	PHF19	4,06	1,69
ENSG00000083807	SLC27A5	4,05	1,90
ENSG00000004700	RECQL	4,05	1,70
ENSG00000156232	WHAMM	4,05	1,65
ENSG00000136842	TMOD1	4,05	2,23
ENSG00000131871	VIMP	4,05	1,51
ENSG00000162929	KIAA1841	4,04	1,57
ENSG00000143815	LBR	4,04	1,84
ENSG00000099995	SF3A1	4,04	1,49
ENSG00000067334	DNTTIP2	4,04	1,68
ENSG00000123143	PKN1	4,04	1,45

ensembl gene	gene symbol	Score(d)	Fold Change
ENSG00000178287	SPAG11A	4,04	1,63
ENSG00000158290	CUL4B	4,03	1,44
ENSG00000163584	RPL22L1	4,03	2,25
ENSG00000177854	TMEM187	4,03	1,53
ENSG00000138231	DBR1	4,03	1,46
ENSG00000173545	ZNF622	4,03	1,57
ENSG00000134198	TSPAN2	4,03	2,81
ENSG00000123240	OPTN	4,03	1,63
ENSG00000164209	SLC25A46	4,02	1,36
ENSG00000175110	MRPS22	4,02	1,44
ENSG00000214309	MBLAC1	4,02	1,79
ENSG00000197930	ERO1A	4,02	1,43
ENSG00000130489	SCO2	4,02	1,37
ENSG00000189306	RRP7A	4,02	1,27
ENSG00000163743	RCHY1	4,02	1,45
ENSG00000048544	MRPS10	4,01	1,41
ENSG00000064419	TNPO3	4,01	1,28
ENSG00000157796	WDR19	4,01	1,86
ENSG00000259075	POC1B-GALNT4	4,01	1,85
ENSG00000100003	SEC14L2	4,01	2,16
ENSG00000009780	FAM76A	4,01	1,41
ENSG00000129083	COPB1	4,01	2,32
ENSG00000008018	PSMB1	4,00	1,53
ENSG00000205531	NAP1L4	3,99	1,38
ENSG00000069345	DNAJA2	3,99	1,56
ENSG00000107863	ARHGAP21	3,99	2,05
ENSG00000119041	GTF3C3	3,98	1,52
ENSG00000119185	ITGB1BP1	3,98	1,56
ENSG00000240542	KRTAP9-1	3,98	1,64
ENSG00000136436	CALCOCO2	3,98	1,41
ENSG00000087470	DNM1L	3,98	1,48
ENSG00000149136	SSRP1	3,98	1,68
ENSG00000102007	PLP2	3,97	1,38
ENSG00000213213	CCDC183	3,97	1,62
ENSG00000101350	KIF3B	3,97	1,42
ENSG00000197885	NKIRAS1	3,97	2,05
ENSG00000237765	FAM200B	3,96	1,54
ENSG00000224960	PPP4R3CP	3,96	1,94
ENSG00000163041	H3F3A	3,96	1,56
ENSG00000184545	DUSP8	3,96	1,77
ENSG00000073712	FERMT2	3,96	3,38
ENSG00000102858	MGRN1	3,95	1,39
ENSG00000136754	ABI1	3,95	1,47
ENSG00000128309	MPST	3,94	1,28
ENSG00000121594	CD80	3,94	1,71
ENSG00000133703	KRAS	3,94	1,62
ENSG00000155926	SLA	3,93	2,75
ENSG00000197928	ZNF677	3,93	2,07
ENSG00000112701	SENP6	3,93	1,34

ensembl gene	gene symbol	Score(d)	Fold Change
ENSG00000107897	ACBD5	3,92	1,41
ENSG00000158669	GPAT4	3,92	1,51
ENSG00000079134	THOC1	3,92	1,47
ENSG00000165502	RPL36AL	3,92	1,49
ENSG00000167613	LAIR1	3,92	2,83
ENSG00000197780	TAF13	3,92	1,67
ENSG00000183735	TBK1	3,92	1,80
ENSG00000080824	HSP90AA1	3,91	1,95
ENSG00000123983	ACSL3	3,91	1,47
ENSG00000103507	BCKDK	3,91	1,50
ENSG00000103502	CDIPT	3,91	1,44
ENSG00000162520	SYNC	3,91	1,39
ENSG00000163946	FAM208A	3,90	1,52
ENSG00000122565	CBX3	3,90	1,64
ENSG00000087191	PSMC5	3,90	1,70
ENSG00000163634	THOC7	3,89	1,61
ENSG00000174885	NLRP6	3,89	1,66
ENSG00000031698	SARS	3,89	1,81
ENSG00000182944	EWSR1	3,88	1,40
ENSG00000107485	GATA3	3,88	2,10
ENSG00000015171	ZMYND11	3,88	1,44
ENSG00000114416	FXR1	3,88	1,41
ENSG00000100056	DGCR14	3,88	1,31
ENSG00000161057	PSMC2	3,87	1,46
ENSG00000171100	MTM1	3,87	1,60
ENSG00000132792	CTNNBL1	3,86	1,38
ENSG00000086061	DNAJA1	3,86	1,72
ENSG00000115339	GALNT3	3,86	2,92
ENSG00000184144	CNTN2	3,86	1,50
ENSG00000153147	SMARCA5	3,86	1,31
ENSG00000119326	CTNNAL1	3,86	2,49
ENSG00000144741	SLC25A26	3,85	1,38
ENSG00000171102	OBP2B	3,85	1,74
ENSG00000116691	MIIP	3,85	1,40
ENSG00000196967	ZNF585A	3,85	1,66
ENSG00000131504	DIAPH1	3,84	1,70
ENSG00000154978	VOPP1	3,84	2,07
ENSG00000119541	VPS4B	3,84	1,50
ENSG00000125821	DTD1	3,84	1,51
ENSG00000099800	TIMM13	3,84	1,76
ENSG00000108588	CCDC47	3,83	1,33
ENSG00000138081	FBXO11	3,83	1,43
ENSG00000101474	APMAP	3,83	1,43
ENSG00000198758	EPS8L3	3,83	1,42
ENSG00000023734	STRAP	3,83	1,48
ENSG00000114742	WDR48	3,83	1,84
ENSG00000215906	LACTBL1	3,82	1,21
ENSG00000232388	LINC00493	3,82	1,68
ENSG00000162441	LZIC	3,82	1,50

ensembl gene	gene symbol	Score(d)	Fold Change
ENSG00000104856	RELB	3,82	1,73
ENSG00000156269	NAA11	3,81	1,60
ENSG00000138068	SULT6B1	3,81	1,29
ENSG00000130764	LRRC47	3,81	1,36
ENSG00000176531	PHLDB3	3,80	1,45
ENSG00000164221	CCDC112	3,80	1,61
ENSG00000143971	ETAA1	3,80	1,76
ENSG00000151576	QTRT2	3,79	1,46
ENSG00000137955	RABGGTB	3,79	1,62
ENSG00000204659	CBY3	3,79	1,41
ENSG00000018510	AGPS	3,79	1,39
ENSG00000187522	HSPA14	3,79	1,65
ENSG00000173660	UQCRH	3,79	1,70
ENSG00000104312	RIPK2	3,78	1,66
ENSG00000123737	EXOSC9	3,78	1,37
ENSG00000156795	WDYHV1	3,78	1,50
ENSG00000176853	FAM91A1	3,78	1,35
ENSG00000035681	NSMAF	3,78	1,77
ENSG00000140575	IQGAP1	3,78	1,38
ENSG00000130766	SESN2	3,78	2,31
ENSG00000170802	FOXN2	3,78	1,50
ENSG00000183549	ACSM5	3,78	1,46
ENSG00000116171	SCP2	3,77	1,54
ENSG00000081377	CDC14B	3,77	1,93
ENSG00000092201	SUPT16H	3,77	1,64
ENSG00000196639	HRH1	3,77	1,28
ENSG00000144744	UBA3	3,76	1,32
ENSG00000181523	SGSH	3,76	1,50
ENSG00000172538	FAM170B	3,76	1,21
ENSG00000155906	RMND1	3,76	1,59
ENSG00000101146	RAE1	3,76	1,40
ENSG00000101052	IFT52	3,75	1,55
ENSG00000112308	C6orf62	3,75	1,41
ENSG00000154640	BTG3	3,75	2,29
ENSG00000262919	FAM58A	3,75	1,37
ENSG00000104549	SQLE	3,75	2,25
ENSG00000254087	LYN	3,75	1,63
ENSG00000005059	CCDC109B	3,74	1,50
ENSG00000236637	IFNA4	3,74	1,48
ENSG00000067533	RRP15	3,74	1,70
ENSG00000166483	WEE1	3,74	3,04
ENSG00000169020	ATP5I	3,74	1,36
ENSG00000136167	LCP1	3,74	3,44
ENSG00000187555	USP7	3,74	1,28
ENSG00000265241	RBM8A	3,74	1,61
ENSG00000089234	BRAP	3,73	1,46
ENSG00000166913	YWHAB	3,73	1,40
ENSG00000125970	RALY	3,73	1,56
ENSG00000213231	TCL1B	3,73	2,33

ensembl gene	gene symbol	Score(d)	Fold Change
ENSG00000174021	GNG5	3,73	1,98
ENSG00000102271	KLHL4	3,72	2,96
ENSG00000105254	TBCB	3,72	1,56
ENSG00000101084	C20orf24	3,72	1,67
ENSG00000182809	CRIP2	3,72	1,80
ENSG00000138794	CASP6	3,72	1,59
ENSG00000179826	MRGPRX3	3,72	1,33
ENSG00000080845	DLGAP4	3,72	1,62
ENSG00000160932	LY6E	3,71	2,64
ENSG00000127328	RAB3IP	3,71	1,55
ENSG00000165637	VDAC2	3,71	1,29
ENSG00000204264	PSMB8	3,70	1,94
ENSG00000146425	DYNLT1	3,70	1,71
ENSG00000162341	TPCN2	3,70	1,43
ENSG00000160799	CCDC12	3,70	1,49
ENSG00000237190	CDKN2AIPNL	3,70	1,48
ENSG00000204887	KRTAP1-4	3,70	1,41
ENSG00000131828	PDHA1	3,70	1,39
ENSG00000167720	SRR	3,70	1,67
ENSG00000135090	TAOK3	3,70	1,65
ENSG00000115091	ACTR3	3,69	1,31
ENSG00000167291	TBC1D16	3,69	1,48
ENSG00000119950	MXI1	3,69	1,65
ENSG00000122335	SERAC1	3,69	1,54
ENSG00000114520	SNX4	3,69	1,53
ENSG00000170340	B3GNT2	3,69	1,45
ENSG00000168003	SLC3A2	3,68	1,48
ENSG00000134313	KIDINS220	3,68	1,51
ENSG00000182208	MOB2	3,68	1,34
ENSG00000172893	DHCR7	3,68	1,51
ENSG00000100097	LGALS1	3,68	1,84
ENSG00000131711	MAP1B	3,68	4,06
ENSG00000197106	SLC6A17	3,68	1,37
ENSG00000266338	NBPF15	3,67	1,58
ENSG00000144635	DYNC1LI1	3,67	1,40
ENSG00000163590	PPM1L	3,67	1,85
ENSG00000155330	C16orf87	3,67	1,79
ENSG00000165644	COMTD1	3,67	1,38
ENSG00000100596	SPTLC2	3,67	1,70
ENSG00000006007	GDE1	3,67	1,37
ENSG00000272899	RP11-309L24.4	3,67	1,37
ENSG00000093000	NUP50	3,66	1,43
ENSG00000174749	C4orf32	3,66	1,61
ENSG00000197540	GZMM	3,66	1,23
ENSG00000221823	PPP3R1	3,66	1,34
ENSG00000143367	TUFT1	3,65	1,54
ENSG00000187608	ISG15	3,65	1,65
ENSG00000062650	WAPL	3,65	1,39
ENSG00000162909	CAPN2	3,64	1,83

ensembl gene	gene symbol	Score(d)	Fold Change
ENSG00000183741	CBX6	3,64	1,71
ENSG00000281899	C1QTNF9B-AS1	3,64	1,28
ENSG00000170385	SLC30A1	3,64	1,72
ENSG00000196470	SIAH1	3,63	1,79
ENSG00000157870	FAM213B	3,63	1,33
ENSG00000107771	CCSER2	3,63	1,43
ENSG00000013441	CLK1	3,63	1,53
ENSG00000185189	NRBP2	3,63	1,28
ENSG00000197125	OR8B8	3,62	1,29
ENSG00000165006	UBAP1	3,62	1,46
ENSG00000230124	ACBD6	3,62	1,52
ENSG00000118007	STAG1	3,62	1,33
ENSG00000119285	HEATR1	3,62	1,63
ENSG00000153165	RGPD3	3,62	1,88
ENSG00000168159	RNF187	3,61	1,61
ENSG00000130254	SAFB2	3,61	1,55
ENSG00000015479	MATR3	3,61	1,81
ENSG00000163904	SENP2	3,61	1,66
ENSG00000118242	MREG	3,61	1,78
ENSG00000240204	SMKR1	3,61	1,54
ENSG00000151287	TEX30	3,61	1,87
ENSG00000134698	AGO4	3,60	2,01
ENSG00000119673	ACOT2	3,60	2,23
ENSG00000224531	SMIM13	3,60	1,44
ENSG00000091436	AC013461.1	3,60	2,00
ENSG00000128283	CDC42EP1	3,60	1,33
ENSG00000112305	SMAP1	3,59	1,35
ENSG00000214367	HAUS3	3,59	2,01
ENSG00000151553	FAM160B1	3,59	1,69
ENSG00000160271	RALGDS	3,59	1,69
ENSG00000145365	TIFA	3,59	1,69
ENSG00000124089	MC3R	3,59	1,22
ENSG00000275152	CCL16	3,59	1,32
ENSG00000178473	UCN3	3,59	1,23
ENSG00000174840	PDE12	3,58	1,46
ENSG00000060971	ACAA1	3,58	1,39
ENSG00000185633	NDUFA4L2	3,58	1,45
ENSG00000143633	C1orf131	3,58	1,54
ENSG00000163444	TMEM183A	3,57	1,65
ENSG00000177733	HNRNPA0	3,57	1,38
ENSG00000152242	C18orf25	3,56	1,41
ENSG00000100345	MYH9	3,56	1,69
ENSG00000157368	IL34	3,56	1,33
ENSG00000117614	SYF2	3,55	1,43
ENSG00000142871	CYR61	3,55	1,33
ENSG00000156515	HK1	3,55	1,72
ENSG00000244509	APOBEC3C	3,55	1,78
ENSG00000181826	RELL1	3,55	1,89
ENSG00000240857	RDH14	3,55	1,31

ensembl gene	gene symbol	Score(d)	Fold Change
ENSG00000107593	PKD2L1	3,54	1,32
ENSG00000169860	P2RY1	3,54	1,25
ENSG00000067167	TRAM1	3,54	1,28
ENSG00000136710	CCDC115	3,54	1,24
ENSG00000124140	SLC12A5	3,54	1,45
ENSG00000134697	GNL2	3,54	1,48
ENSG00000182557	SPNS3	3,53	1,30
ENSG00000124134	KCNS1	3,53	1,20
ENSG00000103126	AXIN1	3,53	1,32
ENSG00000127334	DYRK2	3,53	1,80
ENSG00000010810	FYN	3,52	2,26
ENSG00000180488	FAM73A	3,52	1,43
ENSG00000153574	RPIA	3,52	1,43
ENSG00000083168	KAT6A	3,52	1,38
ENSG00000099942	CRKL	3,52	1,32
ENSG00000151914	DST	3,51	2,52
ENSG00000092330	TINF2	3,51	1,34
ENSG00000047849	MAP4	3,51	1,46
ENSG00000153310	FAM49B	3,51	1,54
ENSG00000104064	GABPB1	3,51	1,74
ENSG00000137776	SLTM	3,51	1,41
ENSG00000104447	TRPS1	3,51	2,69
ENSG00000109586	GALNT7	3,51	1,64
ENSG00000167618	LAIR2	3,50	1,58
ENSG00000155961	RAB39B	3,50	1,54
ENSG00000109846	CRYAB	3,50	1,21
ENSG00000118197	DDX59	3,50	1,60
ENSG00000171861	MRM3	3,50	1,48
ENSG00000197417	SHPK	3,50	1,64
ENSG00000160993	ALKBH4	3,50	1,24
ENSG00000145832	SLC25A48	3,49	1,42
ENSG00000145216	FIP1L1	3,49	1,34
ENSG00000170876	TMEM43	3,49	1,54
ENSG00000136930	PSMB7	3,49	1,37
ENSG00000100485	SOS2	3,49	1,84
ENSG00000103266	STUB1	3,49	1,47
ENSG00000144747	TMF1	3,49	1,46
ENSG00000184647	PRSS55	3,49	1,24
ENSG00000139697	SBNO1	3,48	1,39
ENSG00000135801	TAF5L	3,48	1,40
ENSG00000100591	AHSA1	3,48	1,56
ENSG00000160908	ZNF394	3,48	1,33
ENSG00000144426	NBEAL1	3,48	1,55
ENSG00000177684	DEFB114	3,48	1,31
ENSG00000100083	GGA1	3,48	1,31
ENSG00000145247	OCIAD2	3,47	1,46
ENSG00000137876	RSL24D1	3,47	1,49
ENSG00000140612	SEC11A	3,47	1,54
ENSG00000105507	CABP5	3,47	1,25

ensembl gene	gene symbol	Score(d)	Fold Change
ENSG00000168591	TMUB2	3,47	1,23
ENSG00000136634	IL10	3,47	1,86
ENSG00000106397	PLOD3	3,46	1,59
ENSG00000170185	USP38	3,46	1,39
ENSG00000197019	SERTAD1	3,46	1,46
ENSG00000049541	RFC2	3,46	1,52
ENSG00000104472	CHRAC1	3,46	1,50
ENSG00000165678	GHITM	3,46	1,30
ENSG00000100297	MCM5	3,46	1,58
ENSG00000092871	RFFL	3,46	1,51
ENSG00000159023	EPB41	3,46	1,30
ENSG00000188295	ZNF669	3,46	1,30
ENSG00000175203	DCTN2	3,45	1,43
ENSG00000002919	SNX11	3,45	1,33
ENSG00000072864	NDE1	3,45	1,51
ENSG00000160179	ABCG1	3,45	2,80
ENSG00000105251	SHD	3,45	1,40
ENSG00000166333	ILK	3,44	1,42
ENSG00000116752	BCAS2	3,44	1,36
ENSG00000180185	FAHD1	3,44	1,31
ENSG00000068903	SIRT2	3,44	1,53
ENSG00000154174	TOMM70	3,44	1,53
ENSG00000166012	TAF1D	3,44	1,61
ENSG00000105193	RPS16	3,44	1,99
ENSG00000174775	HRAS	3,43	1,22
ENSG00000139269	INHBE	3,43	2,84
ENSG00000104142	VPS18	3,43	1,27
ENSG00000102172	SMS	3,43	1,54
ENSG00000157045	NTAN1	3,43	1,84
ENSG00000111911	HINT3	3,42	1,36
ENSG00000101928	MOSPD1	3,42	1,50
ENSG00000213341	CHUK	3,42	1,53
ENSG00000214642	DEFB113	3,42	1,26
ENSG00000160326	SLC2A6	3,42	1,27
ENSG00000197951	ZNF71	3,42	1,38
ENSG00000181744	C3orf58	3,42	1,71
ENSG00000137055	PLAA	3,42	1,55
ENSG00000196466	ZNF799	3,42	1,64
ENSG00000123505	AMD1	3,42	1,55
ENSG00000188739	RBM34	3,41	1,98
ENSG00000128609	NDUFA5	3,41	1,36
ENSG00000115649	CNPPD1	3,41	1,54
ENSG00000198060	42799	3,41	1,58
ENSG00000204524	ZNF805	3,40	1,53
ENSG00000176920	FUT2	3,40	1,30
ENSG00000215301	DDX3X	3,40	1,37
ENSG00000126775	ATG14	3,40	1,63
ENSG00000178988	MRFAP1L1	3,40	1,37
ENSG00000123689	G0S2	3,40	2,12

ensembl gene	gene symbol	Score(d)	Fold Change
ENSG00000144713	RPL32	3,39	1,60
ENSG00000114166	KAT2B	3,39	1,42
ENSG00000119718	EIF2B2	3,39	1,46
ENSG00000119689	DLST	3,39	1,44
ENSG00000068079	IFI35	3,39	1,58
ENSG00000069493	CLEC2D	3,39	2,42
ENSG00000136960	ENPP2	3,39	2,90
ENSG00000221989	OR2A2	3,39	1,36
ENSG00000172175	MALT1	3,39	1,51
ENSG00000241563	CORT	3,39	1,33
ENSG00000227471	AKR1B15	3,39	1,77
ENSG00000151461	UPF2	3,38	1,49
ENSG00000114978	MOB1A	3,38	1,37
ENSG00000138685	FGF2	3,38	3,17
ENSG00000143167	GPA33	3,38	1,35
ENSG00000007944	MYLIP	3,38	1,90
ENSG00000162377	COA7	3,38	1,38
ENSG00000124228	DDX27	3,38	1,23
ENSG00000204388	HSPA1B	3,38	2,71
ENSG00000115762	PLEKHB2	3,38	1,50
ENSG00000108651	UTP6	3,37	1,44
ENSG00000121691	CAT	3,37	1,50
ENSG00000198271	KRTAP4-5	3,37	1,23
ENSG00000072609	CHFR	3,37	1,44
ENSG00000166780	C16orf45	3,37	1,37
ENSG00000242173	ARHGDIG	3,37	1,27
ENSG00000017797	RALBP1	3,37	1,46
ENSG00000198909	MAP3K3	3,37	1,32
ENSG00000142867	BCL10	3,36	1,51
ENSG00000198898	CAPZA2	3,36	1,41
ENSG00000023228	NDUFS1	3,35	1,32
ENSG00000155256	ZFYVE27	3,35	1,40
ENSG00000008324	SS18L2	3,35	1,41
ENSG00000069509	FUNDC1	3,35	1,36
ENSG00000131381	RBSN	3,35	1,38
ENSG00000182512	GLRX5	3,35	1,41
ENSG00000189030	VHLL	3,34	1,24
ENSG00000184945	AQP12A	3,34	1,23
ENSG00000253719	ATXN7L3B	3,34	1,32
ENSG00000149231	CCDC82	3,34	1,43
ENSG00000214819	CDRT15L2	3,34	1,52
ENSG00000179476	C14orf28	3,34	1,56
ENSG00000101132	PFDN4	3,33	1,39
ENSG00000181626	ANKRD62	3,33	1,18
ENSG00000177370	TIMM22	3,33	1,38
ENSG00000006451	RALA	3,33	1,41
ENSG00000001497	LAS1L	3,33	1,21
ENSG00000101972	STAG2	3,33	1,45
ENSG00000108671	PSMD11	3,33	1,26

ensembl gene	gene symbol	Score(d)	Fold Change
ENSG00000060762	MPC1	3,33	1,48
ENSG00000132259	CNGA4	3,33	1,24
ENSG00000130045	NXNL2	3,33	1,33
ENSG00000157954	WIPI2	3,33	1,54
ENSG00000067064	IDI1	3,33	1,31
ENSG00000143878	RHOB	3,33	1,94
ENSG00000082515	MRPL22	3,32	1,45
ENSG00000103044	HAS3	3,32	1,24
ENSG00000023892	DEF6	3,32	1,51
ENSG00000169762	TAPT1	3,32	1,41
ENSG00000159596	TMEM69	3,32	1,60
ENSG00000189164	ZNF527	3,32	1,87
ENSG00000153933	DGKE	3,32	1,45
ENSG00000102119	EMD	3,32	1,43
ENSG00000122085	MTERF4	3,31	1,27
ENSG00000169446	MMGT1	3,31	1,36
ENSG00000111445	RFC5	3,31	1,70
ENSG00000165512	ZNF22	3,31	1,29
ENSG00000110075	PPP6R3	3,31	1,28
ENSG00000196365	LONP1	3,31	1,42
ENSG00000136536	42801	3,31	1,43
ENSG00000067829	IDH3G	3,30	1,37
ENSG00000173013	CCDC96	3,30	1,19
ENSG00000168890	TMEM150A	3,30	1,37
ENSG00000109255	NMU	3,30	1,53
ENSG00000088986	DYNLL1	3,30	1,89
ENSG00000149792	MRPL49	3,30	1,39
ENSG00000101361	NOP56	3,30	1,37
ENSG00000115761	NOL10	3,30	1,40
ENSG00000184381	PLA2G6	3,30	1,39
ENSG00000102001	CACNA1F	3,30	1,49
ENSG00000082512	TRAF5	3,29	2,02
ENSG00000169914	OTUD3	3,29	1,50
ENSG00000177954	RPS27	3,29	1,50
ENSG00000116514	RNF19B	3,29	1,67
ENSG00000150676	CCDC83	3,29	1,30
ENSG00000182899	RPL35A	3,29	1,23
ENSG00000213741	RPS29	3,29	1,61
ENSG00000185250	PPIL6	3,29	1,77
ENSG00000120798	NR2C1	3,29	1,49
ENSG00000156860	FBRS	3,29	1,33
ENSG00000198390	KRTAP13-1	3,28	1,18
ENSG00000229676	ZNF492	3,28	1,23
ENSG00000149923	PPP4C	3,28	1,28
ENSG00000103274	NUBP1	3,28	1,41
ENSG00000159259	CHAF1B	3,28	1,94
ENSG00000164919	COX6C	3,28	1,45
ENSG00000175105	ZNF654	3,28	1,38
ENSG00000100532	CGRRF1	3,28	1,49

ensembl gene	gene symbol	Score(d)	Fold Change
ENSG00000240065	PSMB9	3,28	1,54
ENSG00000134109	EDEM1	3,27	1,51
ENSG00000166889	PATL1	3,27	1,40
ENSG00000069329	VPS35	3,27	1,47
ENSG00000149150	SLC43A1	3,27	1,81
ENSG00000162972	C2orf47	3,26	1,46
ENSG00000105379	ETFB	3,26	1,54
ENSG00000197323	TRIM33	3,26	1,51
ENSG00000110063	DCPS	3,26	1,45
ENSG00000198836	OPA1	3,26	1,44
ENSG00000022277	RTFDC1	3,26	1,34
ENSG00000107959	PITRM1	3,26	1,44
ENSG00000111142	METAP2	3,26	1,30
ENSG00000160310	PRMT2	3,26	1,43
ENSG00000179299	NSUN7	3,25	2,07
ENSG00000034152	MAP2K3	3,25	1,72
ENSG00000111371	SLC38A1	3,25	1,38
ENSG00000234224	TMEM229A	3,25	1,44
ENSG00000108349	CASC3	3,24	1,35
ENSG00000095951	HIVEP1	3,24	1,54
ENSG00000116747	TROVE2	3,24	1,56
ENSG00000173264	GPR137	3,24	1,27
ENSG00000108582	CPD	3,24	1,65
ENSG00000111450	STX2	3,24	1,68
ENSG00000070950	RAD18	3,24	1,51
ENSG00000249141	RP11-514O12.4	3,24	1,87
ENSG00000117620	SLC35A3	3,23	1,49
ENSG00000110324	IL10RA	3,23	2,70
ENSG00000112306	RPS12	3,23	1,89
ENSG00000135114	OASL	3,23	1,52
ENSG00000164849	GPR146	3,23	1,32
ENSG00000134602	STK26	3,23	1,43
ENSG00000106052	TAX1BP1	3,23	1,32
ENSG00000160185	UBASH3A	3,23	1,17
ENSG00000164729	SLC35G3	3,23	1,16
ENSG00000198453	ZNF568	3,23	1,48
ENSG00000273540	AGBL1	3,22	1,24
ENSG00000180228	PRKRA	3,22	1,37
ENSG00000170369	CST2	3,22	1,23
ENSG00000171492	LRRC8D	3,22	1,53
ENSG00000161203	AP2M1	3,22	1,45
ENSG00000150593	PDCD4	3,22	2,30
ENSG00000170903	MSANTD4	3,22	1,68
ENSG00000196224	KRTAP5-3	3,22	1,35
ENSG00000163599	CTLA4	3,22	1,64
ENSG00000100473	COCH	3,22	2,40
ENSG00000143947	RPS27A	3,21	1,87
ENSG00000188807	TMEM201	3,21	1,28
ENSG00000185825	BCAP31	3,21	1,29

ensembl gene	gene symbol	Score(d)	Fold Change
ENSG00000145901	TNIP1	3,21	1,71
ENSG00000087510	TFAP2C	3,21	1,34
ENSG00000072182	ASIC4	3,21	1,17
ENSG00000167106	FAM102A	3,21	2,04
ENSG00000177105	RHOG	3,21	1,37
ENSG00000197265	GTF2E2	3,20	1,43
ENSG00000196420	S100A5	3,20	1,22
ENSG00000162687	KCNT2	3,20	1,66
ENSG00000181264	TMEM136	3,20	1,71
ENSG00000109832	DDX25	3,20	1,24
ENSG00000008300	CELSR3	3,20	1,23
ENSG00000137574	TGS1	3,20	1,30
ENSG00000129187	DCTD	3,19	1,30
ENSG00000052802	MSMO1	3,19	1,65
ENSG00000151917	BEND6	3,19	1,60
ENSG00000105329	TGFB1	3,19	1,58
ENSG00000124783	SSR1	3,19	1,28
ENSG00000178860	MSC	3,19	1,68
ENSG00000138303	ASCC1	3,19	1,63
ENSG00000241258	CRCP	3,19	1,36
ENSG00000115241	PPM1G	3,18	1,27
ENSG00000125870	SNRPB2	3,18	1,32
ENSG00000212710	CTAGE1	3,17	1,26
ENSG00000047365	ARAP2	3,17	2,16
ENSG00000133055	MYBPH	3,17	1,14
ENSG00000147789	ZNF7	3,17	1,29
ENSG00000106025	TSPAN12	3,17	1,43
ENSG00000121022	COPS5	3,17	1,42
ENSG00000178950	GAK	3,17	1,33
ENSG00000152463	OLAH	3,17	2,20
ENSG00000136051	KIAA1033	3,17	1,38
ENSG00000149043	SYT8	3,16	1,43
ENSG00000260286	C6orf229	3,16	1,22
ENSG00000136504	KAT7	3,16	1,38
ENSG00000175305	CCNE2	3,16	2,22
ENSG00000106028	SSBP1	3,16	1,38
ENSG00000188707	ZBED6CL	3,16	1,26
ENSG00000100612	DHRS7	3,16	1,48
ENSG00000034053	APBA2	3,16	2,03
ENSG00000182372	CLN8	3,15	1,69
ENSG00000175550	DRAP1	3,15	1,56
ENSG00000133321	RARRES3	3,15	2,13
ENSG00000135655	USP15	3,15	1,34
ENSG00000168438	CDC40	3,15	1,39
ENSG00000037474	NSUN2	3,15	1,50
ENSG00000240230	COX19	3,15	1,21
ENSG00000187180	LCE2C	3,15	1,30
ENSG00000170619	COMMD5	3,15	1,24
ENSG00000114062	UBE3A	3,15	1,28

ensembl gene	gene symbol	Score(d)	Fold Change
ENSG00000130881	LRP3	3,15	1,27
ENSG00000141668	CBLN2	3,14	1,33
ENSG00000071537	SEL1L	3,14	1,49
ENSG00000103671	TRIP4	3,14	1,29
ENSG00000169032	MAP2K1	3,14	1,47
ENSG00000214694	ARHGEF33	3,14	1,36
ENSG00000144895	EIF2A	3,14	1,39
ENSG00000173692	PSMD1	3,14	1,25
ENSG00000196378	ZNF34	3,14	1,30
ENSG00000130598	TNNI2	3,14	1,23
ENSG00000242259	C22orf39	3,14	1,27
ENSG00000165269	AQP7	3,13	1,33
ENSG00000172059	KLF11	3,13	1,92
ENSG00000014824	SLC30A9	3,13	1,38
ENSG00000095917	TPSD1	3,13	1,22
ENSG00000138802	SEC24B	3,13	1,34
ENSG00000107745	MICU1	3,13	1,38
ENSG00000182187	CRYGB	3,13	1,25
ENSG00000183569	SERHL2	3,13	1,50
ENSG00000019995	ZRANB1	3,12	1,45
ENSG00000100425	BRD1	3,12	1,33
ENSG00000160948	VPS28	3,12	1,29
ENSG00000143155	TIPRL	3,12	1,41
ENSG00000171503	ETFDH	3,12	1,47
ENSG00000167595	PROSER3	3,12	1,22
ENSG00000161031	PGLYRP2	3,12	1,30
ENSG00000105851	PIK3CG	3,12	1,79
ENSG00000135317	SNX14	3,12	1,27
ENSG00000100206	DMC1	3,11	1,37
ENSG00000100982	PCIF1	3,11	1,39
ENSG00000138182	KIF20B	3,11	1,71
ENSG00000132432	SEC61G	3,11	1,47
ENSG00000122359	ANXA11	3,11	1,49
ENSG00000147687	TATDN1	3,11	1,63
ENSG00000072786	STK10	3,10	1,39
ENSG00000154781	CCDC174	3,10	1,31
ENSG00000120254	MTHFD1L	3,10	1,70
ENSG00000077458	FAM76B	3,10	1,74
ENSG00000165194	PCDH19	3,10	1,42
ENSG00000156875	MFSD14A	3,10	1,65
ENSG00000129744	ART1	3,10	1,42
ENSG00000057757	PITHD1	3,10	1,31
ENSG00000122958	VPS26A	3,10	1,32
ENSG00000142784	WDTC1	3,10	1,31
ENSG00000078549	ADCYAP1R1	3,10	1,14
ENSG00000131437	KIF3A	3,10	1,62
ENSG00000011021	CLCN6	3,10	1,61
ENSG00000168795	ZBTB5	3,09	1,37
ENSG00000148481	FAM188A	3,09	1,37

ensembl gene	gene symbol	Score(d)	Fold Change
ENSG00000130827	PLXNA3	3,09	1,66
ENSG00000106524	ANKMY2	3,09	1,38
ENSG00000093183	SEC22C	3,09	1,34
ENSG00000115484	CCT4	3,09	1,40
ENSG00000198034	RPS4X	3,09	1,29
ENSG00000170677	SOCS6	3,09	1,54
ENSG00000137073	UBAP2	3,08	1,28
ENSG00000132514	CLEC10A	3,08	1,31
ENSG00000124209	RAB22A	3,08	1,43
ENSG00000102054	RBBP7	3,08	1,27
ENSG00000187672	ERC2	3,07	1,79
ENSG00000080709	KCNN2	3,07	1,35
ENSG00000135241	PNPLA8	3,07	1,64
ENSG00000100393	EP300	3,07	1,41
ENSG00000206073	SERPINB4	3,07	1,27
ENSG00000132821	VSTM2L	3,07	1,22
ENSG00000124596	OARD1	3,07	1,24
ENSG00000198695	MT-ND6	3,07	1,58
ENSG00000081154	PCNP	3,07	1,29
ENSG00000163605	PPP4R2	3,07	1,23
ENSG00000185294	SPPL2C	3,06	1,13
ENSG00000109111	SUPT6H	3,06	1,35
ENSG00000213977	TAX1BP3	3,06	1,60
ENSG00000179636	TPPP2	3,06	1,28
ENSG00000088930	XRN2	3,06	1,32
ENSG00000143093	STRIP1	3,06	1,40
ENSG00000187840	EIF4EBP1	3,06	1,89
ENSG00000163412	EIF4E3	3,06	1,39
ENSG00000169994	MYO7B	3,06	1,15
ENSG00000175874	CREG2	3,06	1,40
ENSG00000115267	IFIH1	3,05	1,76
ENSG00000159692	CTBP1	3,05	1,37
ENSG00000105617	LENG1	3,05	1,21
ENSG00000103160	HSDL1	3,05	1,71
ENSG00000198171	DDRGK1	3,05	1,39
ENSG00000115129	TP53I3	3,05	1,40
ENSG00000114480	GBE1	3,05	1,57
ENSG00000106105	GARS	3,05	1,52
ENSG00000100258	LMF2	3,05	1,34
ENSG00000176281	OR4K5	3,05	1,17
ENSG00000062582	MRPS24	3,05	1,52
ENSG00000151247	EIF4E	3,04	1,44
ENSG00000173621	LRFN4	3,04	1,21
ENSG00000140694	PARN	3,04	1,30
ENSG00000127870	RNF6	3,04	1,44
ENSG00000110944	IL23A	3,04	1,57
ENSG00000213822	CEACAM18	3,04	1,16
ENSG00000113013	HSPA9	3,04	1,31
ENSG00000134996	OSTF1	3,04	1,52

ensembl gene	gene symbol	Score(d)	Fold Change
ENSG00000109323	MANBA	3,04	1,33
ENSG00000176410	DNAJC30	3,04	1,26
ENSG00000163378	EOGT	3,03	1,44
ENSG00000112208	BAG2	3,03	1,56
ENSG00000101144	BMP7	3,03	1,37
ENSG00000158813	EDA	3,03	1,63
ENSG00000104331	IMPAD1	3,03	1,30
ENSG00000108239	TBC1D12	3,02	1,43
ENSG00000065911	MTHFD2	3,02	1,57
ENSG00000196437	ZNF569	3,02	1,80
ENSG00000111011	RSRC2	3,02	1,31
ENSG00000214706	IFRD2	3,02	1,34
ENSG00000135316	SYNCRIP	3,02	1,21
ENSG00000166295	ANAPC16	3,01	1,28
ENSG00000108061	SHOC2	3,01	1,32
ENSG00000174718	KIAA1551	3,01	1,58
ENSG00000196767	POU3F4	3,01	1,17
ENSG00000009954	BAZ1B	3,01	1,24
ENSG00000011590	ZBTB32	3,01	1,36
ENSG00000178826	TMEM139	3,01	1,40
ENSG00000095574	IKZF5	3,00	1,42
ENSG00000081307	UBA5	3,00	1,35
ENSG00000110243	APOA5	3,00	1,15
ENSG00000106605	BLVRA	3,00	1,35
ENSG00000166200	COPS2	3,00	1,38
ENSG00000164104	HMGB2	3,00	1,54
ENSG00000101197	BIRC7	3,00	1,23
ENSG00000165916	PSMC3	3,00	1,34
ENSG00000162885	B3GALNT2	3,00	1,47
ENSG00000075945	KIFAP3	3,00	1,49
ENSG00000169217	CD2BP2	2,99	1,29
ENSG00000157119	KLHL40	2,99	1,19
ENSG00000130707	ASS1	2,99	2,51
ENSG00000006062	MAP3K14	2,99	1,60
ENSG00000176783	RUFY1	2,99	1,40
ENSG00000127951	FGL2	2,99	1,87
ENSG00000114120	SLC25A36	2,99	1,47
ENSG00000116863	ADPRHL2	2,99	1,25
ENSG00000181499	OR6T1	2,99	1,27
ENSG00000168303	MPLKIP	2,99	1,29
ENSG00000166949	SMAD3	2,99	1,61
ENSG00000132017	DCAF15	2,99	1,30
ENSG00000164287	CDC20B	2,99	1,18
ENSG00000130023	ERMARD	2,98	1,43
ENSG00000196361	ELAVL3	2,98	1,37
ENSG00000110442	COMMD9	2,98	1,30
ENSG00000152936	LMNTD1	2,98	1,32
ENSG00000169379	ARL13B	2,98	1,40
ENSG00000100522	GNPNAT1	2,98	1,64

ensembl gene	gene symbol	Score(d)	Fold Change
ENSG00000134962	KLB	2,98	1,16
ENSG00000103351	CLUAP1	2,98	1,50
ENSG00000128699	ORMDL1	2,98	1,32
ENSG00000056972	TRAF3IP2	2,98	1,49
ENSG00000175868	CALCB	2,98	1,75
ENSG00000186222	BLOC1S4	2,97	1,24
ENSG00000157502	MUM1L1	2,97	1,22
ENSG00000181804	SLC9A9	2,97	1,84
ENSG00000160161	CILP2	2,97	1,26
ENSG00000126460	PRRG2	2,97	1,16
ENSG00000139620	KANSL2	2,97	1,32
ENSG00000180233	ZNRF2	2,97	1,31
ENSG00000134597	RBMX2	2,97	1,33
ENSG00000114316	USP4	2,97	1,21
ENSG00000179873	NLRP11	2,97	1,64
ENSG00000171858	RPS21	2,96	1,65
ENSG00000187994	RINL	2,96	1,23
ENSG00000159961	OR3A3	2,96	1,27
ENSG00000105053	VRK3	2,96	1,44
ENSG00000205186	FABP9	2,96	1,16
ENSG00000147852	VLDLR	2,96	1,63
ENSG00000174004	NRROS	2,96	1,66
ENSG00000115183	TANC1	2,96	1,89
ENSG00000204595	DPRX	2,96	1,18
ENSG00000137713	PPP2R1B	2,96	1,41
ENSG00000130779	CLIP1	2,96	1,51
ENSG00000102078	SLC25A14	2,96	1,37
ENSG00000147224	PRPS1	2,96	1,29
ENSG00000165521	EML5	2,96	1,65
ENSG00000104885	DOT1L	2,96	1,32
ENSG00000156482	RPL30	2,96	1,59
ENSG00000065809	FAM107B	2,96	2,52
ENSG00000170310	STX8	2,95	1,32
ENSG00000113407	TARS	2,95	1,44
ENSG00000198521	ZNF43	2,95	1,78
ENSG00000236279	CLEC2L	2,95	1,29
ENSG00000132463	GRSF1	2,95	1,30
ENSG00000101343	CRNKL1	2,95	1,29
ENSG00000089225	TBX5	2,95	1,16
ENSG00000066557	LRRC40	2,94	1,46
ENSG00000026025	VIM	2,94	1,37
ENSG00000099960	SLC7A4	2,94	1,17
ENSG00000162923	WDR26	2,94	1,58
ENSG00000174780	SRP72	2,94	1,22
ENSG00000094841	UPRT	2,94	1,27
ENSG00000023287	RB1CC1	2,94	1,56
ENSG00000180776	ZDHHC20	2,94	1,52
ENSG00000159648	TEPP	2,94	1,24
ENSG00000147724	FAM135B	2,94	1,38

ensembl gene	gene symbol	Score(d)	Fold Change
ENSG00000168209	DDIT4	2,94	1,92
ENSG00000179094	PER1	2,94	1,92
ENSG00000078808	SDF4	2,93	1,32
ENSG00000185359	HGS	2,93	1,34
ENSG00000222047	C10orf55	2,93	1,19
ENSG00000121905	HPCA	2,93	1,32
ENSG00000164120	HPGD	2,93	1,44
ENSG00000204899	MZT1	2,93	1,37
ENSG00000133401	PDZD2	2,93	1,48
ENSG00000152133	GPATCH11	2,93	1,36
ENSG00000129515	SNX6	2,93	1,37
ENSG00000041802	LSG1	2,93	1,30
ENSG00000136463	TACO1	2,93	1,27
ENSG00000167384	ZNF180	2,93	1,36
ENSG00000077264	PAK3	2,93	1,32
ENSG00000120458	MSANTD2	2,92	1,32
ENSG00000171368	TPPP	2,92	1,28
ENSG00000185721	DRG1	2,92	1,40
ENSG00000169155	ZBTB43	2,92	1,36
ENSG00000115839	RAB3GAP1	2,92	1,27
ENSG00000268223	ARL14EPL	2,92	1,20
ENSG00000117625	RCOR3	2,92	1,45
ENSG00000182329	KIAA2012	2,92	1,44
ENSG00000170893	TRH	2,92	1,18
ENSG00000131725	WDR44	2,92	1,48
ENSG00000111885	MAN1A1	2,91	1,47
ENSG00000164941	INTS8	2,91	1,25
ENSG00000188730	VWC2	2,91	1,38
ENSG00000017260	ATP2C1	2,91	1,37
ENSG00000129292	PHF20L1	2,91	1,24
ENSG00000164038	SLC9B2	2,91	1,50
ENSG00000100478	AP4S1	2,91	1,47
ENSG00000102081	FMR1	2,91	1,50
ENSG00000065883	CDK13	2,91	1,31
ENSG00000106336	FBXO24	2,91	1,24
ENSG00000033030	ZCCHC8	2,90	1,40
ENSG00000133256	PDE6B	2,90	1,22
ENSG00000092439	TRPM7	2,90	1,31
ENSG00000167536	DHRS13	2,90	1,46
ENSG00000198839	ZNF277	2,90	1,33
ENSG00000112218	GPR63	2,90	2,27
ENSG00000214860	EVPLL	2,90	1,30
ENSG00000134028	ADAMDEC1	2,90	1,44
ENSG00000143702	CEP170	2,90	1,41
ENSG00000087274	ADD1	2,90	1,31
ENSG00000124659	TBCC	2,90	1,29
ENSG00000122884	P4HA1	2,89	1,95
ENSG00000179387	ELMOD2	2,89	1,34
ENSG00000158411	MITD1	2,89	1,36

ensembl gene	gene symbol	Score(d)	Fold Change
ENSG00000153975	ZUFSP	2,89	1,85
ENSG00000135052	GOLM1	2,89	2,13
ENSG00000105642	KCNN1	2,89	1,28
ENSG00000109424	UCP1	2,89	1,15
ENSG00000102218	RP2	2,89	1,47
ENSG00000204308	RNF5	2,89	1,28
ENSG00000181472	ZBTB2	2,89	1,47
ENSG00000213799	ZNF845	2,89	1,39
ENSG00000105258	POLR2I	2,88	1,31
ENSG00000140299	BNIP2	2,88	1,28
ENSG00000102317	RBM3	2,88	1,26
ENSG00000127241	MASP1	2,88	1,36
ENSG00000099949	LZTR1	2,88	1,24
ENSG00000085449	WDFY1	2,88	1,47
ENSG00000170191	NANP	2,88	1,25
ENSG00000204160	ZDHHC18	2,88	1,46
ENSG00000170653	ATF7	2,88	1,53
ENSG00000101546	RBFA	2,88	1,43
ENSG00000197860	SGTB	2,88	1,80
ENSG00000141429	GALNT1	2,88	1,56
ENSG00000068120	COASY	2,88	1,28
ENSG00000026103	FAS	2,88	2,14
ENSG00000137825	ITPKA	2,87	1,18
ENSG00000065665	SEC61A2	2,87	1,63
ENSG00000134668	SPOCD1	2,87	1,16
ENSG00000173418	NAA20	2,87	1,31
ENSG00000239605	C2orf61	2,87	1,24
ENSG00000171931	FBXW10	2,87	1,23
ENSG00000070193	FGF10	2,87	1,20
ENSG00000108523	RNF167	2,87	1,29
ENSG00000134317	GRHL1	2,87	1,39
ENSG00000136697	IL1F10	2,87	1,17
ENSG00000136738	STAM	2,87	1,43
ENSG00000172073	TEX37	2,87	1,16
ENSG00000137500	CCDC90B	2,86	1,28
ENSG00000178965	ERICH3	2,86	1,33
ENSG00000176171	BNIP3	2,86	2,38
ENSG00000154529	CNTNAP3B	2,86	1,27
ENSG00000153113	CAST	2,86	1,27
ENSG00000104714	ERICH1	2,86	1,41
ENSG00000117500	TMED5	2,86	1,30
ENSG00000163218	PGLYRP4	2,86	1,34
ENSG00000108828	VAT1	2,86	1,39
ENSG00000122042	UBL3	2,86	1,62
ENSG00000145975	FAM217A	2,86	1,27
ENSG00000141391	PRELID3A	2,85	1,19
ENSG00000139160	ETFBKMT	2,85	1,50
ENSG00000115944	COX7A2L	2,85	1,42
ENSG00000151726	ACSL1	2,85	1,83

ensembl gene	gene symbol	Score(d)	Fold Change
ENSG00000164128	NPY1R	2,85	1,34
ENSG00000151778	SERP2	2,85	1,34
ENSG00000186141	POLR3C	2,85	1,35
ENSG00000164076	CAMKV	2,85	1,16
ENSG00000221963	APOL6	2,85	1,48
ENSG00000128165	ADM2	2,85	1,28
ENSG00000204315	FKBPL	2,85	1,19
ENSG00000170486	KRT72	2,85	1,29
ENSG00000091583	APOH	2,84	1,79
ENSG00000132530	XAF1	2,84	1,98
ENSG00000100304	TTLL12	2,84	1,31
ENSG00000168061	SAC3D1	2,84	1,19
ENSG00000186868	MAPT	2,84	1,18
ENSG00000172809	RPL38	2,84	1,39
ENSG00000107937	GTPBP4	2,84	1,37
ENSG00000108094	CUL2	2,84	1,25
ENSG00000163320	CGGBP1	2,84	1,20
ENSG00000147432	CHRNB3	2,84	1,20
ENSG00000100220	RTCB	2,84	1,71
ENSG00000165219	GAPVD1	2,83	1,24
ENSG00000152270	PDE3B	2,83	2,63
ENSG00000175073	VCPIP1	2,83	1,29
ENSG00000116704	SLC35D1	2,83	1,41
ENSG00000106089	STX1A	2,83	1,37
ENSG00000107105	ELAVL2	2,83	1,33
ENSG00000139044	B4GALNT3	2,83	1,25
ENSG00000265763	ZNF488	2,83	1,22
ENSG00000157106	SMG1	2,83	1,35
ENSG00000222014	RAB6C	2,82	1,25
ENSG00000103023	PRSS54	2,82	1,16
ENSG00000123684	LPGAT1	2,82	1,59
ENSG00000100325	ASCC2	2,82	1,28
ENSG00000062485	CS	2,82	1,27
ENSG00000158786	PLA2G2F	2,82	1,19
ENSG00000173960	UBXN2A	2,82	1,36
ENSG00000105668	UPK1A	2,82	1,15
ENSG00000080189	SLC35C2	2,82	1,24
ENSG00000004478	FKBP4	2,82	1,24
ENSG00000055332	EIF2AK2	2,82	1,50
ENSG00000101782	RIOK3	2,82	1,45
ENSG00000058272	PPP1R12A	2,82	1,41
ENSG00000165684	SNAPC4	2,82	1,27
ENSG00000122299	ZC3H7A	2,81	1,36
ENSG00000257727	CNPY2	2,81	1,38
ENSG00000104825	NFKBIB	2,81	1,32
ENSG00000112110	MRPL18	2,81	1,37
ENSG00000105619	TFPT	2,81	1,30
ENSG00000172687	ZNF738	2,81	1,54
ENSG00000240694	PNMA2	2,81	1,20

ensembl gene	gene symbol	Score(d)	Fold Change
ENSG00000162654	GBP4	2,81	2,47
ENSG00000089220	PEBP1	2,81	1,62
ENSG00000166888	STAT6	2,80	1,39
ENSG00000140067	FAM181A	2,80	1,26
ENSG00000108064	TFAM	2,80	1,27
ENSG00000176946	THAP4	2,80	1,33
ENSG00000173218	VANGL1	2,80	1,52
ENSG00000110696	C11orf58	2,80	1,24
ENSG00000115963	RND3	2,80	3,17
ENSG00000141522	ARHGDIA	2,80	1,24
ENSG00000179059	ZFP42	2,80	1,23
ENSG00000104689	TNFRSF10A	2,79	1,46
ENSG00000155511	GRIA1	2,79	1,78
ENSG00000090273	NUDC	2,79	1,28
ENSG00000166984	TCP10L2	2,79	1,19
ENSG00000178226	PRSS36	2,79	1,31
ENSG00000240021	TEX35	2,79	1,55
ENSG00000101109	STK4	2,79	1,32
ENSG00000269556	TMEM185A	2,79	1,34
ENSG00000198848	CES1	2,79	1,27
ENSG00000117868	ESYT2	2,78	1,33
ENSG00000138594	TMOD3	2,78	1,25
ENSG00000185347	C14orf80	2,78	1,24
ENSG00000151465	CDC123	2,78	1,25
ENSG00000187189	TSPYL4	2,78	1,33
ENSG00000124103	FAM209A	2,78	1,48
ENSG00000198746	GPATCH3	2,78	1,18
ENSG00000160211	G6PD	2,78	1,35
ENSG00000169756	LIMS1	2,78	1,35
ENSG00000116761	CTH	2,78	2,06
ENSG00000143674	RP5-862P8.2	2,78	1,59
ENSG00000082438	COBLL1	2,78	1,63
ENSG00000175520	UBQLN3	2,78	1,34
ENSG00000170322	NFRKB	2,78	1,34
ENSG00000142327	RNPEPL1	2,78	1,37
ENSG00000170477	KRT4	2,78	1,22
ENSG00000068354	TBC1D25	2,78	1,25
ENSG00000101161	PRPF6	2,77	1,19
ENSG00000169410	PTPN9	2,77	1,28
ENSG00000196498	NCOR2	2,77	1,35
ENSG00000173349	SFT2D3	2,77	1,20
ENSG00000189186	DCAF8L2	2,77	1,34
ENSG00000215274	GAGE10	2,77	1,24
ENSG00000197386	HTT	2,77	1,34
ENSG00000122490	PQLC1	2,77	1,37
ENSG00000169504	CLIC4	2,77	1,39
ENSG00000198455	ZXDB	2,77	1,43
ENSG00000186407	CD300E	2,77	1,29
ENSG00000177551	NHLH2	2,77	1,16

ensembl gene	gene symbol	Score(d)	Fold Change
ENSG00000136628	EPRS	2,77	1,31
ENSG00000186314	PRELID2	2,77	1,47
ENSG00000198625	MDM4	2,77	1,33
ENSG00000162434	JAK1	2,77	1,36
ENSG00000144655	CSRNP1	2,76	1,57
ENSG00000075785	RAB7A	2,76	1,16
ENSG00000155313	USP25	2,76	1,50
ENSG00000105700	KXD1	2,76	1,23
ENSG00000153914	SREK1	2,76	1,22
ENSG00000144381	HSPD1	2,76	1,33
ENSG00000134247	PTGFRN	2,76	1,59
ENSG00000186335	SLC36A2	2,76	1,24
ENSG00000141552	ANAPC11	2,76	1,28
ENSG00000179674	ARL14	2,76	1,18
ENSG00000183379	SYNDIG1L	2,76	1,18
ENSG00000186930	KRTAP6-2	2,75	1,19
ENSG00000108219	TSPAN14	2,75	1,55
ENSG00000060982	BCAT1	2,75	1,96
ENSG00000118503	TNFAIP3	2,75	1,89
ENSG00000107949	BCCIP	2,75	1,39
ENSG00000118513	MYB	2,75	2,20
ENSG00000165943	MOAP1	2,75	1,23
ENSG00000103257	SLC7A5	2,75	1,80
ENSG00000106049	HIBADH	2,75	1,31
ENSG00000081721	DUSP12	2,75	1,41
ENSG00000138750	NUP54	2,75	1,31
ENSG00000183696	UPP1	2,74	1,39
ENSG00000087053	MTMR2	2,74	1,39
ENSG00000165516	KLHDC2	2,74	1,37
ENSG00000243056	EIF4EBP3	2,74	1,52
ENSG00000234545	FAM133B	2,74	1,37
ENSG00000124787	RPP40	2,74	1,80
ENSG00000166135	HIF1AN	2,74	1,28
ENSG00000111877	MCM9	2,74	1,35
ENSG00000147180	ZNF711	2,74	1,85
ENSG00000085511	MAP3K4	2,74	1,36
ENSG00000196900	TEX43	2,74	1,19
ENSG00000205442	IZUMO3	2,74	1,16
ENSG00000197343	ZNF655	2,74	1,39
ENSG00000162545	CAMK2N1	2,73	1,19
ENSG00000198768	APCDD1L	2,73	1,30
ENSG00000066455	GOLGA5	2,73	1,39
ENSG00000147381	MAGEA4	2,73	1,30
ENSG00000124564	SLC17A3	2,73	1,16
ENSG00000144118	RALB	2,73	1,25
ENSG00000244165	P2RY11	2,73	1,50
ENSG00000130813	C19orf66	2,73	1,29
ENSG00000111237	VPS29	2,73	1,27
ENSG00000168476	REEP4	2,73	1,36

ensembl gene	gene symbol	Score(d)	Fold Change
ENSG00000089041	P2RX7	2,73	2,16
ENSG00000162129	CLPB	2,73	1,28
ENSG00000118156	ZNF541	2,72	1,15
ENSG00000225526	MKRN2OS	2,72	1,16
ENSG00000102401	ARMCX3	2,72	1,27
ENSG00000147601	TERF1	2,72	1,38
ENSG00000141378	PTRH2	2,72	1,36
ENSG00000168612	ZSWIM1	2,72	1,27
ENSG00000239474	KLHL41	2,72	1,39
ENSG00000176435	CLEC14A	2,72	1,22
ENSG00000167461	RAB8A	2,72	1,33
ENSG00000180209	MYLPF	2,72	1,16
ENSG00000181852	RNF41	2,72	1,31
ENSG00000112651	MRPL2	2,72	1,33
ENSG00000215375	MYL5	2,72	1,21
ENSG00000183828	NUDT14	2,72	1,23
ENSG00000137818	RPLP1	2,72	1,42
ENSG00000100483	VCPKMT	2,71	1,46
ENSG00000060491	OGFR	2,71	1,35
ENSG00000042429	MED17	2,71	1,31
ENSG00000154451	GBP5	2,71	2,20
ENSG00000188488	SERPINA5	2,71	1,21
ENSG00000071242	RPS6KA2	2,71	1,86
ENSG00000145337	PYURF	2,71	1,30
ENSG00000163918	RFC4	2,71	1,49
ENSG00000116688	MFN2	2,71	1,23
ENSG00000196507	TCEAL3	2,71	1,13
ENSG00000234127	TRIM26	2,71	1,26
ENSG00000163344	PMVK	2,71	1,46
ENSG00000066654	THUMPD1	2,70	1,30
ENSG00000165917	RAPSN	2,70	1,18
ENSG00000138018	EPT1	2,70	1,25
ENSG00000092820	EZR	2,70	1,36
ENSG00000139233	LLPH	2,70	1,24
ENSG00000019169	MARCO	2,70	1,27
ENSG00000179918	SEPHS2	2,70	1,30
ENSG00000100335	MIEF1	2,70	1,34
ENSG00000172594	SMPDL3A	2,70	1,62
ENSG00000186838	SELV	2,70	1,13
ENSG00000060566	CREB3L3	2,70	1,12
ENSG00000107438	PDLIM1	2,70	1,53
ENSG00000032219	ARID4A	2,70	1,82
ENSG00000086619	ERO1B	2,70	1,45
ENSG00000165525	NEMF	2,70	1,30
ENSG00000121766	ZCCHC17	2,69	1,28
ENSG00000124216	SNAI1	2,69	1,31
ENSG00000151552	QDPR	2,69	1,31
ENSG00000112249	ASCC3	2,69	1,46
ENSG00000164828	SUN1	2,69	1,36

ensembl gene	gene symbol	Score(d)	Fold Change
ENSG00000161896	IP6K3	2,69	1,26
ENSG00000070718	AP3M2	2,69	1,39
ENSG00000096384	HSP90AB1	2,69	1,39
ENSG00000135521	LTV1	2,69	1,44
ENSG00000140105	WARS	2,69	1,63
ENSG00000141437	SLC25A52	2,69	1,20
ENSG00000109536	FRG1	2,69	1,47
ENSG00000170967	DDI1	2,69	1,16
ENSG00000110619	CARS	2,69	1,40
ENSG00000139973	SYT16	2,69	1,18
ENSG00000179632	MAF1	2,69	1,31
ENSG00000112715	VEGFA	2,69	1,58
ENSG00000140511	HAPLN3	2,69	1,60
ENSG00000096717	SIRT1	2,69	1,34
ENSG00000109332	UBE2D3	2,69	1,29
ENSG00000180353	HCLS1	2,69	1,31
ENSG00000149488	TMC2	2,69	1,19
ENSG00000119820	YIPF4	2,68	1,34
ENSG00000228607	CLDN25	2,68	1,17
ENSG00000173769	TOPAZ1	2,68	1,10
ENSG00000074211	PPP2R2C	2,68	1,20
ENSG00000172939	OXSR1	2,68	1,31
ENSG00000178586	OR6B3	2,68	1,20
ENSG00000184394	OR4N5	2,68	1,20
ENSG00000168259	DNAJC7	2,68	1,26
ENSG00000106299	WASL	2,68	1,36
ENSG00000147874	HAUS6	2,68	1,32
ENSG00000189046	ALKBH2	2,68	1,40
ENSG00000100426	ZBED4	2,68	1,20
ENSG00000153767	GTF2E1	2,68	1,33
ENSG00000116586	LAMTOR2	2,68	1,29
ENSG00000141873	SLC39A3	2,68	1,72
ENSG00000178425	NT5DC1	2,68	1,59
ENSG00000000938	FGR	2,68	1,28
ENSG00000119048	UBE2B	2,68	1,44
ENSG00000129473	BCL2L2	2,68	1,42
ENSG00000113810	SMC4	2,68	1,46
ENSG00000141027	NCOR1	2,68	1,25
ENSG00000161960	EIF4A1	2,68	1,66
ENSG00000204370	SDHD	2,67	1,50
ENSG00000173557	C2orf70	2,67	1,17
ENSG00000249751	ECSCR	2,67	1,17
ENSG00000104879	CKM	2,67	1,17
ENSG00000203950	FAM127B	2,67	1,34
ENSG00000103549	RNF40	2,66	1,24
ENSG00000101654	RNMT	2,66	1,56
ENSG00000182973	CNOT10	2,66	1,43
ENSG00000107938	EDRF1	2,66	1,31
ENSG00000043093	DCUN1D1	2,66	1,33

ensembl gene	gene symbol	Score(d)	Fold Change
ENSG00000127399	LRRC61	2,66	1,22
ENSG00000130640	TUBGCP2	2,66	1,17
ENSG00000109046	WSB1	2,66	1,38
ENSG00000104805	NUCB1	2,66	1,31
ENSG00000054267	ARID4B	2,66	1,44
ENSG00000170689	HOXB9	2,66	1,23
ENSG00000188725	SMIM15	2,66	1,22
ENSG00000134440	NARS	2,66	1,28
ENSG00000111647	UHRF1BP1L	2,66	1,41
ENSG00000204161	C10orf128	2,65	1,39
ENSG00000176624	MEX3C	2,65	1,37
ENSG00000125484	GTF3C4	2,65	1,30
ENSG00000233412	OR5H15	2,65	1,19
ENSG00000142089	IFITM3	2,65	1,85
ENSG00000090013	BLVRB	2,65	1,45
ENSG00000011523	CEP68	2,65	1,43
ENSG00000065613	SLK	2,65	1,46
ENSG00000235942	LCE6A	2,65	1,11
ENSG00000166441	RPL27A	2,65	1,39
ENSG00000110700	RPS13	2,65	1,32
ENSG00000156802	ATAD2	2,65	1,74
ENSG00000139668	WDFY2	2,65	1,29
ENSG00000125249	RAP2A	2,65	1,63
ENSG00000092148	HECTD1	2,65	1,27
ENSG00000090432	MUL1	2,65	1,32
ENSG00000144736	SHQ1	2,65	1,38
ENSG00000161011	SQSTM1	2,64	1,32
ENSG00000100442	FKBP3	2,64	1,49
ENSG00000105699	LSR	2,64	1,31
ENSG00000102032	RENBP	2,64	1,16
ENSG00000170540	ARL6IP1	2,64	1,37
ENSG00000122971	ACADS	2,64	1,16
ENSG00000072506	HSD17B10	2,64	1,33
ENSG00000186509	OR9Q1	2,64	1,21
ENSG00000139278	GLIPR1	2,64	1,96
ENSG00000258713	C20orf141	2,64	1,26
ENSG00000002822	MAD1L1	2,64	1,27
ENSG00000126067	PSMB2	2,64	1,23
ENSG00000138069	RAB1A	2,64	1,22
ENSG00000168672	FAM84B	2,64	2,77
ENSG00000172500	FIBP	2,64	1,32
ENSG00000205857	NANOGNB	2,64	1,18
ENSG00000006659	LGALS14	2,63	3,06
ENSG00000116350	SRSF4	2,63	1,24
ENSG00000164089	ETNPPL	2,63	1,12
ENSG00000122376	FAM35A	2,63	1,35
ENSG00000126903	SLC10A3	2,63	1,23
ENSG00000117410	ATP6V0B	2,63	1,29
ENSG00000004059	ARF5	2,62	1,31

ensembl gene	gene symbol	Score(d)	Fold Change
ENSG00000171824	EXOSC10	2,62	1,26
ENSG00000119953	SMNDC1	2,62	1,36
ENSG00000184502	GAST	2,62	1,17
ENSG00000171867	PRNP	2,62	1,40
ENSG00000172602	RND1	2,62	1,28
ENSG00000110958	PTGES3	2,62	1,27
ENSG00000141551	CSNK1D	2,62	1,32
ENSG00000092621	PHGDH	2,62	1,73
ENSG00000188786	MTF1	2,62	1,37
ENSG00000135900	MRPL44	2,62	1,24
ENSG00000159459	UBR1	2,61	1,37
ENSG00000161040	FBXL13	2,61	1,40
ENSG00000147419	CCDC25	2,61	1,32
ENSG00000187980	PLA2G2C	2,61	1,23
ENSG00000144029	MRPS5	2,61	1,25
ENSG00000182584	ACTL10	2,61	1,16
ENSG00000131019	ULBP3	2,61	1,27
ENSG00000118680	MYL12B	2,61	1,40
ENSG00000164951	PDP1	2,61	1,28
ENSG00000117682	DHDDS	2,61	1,32
ENSG00000171314	PGAM1	2,61	1,46
ENSG00000173812	EIF1	2,61	1,13
ENSG00000126458	RRAS	2,60	1,92
ENSG00000196550	FAM72A	2,60	1,40
ENSG00000135404	CD63	2,60	1,59
ENSG00000166747	AP1G1	2,60	1,44
ENSG00000127774	EMC6	2,60	1,23
ENSG00000134684	YARS	2,60	1,37
ENSG00000278540	ACACA	2,60	1,32
ENSG00000187097	ENTPD5	2,60	1,35
ENSG00000114942	EEF1B2	2,60	1,50
ENSG00000083520	DIS3	2,60	1,37
ENSG00000092841	MYL6	2,60	1,33
ENSG00000196381	ZNF781	2,59	1,25
ENSG00000182890	GLUD2	2,59	1,23
ENSG00000103550	KNOP1	2,59	1,36
ENSG00000148688	RPP30	2,59	1,28
ENSG00000148680	HTR7	2,59	1,25
ENSG00000144035	NAT8	2,59	1,20
ENSG00000261115	TMEM178B	2,59	1,27
ENSG00000156313	RPGR	2,58	1,33
ENSG00000163931	TKT	2,58	1,48
ENSG00000100814	CCNB1IP1	2,58	1,77
ENSG00000140941	MAP1LC3B	2,58	1,63
ENSG00000127337	YEATS4	2,58	1,32
ENSG00000100629	CEP128	2,58	1,71
ENSG00000143622	RIT1	2,58	1,37
ENSG00000175793	SFN	2,58	1,29
ENSG00000179912	R3HDM2	2,58	1,40

ensembl gene	gene symbol	Score(d)	Fold Change
ENSG00000183287	CCBE1	2,58	1,22
ENSG00000204389	HSPA1A	2,58	2,39
ENSG00000187735	TCEA1	2,58	1,30
ENSG00000180929	GPR62	2,57	1,13
ENSG00000176542	USF3	2,57	1,28
ENSG00000075188	NUP37	2,57	1,38
ENSG00000135776	ABCB10	2,57	1,25
ENSG00000184564	SLITRK6	2,57	1,15
ENSG00000089101	CFAP61	2,57	1,31
ENSG00000136404	TM6SF1	2,57	2,01
ENSG00000105639	JAK3	2,57	1,48
ENSG00000164032	H2AFZ	2,57	1,33
ENSG00000243910	TUBA4B	2,57	1,16
ENSG00000182107	TMEM30B	2,57	1,35
ENSG00000147274	RBMX	2,57	1,38
ENSG00000163098	BIRC8	2,57	1,20
ENSG00000103978	TMEM87A	2,57	1,27
ENSG00000114439	BBX	2,57	1,36
ENSG00000130487	KLHDC7B	2,57	1,37
ENSG00000136048	DRAM1	2,56	1,70
ENSG00000177971	IMP3	2,56	1,23
ENSG00000164211	STARD4	2,56	1,40
ENSG00000213085	CFAP45	2,56	1,40
ENSG00000167550	RHEBL1	2,56	1,32
ENSG00000178623	GPR35	2,56	1,17
ENSG00000244752	CRYBB2	2,56	1,13
ENSG00000117595	IRF6	2,56	1,38
ENSG00000100916	BRMS1L	2,56	1,63
ENSG00000103145	HCFC1R1	2,56	1,23
ENSG00000197622	CDC42SE1	2,56	1,40
ENSG00000129910	CDH15	2,56	1,20
ENSG00000165264	NDUFB6	2,56	1,33
ENSG00000176022	B3GALT6	2,56	1,23
ENSG00000150403	TMCO3	2,55	1,46
ENSG00000180878	C11orf42	2,55	1,16
ENSG00000261609	GAN	2,55	1,11
ENSG00000150779	TIMM8B	2,55	1,31
ENSG00000058729	RIOK2	2,55	1,34
ENSG00000102683	SGCG	2,55	1,19
ENSG00000170633	RNF34	2,55	1,23
ENSG00000163794	UCN	2,55	1,14
ENSG00000155008	APOOL	2,55	1,22
ENSG00000183779	ZNF703	2,55	1,33
ENSG00000135632	SMYD5	2,55	1,27
ENSG00000087206	UIMC1	2,55	1,30
ENSG00000135378	PRRG4	2,55	2,05
ENSG00000176390	CRLF3	2,55	1,30
ENSG00000165449	SLC16A9	2,55	1,32
ENSG00000182199	SHMT2	2,55	1,54

ensembl gene	gene symbol	Score(d)	Fold Change
ENSG00000114854	TNNC1	2,54	1,22
ENSG00000134160	TRPM1	2,54	1,15
ENSG00000128510	CPA4	2,54	1,19
ENSG00000163491	NEK10	2,54	1,20
ENSG00000134575	ACP2	2,54	1,37
ENSG00000123992	DNPEP	2,54	1,21
ENSG00000173110	HSPA6	2,54	1,61
ENSG00000070669	ASNS	2,54	1,50
ENSG00000198742	SMURF1	2,54	1,51
ENSG00000100784	RPS6KA5	2,54	1,46
ENSG00000011114	BTBD7	2,54	1,30
ENSG00000109390	NDUFC1	2,54	1,27
ENSG00000123349	PFDN5	2,54	1,30
ENSG00000103429	BFAR	2,54	1,21
ENSG00000177885	GRB2	2,54	1,25
ENSG00000172890	NADSYN1	2,53	1,23
ENSG00000236981	OR10G9	2,53	1,16
ENSG00000134108	ARL8B	2,53	1,34
ENSG00000172867	KRT2	2,53	1,18
ENSG00000137692	DCUN1D5	2,53	1,44
ENSG00000164604	GPR85	2,53	1,24
ENSG00000165410	CFL2	2,53	1,50
ENSG00000090104	RGS1	2,53	2,32
ENSG00000065978	YBX1	2,53	1,28
ENSG00000165309	ARMC3	2,53	1,35
ENSG00000072401	UBE2D1	2,53	1,41
ENSG00000157625	TAB3	2,52	1,33
ENSG00000283361	RP11-569D9.5	2,52	1,14
ENSG00000179051	RCC2	2,52	1,31
ENSG00000129204	USP6	2,52	1,19
ENSG00000185338	SOCS1	2,52	1,63
ENSG00000117000	RLF	2,52	1,36
ENSG00000160410	SHKBP1	2,52	1,21
ENSG00000186795	KCNK18	2,52	1,12
ENSG00000090539	CHRD	2,52	1,29
ENSG00000071243	ING3	2,52	1,24
ENSG00000105556	MIER2	2,52	1,37
ENSG00000053254	FOXN3	2,52	1,26
ENSG00000217555	CKLF	2,52	1,52
ENSG00000115073	ACTR1B	2,51	1,32
ENSG00000173762	CD7	2,51	1,16
ENSG00000183798	EMILIN3	2,51	1,18
ENSG00000137473	TTC29	2,51	1,21
ENSG00000087266	SH3BP2	2,51	1,52
ENSG00000168280	KIF5C	2,51	1,39
ENSG00000179057	IGSF22	2,51	1,14
ENSG00000179817	MRGPRX4	2,51	1,28
ENSG00000198252	STYX	2,51	1,27
ENSG00000168903	BTNL3	2,51	1,18

ensembl gene	gene symbol	Score(d)	Fold Change
ENSG00000187475	HIST1H1T	2,51	2,13
ENSG00000100385	IL2RB	2,51	2,01
ENSG00000109625	CPZ	2,51	1,20
ENSG00000116786	PLEKHM2	2,51	1,29
ENSG00000141933	TPGS1	2,51	1,23
ENSG00000124812	CRISP1	2,51	1,16
ENSG00000255150	EID3	2,51	1,39
ENSG00000120907	ADRA1A	2,50	1,18
ENSG00000116030	SUMO1	2,50	1,26
ENSG00000103522	IL21R	2,50	1,41
ENSG00000157093	LYZL4	2,50	1,18
ENSG00000169139	UBE2V2	2,50	1,39
ENSG00000205126	ACCSL	2,50	1,16
ENSG00000128805	ARHGAP22	2,50	1,49
ENSG00000120837	NFYB	2,50	1,39
ENSG00000115539	PDCL3	2,50	1,59
ENSG00000099290	FAM21A	2,50	1,28
ENSG00000175193	PARL	2,50	1,32
ENSG00000137200	CMTR1	2,50	1,24
ENSG00000090263	MRPS33	2,50	1,45
ENSG00000126003	PLAGL2	2,50	1,47
ENSG00000054179	ENTPD2	2,50	1,21
ENSG00000148215	OR5C1	2,49	1,13
ENSG00000104325	DECR1	2,49	1,48
ENSG00000115263	GCG	2,49	1,31
ENSG00000109606	DHX15	2,49	1,25
ENSG00000133134	BEX2	2,49	1,40
ENSG00000142168	SOD1	2,49	1,29
ENSG00000163541	SUCLG1	2,49	1,27
ENSG00000114353	GNAI2	2,49	1,21
ENSG00000100296	THOC5	2,49	1,31
ENSG00000116903	EXOC8	2,49	1,41
ENSG00000163902	RPN1	2,49	1,48
ENSG00000187516	HYPM	2,49	1,19
ENSG00000216937	CCDC7	2,49	1,46
ENSG00000163346	PBXIP1	2,49	1,58
ENSG00000076604	TRAF4	2,48	1,33
ENSG00000183684	ALYREF	2,48	1,24
ENSG00000170909	OSCAR	2,48	1,28
ENSG00000105186	ANKRD27	2,48	1,38
ENSG00000174562	KLK15	2,48	1,22
ENSG00000074755	ZZEF1	2,48	1,24
ENSG00000165389	SPTSSA	2,48	1,22
ENSG00000101193	GID8	2,48	1,17
ENSG00000103254	FAM173A	2,48	1,31
ENSG00000167770	OTUB1	2,48	1,17
ENSG00000188672	RHCE	2,48	1,25
ENSG00000085832	EPS15	2,48	1,33
ENSG00000128578	STRIP2	2,48	1,20

ensembl gene	gene symbol	Score(d)	Fold Change
ENSG00000198920	KIAA0753	2,48	1,28
ENSG00000183283	DAZAP2	2,48	1,28
ENSG00000173905	GOLIM4	2,48	1,33
ENSG00000164338	UTP15	2,47	1,26
ENSG00000117569	PTBP2	2,47	1,50
ENSG00000258465	RP11-574F21.3	2,47	1,17
ENSG00000181004	BBS12	2,47	1,26
ENSG00000079335	CDC14A	2,47	1,89
ENSG00000165806	CASP7	2,47	1,43
ENSG00000107521	HPS1	2,47	1,19
ENSG00000205629	LCMT1	2,47	1,26
ENSG00000186834	HEXIM1	2,47	1,38
ENSG00000111727	HCFC2	2,47	1,56
ENSG00000168397	ATG4B	2,47	1,35
ENSG00000204019	CT83	2,47	1,16
ENSG00000127603	MACF1	2,47	1,27
ENSG00000101327	PDYN	2,46	1,15
ENSG00000186281	GPAT2	2,46	1,50
ENSG00000144228	SPOPL	2,46	1,41
ENSG00000105398	SULT2A1	2,46	1,16
ENSG00000142864	SERBP1	2,46	1,32
ENSG00000188542	DUSP28	2,46	1,37
ENSG00000159840	ZYX	2,46	1,51
ENSG00000126653	NSRP1	2,46	1,19
ENSG00000085788	DDHD2	2,46	1,32
ENSG00000103275	UBE2I	2,46	1,28
ENSG00000213638	ADAT3	2,46	1,22
ENSG00000100226	GTPBP1	2,46	1,30
ENSG00000124449	IRGC	2,46	1,22
ENSG00000015153	YAF2	2,46	1,41
ENSG00000155816	FMN2	2,46	1,19
ENSG00000130150	MOSPD2	2,46	1,35
ENSG00000179988	PSTK	2,45	1,26
ENSG00000172331	BPGM	2,45	1,39
ENSG00000135249	RINT1	2,45	1,24
ENSG00000111087	GLI1	2,45	1,17
ENSG00000066027	PPP2R5A	2,45	1,39
ENSG00000007376	RPUSD1	2,45	1,25
ENSG00000196872	KIAA1211L	2,45	1,28
ENSG00000132382	MYBBP1A	2,45	1,23
ENSG00000126062	TMEM115	2,45	1,20
ENSG00000121680	PEX16	2,45	1,18
ENSG00000104518	GSDMD	2,45	1,36
ENSG00000152409	JMY	2,45	1,46
ENSG00000055070	SZRD1	2,45	1,26
ENSG00000130635	COL5A1	2,44	1,10
ENSG00000138796	HADH	2,44	1,50
ENSG00000186020	ZNF529	2,44	1,58
ENSG00000132589	FLOT2	2,44	1,28

ensembl gene	gene symbol	Score(d)	Fold Change
ENSG00000205856	C22orf42	2,44	1,19
ENSG00000250120	PCDHA10	2,44	1,17
ENSG00000137996	RTCA	2,44	1,32
ENSG00000177602	GSG2	2,44	1,40
ENSG00000078668	VDAC3	2,44	1,40
ENSG00000172586	CHCHD1	2,44	1,37
ENSG00000110244	APOA4	2,43	1,14
ENSG00000165688	PMPCA	2,43	1,29
ENSG00000177084	POLE	2,43	1,33
ENSG00000126895	AVPR2	2,43	1,14
ENSG00000145476	CYP4V2	2,43	1,74
ENSG00000132825	PPP1R3D	2,43	1,17
ENSG00000108774	RAB5C	2,43	1,27
ENSG00000187950	OVCH1	2,43	1,25
ENSG00000104442	ARMC1	2,43	1,38
ENSG00000145912	NHP2	2,42	1,33
ENSG00000139154	AEBP2	2,42	1,29
ENSG00000188763	FZD9	2,42	1,16
ENSG00000104381	GDAP1	2,42	1,50
ENSG00000139200	PIANP	2,42	1,22
ENSG00000185958	FAM186A	2,42	1,09
ENSG00000180921	FAM83H	2,42	1,21
ENSG00000168454	TXNDC2	2,42	1,18
ENSG00000143207	RFWD2	2,42	1,31
ENSG00000164615	CAMLG	2,42	1,31
ENSG00000104320	NBN	2,42	1,41
ENSG00000126215	XRCC3	2,42	1,19
ENSG00000031081	ARHGAP31	2,42	1,41
ENSG00000135220	UGT2A3	2,42	1,19
ENSG00000129518	EAPP	2,42	1,30
ENSG00000134897	BIVM	2,42	1,43
ENSG00000128513	POT1	2,41	1,42
ENSG00000186063	AIDA	2,41	1,57
ENSG00000183166	CALN1	2,41	1,20
ENSG00000169436	COL22A1	2,41	1,37
ENSG00000108179	PPIF	2,41	1,36
ENSG00000176244	ACBD7	2,41	1,19
ENSG00000105499	PLA2G4C	2,41	1,43
ENSG00000171431	KRT20	2,41	1,26
ENSG00000006715	VPS41	2,41	1,35
ENSG00000116459	ATP5F1	2,41	1,23
ENSG00000135245	HILPDA	2,41	1,47
ENSG00000165704	HPRT1	2,40	1,47
ENSG00000204574	ABCF1	2,40	1,21
ENSG00000174720	LARP7	2,40	1,28
ENSG00000137251	TINAG	2,40	1,24
ENSG00000206026	SMIM21	2,40	1,24
ENSG00000146809	ASB15	2,40	1,13
ENSG00000144821	MYH15	2,40	1,43

ensembl gene	gene symbol	Score(d)	Fold Change
ENSG00000150316	CWC15	2,40	1,20
ENSG00000122483	CCDC18	2,40	1,48
ENSG00000037280	FLT4	2,40	1,16
ENSG00000170965	PLAC1	2,40	1,52
ENSG00000119599	DCAF4	2,40	1,28
ENSG00000108384	RAD51C	2,40	1,44
ENSG00000255552	LY6G6E	2,40	1,21
ENSG00000182957	SPATA13	2,40	2,05
ENSG00000177683	THAP5	2,40	1,36
ENSG00000144369	FAM171B	2,40	1,93
ENSG00000164609	SLU7	2,40	1,25
ENSG00000104756	KCTD9	2,40	1,45
ENSG00000196131	VN1R2	2,39	1,29
ENSG00000023445	BIRC3	2,39	2,49
ENSG00000204396	VWA7	2,39	1,27
ENSG00000006695	COX10	2,39	1,31
ENSG00000163530	DPPA2	2,39	1,22
ENSG00000033867	SLC4A7	2,39	1,43
ENSG00000121644	DESI2	2,39	1,32
ENSG00000025708	TYMP	2,39	1,52
ENSG00000166189	HPS6	2,39	1,14
ENSG00000164935	DCSTAMP	2,39	1,19
ENSG00000080618	CPB2	2,39	1,14
ENSG00000123575	FAM199X	2,39	1,30
ENSG00000205323	SARNP	2,39	1,48
ENSG00000128050	PAICS	2,38	1,39
ENSG00000132405	TBC1D14	2,38	1,26
ENSG00000185236	RAB11B	2,38	1,18
ENSG00000151748	SAV1	2,38	1,63
ENSG00000020633	RUNX3	2,38	1,42
ENSG00000171798	KNDC1	2,38	1,20
ENSG00000185105	MYADML2	2,38	1,16
ENSG00000166292	TMEM100	2,38	1,19
ENSG00000180044	C3orf80	2,38	1,20
ENSG00000153498	SPACA7	2,38	1,15
ENSG00000162755	KLHDC9	2,38	1,34
ENSG00000125430	HS3ST3B1	2,38	1,71
ENSG00000134594	RAB33A	2,38	1,72
ENSG00000057657	PRDM1	2,38	1,27
ENSG00000059691	GATB	2,38	1,33
ENSG00000156273	BACH1	2,38	1,35
ENSG00000137337	MDC1	2,38	1,18
ENSG00000213281	NRAS	2,38	1,36
ENSG00000186575	NF2	2,38	1,31
ENSG00000123908	AGO2	2,38	1,40
ENSG00000178105	DDX10	2,37	1,44
ENSG00000163618	CADPS	2,37	1,95
ENSG00000168273	SMIM4	2,37	1,21
ENSG00000099985	OSM	2,37	1,25

ensembl gene	gene symbol	Score(d)	Fold Change
ENSG00000137154	RPS6	2,37	1,36
ENSG00000125850	OVOL2	2,37	1,17
ENSG00000100731	PCNX1	2,37	1,50
ENSG00000260596	DUX4	2,37	1,45
ENSG00000204583	LRCOL1	2,37	1,12
ENSG00000159658	EFCAB14	2,36	1,25
ENSG00000198938	MT-CO3	2,36	1,08
ENSG00000133131	MORC4	2,36	1,41
ENSG00000018610	CXorf56	2,36	1,31
ENSG00000117010	ZNF684	2,36	1,34
ENSG00000188991	SLC15A5	2,36	1,12
ENSG00000169306	IL1RAPL1	2,36	1,12
ENSG00000117395	EBNA1BP2	2,36	1,24
ENSG00000133169	BEX1	2,36	1,51
ENSG00000166710	B2M	2,36	1,24
ENSG00000196376	SLC35F1	2,36	1,96
ENSG00000100138	SNU13	2,36	1,30
ENSG00000168930	TRIM49	2,36	1,23
ENSG00000164236	ANKRD33B	2,36	1,31
ENSG00000140284	SLC27A2	2,36	1,53
ENSG00000130720	FIBCD1	2,35	1,25
ENSG00000159708	LRRC36	2,35	1,23
ENSG00000173821	RNF213	2,35	1,46
ENSG00000143401	ANP32E	2,35	1,38
ENSG00000164045	CDC25A	2,35	1,29
ENSG00000143324	XPR1	2,35	1,32
ENSG00000178096	BOLA1	2,35	1,44
ENSG00000186462	NAP1L2	2,35	2,08
ENSG00000187323	DCC	2,35	2,57
ENSG00000254685	FPGT	2,35	1,37
ENSG00000064726	BTBD1	2,35	1,39
ENSG00000130943	PKDREJ	2,35	1,13
ENSG00000143314	MRPL24	2,35	1,50
ENSG00000172421	EFCAB3	2,35	1,17
ENSG00000135677	GNS	2,35	1,24
ENSG00000139168	ZCRB1	2,35	1,24
ENSG00000180745	CLRN3	2,35	1,12
ENSG00000172262	ZNF131	2,35	1,26
ENSG00000176749	CDK5R1	2,35	1,27
ENSG00000111266	DUSP16	2,35	1,23
ENSG00000163689	C3orf67	2,35	1,41
ENSG00000059758	CDK17	2,34	1,31
ENSG00000015475	BID	2,34	1,22
ENSG00000107560	RAB11FIP2	2,34	1,25
ENSG00000241697	TMEFF1	2,34	1,33
ENSG00000116670	MAD2L2	2,34	1,30
ENSG00000147041	SYTL5	2,34	1,28
ENSG00000167258	CDK12	2,34	1,18
ENSG00000123892	RAB38	2,34	1,23

ensembl gene	gene symbol	Score(d)	Fold Change
ENSG00000100109	TFIP11	2,34	1,20
ENSG00000127946	HIP1	2,34	1,36
ENSG00000187624	C17orf97	2,34	1,24
ENSG00000053900	ANAPC4	2,34	1,33
ENSG00000148719	DNAJB12	2,34	1,23
ENSG00000172020	GAP43	2,34	1,17
ENSG00000134107	BHLHE40	2,34	1,74
ENSG00000186566	GPATCH8	2,34	1,18
ENSG00000168118	RAB4A	2,34	1,50
ENSG00000175018	TEX36	2,33	1,15
ENSG00000136045	PWP1	2,33	1,24
ENSG00000130675	MNX1	2,33	1,20
ENSG00000204616	TRIM31	2,33	1,17
ENSG00000255274	TMPRSS4-AS1	2,33	1,22
ENSG00000114933	INO80D	2,33	1,26
ENSG00000163866	SMIM12	2,33	1,20
ENSG00000197586	ENTPD6	2,33	1,24
ENSG00000119203	CPSF3	2,33	1,25
ENSG00000169727	GPS1	2,33	1,24
ENSG00000136286	MYO1G	2,33	1,77
ENSG00000125363	AMELX	2,32	1,24
ENSG00000125827	TMX4	2,32	1,56
ENSG00000181192	DHTKD1	2,32	1,24
ENSG00000050767	COL23A1	2,32	1,22
ENSG00000085644	ZNF213	2,32	1,18
ENSG00000163811	WDR43	2,32	1,30
ENSG00000256713	PGA5	2,32	1,32
ENSG00000165985	C1QL3	2,32	1,54
ENSG00000143450	OAZ3	2,32	1,21
ENSG00000105141	CASP14	2,32	1,26
ENSG00000108100	CCNY	2,32	1,21
ENSG00000145911	N4BP3	2,32	1,19
ENSG00000152969	JAKMIP1	2,32	1,17
ENSG00000165175	MID1IP1	2,32	1,36
ENSG00000099977	DDT	2,32	1,31
ENSG00000205302	SNX2	2,31	1,31
ENSG00000265354	TIMM23	2,31	1,22
ENSG00000135842	FAM129A	2,31	2,04
ENSG00000138382	METTL5	2,31	1,28
ENSG00000107807	TLX1	2,31	1,28
ENSG00000166159	LRTM2	2,31	1,24
ENSG00000188993	LRRC66	2,31	1,25
ENSG00000124207	CSE1L	2,31	1,31
ENSG00000187796	CARD9	2,31	1,16
ENSG00000186288	PABPC1L2A	2,31	1,17
ENSG00000117153	KLHL12	2,31	1,32
ENSG00000086666	ZFAND6	2,31	1,32
ENSG00000275896	PRSS2	2,31	1,22
ENSG00000155592	ZKSCAN2	2,31	1,22

ensembl gene	gene symbol	Score(d)	Fold Change
ENSG00000108639	SYNGR2	2,31	1,35
ENSG00000090061	CCNK	2,31	1,23
ENSG00000163754	GYG1	2,31	1,34
ENSG00000142230	SAE1	2,31	1,29
ENSG00000186625	KATNA1	2,31	1,24
ENSG00000034677	RNF19A	2,30	1,36
ENSG00000134419	RPS15A	2,30	1,44
ENSG00000145423	SFRP2	2,30	1,62
ENSG00000120211	INSL4	2,30	1,14
ENSG00000135625	EGR4	2,30	1,15
ENSG00000237110	TAAR9	2,30	1,14
ENSG00000130052	STARD8	2,30	1,35
ENSG00000180988	OR52N2	2,30	1,16
ENSG00000162650	ATXN7L2	2,30	1,19
ENSG00000132323	ILKAP	2,30	1,26
ENSG00000205838	TTC23L	2,30	1,16
ENSG00000034533	ASTE1	2,30	1,27
ENSG00000239732	TLR9	2,30	1,40
ENSG00000183864	TOB2	2,30	1,36
ENSG00000206549	PRSS50	2,30	1,09
ENSG00000101752	MIB1	2,30	1,31
ENSG00000243710	CFAP57	2,29	1,15
ENSG00000105393	BABAM1	2,29	1,27
ENSG00000163216	SPRR2D	2,29	1,30
ENSG00000206557	TRIM71	2,29	1,14
ENSG00000160445	ZER1	2,29	1,19
ENSG00000162734	PEA15	2,29	1,47
ENSG00000142544	CTU1	2,29	1,21
ENSG00000173726	TOMM20	2,29	1,51
ENSG00000088386	SLC15A1	2,29	1,14
ENSG00000141279	NPEPPS	2,29	1,33
ENSG00000116885	OSCP1	2,29	1,31
ENSG00000006611	USH1C	2,29	1,21
ENSG00000171488	LRRC8C	2,29	1,61
ENSG00000184156	KCNQ3	2,29	1,41
ENSG00000120948	TARDBP	2,29	1,54
ENSG00000169016	E2F6	2,29	1,27
ENSG00000188649	CC2D2B	2,29	1,31
ENSG00000106436	MYL10	2,29	1,13
ENSG00000163510	CWC22	2,29	1,27
ENSG00000145632	PLK2	2,29	1,82
ENSG00000235863	B3GALT4	2,29	1,30
ENSG00000108175	ZMIZ1	2,29	1,77
ENSG00000102225	CDK16	2,28	1,21
ENSG00000125454	SLC25A19	2,28	1,42
ENSG00000151694	ADAM17	2,28	1,35
ENSG00000186104	CYP2R1	2,28	1,39
ENSG00000278053	DDX52	2,28	1,27
ENSG00000206432	TMEM200C	2,28	1,12

ensembl gene	gene symbol	Score(d)	Fold Change
ENSG00000143436	MRPL9	2,28	1,22
ENSG00000122687	MRM2	2,28	1,25
ENSG00000135722	FBXL8	2,28	1,17
ENSG00000174373	RALGAPA1	2,28	1,22
ENSG00000111605	CPSF6	2,28	1,25
ENSG00000121067	SPOP	2,28	1,32
ENSG00000164944	KIAA1429	2,28	1,33
ENSG00000185559	DLK1	2,28	1,25
ENSG00000125945	ZNF436	2,28	1,25
ENSG00000236609	ZNF853	2,28	1,13
ENSG00000272886	DCP1A	2,28	1,24
ENSG00000158764	ITLN2	2,28	1,15
ENSG00000125089	SH3TC1	2,28	1,30
ENSG00000131653	TRAF7	2,28	1,31
ENSG00000115419	GLS	2,28	1,25
ENSG00000128641	MYO1B	2,28	1,77
ENSG00000160695	VPS11	2,28	1,20
ENSG00000246705	H2AFJ	2,28	1,42
ENSG00000244274	DBNDD2	2,28	1,24
ENSG00000080007	DDX43	2,28	1,62
ENSG00000150787	PTS	2,27	1,21
ENSG00000187642	PERM1	2,27	1,13
ENSG00000197619	ZNF615	2,27	1,50
ENSG00000188227	ZNF793	2,27	1,24
ENSG00000021574	SPAST	2,27	1,22
ENSG00000239810	PRAMEF11	2,27	1,19
ENSG00000054690	PLEKHH1	2,27	1,38
ENSG00000077713	SLC25A43	2,27	1,34
ENSG00000084676	NCOA1	2,27	1,18
ENSG00000186075	ZPBP2	2,27	1,30
ENSG00000188095	MESP2	2,27	1,23
ENSG00000267855	NDUFA7	2,27	1,57
ENSG00000171621	SPSB1	2,27	1,54
ENSG00000109466	KLHL2	2,27	1,32
ENSG00000129235	TXNDC17	2,27	1,34
ENSG00000029363	BCLAF1	2,27	1,28
ENSG00000107282	APBA1	2,27	1,24
ENSG00000242732	RGAG4	2,26	1,32
ENSG00000070831	CDC42	2,26	1,36
ENSG00000131697	NPHP4	2,26	1,16
ENSG00000104626	ERI1	2,26	1,36
ENSG00000080910	CFHR2	2,26	1,35
ENSG00000155304	HSPA13	2,26	1,23
ENSG00000232838	PET117	2,26	1,19
ENSG00000100749	VRK1	2,26	1,52
ENSG00000164112	TMEM155	2,26	1,11
ENSG00000100312	ACR	2,26	1,14
ENSG00000176399	DMRTA1	2,26	1,32
ENSG00000109787	KLF3	2,26	1,33

ensembl gene	gene symbol	Score(d)	Fold Change
ENSG00000151445	VIPAS39	2,26	1,31
ENSG00000073670	ADAM11	2,26	1,22
ENSG00000164284	GRPEL2	2,25	1,32
ENSG00000125492	BARHL1	2,25	1,16
ENSG00000205277	MUC12	2,25	1,13
ENSG00000196843	ARID5A	2,25	1,61
ENSG00000147872	PLIN2	2,25	1,17
ENSG00000179262	RAD23A	2,25	1,17
ENSG00000171103	TRMT61B	2,25	1,32
ENSG00000178125	PPP1R42	2,25	1,11
ENSG00000147457	CHMP7	2,25	1,25
ENSG00000235387	LINC00961	2,25	1,13
ENSG00000155438	NIFK	2,25	1,29
ENSG00000158156	XKR8	2,25	1,32
ENSG00000147439	BIN3	2,24	1,31
ENSG00000213047	DENND1B	2,24	1,37
ENSG00000067560	RHOA	2,24	1,12
ENSG00000183559	C10orf120	2,24	1,16
ENSG00000130338	TULP4	2,24	1,31
ENSG00000151806	GUF1	2,24	1,29
ENSG00000188191	PRKAR1B	2,24	1,25
ENSG00000196465	MYL6B	2,24	1,28
ENSG00000144566	RAB5A	2,24	1,22
ENSG00000161904	LEMD2	2,24	1,25
ENSG00000105948	TTC26	2,24	1,26
ENSG00000177989	ODF3B	2,24	1,48
ENSG00000181016	LSMEM1	2,24	1,31
ENSG00000090238	YPEL3	2,24	1,37
ENSG00000225921	NOL7	2,24	1,23
ENSG00000169813	HNRNPF	2,24	1,20
ENSG00000104976	SNAPC2	2,24	1,16
ENSG00000137673	MMP7	2,24	1,17
ENSG00000170381	SEMA3E	2,24	1,30
ENSG00000214753	HNRNPUL2	2,24	1,57
ENSG00000055211	GINM1	2,23	1,56
ENSG00000255501	CARD18	2,23	1,15
ENSG00000184908	CLCNKB	2,23	1,28
ENSG00000187848	P2RX2	2,23	1,13
ENSG00000164897	TMUB1	2,23	1,26
ENSG00000174667	OR7D4	2,23	1,21
ENSG00000099864	PALM	2,23	1,21
ENSG00000105856	HBP1	2,23	1,34
ENSG00000158639	PAGE5	2,23	1,54
ENSG00000109320	NFKB1	2,23	1,43
ENSG00000134802	SLC43A3	2,23	1,37
ENSG00000253649	PRSS51	2,23	1,95
ENSG00000106236	NPTX2	2,23	1,18
ENSG00000178974	FBXO34	2,23	1,35
ENSG00000153714	LURAP1L	2,23	1,79

ensembl gene	gene symbol	Score(d)	Fold Change
ENSG00000173926	42797	2,23	1,59
ENSG00000197467	COL13A1	2,23	1,14
ENSG00000188372	ZP3	2,23	1,45
ENSG00000100311	PDGFB	2,22	1,21
ENSG00000182521	TBPL2	2,22	1,16
ENSG00000131378	RFTN1	2,22	1,37
ENSG00000274736	CCL23	2,22	1,14
ENSG00000173674	EIF1AX	2,22	1,40
ENSG00000169271	HSPB3	2,22	1,11
ENSG00000205869	KRTAP5-1	2,22	1,13
ENSG00000253958	CLDN23	2,22	1,13
ENSG00000141682	PMAIP1	2,22	2,86
ENSG00000145362	ANK2	2,22	1,35
ENSG00000101222	SPEF1	2,22	1,12
ENSG00000162877	PM20D1	2,22	1,55
ENSG00000166311	SMPD1	2,22	1,32
ENSG00000147437	GNRH1	2,22	1,21
ENSG00000168615	ADAM9	2,22	1,65
ENSG00000105248	CCDC94	2,22	1,22
ENSG00000255974	CYP2A6	2,22	1,13
ENSG00000137312	FLOT1	2,22	1,24
ENSG00000163734	CXCL3	2,22	1,16
ENSG00000163900	TMEM41A	2,21	1,37
ENSG00000143753	DEGS1	2,21	1,36
ENSG00000138758	42989	2,21	1,37
ENSG00000105605	CACNG7	2,21	1,23
ENSG00000101935	AMMECR1	2,21	1,39
ENSG00000133773	CCDC59	2,21	1,32
ENSG00000144746	ARL6IP5	2,21	1,48
ENSG00000073067	CYP2W1	2,21	1,16
ENSG00000164649	CDCA7L	2,21	1,41
ENSG00000179580	RNF151	2,21	1,13
ENSG00000151470	C4orf33	2,21	1,52
ENSG00000151338	MIPOL1	2,21	1,11
ENSG00000084070	SMAP2	2,21	1,54
ENSG00000165113	GKAP1	2,21	1,49
ENSG00000166788	SAAL1	2,21	1,35
ENSG00000105409	ATP1A3	2,21	1,40
ENSG00000101974	ATP11C	2,21	1,36
ENSG00000007171	NOS2	2,20	1,13
ENSG00000133226	SRRM1	2,20	1,31
ENSG00000159210	SNF8	2,20	1,19
ENSG00000128989	ARPP19	2,20	1,29
ENSG00000129951	PLPPR3	2,20	1,12
ENSG00000110446	SLC15A3	2,20	1,46
ENSG00000102103	PQBP1	2,20	1,17
ENSG00000038210	PI4K2B	2,19	1,17
ENSG00000168924	LETM1	2,19	1,32
ENSG00000132680	KIAA0907	2,19	1,38

ensembl gene	gene symbol	Score(d)	Fold Change
ENSG00000171806	METTL18	2,19	1,29
ENSG00000214756	METTL12	2,19	1,47
ENSG00000090020	SLC9A1	2,19	1,26
ENSG00000154124	OTULIN	2,19	1,32
ENSG00000167272	POP5	2,19	1,35
ENSG00000131979	GCH1	2,19	1,51
ENSG00000132002	DNAJB1	2,19	1,27
ENSG00000178338	ZNF354B	2,19	1,15
ENSG00000120949	TNFRSF8	2,19	1,15
ENSG00000187398	LUZP2	2,19	1,15
ENSG00000132681	ATP1A4	2,19	1,10
ENSG00000120509	PDZD11	2,19	1,24
ENSG00000249992	TMEM158	2,19	1,12
ENSG00000188015	S100A3	2,19	1,11
ENSG00000141543	EIF4A3	2,19	1,40
ENSG00000274252	GGTLC3	2,19	1,40
ENSG00000100156	SLC16A8	2,18	1,13
ENSG00000173275	ZNF449	2,18	1,34
ENSG00000057608	GDI2	2,18	1,24
ENSG00000279745	AL365202.1	2,18	1,37
ENSG00000183734	ASCL2	2,18	1,13
ENSG00000127838	PNKD	2,18	1,16
ENSG00000117505	DR1	2,18	1,32
ENSG00000152952	PLOD2	2,18	2,63
ENSG00000151923	TIAL1	2,18	1,16
ENSG00000198689	SLC9A6	2,18	1,26
ENSG00000131203	IDO1	2,18	1,24
ENSG00000144843	ADPRH	2,18	1,31
ENSG00000198954	KIF1BP	2,18	1,24
ENSG00000171960	PPIH	2,18	1,27
ENSG00000167552	TUBA1A	2,18	1,69
ENSG00000196267	ZNF836	2,18	1,46
ENSG00000084072	PPIE	2,18	1,26
ENSG00000039123	SKIV2L2	2,18	1,25
ENSG00000172661	FAM21C	2,18	1,23
ENSG00000112130	RNF8	2,18	1,25
ENSG00000088782	DEFB127	2,18	1,10
ENSG00000276747	PADI6	2,18	1,13
ENSG00000101470	TNNC2	2,18	1,16
ENSG00000168874	ATOH8	2,18	1,23
ENSG00000239305	RNF103	2,18	1,26
ENSG00000177542	SLC25A22	2,18	1,29
ENSG00000120334	CENPL	2,17	1,22
ENSG00000142408	CACNG8	2,17	1,16
ENSG00000160886	LY6K	2,17	1,11
ENSG00000129219	PLD2	2,17	1,29
ENSG00000205642	VCX3B	2,17	1,26
ENSG00000134398	ERN2	2,17	1,11
ENSG00000119655	NPC2	2,17	1,41

ensembl gene	gene symbol	Score(d)	Fold Change
ENSG00000123159	GIPC1	2,17	1,19
ENSG00000125779	PANK2	2,17	1,19
ENSG00000182783	OR2T29	2,17	1,15
ENSG00000104388	RAB2A	2,17	1,20
ENSG00000085465	OVGP1	2,17	1,19
ENSG00000110046	ATG2A	2,17	1,21
ENSG00000101246	ARFRP1	2,17	1,15
ENSG00000173599	PC	2,17	1,22
ENSG00000100033	PRODH	2,17	1,16
ENSG00000179284	DAND5	2,17	1,12
ENSG00000167670	CHAF1A	2,17	1,29
ENSG00000177096	FAM109B	2,17	1,18
ENSG00000119919	NKX2-3	2,17	1,10
ENSG00000205220	PSMB10	2,17	1,26
ENSG00000114646	CSPG5	2,17	1,15
ENSG00000167011	NAT16	2,16	1,12
ENSG00000134453	RBM17	2,16	1,30
ENSG00000162971	TYW5	2,16	1,29
ENSG00000099889	ARVCF	2,16	1,14
ENSG00000106624	AEBP1	2,16	1,11
ENSG00000115415	STAT1	2,16	1,38
ENSG00000145220	LYAR	2,16	1,42
ENSG00000138698	RAP1GDS1	2,16	1,29
ENSG00000170776	AKAP13	2,16	1,38
ENSG00000169991	IFFO2	2,16	1,40
ENSG00000104907	TRMT1	2,16	1,34
ENSG00000135108	FBXO21	2,16	1,22
ENSG00000170891	CYTL1	2,16	1,24
ENSG00000116906	GNPAT	2,16	1,27
ENSG00000115641	FHL2	2,16	1,18
ENSG00000100319	ZMAT5	2,16	1,17
ENSG00000039068	CDH1	2,16	2,09
ENSG00000156968	MPV17L	2,16	1,40
ENSG00000135472	FAIM2	2,16	1,17
ENSG00000117362	APH1A	2,16	1,28
ENSG00000165995	CACNB2	2,16	1,18
ENSG00000130204	TOMM40	2,16	1,21
ENSG00000106462	EZH2	2,16	1,42
ENSG00000241123	KRTAP10-5	2,16	1,20
ENSG00000151023	ENKUR	2,16	1,15
ENSG00000183347	GBP6	2,16	1,34
ENSG00000163518	FCRL4	2,15	1,25
ENSG00000108443	RPS6KB1	2,15	1,25
ENSG00000005812	FBXL3	2,15	1,35
ENSG00000138433	CIR1	2,15	1,27
ENSG00000206104	KRTAP20-3	2,15	1,16
ENSG00000221968	FADS3	2,15	1,32
ENSG00000141977	CIB3	2,15	1,12
ENSG00000099917	MED15	2,15	1,22

ensembl gene	gene symbol	Score(d)	Fold Change
ENSG00000171863	RPS7	2,15	1,16
ENSG00000089009	RPL6	2,15	1,39
ENSG00000141456	PELP1	2,15	1,32
ENSG00000115561	CHMP3	2,15	1,66
ENSG00000221858	OR2A12	2,15	1,11
ENSG00000100867	DHRS2	2,15	1,12
ENSG00000170791	CHCHD7	2,15	1,41
ENSG00000118620	ZNF430	2,15	1,54
ENSG00000175820	CCDC168	2,14	1,09
ENSG00000114491	UMPS	2,14	1,21
ENSG00000171425	ZNF581	2,14	1,36
ENSG00000203326	ZNF525	2,14	1,53
ENSG00000165527	ARF6	2,14	1,22
ENSG00000132664	POLR3F	2,14	1,34
ENSG00000186895	FGF3	2,14	1,15
ENSG00000198431	TXNRD1	2,14	1,22
ENSG00000005102	MEOX1	2,14	1,14
ENSG00000176986	SEC24C	2,14	1,15
ENSG00000177202	SPACA4	2,14	1,10
ENSG00000204946	ZNF783	2,14	1,26
ENSG00000167780	SOAT2	2,14	1,19
ENSG00000169488	OR4K15	2,14	1,10
ENSG00000163660	CCNL1	2,14	1,21
ENSG00000133124	IRS4	2,14	1,22
ENSG00000067840	PDZD4	2,14	1,14
ENSG00000100079	LGALS2	2,14	1,18
ENSG00000157349	DDX19B	2,13	1,29
ENSG00000171872	KLF17	2,13	1,15
ENSG00000196428	TSC22D2	2,13	1,45
ENSG00000124486	USP9X	2,13	1,30
ENSG00000144681	STAC	2,13	1,30
ENSG00000185753	CXorf38	2,13	1,22
ENSG00000196136	SERPINA3	2,13	1,30
ENSG00000184160	ADRA2C	2,13	1,12
ENSG00000196387	ZNF140	2,13	1,50
ENSG00000112238	PRDM13	2,13	1,15
ENSG00000136939	OR1L4	2,13	1,23
ENSG00000088247	KHSRP	2,13	1,17
ENSG00000004779	NDUFAB1	2,12	1,22
ENSG00000092199	HNRNPC	2,12	1,24
ENSG00000162585	FAAP20	2,12	1,16
ENSG00000121064	SCPEP1	2,12	1,46
ENSG00000155868	MED7	2,12	1,17
ENSG00000167565	SERTAD3	2,12	1,27
ENSG00000143337	TOR1AIP1	2,12	1,23
ENSG00000156384	SFR1	2,12	1,42
ENSG00000170315	UBB	2,12	1,71
ENSG00000238205	MPC1L	2,12	1,11
ENSG00000150045	KLRF1	2,12	2,17

ensembl gene	gene symbol	Score(d)	Fold Change
ENSG00000143164	DCAF6	2,12	1,27
ENSG00000115840	SLC25A12	2,12	1,26
ENSG00000101057	MYBL2	2,12	1,32
ENSG00000198399	ITSN2	2,12	1,18
ENSG00000198911	SREBF2	2,12	1,22
ENSG00000141526	SLC16A3	2,12	1,56
ENSG00000158122	AAED1	2,12	1,46
ENSG00000155636	RBM45	2,12	1,23
ENSG00000198133	TMEM229B	2,12	1,23
ENSG00000113851	CRBN	2,12	1,18
ENSG00000131808	FSHB	2,12	1,15
ENSG00000119686	FLVCR2	2,11	1,19
ENSG00000140320	BAHD1	2,11	1,22
ENSG00000197838	CYP2A13	2,11	1,14
ENSG00000102034	ELF4	2,11	1,51
ENSG00000117528	ABCD3	2,11	1,35
ENSG00000250378	RP11-119J18.1	2,11	1,13
ENSG00000154370	TRIM11	2,11	1,23
ENSG00000196517	SLC6A9	2,11	1,44
ENSG00000112081	SRSF3	2,11	1,14
ENSG00000123595	RAB9A	2,11	1,30
ENSG00000152102	FAM168B	2,11	1,18
ENSG00000175324	LSM1	2,11	1,24
ENSG00000168275	COA6	2,11	1,33
ENSG00000214087	ARL16	2,11	1,25
ENSG00000122965	RBM19	2,11	1,36
ENSG00000153292	ADGRF1	2,11	1,17
ENSG00000187824	TMEM220	2,11	1,57
ENSG00000173141	MRPL57	2,10	1,29
ENSG00000144233	AMMECR1L	2,10	1,18
ENSG00000179528	LBX2	2,10	1,20
ENSG00000132718	SYT11	2,10	1,24
ENSG00000101596	SMCHD1	2,10	1,20
ENSG00000169126	ARMC4	2,10	1,55
ENSG00000174448	STARD6	2,10	1,21
ENSG00000213928	IRF9	2,10	1,29
ENSG00000185158	LRRC37B	2,10	1,30
ENSG00000140374	ETFA	2,10	1,29
ENSG00000257057	C11orf97	2,10	1,10
ENSG00000121350	PYROXD1	2,10	1,37
ENSG00000163395	IGFN1	2,10	1,26
ENSG00000203784	LELP1	2,10	1,11
ENSG00000153774	CFDP1	2,10	1,26
ENSG00000140829	DHX38	2,09	1,19
ENSG00000080815	PSEN1	2,09	1,27
ENSG00000185104	FAF1	2,09	1,25
ENSG00000217442	SYCE3	2,09	1,14
ENSG00000129038	LOXL1	2,09	1,23
ENSG00000091664	SLC17A6	2,09	1,22

ensembl gene	gene symbol	Score(d)	Fold Change
ENSG00000043591	ADRB1	2,09	1,18
ENSG00000147036	LANCL3	2,09	1,16
ENSG00000066230	SLC9A3	2,09	1,11
ENSG00000156030	ELMSAN1	2,09	1,27
ENSG00000156671	SAMD8	2,09	1,33
ENSG00000145425	RPS3A	2,09	1,22
ENSG00000125351	UPF3B	2,09	1,14
ENSG00000105821	DNAJC2	2,09	1,22
ENSG00000137463	MGARP	2,09	1,14
ENSG00000159884	CCDC107	2,09	1,16
ENSG00000164175	SLC45A2	2,09	1,24
ENSG00000178078	STAP2	2,09	1,29
ENSG00000198668	CALM1	2,09	1,37
ENSG00000157823	AP3S2	2,09	1,28
ENSG00000182220	ATP6AP2	2,09	1,28
ENSG00000143891	GALM	2,09	1,65
ENSG00000255713	OR4D2	2,09	1,19
ENSG00000126777	KTN1	2,09	1,30
ENSG00000135829	DHX9	2,09	1,21
ENSG00000146926	ASB10	2,09	1,12
ENSG00000132294	EFR3A	2,09	1,18
ENSG00000100416	TRMU	2,09	1,16
ENSG00000103067	ESRP2	2,09	1,30
ENSG00000155975	VPS37A	2,09	1,28
ENSG00000204950	LRRC10B	2,09	1,13
ENSG00000166455	C16orf46	2,08	1,19
ENSG00000174611	KY	2,08	1,25
ENSG00000171970	ZNF57	2,08	1,29
ENSG00000125903	DEFB129	2,08	1,09
ENSG00000092010	PSME1	2,08	1,21
ENSG00000107164	FUBP3	2,08	1,14
ENSG00000139433	GLTP	2,08	1,18
ENSG00000145217	SLC26A1	2,08	1,18
ENSG00000100987	VSX1	2,08	1,18
ENSG00000138592	USP8	2,08	1,23
ENSG00000279804	PRAMEF18	2,08	1,23
ENSG00000130826	DKC1	2,08	1,25
ENSG00000197061	HIST1H4C	2,08	1,37
ENSG00000169189	NSMCE1	2,08	1,37
ENSG00000106546	AHR	2,08	2,14
ENSG00000183668	PSG9	2,08	1,21
ENSG00000167588	GPD1	2,08	1,16
ENSG00000138678	GPAT3	2,08	1,28
ENSG00000111145	ELK3	2,08	1,37
ENSG00000065060	UHRF1BP1	2,08	1,25
ENSG00000108010	GLRX3	2,08	1,23
ENSG00000215114	UBXN2B	2,08	1,30
ENSG00000172742	OR4D9	2,08	1,13
ENSG00000102125	TAZ	2,08	1,19

ensembl gene	gene symbol	Score(d)	Fold Change
ENSG00000259956	RBM15B	2,08	1,13
ENSG00000156853	ZNF689	2,08	1,21
ENSG00000196950	SLC39A10	2,08	1,34
ENSG00000247595	SPTY2D1-AS1	2,07	1,19
ENSG00000149600	COMMD7	2,07	1,21
ENSG00000120925	RNF170	2,07	1,21
ENSG00000071051	NCK2	2,07	1,30
ENSG00000117151	CTBS	2,07	1,30
ENSG00000133636	NTS	2,07	1,12
ENSG00000159208	CIART	2,07	1,61
ENSG00000174227	PIGG	2,07	1,28
ENSG00000107819	SFXN3	2,07	1,28
ENSG00000139684	ESD	2,07	1,32
ENSG00000174599	TRAM1L1	2,07	1,17
ENSG00000134759	ELP2	2,07	1,28
ENSG00000170775	GPR37	2,07	1,44
ENSG00000137252	HCRTR2	2,07	1,12
ENSG00000143162	CREG1	2,07	1,32
ENSG00000130544	ZNF557	2,07	1,21
ENSG00000131773	KHDRBS3	2,07	1,55
ENSG00000184032	KRTAP20-2	2,07	1,14
ENSG00000181585	TMIE	2,07	1,12
ENSG00000175213	ZNF408	2,06	1,24
ENSG00000134987	WDR36	2,06	1,21
ENSG00000189266	PNRC2	2,06	1,14
ENSG00000133328	HRASLS2	2,06	1,86
ENSG00000050130	JKAMP	2,06	1,23
ENSG00000176194	CIDEA	2,06	1,10
ENSG00000074695	LMAN1	2,06	1,22
ENSG00000117601	SERPINC1	2,06	1,12
ENSG00000122482	ZNF644	2,06	1,28
ENSG00000125746	EML2	2,06	1,21
ENSG00000197566	ZNF624	2,06	1,23
ENSG00000156127	BATF	2,06	1,18
ENSG00000167617	CDC42EP5	2,06	1,15
ENSG00000109610	SOD3	2,06	1,22
ENSG00000172197	MBOAT1	2,06	1,67
ENSG00000166349	RAG1	2,06	1,11
ENSG00000118181	RPS25	2,06	1,32
ENSG00000141971	MVB12A	2,06	1,27
ENSG00000155096	AZIN1	2,06	1,28
ENSG00000174748	RPL15	2,06	1,21
ENSG00000073050	XRCC1	2,05	1,20
ENSG00000102879	CORO1A	2,05	1,37
ENSG00000140931	CMTM3	2,05	1,55
ENSG00000204070	SYS1	2,05	1,26
ENSG00000177156	TALDO1	2,05	1,24
ENSG00000114268	PFKFB4	2,05	1,18
ENSG00000223638	RFPL4A	2,05	1,15

ensembl gene	gene symbol	Score(d)	Fold Change
ENSG00000187969	ZCCHC13	2,05	1,14
ENSG00000167566	NCKAP5L	2,05	1,16
ENSG00000149532	CPSF7	2,05	1,28
ENSG00000120705	ETF1	2,05	1,19
ENSG00000185028	LRRC14B	2,05	1,11
ENSG00000003096	KLHL13	2,05	1,21
ENSG00000258366	RTEL1	2,05	1,14
ENSG00000132591	ERAL1	2,05	1,28
ENSG00000155363	MOV10	2,04	1,43
ENSG00000077454	LRCH4	2,04	1,23
ENSG00000163071	SPATA18	2,04	1,23
ENSG00000237693	IRGM	2,04	1,31
ENSG00000100191	SLC5A4	2,04	1,14
ENSG00000005486	RHBDD2	2,04	1,22
ENSG00000101448	EPPIN	2,04	1,25
ENSG00000170075	GPR37L1	2,04	1,12
ENSG00000111615	KRR1	2,04	1,27
ENSG00000165322	ARHGAP12	2,04	1,31
ENSG00000164440	TXLNB	2,04	1,46
ENSG00000197826	C4orf22	2,04	1,19
ENSG00000125845	BMP2	2,04	1,17
ENSG00000105643	ARRDC2	2,03	1,33
ENSG00000163602	RYBP	2,03	1,31
ENSG00000131668	BARX1	2,03	1,13
ENSG00000102189	EEA1	2,03	1,38
ENSG00000239282	GATSL3	2,03	1,42
ENSG00000100077	GRK3	2,03	1,44
ENSG00000112365	ZBTB24	2,03	1,24
ENSG00000167822	OR8J3	2,03	1,11
ENSG00000134330	IAH1	2,03	1,18
ENSG00000135968	GCC2	2,03	1,23
ENSG00000110367	DDX6	2,03	1,19
ENSG00000111058	ACSS3	2,03	1,44
ENSG00000116161	CACYBP	2,03	1,22
ENSG00000140937	CDH11	2,03	1,18
ENSG00000111701	APOBEC1	2,03	1,13
ENSG00000110880	CORO1C	2,03	1,37
ENSG00000169359	SLC33A1	2,03	1,35
ENSG00000162039	MEIOB	2,03	1,12
ENSG00000162931	TRIM17	2,03	1,15
ENSG00000243156	MICAL3	2,02	1,30
ENSG00000082701	GSK3B	2,02	1,18
ENSG00000068885	IFT80	2,02	1,38
ENSG00000174886	NDUFA11	2,02	1,19
ENSG00000074935	TUBE1	2,02	1,28
ENSG00000149582	TMEM25	2,02	1,22
ENSG00000205208	C4orf46	2,02	1,22
ENSG00000080573	COL5A3	2,02	1,15
ENSG00000188766	SPRED3	2,02	1,20

ensembl gene	gene symbol	Score(d)	Fold Change
ENSG00000152495	CAMK4	2,02	1,60
ENSG00000093010	COMT	2,02	1,22
ENSG00000105819	PMPCB	2,02	1,17
ENSG00000181085	MAPK15	2,02	1,13
ENSG00000198589	LRBA	2,02	1,23
ENSG00000162771	FAM71A	2,02	1,37
ENSG00000253250	C8orf88	2,02	1,57
ENSG00000124406	ATP8A1	2,02	1,53
ENSG00000213397	HAUS7	2,02	1,71
ENSG00000196588	MKL1	2,02	1,25
ENSG00000167395	ZNF646	2,02	1,18
ENSG00000015676	NUDCD3	2,02	1,29
ENSG00000197045	GMFB	2,02	1,33
ENSG00000077514	POLD3	2,02	1,43
ENSG00000180138	CSNK1A1L	2,02	1,11
ENSG00000073060	SCARB1	2,01	1,48
ENSG00000167910	CYP7A1	2,01	1,12
ENSG00000182447	OTOL1	2,01	1,14
ENSG00000162882	HAAO	2,01	1,22
ENSG00000130939	UBE4B	2,01	1,18
ENSG00000097033	SH3GLB1	2,01	1,28
ENSG00000168875	SOX14	2,01	1,10
ENSG00000131051	RBM39	2,01	1,21
ENSG00000128789	PSMG2	2,01	1,20
ENSG00000231068	KRTAP21-3	2,01	1,14
ENSG00000197747	S100A10	2,01	1,75
ENSG00000057663	ATG5	2,01	1,19
ENSG00000123080	CDKN2C	2,01	1,80
ENSG00000018869	ZNF582	2,01	1,26
ENSG00000187581	COX8C	2,01	1,11
ENSG00000078795	PKD2L2	2,01	1,29
ENSG00000133687	TMTC1	2,01	1,63
ENSG00000164871	SPAG11B	2,01	1,24
ENSG00000101400	SNTA1	2,01	1,18
ENSG00000061455	PRDM6	2,01	1,21
ENSG00000102312	PORCN	2,01	1,22
ENSG00000041357	PSMA4	2,01	1,39
ENSG00000067082	KLF6	2,01	1,40
ENSG00000018408	WWTR1	2,01	1,26
ENSG00000117616	RSRP1	2,01	1,29
ENSG00000007216	SLC13A2	2,01	1,09
ENSG00000183665	TRMT12	2,00	1,20
ENSG00000253506	NACA2	2,00	1,13
ENSG00000204130	RUFY2	2,00	1,20
ENSG00000114573	ATP6V1A	2,00	1,29
ENSG00000170855	TRIAP1	2,00	1,25
ENSG00000204642	HLA-F	2,00	1,20
ENSG00000198807	PAX9	2,00	1,14
ENSG00000169297	NR0B1	2,00	1,13

ensembl gene	gene symbol	Score(d)	Fold Change
ENSG00000205730	ITPRIPL2	2,00	1,21
ENSG00000175946	KLHL38	2,00	1,10
ENSG00000106682	EIF4H	2,00	1,30
ENSG00000131055	COX4I2	2,00	1,16
ENSG00000096060	FKBP5	2,00	1,54
ENSG00000092964	DPYSL2	2,00	1,81
ENSG00000106483	SFRP4	2,00	1,14
ENSG00000133731	IMPA1	2,00	1,22
ENSG00000100889	PCK2	2,00	1,49
ENSG00000273899	NOL12	2,00	1,31
ENSG00000106268	NUDT1	2,00	1,16
ENSG00000162368	CMPK1	2,00	1,21
ENSG00000130303	BST2	2,00	1,41
ENSG00000142279	WTIP	2,00	1,09
ENSG00000183092	BEGAIN	1,99	1,22
ENSG00000110436	SLC1A2	1,99	1,11
ENSG00000155508	CNOT8	1,99	1,21
ENSG00000164535	DAGLB	1,99	1,27
ENSG00000137959	IFI44L	1,99	2,36
ENSG00000171603	CLSTN1	1,99	1,19
ENSG00000163629	PTPN13	1,99	1,85
ENSG00000022840	RNF10	1,99	1,21
ENSG00000243130	PSG11	1,99	1,13
ENSG00000164411	GJB7	1,99	1,13
ENSG00000115361	ACADL	1,99	1,16
ENSG00000205795	CYS1	1,99	1,17
ENSG00000167311	ART5	1,99	1,16
ENSG00000110330	BIRC2	1,99	1,31
ENSG00000177707	NECTIN3	1,99	1,69
ENSG00000184743	ATL3	1,99	1,19
ENSG00000167244	IGF2	1,99	1,22
ENSG00000100410	PHF5A	1,99	1,21
ENSG00000166090	IL25	1,99	1,13
ENSG00000160352	ZNF714	1,99	1,33
ENSG00000160654	CD3G	1,99	1,13
ENSG00000204022	LIPJ	1,99	1,35
ENSG00000169021	UQCRFS1	1,99	1,15
ENSG00000205867	KRTAP5-2	1,99	1,17
ENSG00000119446	RBM18	1,99	1,21
ENSG00000139899	CBLN3	1,99	1,23
ENSG00000175087	PDIK1L	1,99	1,23
ENSG00000248487	ABHD14A	1,99	1,20
ENSG00000082458	DLG3	1,99	1,36
ENSG00000145293	ENOPH1	1,99	1,19
ENSG00000164182	NDUFAF2	1,99	1,26
ENSG00000196453	ZNF777	1,98	1,13
ENSG00000099203	TMED1	1,98	1,14
ENSG00000102055	PPP1R2P9	1,98	1,09
ENSG00000112462	OR12D3	1,98	1,07

ensembl gene	gene symbol	Score(d)	Fold Change
ENSG00000167360	OR51Q1	1,98	1,11
ENSG00000173705	SUSD5	1,98	1,14
ENSG00000206013	IFITM5	1,98	1,12
ENSG00000173442	EHBP1L1	1,98	1,27
ENSG00000158270	COLEC12	1,98	1,36
ENSG00000139645	ANKRD52	1,98	1,18
ENSG00000104892	KLC3	1,98	1,25
ENSG00000121964	GTDC1	1,98	1,41
ENSG00000119844	AFTPH	1,98	1,15
ENSG00000164816	DEFA5	1,98	1,13
ENSG00000168071	CCDC88B	1,98	1,16
ENSG00000172830	SSH3	1,98	1,30
ENSG00000101230	ISM1	1,98	1,11
ENSG00000065621	GSTO2	1,98	1,35
ENSG00000263429	LINC00675	1,98	1,13
ENSG00000164402	42986	1,98	1,30
ENSG00000152234	ATP5A1	1,98	1,23
ENSG00000186146	DEFB131	1,98	1,29
ENSG00000262246	CORO7	1,98	1,25
ENSG00000109471	IL2	1,98	1,10
ENSG00000188585	LINC00083	1,98	1,19
ENSG00000138684	IL21	1,98	1,08
ENSG00000185803	SLC52A2	1,98	1,23
ENSG00000104889	RNASEH2A	1,98	1,28
ENSG00000156535	CD109	1,98	1,53
ENSG00000120008	WDR11	1,97	1,33
ENSG00000221859	KRTAP10-10	1,97	1,15
ENSG00000153207	AHCTF1	1,97	1,24
ENSG00000151611	MMAA	1,97	1,21
ENSG00000138760	SCARB2	1,97	1,30
ENSG00000164645	C7orf62	1,97	1,12
ENSG00000150753	CCT5	1,97	1,44
ENSG00000184735	DDX53	1,97	1,31
ENSG00000138185	ENTPD1	1,97	2,50
ENSG00000112149	CD83	1,97	1,53
ENSG00000069974	RAB27A	1,97	1,42
ENSG00000101200	AVP	1,97	1,11
ENSG00000179796	LRRC3B	1,97	1,17
ENSG00000197170	PSMD12	1,97	1,19
ENSG00000198054	DSCR8	1,97	1,89
ENSG00000114857	NKTR	1,97	1,17
ENSG00000115350	POLE4	1,97	1,21
ENSG00000143368	SF3B4	1,96	1,24
ENSG00000203760	CENPW	1,96	1,35
ENSG00000162378	ZYG11B	1,96	1,23
ENSG00000179593	ALOX15B	1,96	1,11
ENSG00000171302	CANT1	1,96	1,14
ENSG00000145113	MUC4	1,96	1,29
ENSG00000204922	UQCC3	1,96	1,13

ensembl gene	gene symbol	Score(d)	Fold Change
ENSG00000143952	VPS54	1,96	1,23
ENSG00000120860	CCDC53	1,96	1,23
ENSG00000148204	CRB2	1,96	1,12
ENSG00000255398	HCAR3	1,96	1,15
ENSG00000169583	CLIC3	1,96	1,11
ENSG00000198182	ZNF607	1,96	1,29
ENSG00000085433	WDR47	1,96	1,44
ENSG00000278889	OR2S2	1,96	1,14
ENSG00000143106	PSMA5	1,96	1,25
ENSG00000183378	OVCH2	1,95	1,11
ENSG00000125482	TTF1	1,95	1,21
ENSG00000013583	HEBP1	1,95	1,28
ENSG00000261857	MIA	1,95	1,63
ENSG00000100906	NFKBIA	1,95	1,46
ENSG00000169131	ZNF354A	1,95	1,26
ENSG00000004766	VPS50	1,95	1,17
ENSG00000188694	KRTAP24-1	1,95	1,11
ENSG00000151422	FER	1,95	1,32
ENSG00000172724	CCL19	1,95	1,17
ENSG00000249222	ATP5L2	1,95	1,19
ENSG00000155368	DBI	1,95	1,29
ENSG00000147434	CHRNA6	1,95	1,23
ENSG00000107731	UNC5B	1,95	1,22
ENSG00000161671	EMC10	1,95	1,21
ENSG00000041988	THAP3	1,95	1,23
ENSG00000100865	CINP	1,95	1,28
ENSG00000186603	HPDL	1,95	1,20
ENSG00000198492	YTHDF2	1,95	1,16
ENSG00000105671	DDX49	1,95	1,19
ENSG00000079156	OSBPL6	1,95	1,41
ENSG00000262628	OR1D5	1,95	1,22
ENSG00000159763	PIP	1,94	1,10
ENSG00000126778	SIX1	1,94	1,20
ENSG00000176979	TRIM60	1,94	1,11
ENSG00000054654	SYNE2	1,94	1,50
ENSG00000147145	LPAR4	1,94	1,17
ENSG00000117152	RGS4	1,94	1,16
ENSG00000173825	TIGD3	1,94	1,11
ENSG00000159182	PRAC1	1,94	1,14
ENSG00000185728	YTHDF3	1,94	1,16
ENSG00000141994	DUS3L	1,94	1,18
ENSG00000154227	CERS3	1,94	1,16
ENSG00000157181	C1orf27	1,94	1,26
ENSG00000120694	HSPH1	1,94	1,33
ENSG00000134644	PUM1	1,94	1,18
ENSG00000129675	ARHGEF6	1,94	1,60
ENSG00000205445	KRTAP10-2	1,94	1,14
ENSG00000163440	PDCL2	1,94	1,24
ENSG00000198040	ZNF84	1,94	1,36

ensembl gene	gene symbol	Score(d)	Fold Change
ENSG00000012124	CD22	1,94	1,28
ENSG00000135336	ORC3	1,94	1,24
ENSG00000137843	PAK6	1,94	1,44
ENSG00000151962	RBM46	1,94	1,13
ENSG00000184863	RBM33	1,93	1,19
ENSG00000163374	YY1AP1	1,93	1,32
ENSG00000182903	ZNF721	1,93	1,40
ENSG00000214787	MS4A4E	1,93	1,37
ENSG00000255009	UBTFL1	1,93	1,22
ENSG00000115844	DLX2	1,93	1,13
ENSG00000174915	PTDSS2	1,93	1,30
ENSG00000188820	FAM26F	1,93	1,79
ENSG00000178385	PLEKHM3	1,93	1,20
ENSG00000076043	REXO2	1,93	1,45
ENSG00000198858	R3HDM4	1,93	1,15
ENSG00000223443	USP17L2	1,93	1,13
ENSG00000146872	TLK2	1,93	1,20
ENSG00000169955	ZNF747	1,93	1,31
ENSG00000143933	CALM2	1,93	1,29
ENSG00000187140	FOXD3	1,93	1,12
ENSG00000147168	IL2RG	1,93	1,36
ENSG00000107796	ACTA2	1,92	1,28
ENSG00000074803	SLC12A1	1,92	1,19
ENSG00000125816	NKX2-4	1,92	1,14
ENSG00000174521	TTC9B	1,92	1,09
ENSG00000198754	OXCT2	1,92	1,75
ENSG00000261649	GOLGA6L7P	1,92	1,31
ENSG00000162772	ATF3	1,92	1,63
ENSG00000005889	ZFX	1,92	1,18
ENSG00000166439	RNF169	1,92	1,30
ENSG00000136630	HLX	1,92	1,32
ENSG00000198601	OR2M2	1,92	1,12
ENSG00000164687	FABP5	1,92	1,36
ENSG00000108854	SMURF2	1,92	1,23
ENSG00000138107	ACTR1A	1,92	1,23
ENSG00000008517	IL32	1,92	1,29
ENSG00000115317	HTRA2	1,92	1,26
ENSG00000115956	PLEK	1,92	1,75
ENSG00000204118	NAP1L6	1,92	1,12
ENSG00000175416	CLTB	1,92	1,21
ENSG00000162761	LMX1A	1,92	1,12
ENSG00000136052	SLC41A2	1,92	1,29
ENSG00000179981	TSHZ1	1,92	1,23
ENSG00000155792	DEPTOR	1,92	1,75
ENSG00000232125	DYTN	1,92	1,30
ENSG00000183060	LYSMD4	1,92	1,15
ENSG00000134253	TRIM45	1,92	1,19
ENSG00000075234	TTC38	1,92	1,27
ENSG00000171495	MROH2B	1,91	1,10

ensembl gene	gene symbol	Score(d)	Fold Change
ENSG00000171408	PDE7B	1,91	1,12
ENSG00000132386	SERPINF1	1,91	1,25
ENSG00000047249	ATP6V1H	1,91	1,29
ENSG00000127377	CRYGN	1,91	1,10
ENSG00000197555	SIPA1L1	1,91	1,34
ENSG00000196156	KRTAP4-3	1,91	1,13
ENSG00000101849	TBL1X	1,91	1,23
ENSG00000170873	MTSS1	1,91	1,73
ENSG00000133488	SEC14L4	1,91	1,15
ENSG00000139410	SDSL	1,91	1,19
ENSG00000143340	FAM163A	1,91	1,10
ENSG00000253368	TRNP1	1,91	1,17
ENSG00000148803	FUOM	1,91	1,38
ENSG00000183496	MEX3B	1,91	1,16
ENSG00000127884	ECHS1	1,91	1,20
ENSG00000280260	AC017028.1	1,90	1,14
ENSG00000094796	KRT31	1,90	1,13
ENSG00000196935	SRGAP1	1,90	1,32
ENSG00000188152	NUTM2G	1,90	1,16
ENSG00000117016	RIMS3	1,90	1,12
ENSG00000104499	GML	1,90	1,16
ENSG00000110628	SLC22A18	1,90	1,22
ENSG00000188243	COMMD6	1,90	1,48
ENSG00000100350	FOXRED2	1,90	1,31
ENSG00000100604	CHGA	1,90	1,18
ENSG00000197579	TOPORS	1,90	1,18
ENSG00000197971	MBP	1,90	1,18
ENSG00000165102	HGSNAT	1,90	1,31
ENSG00000130402	ACTN4	1,90	1,24
ENSG00000180628	PCGF5	1,90	1,29
ENSG00000036054	TBC1D23	1,90	1,32
ENSG00000203785	SPRR2E	1,90	1,09
ENSG00000228083	IFNA14	1,90	1,16
ENSG00000068724	TTC7A	1,90	1,17
ENSG00000109689	STIM2	1,90	1,22
ENSG00000175970	UNC119B	1,90	1,21
ENSG00000141580	WDR45B	1,90	1,15
ENSG00000116031	CD207	1,90	1,11
ENSG00000162706	CADM3	1,89	1,16
ENSG00000206172	HBA1	1,89	1,22
ENSG00000171126	KCNG3	1,89	1,09
ENSG00000187612	OR5W2	1,89	1,11
ENSG00000131669	NINJ1	1,89	1,27
ENSG00000175984	DENND2C	1,89	1,40
ENSG00000151729	SLC25A4	1,89	1,34
ENSG00000261236	BOP1	1,89	1,20
ENSG00000146426	TIAM2	1,89	1,48
ENSG00000142794	NBPF3	1,89	1,23
ENSG00000272573	MUSTN1	1,89	1,31

ensembl gene	gene symbol	Score(d)	Fold Change
ENSG00000180730	SHISA2	1,89	1,41
ENSG00000077235	GTF3C1	1,89	1,19
ENSG00000079387	SENP1	1,89	1,19
ENSG00000108395	TRIM37	1,89	1,19
ENSG00000108946	PRKAR1A	1,89	1,26
ENSG00000163874	ZC3H12A	1,89	1,40
ENSG00000170085	SIMC1	1,89	1,23
ENSG00000102882	MAPK3	1,89	1,18
ENSG00000183621	ZNF438	1,89	1,10
ENSG00000120709	FAM53C	1,89	1,20
ENSG00000128487	SPECC1	1,88	1,79
ENSG00000157330	C1orf158	1,88	1,13
ENSG00000100614	PPM1A	1,88	1,24
ENSG00000100918	REC8	1,88	1,28
ENSG00000183207	RUVBL2	1,88	1,28
ENSG00000184990	SIVA1	1,88	1,22
ENSG00000241224	FLJ22763	1,88	1,14
ENSG00000104823	ECH1	1,88	1,22
ENSG00000029639	TFB1M	1,88	1,31
ENSG00000115524	SF3B1	1,88	1,18
ENSG00000155744	FAM126B	1,88	1,25
ENSG00000141076	UTP4	1,88	1,27
ENSG00000196628	TCF4	-7,90	0,13
ENSG00000140382	HMG20A	-7,02	0,50
ENSG00000137814	HAUS2	-6,53	0,46
ENSG00000144645	OSBPL10	-6,38	0,11
ENSG00000033170	FUT8	-6,29	0,17
ENSG00000244734	HBB	-6,22	0,14
ENSG00000255837	TAS2R20	-6,21	0,34
ENSG00000197054	ZNF763	-6,12	0,44
ENSG00000070610	GBA2	-6,09	0,56
ENSG00000065457	ADAT1	-6,07	0,42
ENSG00000104866	PPP1R37	-6,03	0,51
ENSG00000110717	NDUFS8	-6,00	0,51
ENSG00000241106	HLA-DOB	-6,00	0,27
ENSG00000111906	HDDC2	-5,73	0,56
ENSG00000164406	LEAP2	-5,62	0,60
ENSG00000206530	CFAP44	-5,59	0,19
ENSG00000114956	DGUOK	-5,54	0,65
ENSG00000151746	BICD1	-5,48	0,53
ENSG00000154144	TBRG1	-5,45	0,56
ENSG00000106415	GLCCI1	-5,44	0,43
ENSG00000168283	BMI1	-5,40	0,49
ENSG00000212127	TAS2R14	-5,32	0,40
ENSG00000154016	GRAP	-5,28	0,53
ENSG00000198417	MT1F	-5,27	0,36
ENSG00000169397	RNASE3	-5,25	0,14
ENSG00000162739	SLAMF6	-5,25	0,15
ENSG00000212126	TAS2R50	-5,24	0,42

ensembl gene	gene symbol	Score(d)	Fold Change
ENSG00000177045	SIX5	-5,23	0,65
ENSG00000212124	TAS2R19	-5,21	0,37
ENSG00000117009	KMO	-5,21	0,21
ENSG00000139737	SLAIN1	-5,18	0,46
ENSG00000064115	TM7SF3	-5,16	0,44
ENSG00000102471	NDFIP2	-5,12	0,43
ENSG00000139190	VAMP1	-5,10	0,43
ENSG00000213024	NUP62	-5,09	0,47
ENSG00000131067	GGT7	-5,07	0,63
ENSG00000118363	SPCS2	-5,06	0,40
ENSG00000105698	USF2	-5,05	0,69
ENSG00000180953	ST20	-5,04	0,45
ENSG00000049323	LTBP1	-5,03	0,37
ENSG00000104880	ARHGEF18	-5,03	0,37
ENSG00000135185	TMEM243	-4,98	0,57
ENSG00000113068	PFDN1	-4,96	0,52
ENSG00000223501	VPS52	-4,95	0,66
ENSG00000149547	EI24	-4,94	0,54
ENSG00000177830	CHID1	-4,92	0,62
ENSG00000116005	PCYOX1	-4,90	0,55
ENSG00000167523	SPATA33	-4,88	0,51
ENSG00000149516	MS4A3	-4,88	0,26
ENSG00000113638	TTC33	-4,88	0,53
ENSG00000205707	LYRM5	-4,87	0,59
ENSG00000105205	CLC	-4,87	0,23
ENSG00000169696	ASPSCR1	-4,86	0,71
ENSG00000136694	IL36A	-4,85	0,57
ENSG00000158604	TMED4	-4,85	0,63
ENSG00000169877	AHSP	-4,85	0,31
ENSG00000091972	CD200	-4,83	0,24
ENSG00000163221	S100A12	-4,82	0,21
ENSG00000104967	NOVA2	-4,82	0,62
ENSG00000151612	ZNF827	-4,81	0,48
ENSG00000099797	TECR	-4,81	0,66
ENSG00000198429	ZNF69	-4,80	0,53
ENSG00000143297	FCRL5	-4,80	0,35
ENSG00000219545	UMAD1	-4,79	0,59
ENSG00000005381	MPO	-4,79	0,19
ENSG00000105656	ELL	-4,79	0,54
ENSG00000140400	MAN2C1	-4,76	0,57
ENSG00000135862	LAMC1	-4,75	0,39
ENSG00000213593	TMX2	-4,73	0,61
ENSG00000178104	PDE4DIP	-4,73	0,48
ENSG00000163527	STT3B	-4,72	0,66
ENSG00000163736	PPBP	-4,71	0,22
ENSG00000004534	RBM6	-4,70	0,62
ENSG00000005961	ITGA2B	-4,68	0,28
ENSG00000172915	NBEA	-4,68	0,39
ENSG00000170296	GABARAP	-4,67	0,73

ensembl gene	gene symbol	Score(d)	Fold Change
ENSG00000148358	GPR107	-4,67	0,71
ENSG00000177463	NR2C2	-4,66	0,66
ENSG00000118113	MMP8	-4,66	0,19
ENSG00000172366	MCRIP2	-4,66	0,52
ENSG00000196476	C20orf96	-4,64	0,56
ENSG00000128563	PRKRIP1	-4,64	0,73
ENSG00000213999	MEF2B	-4,63	0,72
ENSG00000074201	CLNS1A	-4,62	0,54
ENSG00000182054	IDH2	-4,61	0,53
ENSG00000175727	MLXIP	-4,61	0,63
ENSG00000130748	TMEM160	-4,61	0,55
ENSG00000118689	FOXO3	-4,60	0,44
ENSG00000160991	ORAI2	-4,59	0,65
ENSG00000153339	TRAPPC8	-4,59	0,52
ENSG00000086200	IPO11	-4,58	0,52
ENSG00000111676	ATN1	-4,58	0,67
ENSG00000137509	PRCP	-4,57	0,59
ENSG00000154146	NRGN	-4,57	0,64
ENSG00000185565	LSAMP	-4,56	0,22
ENSG00000121318	TAS2R10	-4,56	0,41
ENSG00000213347	MXD3	-4,53	0,63
ENSG00000157014	TATDN2	-4,52	0,56
ENSG00000123352	SPATS2	-4,49	0,63
ENSG00000133740	E2F5	-4,49	0,52
ENSG00000214078	CPNE1	-4,48	0,55
ENSG00000241399	CD302	-4,47	0,49
ENSG00000143222	UFC1	-4,45	0,69
ENSG00000096006	CRISP3	-4,45	0,33
ENSG00000095303	PTGS1	-4,45	0,57
ENSG00000143479	DYRK3	-4,43	0,52
ENSG00000148057	IDNK	-4,43	0,59
ENSG00000237441	RGL2	-4,40	0,59
ENSG00000177076	ACER2	-4,40	0,55
ENSG00000153936	HS2ST1	-4,40	0,59
ENSG00000149292	TTC12	-4,39	0,61
ENSG00000073605	GSDMB	-4,39	0,57
ENSG00000172716	SLFN11	-4,38	0,36
ENSG00000120833	SOCS2	-4,38	0,65
ENSG00000005020	SKAP2	-4,37	0,45
ENSG00000185305	ARL15	-4,36	0,57
ENSG00000129245	FXR2	-4,36	0,53
ENSG00000107341	UBE2R2	-4,36	0,70
ENSG00000180357	ZNF609	-4,35	0,68
ENSG00000071655	MBD3	-4,35	0,62
ENSG00000123095	BHLHE41	-4,34	0,40
ENSG00000110713	NUP98	-4,34	0,67
ENSG00000152818	UTRN	-4,34	0,49
ENSG00000134369	NAV1	-4,34	0,55
ENSG00000140396	NCOA2	-4,34	0,64

ensembl gene	gene symbol	Score(d)	Fold Change
ENSG00000154229	PRKCA	-4,33	0,30
ENSG00000106603	COA1	-4,33	0,65
ENSG00000215883	CYB5RL	-4,32	0,75
ENSG00000188916	FAM196A	-4,30	0,83
ENSG00000179833	SERTAD2	-4,30	0,53
ENSG00000064225	ST3GAL6	-4,30	0,57
ENSG00000274641	HIST1H2BO	-4,29	0,44
ENSG00000183401	CCDC159	-4,29	0,57
ENSG00000172375	C2CD2L	-4,29	0,65
ENSG00000111328	CDK2AP1	-4,28	0,55
ENSG00000139352	ASCL1	-4,28	0,72
ENSG00000259207	ITGB3	-4,27	0,31
ENSG00000136267	DGKB	-4,27	0,71
ENSG00000167644	C19orf33	-4,27	0,65
ENSG00000198900	TOP1	-4,25	0,68
ENSG00000168781	PPIP5K1	-4,25	0,55
ENSG00000198879	SFMBT2	-4,24	0,54
ENSG00000186815	TPCN1	-4,24	0,51
ENSG00000197959	DNM3	-4,24	0,58
ENSG00000129911	KLF16	-4,24	0,67
ENSG00000237172	B3GNT9	-4,24	0,56
ENSG00000123096	SSPN	-4,23	0,50
ENSG00000182158	CREB3L2	-4,23	0,64
ENSG00000119514	GALNT12	-4,23	0,51
ENSG00000241127	YAE1D1	-4,23	0,47
ENSG00000118420	UBE3D	-4,23	0,56
ENSG00000141458	NPC1	-4,22	0,56
ENSG00000197713	RPE	-4,22	0,56
ENSG00000139193	CD27	-4,21	0,19
ENSG00000120805	ARL1	-4,21	0,59
ENSG00000213934	HBG1	-4,21	0,71
ENSG00000154258	ABCA9	-4,21	0,53
ENSG00000103043	VAC14	-4,20	0,62
ENSG00000125107	CNOT1	-4,20	0,62
ENSG00000123384	LRP1	-4,19	0,36
ENSG00000075651	PLD1	-4,19	0,54
ENSG00000073756	PTGS2	-4,19	0,32
ENSG00000158042	MRPL17	-4,18	0,57
ENSG00000103018	CYB5B	-4,17	0,53
ENSG00000132535	DLG4	-4,16	0,46
ENSG00000034713	GABARAPL2	-4,16	0,59
ENSG00000102053	ZC3H12B	-4,16	0,54
ENSG00000197536	C5orf56	-4,16	0,64
ENSG00000081791	KIAA0141	-4,15	0,64
ENSG00000112893	MAN2A1	-4,15	0,58
ENSG00000185155	MIXL1	-4,15	0,39
ENSG00000136144	RCBTB1	-4,15	0,58
ENSG00000103365	GGA2	-4,14	0,67
ENSG00000057935	MTA3	-4,14	0,56

ensembl gene	gene symbol	Score(d)	Fold Change
ENSG00000196562	SULF2	-4,14	0,22
ENSG00000167113	COQ4	-4,14	0,69
ENSG00000103064	SLC7A6	-4,13	0,58
ENSG00000169926	KLF13	-4,13	0,63
ENSG00000242220	TCP10L	-4,13	0,66
ENSG00000226761	TAS2R46	-4,13	0,45
ENSG00000130347	RTN4IP1	-4,12	0,61
ENSG00000169330	KIAA1024	-4,12	0,56
ENSG00000188732	FAM221A	-4,12	0,48
ENSG00000183688	FAM101B	-4,12	0,41
ENSG00000175634	RPS6KB2	-4,11	0,68
ENSG00000102221	JADE3	-4,11	0,67
ENSG00000159199	ATP5G1	-4,11	0,50
ENSG00000178115	GOLGA8Q	-4,10	0,48
ENSG00000076108	BAZ2A	-4,10	0,63
ENSG00000184831	APOO	-4,10	0,48
ENSG00000198837	DENND4B	-4,10	0,64
ENSG00000171310	CHST11	-4,10	0,53
ENSG00000168491	CCDC110	-4,09	0,41
ENSG00000107984	DKK1	-4,09	0,19
ENSG00000063854	HAGH	-4,09	0,54
ENSG00000161911	TREML1	-4,09	0,48
ENSG00000174851	YIF1A	-4,08	0,60
ENSG00000197385	ZNF860	-4,08	0,50
ENSG00000188215	DCUN1D3	-4,07	0,60
ENSG00000134504	KCTD1	-4,07	0,35
ENSG00000172775	FAM192A	-4,07	0,69
ENSG00000145088	EAF2	-4,07	0,52
ENSG00000163171	CDC42EP3	-4,06	0,53
ENSG00000156928	MALSU1	-4,06	0,62
ENSG00000106344	RBM28	-4,06	0,61
ENSG00000169398	PTK2	-4,05	0,40
ENSG00000196220	SRGAP3	-4,05	0,63
ENSG00000133048	CHI3L1	-4,05	0,52
ENSG00000171481	OR1L3	-4,04	0,48
ENSG00000136205	TNS3	-4,04	0,41
ENSG00000205209	SCGB2B2	-4,04	0,66
ENSG00000139174	PRICKLE1	-4,04	0,57
ENSG00000115828	QPCT	-4,04	0,17
ENSG00000198270	TMEM116	-4,03	0,66
ENSG00000156575	PRG3	-4,03	0,65
ENSG00000155506	LARP1	-4,03	0,76
ENSG00000163626	COX18	-4,02	0,65
ENSG00000130749	ZC3H4	-4,02	0,63
ENSG00000175106	TVP23C	-4,02	0,54
ENSG00000185219	ZNF445	-4,01	0,65
ENSG00000168243	GNG4	-4,01	0,75
ENSG00000159063	ALG8	-4,01	0,68
ENSG00000164180	TMEM161B	-4,01	0,64

ensembl gene	gene symbol	Score(d)	Fold Change
ENSG00000079215	SLC1A3	-3,99	0,73
ENSG00000137726	FXYD6	-3,99	0,70
ENSG00000169385	RNASE2	-3,99	0,27
ENSG00000279342	AP000866.1	-3,98	0,54
ENSG00000137338	PGBD1	-3,98	0,70
ENSG00000142538	PTH2	-3,98	0,79
ENSG00000146021	KLHL3	-3,98	0,55
ENSG00000124256	ZBP1	-3,98	0,54
ENSG00000067141	NEO1	-3,98	0,48
ENSG00000119801	YPEL5	-3,97	0,73
ENSG00000119777	TMEM214	-3,96	0,74
ENSG00000136193	SCRN1	-3,96	0,47
ENSG00000166341	DCHS1	-3,96	0,78
ENSG00000101290	CDS2	-3,96	0,61
ENSG00000125703	ATG4C	-3,95	0,50
ENSG00000145335	SNCA	-3,95	0,46
ENSG00000099326	MZF1	-3,95	0,65
ENSG00000070614	NDST1	-3,94	0,77
ENSG00000244405	ETV5	-3,94	0,43
ENSG00000104774	MAN2B1	-3,94	0,57
ENSG00000139197	PEX5	-3,94	0,64
ENSG00000105223	PLD3	-3,94	0,62
ENSG00000130382	MLLT1	-3,93	0,65
ENSG00000120727	PAIP2	-3,93	0,71
ENSG00000079150	FKBP7	-3,93	0,50
ENSG00000167548	KMT2D	-3,93	0,64
ENSG00000112977	DAP	-3,92	0,64
ENSG00000011478	QPCTL	-3,92	0,64
ENSG00000177570	SAMD12	-3,92	0,48
ENSG00000174282	ZBTB4	-3,91	0,73
ENSG00000162777	DENND2D	-3,91	0,67
ENSG00000126883	NUP214	-3,91	0,81
ENSG00000157429	ZNF19	-3,90	0,73
ENSG00000196415	PRTN3	-3,89	0,55
ENSG00000185245	GP1BA	-3,88	0,72
ENSG00000137494	ANKRD42	-3,88	0,62
ENSG00000187266	EPOR	-3,88	0,74
ENSG00000283199	BC13-47488600E17	-3,88	0,59
ENSG00000122592	HOXA7	-3,88	0,76
ENSG00000198146	ZNF770	-3,87	0,65
ENSG00000086506	HBQ1	-3,87	0,77
ENSG00000176182	MYPOP	-3,87	0,74
ENSG00000127526	SLC35E1	-3,86	0,68
ENSG00000269028	MTRNR2L12	-3,86	0,48
ENSG00000111554	MDM1	-3,85	0,61
ENSG00000125571	IL37	-3,85	0,75
ENSG00000181722	ZBTB20	-3,85	0,61
ENSG00000137845	ADAM10	-3,84	0,60
ENSG00000225830	ERCC6	-3,84	0,61

ensembl gene	gene symbol	Score(d)	Fold Change
ENSG00000179151	EDC3	-3,83	0,58
ENSG00000186283	TOR3A	-3,82	0,64
ENSG00000137198	GMPR	-3,82	0,44
ENSG00000127578	WFIKKN1	-3,82	0,84
ENSG00000154237	LRRK1	-3,81	0,48
ENSG00000165795	NDRG2	-3,81	0,30
ENSG00000077044	DGKD	-3,81	0,52
ENSG00000104957	CCDC130	-3,80	0,74
ENSG00000105173	CCNE1	-3,80	0,52
ENSG00000197561	ELANE	-3,80	0,54
ENSG00000173200	PARP15	-3,79	0,23
ENSG00000130723	PRRC2B	-3,79	0,74
ENSG00000165490	DDIAS	-3,79	0,48
ENSG00000184719	RNLS	-3,79	0,62
ENSG00000187372	PCDHB13	-3,79	0,42
ENSG00000149636	DSN1	-3,79	0,68
ENSG00000166435	XRRA1	-3,78	0,53
ENSG00000180448	ARHGAP45	-3,78	0,62
ENSG00000240764	PCDHGC5	-3,77	0,56
ENSG00000164889	SLC4A2	-3,77	0,68
ENSG00000101441	CST4	-3,76	0,68
ENSG00000141759	TXNL4A	-3,76	0,71
ENSG00000166145	SPINT1	-3,76	0,49
ENSG00000178252	WDR6	-3,76	0,63
ENSG00000011451	WIZ	-3,75	0,76
ENSG00000178904	DPY19L3	-3,75	0,66
ENSG00000182087	TMEM259	-3,74	0,70
ENSG00000151117	TMEM86A	-3,73	0,68
ENSG00000165215	CLDN3	-3,73	0,65
ENSG00000125637	PSD4	-3,72	0,72
ENSG00000166368	OR2D2	-3,72	0,47
ENSG00000249709	ZNF564	-3,72	0,59
ENSG00000109743	BST1	-3,72	0,62
ENSG00000154645	CHODL	-3,72	0,56
ENSG00000120915	EPHX2	-3,72	0,51
ENSG00000170323	FABP4	-3,72	0,47
ENSG00000177666	PNPLA2	-3,71	0,69
ENSG00000212128	TAS2R13	-3,71	0,55
ENSG00000170549	IRX1	-3,71	0,81
ENSG00000174132	FAM174A	-3,70	0,64
ENSG00000170271	FAXDC2	-3,70	0,58
ENSG00000160999	SH2B2	-3,70	0,53
ENSG00000172765	TMCC1	-3,70	0,75
ENSG00000177238	TRIM72	-3,69	0,79
ENSG00000164587	RPS14	-3,69	0,69
ENSG00000163645	ERICH6	-3,69	0,80
ENSG00000183323	CCDC125	-3,69	0,55
ENSG00000137504	CREBZF	-3,69	0,75
ENSG00000132640	BTBD3	-3,69	0,24

ensembl gene	gene symbol	Score(d)	Fold Change
ENSG00000275023	MLLT6	-3,68	0,70
ENSG00000184363	PKP3	-3,68	0,77
ENSG00000137634	NXPE4	-3,68	0,22
ENSG00000107077	KDM4C	-3,68	0,67
ENSG00000164142	FAM160A1	-3,68	0,33
ENSG00000157680	DGKI	-3,67	0,49
ENSG00000107290	SETX	-3,67	0,65
ENSG00000126464	PRR12	-3,67	0,75
ENSG00000169814	BTD	-3,66	0,71
ENSG00000138617	PARP16	-3,66	0,63
ENSG00000125995	ROMO1	-3,66	0,69
ENSG00000100320	RBFOX2	-3,65	0,57
ENSG00000242616	GNG10	-3,65	0,61
ENSG00000149131	SERPING1	-3,65	0,38
ENSG00000165140	FBP1	-3,65	0,34
ENSG00000101162	TUBB1	-3,65	0,51
ENSG00000135439	AGAP2	-3,64	0,65
ENSG00000240771	ARHGEF25	-3,64	0,63
ENSG00000073849	ST6GAL1	-3,64	0,63
ENSG00000103037	SETD6	-3,64	0,72
ENSG00000072134	EPN2	-3,64	0,63
ENSG00000104361	NIPAL2	-3,64	0,52
ENSG00000117090	SLAMF1	-3,64	0,21
ENSG00000134121	CHL1	-3,64	0,71
ENSG00000104341	LAPTM4B	-3,64	0,26
ENSG00000145708	CRHBP	-3,64	0,46
ENSG00000176788	BASP1	-3,63	0,28
ENSG00000142494	SLC47A1	-3,63	0,18
ENSG00000105738	SIPA1L3	-3,63	0,64
ENSG00000198105	ZNF248	-3,63	0,64
ENSG00000012061	ERCC1	-3,62	0,69
ENSG00000250803	CTC-441N14.4	-3,62	0,68
ENSG00000257335	MGAM	-3,62	0,49
ENSG00000171044	XKR6	-3,61	0,51
ENSG00000186765	FSCN2	-3,61	0,82
ENSG00000070366	SMG6	-3,60	0,70
ENSG00000006015	C19orf60	-3,60	0,72
ENSG00000049860	HEXB	-3,60	0,67
ENSG00000197233	OR1J2	-3,60	0,58
ENSG00000121053	EPX	-3,59	0,43
ENSG00000165702	GFI1B	-3,59	0,79
ENSG00000197594	ENPP1	-3,58	0,38
ENSG00000262209	PCDHGB3	-3,58	0,53
ENSG00000184330	S100A7A	-3,58	0,70
ENSG00000186487	MYT1L	-3,58	0,72
ENSG00000088053	GP6	-3,58	0,81
ENSG00000120327	PCDHB14	-3,58	0,37
ENSG00000104881	PPP1R13L	-3,57	0,75
ENSG00000136868	SLC31A1	-3,57	0,66

ensembl gene	gene symbol	Score(d)	Fold Change
ENSG00000113595	TRIM23	-3,57	0,72
ENSG00000074370	ATP2A3	-3,57	0,55
ENSG00000168300	PCMTD1	-3,57	0,69
ENSG00000197530	MIB2	-3,57	0,77
ENSG00000154760	SLFN13	-3,57	0,57
ENSG00000107779	BMPR1A	-3,57	0,32
ENSG00000072736	NFATC3	-3,57	0,64
ENSG00000111424	VDR	-3,56	0,57
ENSG00000215455	KRTAP10-1	-3,56	0,71
ENSG00000102897	LYRM1	-3,56	0,69
ENSG00000181751	C5orf30	-3,55	0,63
ENSG00000145779	TNFAIP8	-3,55	0,54
ENSG00000119042	SATB2	-3,55	0,60
ENSG00000186081	KRT5	-3,55	0,65
ENSG00000163554	SPTA1	-3,55	0,69
ENSG00000171840	NINJ2	-3,55	0,70
ENSG00000076662	ICAM3	-3,55	0,60
ENSG00000171720	HDAC3	-3,54	0,71
ENSG00000253485	PCDHGA5	-3,54	0,61
ENSG00000102760	RGCC	-3,54	0,25
ENSG00000121741	ZMYM2	-3,54	0,66
ENSG00000197774	EME2	-3,54	0,82
ENSG00000226763	SRRM5	-3,54	0,75
ENSG00000159388	BTG2	-3,54	0,66
ENSG00000114529	C3orf52	-3,54	0,59
ENSG00000275385	CCL18	-3,54	0,78
ENSG00000163083	INHBB	-3,54	0,84
ENSG00000179222	MAGED1	-3,53	0,66
ENSG00000183091	NEB	-3,53	0,27
ENSG00000135503	ACVR1B	-3,53	0,64
ENSG00000113456	RAD1	-3,53	0,76
ENSG00000111325	OGFOD2	-3,52	0,69
ENSG00000177807	KCNJ10	-3,52	0,71
ENSG00000144959	NCEH1	-3,51	0,69
ENSG00000177354	C10orf71	-3,51	0,76
ENSG00000183778	B3GALT5	-3,51	0,70
ENSG00000131094	C1QL1	-3,51	0,69
ENSG00000142798	HSPG2	-3,50	0,70
ENSG00000197776	KLHDC1	-3,50	0,55
ENSG00000183258	DDX41	-3,50	0,71
ENSG00000160209	PDXK	-3,50	0,69
ENSG00000152926	ZNF117	-3,50	0,51
ENSG00000169223	LMAN2	-3,50	0,74
ENSG00000157350	ST3GAL2	-3,50	0,61
ENSG00000170482	SLC23A1	-3,50	0,68
ENSG00000174175	SELP	-3,50	0,54
ENSG00000107185	RGP1	-3,50	0,76
ENSG00000105662	CRTC1	-3,49	0,73
ENSG00000128000	ZNF780B	-3,49	0,49

ensembl gene	gene symbol	Score(d)	Fold Change
ENSG00000180979	LRRC57	-3,49	0,70
ENSG00000166603	MC4R	-3,49	0,25
ENSG00000130702	LAMA5	-3,48	0,53
ENSG00000185697	MYBL1	-3,48	0,23
ENSG00000250722	SEPP1	-3,48	0,50
ENSG00000142507	PSMB6	-3,48	0,64
ENSG00000069966	GNB5	-3,48	0,63
ENSG00000021461	CYP3A43	-3,48	0,42
ENSG00000172380	GNG12	-3,48	0,57
ENSG00000178057	NDUFAF3	-3,48	0,68
ENSG00000178927	C17orf62	-3,48	0,68
ENSG00000178741	COX5A	-3,48	0,68
ENSG00000113761	ZNF346	-3,48	0,74
ENSG00000185345	PARK2	-3,47	0,65
ENSG00000100307	CBX7	-3,47	0,57
ENSG00000028528	SNX1	-3,47	0,74
ENSG00000028277	POU2F2	-3,47	0,57
ENSG00000079691	LRRC16A	-3,47	0,58
ENSG00000109208	SMR3A	-3,47	0,74
ENSG00000198794	SCAMP5	-3,47	0,49
ENSG00000039319	ZFYVE16	-3,46	0,63
ENSG00000147535	PLPP5	-3,46	0,71
ENSG00000225698	IGHV3-72	-3,46	0,40
ENSG00000156858	PRR14	-3,46	0,70
ENSG00000157379	DHRS1	-3,46	0,70
ENSG00000204560	DHX16	-3,45	0,77
ENSG00000116539	ASH1L	-3,45	0,62
ENSG00000061273	HDAC7	-3,45	0,75
ENSG00000185267	CDNF	-3,45	0,60
ENSG00000140262	TCF12	-3,45	0,77
ENSG00000240184	PCDHGC3	-3,44	0,52
ENSG00000172922	RNASEH2C	-3,44	0,69
ENSG00000198586	TLK1	-3,44	0,73
ENSG00000184933	OR6A2	-3,43	0,59
ENSG00000101439	CST3	-3,43	0,41
ENSG00000040933	INPP4A	-3,42	0,64
ENSG00000146963	LUC7L2	-3,42	0,75
ENSG00000081189	MEF2C	-3,42	0,69
ENSG00000143786	CNIH3	-3,42	0,67
ENSG00000088812	ATRN	-3,42	0,66
ENSG00000108312	UBTF	-3,42	0,72
ENSG00000104824	HNRNPL	-3,41	0,75
ENSG00000040633	PHF23	-3,41	0,70
ENSG00000137101	CD72	-3,41	0,69
ENSG00000148842	CNNM2	-3,41	0,64
ENSG00000100321	SYNGR1	-3,41	0,54
ENSG00000112812	PRSS16	-3,41	0,64
ENSG00000105323	HNRNPUL1	-3,41	0,78
ENSG00000103056	SMPD3	-3,41	0,64

ensembl gene	gene symbol	Score(d)	Fold Change
ENSG00000204482	LST1	-3,40	0,66
ENSG00000172819	RARG	-3,40	0,70
ENSG00000179397	C1orf101	-3,40	0,67
ENSG00000166233	ARIH1	-3,40	0,72
ENSG00000137513	NARS2	-3,40	0,67
ENSG00000164181	ELOVL7	-3,39	0,41
ENSG00000188033	ZNF490	-3,39	0,77
ENSG00000126261	UBA2	-3,39	0,74
ENSG00000253731	PCDHGA6	-3,39	0,55
ENSG00000114738	MAPKAPK3	-3,38	0,71
ENSG00000243789	JMJD7	-3,38	0,59
ENSG00000105723	GSK3A	-3,38	0,79
ENSG00000196227	FAM217B	-3,38	0,63
ENSG00000176261	ZBTB8OS	-3,37	0,66
ENSG00000133275	CSNK1G2	-3,37	0,66
ENSG00000091879	ANGPT2	-3,37	0,83
ENSG00000114544	SLC41A3	-3,37	0,73
ENSG00000166405	RIC3	-3,36	0,44
ENSG00000124767	GLO1	-3,36	0,74
ENSG00000021776	AQR	-3,36	0,77
ENSG00000188124	OR2AG2	-3,36	0,63
ENSG00000168958	MFF	-3,36	0,75
ENSG00000256222	MTRNR2L3	-3,36	0,77
ENSG00000168675	LDLRAD4	-3,36	0,67
ENSG00000174684	B4GAT1	-3,36	0,79
ENSG00000224470	ATXN1L	-3,36	0,78
ENSG00000106823	ECM2	-3,35	0,77
ENSG00000154485	MMP21	-3,35	0,83
ENSG00000165685	TMEM52B	-3,35	0,37
ENSG00000172379	ARNT2	-3,35	0,47
ENSG00000147799	ARHGAP39	-3,35	0,71
ENSG00000156642	NPTN	-3,35	0,67
ENSG00000153071	DAB2	-3,35	0,39
ENSG00000127445	PIN1	-3,35	0,79
ENSG00000126351	THRA	-3,34	0,69
ENSG00000132612	VPS4A	-3,34	0,69
ENSG00000124733	MEA1	-3,34	0,69
ENSG00000187742	SECISBP2	-3,34	0,77
ENSG00000170456	DENND5B	-3,34	0,69
ENSG00000136929	HEMGN	-3,33	0,62
ENSG00000117335	CD46	-3,33	0,67
ENSG00000206535	LNP1	-3,33	0,61
ENSG00000066382	MPPED2	-3,33	0,32
ENSG00000189056	RELN	-3,33	0,31
ENSG00000048828	FAM120A	-3,33	0,65
ENSG00000130312	MRPL34	-3,33	0,58
ENSG00000198728	LDB1	-3,33	0,74
ENSG00000137642	SORL1	-3,32	0,51
ENSG00000120451	SNX19	-3,32	0,73

ensembl gene	gene symbol	Score(d)	Fold Change
ENSG00000118137	APOA1	-3,32	0,83
ENSG00000012223	LTF	-3,32	0,45
ENSG00000104979	C19orf53	-3,31	0,79
ENSG00000149932	TMEM219	-3,31	0,79
ENSG00000167202	TBC1D2B	-3,30	0,60
ENSG00000118523	CTGF	-3,30	0,33
ENSG00000176142	TMEM39A	-3,30	0,76
ENSG00000177576	C18orf32	-3,30	0,67
ENSG00000115884	SDC1	-3,29	0,59
ENSG00000122696	SLC25A51	-3,29	0,81
ENSG00000204977	TRIM13	-3,29	0,68
ENSG00000133742	CA1	-3,29	0,57
ENSG00000140563	MCTP2	-3,29	0,59
ENSG00000138101	DTNB	-3,29	0,73
ENSG00000158473	CD1D	-3,29	0,44
ENSG00000188352	FOCAD	-3,28	0,67
ENSG00000176102	CSTF3	-3,28	0,61
ENSG00000170092	SPDYE5	-3,28	0,73
ENSG00000118690	ARMC2	-3,28	0,42
ENSG00000123358	NR4A1	-3,28	0,47
ENSG00000183145	RIPPLY3	-3,27	0,82
ENSG00000211584	SLC48A1	-3,27	0,71
ENSG00000183615	FAM167B	-3,27	0,81
ENSG00000156398	SFXN2	-3,27	0,74
ENSG00000185737	NRG3	-3,27	0,34
ENSG00000272325	NUDT3	-3,27	0,68
ENSG00000122188	LAX1	-3,27	0,43
ENSG00000125740	FOSB	-3,27	0,54
ENSG00000253767	PCDHGA8	-3,27	0,45
ENSG00000161267	BDH1	-3,27	0,53
ENSG00000171766	GATM	-3,27	0,38
ENSG00000079931	MOXD1	-3,27	0,36
ENSG00000186918	ZNF395	-3,26	0,60
ENSG00000165238	WNK2	-3,26	0,60
ENSG00000130255	RPL36	-3,26	0,70
ENSG00000198917	C9orf114	-3,26	0,62
ENSG00000253846	PCDHGA10	-3,26	0,48
ENSG00000182986	ZNF320	-3,25	0,64
ENSG00000164002	EXO5	-3,25	0,68
ENSG00000170571	EMB	-3,25	0,76
ENSG00000196865	NHLRC2	-3,25	0,72
ENSG00000096968	JAK2	-3,25	0,65
ENSG00000106771	TMEM245	-3,25	0,71
ENSG00000148943	LIN7C	-3,25	0,71
ENSG00000107242	PIP5K1B	-3,25	0,49
ENSG00000108423	TUBD1	-3,25	0,59
ENSG00000164821	DEFA4	-3,25	0,58
ENSG00000128881	TTBK2	-3,25	0,74
ENSG00000148229	POLE3	-3,25	0,80

ensembl gene	gene symbol	Score(d)	Fold Change
ENSG00000173914	RBM4B	-3,25	0,68
ENSG00000182870	GALNT9	-3,24	0,71
ENSG00000030582	GRN	-3,24	0,53
ENSG00000197136	PCNX3	-3,24	0,76
ENSG00000184445	KNTC1	-3,24	0,60
ENSG00000132313	MRPL35	-3,24	0,63
ENSG00000106723	SPIN1	-3,24	0,65
ENSG00000108773	KAT2A	-3,24	0,65
ENSG00000167468	GPX4	-3,23	0,72
ENSG00000204424	LY6G6F	-3,23	0,62
ENSG00000088387	DOCK9	-3,23	0,61
ENSG00000204231	RXRB	-3,23	0,78
ENSG00000198881	ASB12	-3,23	0,78
ENSG00000166471	TMEM41B	-3,23	0,75
ENSG00000152422	XRCC4	-3,23	0,55
ENSG00000143315	PIGM	-3,23	0,63
ENSG00000165185	KIAA1958	-3,23	0,71
ENSG00000124588	NQO2	-3,23	0,62
ENSG00000135218	CD36	-3,23	0,49
ENSG00000102805	CLN5	-3,23	0,57
ENSG00000116157	GPX7	-3,23	0,69
ENSG00000082805	ERC1	-3,23	0,65
ENSG00000133678	TMEM254	-3,22	0,69
ENSG00000004939	SLC4A1	-3,22	0,61
ENSG00000253537	PCDHGA7	-3,22	0,53
ENSG00000175591	P2RY2	-3,22	0,58
ENSG00000197822	OCLN	-3,22	0,35
ENSG00000231389	HLA-DPA1	-3,22	0,43
ENSG00000213920	MDP1	-3,21	0,71
ENSG00000196188	CTSE	-3,21	0,67
ENSG00000116750	UCHL5	-3,21	0,66
ENSG00000100934	SEC23A	-3,21	0,71
ENSG00000070087	PFN2	-3,21	0,47
ENSG00000103876	FAH	-3,21	0,65
ENSG00000153815	CMIP	-3,20	0,67
ENSG00000123360	PDE1B	-3,20	0,78
ENSG00000115252	PDE1A	-3,20	0,60
ENSG00000168517	HEXIM2	-3,20	0,75
ENSG00000136937	NCBP1	-3,20	0,74
ENSG00000159322	ADPGK	-3,20	0,76
ENSG00000102763	VWA8	-3,19	0,64
ENSG00000182612	TSPAN10	-3,19	0,83
ENSG00000112984	KIF20A	-3,19	0,58
ENSG00000254999	BRK1	-3,19	0,72
ENSG00000135272	MDFIC	-3,18	0,73
ENSG00000160796	NBEAL2	-3,18	0,63
ENSG00000114487	MORC1	-3,18	0,36
ENSG00000138119	MYOF	-3,18	0,51
ENSG00000171757	LRRC34	-3,18	0,57

ensembl gene	gene symbol	Score(d)	Fold Change
ENSG00000185015	CA13	-3,18	0,53
ENSG00000129625	REEP5	-3,17	0,79
ENSG00000171357	LURAP1	-3,17	0,87
ENSG00000182492	BGN	-3,17	0,73
ENSG00000224877	C17orf89	-3,17	0,77
ENSG00000163832	ELP6	-3,17	0,75
ENSG00000171604	CXXC5	-3,17	0,71
ENSG00000072954	TMEM38A	-3,17	0,52
ENSG00000148290	SURF1	-3,17	0,74
ENSG00000255823	MTRNR2L8	-3,17	0,80
ENSG00000050820	BCAR1	-3,16	0,72
ENSG00000166167	BTRC	-3,16	0,73
ENSG00000075568	TMEM131	-3,16	0,73
ENSG00000175699	LINC00521	-3,16	0,83
ENSG00000196329	GIMAP5	-3,16	0,54
ENSG00000122176	FMOD	-3,15	0,79
ENSG00000176978	DPP7	-3,15	0,67
ENSG00000011243	AKAP8L	-3,15	0,81
ENSG00000172728	FUT10	-3,15	0,70
ENSG00000204060	FOXO6	-3,15	0,78
ENSG00000196735	HLA-DQA1	-3,15	0,48
ENSG00000196290	NIF3L1	-3,14	0,67
ENSG00000181704	YIPF6	-3,14	0,74
ENSG00000103512	NOMO1	-3,14	0,59
ENSG00000105767	CADM4	-3,14	0,44
ENSG00000197429	IPP	-3,14	0,55
ENSG00000204149	AGAP6	-3,14	0,76
ENSG00000008438	PGLYRP1	-3,14	0,61
ENSG00000213865	C8orf44	-3,14	0,65
ENSG00000188938	FAM120AOS	-3,14	0,76
ENSG00000204682	CASC10	-3,14	0,84
ENSG00000173120	KDM2A	-3,14	0,72
ENSG00000198732	SMOC1	-3,13	0,44
ENSG00000144619	CNTN4	-3,13	0,63
ENSG00000167110	GOLGA2	-3,13	0,76
ENSG00000100985	MMP9	-3,13	0,48
ENSG00000091010	POU4F3	-3,13	0,84
ENSG00000066468	FGFR2	-3,13	0,74
ENSG00000073754	CD5L	-3,13	0,53
ENSG00000239264	TXNDC5	-3,13	0,59
ENSG00000152700	SAR1B	-3,13	0,75
ENSG00000198478	SH3BGRL2	-3,13	0,46
ENSG00000170122	FOXD4	-3,12	0,57
ENSG00000104660	LEPROTL1	-3,12	0,72
ENSG00000078967	UBE2D4	-3,12	0,73
ENSG00000164620	RELL2	-3,12	0,73
ENSG00000120738	EGR1	-3,12	0,44
ENSG00000126545	CSN1S1	-3,12	0,66
ENSG00000175390	EIF3F	-3,12	0,81

ensembl gene	gene symbol	Score(d)	Fold Change
ENSG00000198108	CHSY3	-3,11	0,36
ENSG00000169220	RGS14	-3,11	0,77
ENSG00000166762	CATSPER2	-3,11	0,69
ENSG00000105997	HOXA3	-3,11	0,77
ENSG00000108559	NUP88	-3,11	0,71
ENSG00000137070	IL11RA	-3,11	0,73
ENSG00000127364	TAS2R4	-3,10	0,70
ENSG00000179909	ZNF154	-3,10	0,53
ENSG00000164040	PGRMC2	-3,10	0,77
ENSG00000163002	NUP35	-3,10	0,76
ENSG00000162482	AKR7A3	-3,10	0,79
ENSG00000160588	MPZL3	-3,10	0,57
ENSG00000010278	CD9	-3,10	0,38
ENSG00000134326	CMPK2	-3,10	0,38
ENSG00000105726	ATP13A1	-3,10	0,71
ENSG00000253159	PCDHGA12	-3,09	0,54
ENSG00000151623	NR3C2	-3,09	0,62
ENSG00000144120	TMEM177	-3,09	0,58
ENSG00000182150	ERCC6L2	-3,09	0,70
ENSG00000198833	UBE2J1	-3,09	0,86
ENSG00000048462	TNFRSF17	-3,09	0,59
ENSG00000169871	TRIM56	-3,08	0,67
ENSG00000243927	MRPS6	-3,08	0,72
ENSG00000139329	LUM	-3,08	0,65
ENSG00000188581	KRTAP1-1	-3,08	0,81
ENSG00000241233	KRTAP5-8	-3,08	0,74
ENSG00000186088	GSAP	-3,07	0,68
ENSG00000103363	TCEB2	-3,07	0,71
ENSG00000175567	UCP2	-3,07	0,60
ENSG00000108417	KRT37	-3,07	0,82
ENSG00000181555	SETD2	-3,07	0,77
ENSG00000172139	SLC9C1	-3,07	0,61
ENSG00000162241	SLC25A45	-3,07	0,74
ENSG00000139629	GALNT6	-3,06	0,70
ENSG00000115275	MOGS	-3,06	0,73
ENSG00000144504	ANKMY1	-3,06	0,74
ENSG00000165966	PDZRN4	-3,06	0,48
ENSG00000159792	PSKH1	-3,06	0,82
ENSG00000183807	FAM162B	-3,06	0,88
ENSG00000111341	MGP	-3,05	0,66
ENSG00000172590	MRPL52	-3,05	0,76
ENSG00000138722	MMRN1	-3,05	0,48
ENSG00000127415	IDUA	-3,05	0,67
ENSG00000089006	SNX5	-3,05	0,77
ENSG00000132470	ITGB4	-3,05	0,74
ENSG00000168872	DDX19A	-3,05	0,73
ENSG00000149357	LAMTOR1	-3,05	0,77
ENSG00000187607	ZNF286A	-3,04	0,69
ENSG00000204361	NXPE2	-3,04	0,71

ensembl gene	gene symbol	Score(d)	Fold Change
ENSG00000101353	MROH8	-3,04	0,67
ENSG00000108840	HDAC5	-3,04	0,74
ENSG00000205464	ATP6AP1L	-3,04	0,67
ENSG00000172650	AGAP5	-3,04	0,64
ENSG00000164047	CAMP	-3,04	0,71
ENSG00000104133	SPG11	-3,04	0,69
ENSG00000150244	TRIM48	-3,04	0,75
ENSG00000254901	BORCS8	-3,04	0,65
ENSG00000276234	TADA2A	-3,04	0,66
ENSG00000099834	CDHR5	-3,03	0,84
ENSG00000213199	ASIC3	-3,03	0,77
ENSG00000115607	IL18RAP	-3,03	0,58
ENSG00000186132	C2orf76	-3,03	0,72
ENSG00000198520	C1orf228	-3,03	0,67
ENSG00000112232	KHDRBS2	-3,03	0,39
ENSG00000204420	C6orf25	-3,03	0,75
ENSG00000158825	CDA	-3,03	0,63
ENSG00000003400	CASP10	-3,03	0,64
ENSG00000185989	RASA3	-3,03	0,61
ENSG00000158856	DMTN	-3,02	0,69
ENSG00000159618	ADGRG5	-3,02	0,44
ENSG00000275074	NUDT18	-3,02	0,84
ENSG00000111785	RIC8B	-3,02	0,72
ENSG00000115380	EFEMP1	-3,02	0,32
ENSG00000106404	CLDN15	-3,02	0,77
ENSG00000084110	HAL	-3,02	0,74
ENSG00000035720	STAP1	-3,02	0,32
ENSG00000155846	PPARGC1B	-3,02	0,67
ENSG00000183723	CMTM4	-3,02	0,64
ENSG00000173578	XCR1	-3,02	0,81
ENSG00000170180	GYPA	-3,02	0,57
ENSG00000101958	GLRA2	-3,02	0,84
ENSG00000168389	MFSD2A	-3,02	0,55
ENSG00000178229	ZNF543	-3,01	0,73
ENSG00000169180	XPO6	-3,01	0,80
ENSG00000172780	RAB43	-3,01	0,71
ENSG00000233493	TMEM238	-3,01	0,65
ENSG00000124491	F13A1	-3,01	0,51
ENSG00000141956	PRDM15	-3,01	0,47
ENSG00000104691	UBXN8	-3,01	0,62
ENSG00000106066	CPVL	-3,01	0,44
ENSG00000122877	EGR2	-3,01	0,38
ENSG00000256436	TAS2R31	-3,01	0,64
ENSG00000111077	TNS2	-3,01	0,84
ENSG00000118096	IFT46	-3,01	0,66
ENSG00000197734	C14orf178	-3,01	0,82
ENSG00000133030	MPRIP	-3,01	0,73
ENSG00000130818	ZNF426	-3,00	0,58
ENSG00000136371	MTHFS	-3,00	0,75

ensembl gene	gene symbol	Score(d)	Fold Change
ENSG00000022267	FHL1	-3,00	0,38
ENSG00000084693	AGBL5	-3,00	0,71
ENSG00000187741	FANCA	-3,00	0,64
ENSG00000205155	PSENEN	-3,00	0,74
ENSG00000172992	DCAKD	-3,00	0,71
ENSG00000171097	KYAT1	-3,00	0,79
ENSG00000134215	VAV3	-3,00	0,47
ENSG00000064393	HIPK2	-2,99	0,73
ENSG00000185664	PMEL	-2,99	0,72
ENSG00000182919	C11orf54	-2,99	0,69
ENSG00000213619	NDUFS3	-2,99	0,76
ENSG00000166211	SPIC	-2,99	0,49
ENSG00000261934	PCDHGA9	-2,99	0,57
ENSG00000205476	CCDC85C	-2,99	0,82
ENSG00000277893	SRD5A2	-2,99	0,81
ENSG00000067992	PDK3	-2,99	0,66
ENSG00000173237	C11orf86	-2,99	0,87
ENSG00000187559	FOXD4L3	-2,99	0,78
ENSG00000072756	TRNT1	-2,99	0,72
ENSG00000107249	GLIS3	-2,99	0,34
ENSG00000099364	FBXL19	-2,99	0,74
ENSG00000134827	TCN1	-2,99	0,53
ENSG00000111269	CREBL2	-2,98	0,72
ENSG00000205076	LGALS7	-2,98	0,71
ENSG00000109452	INPP4B	-2,98	0,59
ENSG00000182742	HOXB4	-2,98	0,79
ENSG00000167964	RAB26	-2,98	0,76
ENSG00000130475	FCHO1	-2,98	0,72
ENSG00000127511	SIN3B	-2,98	0,79
ENSG00000068784	SRBD1	-2,98	0,71
ENSG00000114125	RNF7	-2,98	0,57
ENSG00000083814	ZNF671	-2,98	0,71
ENSG00000178802	MPI	-2,98	0,70
ENSG00000111203	ITFG2	-2,98	0,73
ENSG00000175130	MARCKSL1	-2,97	0,76
ENSG00000124181	PLCG1	-2,97	0,78
ENSG00000143819	EPHX1	-2,97	0,58
ENSG00000161010	C5orf45	-2,97	0,77
ENSG00000149294	NCAM1	-2,97	0,24
ENSG00000249948	GBA3	-2,97	0,37
ENSG00000142046	TMEM91	-2,97	0,75
ENSG00000181408	UTS2R	-2,97	0,66
ENSG00000101425	BPI	-2,97	0,56
ENSG00000145781	COMMD10	-2,96	0,75
ENSG00000177628	GBA	-2,96	0,68
ENSG00000163960	UBXN7	-2,96	0,75
ENSG00000122641	INHBA	-2,96	0,84
ENSG00000155099	TMEM55A	-2,96	0,71
ENSG00000065485	PDIA5	-2,96	0,68

ensembl gene	gene symbol	Score(d)	Fold Change
ENSG00000166317	SYNPO2L	-2,96	0,71
ENSG00000037757	MRI1	-2,96	0,66
ENSG00000163406	SLC15A2	-2,95	0,52
ENSG00000168404	MLKL	-2,95	0,55
ENSG00000094916	CBX5	-2,95	0,66
ENSG00000157554	ERG	-2,95	0,78
ENSG00000178150	ZNF114	-2,95	0,81
ENSG00000140749	IGSF6	-2,95	0,57
ENSG00000116128	BCL9	-2,95	0,63
ENSG00000132182	NUP210	-2,95	0,68
ENSG00000204713	TRIM27	-2,95	0,77
ENSG00000105372	RPS19	-2,95	0,72
ENSG00000189298	ZKSCAN3	-2,95	0,74
ENSG00000134007	ADAM20	-2,95	0,74
ENSG00000164627	KIF6	-2,95	0,60
ENSG00000113108	APBB3	-2,95	0,72
ENSG00000171388	APLN	-2,95	0,83
ENSG00000164241	C5orf63	-2,95	0,57
ENSG00000100448	CTSG	-2,95	0,60
ENSG00000186889	TMEM17	-2,94	0,68
ENSG00000258417	RP11-240B13.2	-2,94	0,64
ENSG00000116729	WLS	-2,94	0,49
ENSG00000115652	UXS1	-2,94	0,70
ENSG00000263465	SRSF8	-2,94	0,78
ENSG00000105352	CEACAM4	-2,94	0,77
ENSG00000135407	AVIL	-2,94	0,70
ENSG00000123329	ARHGAP9	-2,94	0,76
ENSG00000152582	SPEF2	-2,94	0,63
ENSG00000182022	CHST15	-2,94	0,72
ENSG00000105829	BET1	-2,94	0,59
ENSG00000171843	MLLT3	-2,94	0,59
ENSG00000128928	IVD	-2,94	0,72
ENSG00000153707	PTPRD	-2,93	0,46
ENSG00000205085	FAM71F2	-2,93	0,67
ENSG00000175893	ZDHHC21	-2,93	0,62
ENSG00000198130	HIBCH	-2,93	0,65
ENSG00000077157	PPP1R12B	-2,93	0,64
ENSG00000189091	SF3B3	-2,92	0,69
ENSG00000184148	SPRR4	-2,92	0,84
ENSG00000165202	OR1Q1	-2,92	0,56
ENSG00000162139	NEU3	-2,92	0,66
ENSG00000221970	OR2A1	-2,92	0,72
ENSG00000100365	NCF4	-2,92	0,53
ENSG00000119927	GPAM	-2,91	0,69
ENSG00000106686	SPATA6L	-2,91	0,64
ENSG00000100234	TIMP3	-2,91	0,78
ENSG00000109083	IFT20	-2,91	0,77
ENSG00000176422	SPRYD4	-2,91	0,68
ENSG00000115486	GGCX	-2,91	0,77

ensembl gene	gene symbol	Score(d)	Fold Change
ENSG00000186166	CCDC84	-2,91	0,73
ENSG00000149262	INTS4	-2,91	0,74
ENSG00000240891	PLCXD2	-2,91	0,59
ENSG00000181027	FKRP	-2,90	0,78
ENSG00000132507	EIF5A	-2,90	0,70
ENSG00000073417	PDE8A	-2,90	0,73
ENSG00000119535	CSF3R	-2,90	0,57
ENSG00000198832	SELM	-2,90	0,63
ENSG00000133424	LARGE1	-2,90	0,41
ENSG00000135443	KRT85	-2,90	0,83
ENSG00000135205	CCDC146	-2,90	0,66
ENSG00000235750	KIAA0040	-2,90	0,69
ENSG00000134138	MEIS2	-2,90	0,42
ENSG00000169118	CSNK1G1	-2,89	0,70
ENSG00000254834	OR5M10	-2,89	0,67
ENSG00000254858	MPV17L2	-2,89	0,62
ENSG00000188687	SLC4A5	-2,89	0,58
ENSG00000125734	GPR108	-2,89	0,77
ENSG00000144021	CIAO1	-2,89	0,80
ENSG00000179889	PDXDC1	-2,88	0,74
ENSG00000170345	FOS	-2,88	0,61
ENSG00000079385	CEACAM1	-2,88	0,54
ENSG00000214413	BBIP1	-2,88	0,72
ENSG00000169900	PYDC1	-2,88	0,76
ENSG00000072071	ADGRL1	-2,88	0,75
ENSG00000162694	EXTL2	-2,88	0,51
ENSG00000165209	STRBP	-2,88	0,63
ENSG00000160213	CSTB	-2,88	0,76
ENSG00000152192	POU4F1	-2,88	0,56
ENSG00000100368	CSF2RB	-2,88	0,53
ENSG00000001631	KRIT1	-2,88	0,80
ENSG00000175455	CCDC14	-2,88	0,63
ENSG00000205364	MT1M	-2,87	0,81
ENSG00000157833	GAREM2	-2,87	0,72
ENSG00000163563	MNDA	-2,87	0,52
ENSG00000183251	OR51B4	-2,87	0,73
ENSG00000164975	SNAPC3	-2,87	0,62
ENSG00000127663	KDM4B	-2,87	0,75
ENSG00000117091	CD48	-2,87	0,52
ENSG00000170819	BFSP2	-2,87	0,76
ENSG00000163348	PYGO2	-2,87	0,51
ENSG00000258839	MC1R	-2,87	0,78
ENSG00000103381	CPPED1	-2,86	0,55
ENSG00000163295	ALPI	-2,86	0,81
ENSG00000256294	ZNF225	-2,86	0,72
ENSG00000161980	POLR3K	-2,86	0,65
ENSG00000100647	SUSD6	-2,86	0,67
ENSG00000150907	FOXO1	-2,86	0,66
ENSG00000116209	TMEM59	-2,86	0,74

ensembl gene	gene symbol	Score(d)	Fold Change
ENSG00000120158	RCL1	-2,86	0,76
ENSG00000204852	TCTN1	-2,86	0,60
ENSG00000135297	MTO1	-2,86	0,70
ENSG00000100429	HDAC10	-2,86	0,50
ENSG00000115204	MPV17	-2,85	0,74
ENSG00000163785	RYK	-2,85	0,76
ENSG00000072694	FCGR2B	-2,85	0,55
ENSG00000225873	LINC00694	-2,85	0,82
ENSG00000108433	GOSR2	-2,85	0,79
ENSG00000008869	HEATR5B	-2,85	0,67
ENSG00000167264	DUS2	-2,85	0,63
ENSG00000154928	EPHB1	-2,85	0,54
ENSG00000111271	ACAD10	-2,85	0,73
ENSG00000128739	SNRPN	-2,85	0,74
ENSG00000128594	LRRC4	-2,85	0,82
ENSG00000091409	ITGA6	-2,84	0,52
ENSG00000206177	HBM	-2,84	0,75
ENSG00000115677	HDLBP	-2,84	0,81
ENSG00000175938	ORAI3	-2,84	0,75
ENSG00000105538	RASIP1	-2,84	0,82
ENSG00000167614	TTYH1	-2,84	0,83
ENSG00000147905	ZCCHC7	-2,84	0,72
ENSG00000102349	KLF8	-2,84	0,73
ENSG00000129197	RPAIN	-2,84	0,76
ENSG00000121211	MND1	-2,84	0,60
ENSG00000242550	SERPINB10	-2,84	0,64
ENSG00000111679	PTPN6	-2,84	0,61
ENSG00000243317	C7orf73	-2,84	0,77
ENSG00000167280	ENGASE	-2,84	0,71
ENSG00000111653	ING4	-2,83	0,74
ENSG00000149564	ESAM	-2,83	0,56
ENSG00000174444	RPL4	-2,83	0,79
ENSG00000171552	BCL2L1	-2,83	0,64
ENSG00000115042	FAHD2A	-2,83	0,70
ENSG00000179152	TCAIM	-2,83	0,57
ENSG00000134716	CYP2J2	-2,83	0,59
ENSG00000105771	SMG9	-2,82	0,73
ENSG00000131069	ACSS2	-2,82	0,68
ENSG00000189401	OTUD6A	-2,82	0,87
ENSG00000148400	NOTCH1	-2,82	0,73
ENSG00000183508	FAM46C	-2,82	0,70
ENSG00000176900	OR51T1	-2,82	0,88
ENSG00000081052	COL4A4	-2,82	0,78
ENSG00000253910	PCDHGB2	-2,82	0,59
ENSG00000082196	C1QTNF3	-2,82	0,64
ENSG00000160688	FLAD1	-2,82	0,70
ENSG00000188613	NANOS1	-2,81	0,65
ENSG00000168813	ZNF507	-2,81	0,74
ENSG00000161791	FMNL3	-2,81	0,56

ensembl gene	gene symbol	Score(d)	Fold Change
ENSG00000186185	KIF18B	-2,81	0,83
ENSG00000163138	PACRGL	-2,81	0,67
ENSG00000133812	SBF2	-2,81	0,56
ENSG00000165282	PIGO	-2,81	0,75
ENSG00000112175	BMP5	-2,81	0,82
ENSG00000155629	PIK3AP1	-2,81	0,63
ENSG00000124802	EEF1E1	-2,81	0,51
ENSG00000086696	HSD17B2	-2,81	0,76
ENSG00000145604	SKP2	-2,81	0,60
ENSG00000166965	RCCD1	-2,81	0,79
ENSG00000183337	BCOR	-2,81	0,68
ENSG00000198301	SDAD1	-2,81	0,81
ENSG00000058668	ATP2B4	-2,80	0,49
ENSG00000198513	ATL1	-2,80	0,67
ENSG00000167196	FBXO22	-2,80	0,74
ENSG00000112624	GLTSCR1L	-2,80	0,79
ENSG00000087085	ACHE	-2,80	0,76
ENSG00000102595	UGGT2	-2,80	0,43
ENSG00000228486	LINC01125	-2,80	0,77
ENSG00000144115	THNSL2	-2,80	0,61
ENSG00000091136	LAMB1	-2,80	0,74
ENSG00000170142	UBE2E1	-2,80	0,72
ENSG00000141425	RPRD1A	-2,80	0,76
ENSG00000171443	ZNF524	-2,79	0,84
ENSG00000131495	NDUFA2	-2,79	0,73
ENSG00000037042	TUBG2	-2,79	0,69
ENSG00000109079	TNFAIP1	-2,79	0,78
ENSG00000185591	SP1	-2,79	0,77
ENSG00000237649	KIFC1	-2,79	0,67
ENSG00000050748	MAPK9	-2,79	0,72
ENSG00000183454	GRIN2A	-2,79	0,75
ENSG00000183722	LHFP	-2,78	0,83
ENSG00000099330	OCEL1	-2,78	0,86
ENSG00000143851	PTPN7	-2,78	0,64
ENSG00000241119	UGT1A9	-2,78	0,79
ENSG00000179172	HNRNPCL1	-2,78	0,82
ENSG00000135083	CCNJL	-2,78	0,78
ENSG00000136828	RALGPS1	-2,78	0,71
ENSG00000267508	ZNF285	-2,78	0,80
ENSG00000144668	ITGA9	-2,77	0,71
ENSG00000179104	TMTC2	-2,77	0,68
ENSG00000130726	TRIM28	-2,77	0,75
ENSG00000071564	TCF3	-2,77	0,80
ENSG00000243244	STON1	-2,77	0,76
ENSG00000196411	EPHB4	-2,77	0,80
ENSG00000185344	ATP6V0A2	-2,77	0,78
ENSG00000119866	BCL11A	-2,77	0,52
ENSG00000136104	RNASEH2B	-2,77	0,70
ENSG00000153982	GDPD1	-2,77	0,44

ensembl gene	gene symbol	Score(d)	Fold Change
ENSG00000118600	TMEM5	-2,77	0,75
ENSG00000105649	RAB3A	-2,77	0,71
ENSG00000148655	C10orf11	-2,77	0,47
ENSG00000125648	SLC25A23	-2,76	0,62
ENSG00000148219	ASTN2	-2,76	0,87
ENSG00000168056	LTBP3	-2,76	0,64
ENSG00000187026	KRTAP21-2	-2,76	0,85
ENSG00000166848	TERF2IP	-2,76	0,69
ENSG00000091428	RAPGEF4	-2,76	0,47
ENSG00000120333	MRPS14	-2,76	0,63
ENSG00000101138	CSTF1	-2,76	0,78
ENSG00000120279	MYCT1	-2,76	0,77
ENSG00000254221	PCDHGB1	-2,76	0,55
ENSG00000166801	FAM111A	-2,76	0,67
ENSG00000013503	POLR3B	-2,76	0,69
ENSG00000059145	UNKL	-2,75	0,83
ENSG00000138606	SHF	-2,75	0,76
ENSG00000221995	TIAF1	-2,75	0,72
ENSG00000177311	ZBTB38	-2,75	0,63
ENSG00000120675	DNAJC15	-2,75	0,71
ENSG00000143546	S100A8	-2,75	0,59
ENSG00000061337	LZTS1	-2,75	0,81
ENSG00000170348	TMED10	-2,75	0,78
ENSG00000157077	ZFYVE9	-2,75	0,66
ENSG00000111199	TRPV4	-2,75	0,87
ENSG00000158805	ZNF276	-2,74	0,77
ENSG00000135824	RGS8	-2,74	0,79
ENSG00000214357	NEURL1B	-2,74	0,86
ENSG00000120899	PTK2B	-2,74	0,71
ENSG00000125378	BMP4	-2,74	0,39
ENSG00000115760	BIRC6	-2,74	0,77
ENSG00000111859	NEDD9	-2,74	0,48
ENSG00000007541	PIGQ	-2,74	0,73
ENSG00000124574	ABCC10	-2,74	0,75
ENSG00000173875	ZNF791	-2,74	0,75
ENSG00000164576	SAP30L	-2,74	0,75
ENSG00000170382	LRRN2	-2,74	0,54
ENSG00000063180	CA11	-2,74	0,78
ENSG00000173391	OLR1	-2,74	0,65
ENSG00000109929	SC5D	-2,73	0,57
ENSG00000114446	IFT57	-2,73	0,75
ENSG00000107099	DOCK8	-2,73	0,68
ENSG00000144711	IQSEC1	-2,73	0,70
ENSG00000103227	LMF1	-2,73	0,61
ENSG00000140876	NUDT7	-2,73	0,71
ENSG00000166797	FAM96A	-2,73	0,70
ENSG00000169607	CKAP2L	-2,73	0,63
ENSG00000196715	VKORC1L1	-2,73	0,72
ENSG00000127022	CANX	-2,73	0,87

ensembl gene	gene symbol	Score(d)	Fold Change
ENSG00000131732	ZCCHC9	-2,73	0,70
ENSG00000135248	FAM71F1	-2,73	0,82
ENSG00000131196	NFATC1	-2,73	0,67
ENSG00000112936	C7	-2,73	0,63
ENSG00000146083	RNF44	-2,73	0,74
ENSG00000072858	SIDT1	-2,73	0,54
ENSG00000230510	PPP5D1	-2,72	0,81
ENSG00000196911	KPNA5	-2,72	0,72
ENSG00000144231	POLR2D	-2,72	0,72
ENSG00000152464	RPP38	-2,72	0,79
ENSG00000122729	ACO1	-2,72	0,73
ENSG00000158161	EYA3	-2,72	0,72
ENSG00000171303	KCNK3	-2,72	0,89
ENSG00000166313	APBB1	-2,72	0,76
ENSG00000169495	HTRA4	-2,72	0,86
ENSG00000167635	ZNF146	-2,72	0,71
ENSG00000186529	CYP4F3	-2,71	0,60
ENSG00000107021	TBC1D13	-2,71	0,74
ENSG00000069020	MAST4	-2,71	0,61
ENSG00000138443	ABI2	-2,71	0,67
ENSG00000171735	CAMTA1	-2,71	0,81
ENSG00000113716	HMGXB3	-2,71	0,83
ENSG00000182504	CEP97	-2,71	0,64
ENSG00000125166	GOT2	-2,71	0,73
ENSG00000140853	NLRC5	-2,70	0,65
ENSG00000186638	KIF24	-2,70	0,72
ENSG00000155530	LRGUK	-2,70	0,75
ENSG00000136240	KDELR2	-2,70	0,79
ENSG00000158985	CDC42SE2	-2,70	0,66
ENSG00000168288	MMADHC	-2,70	0,66
ENSG00000081692	JMJD4	-2,70	0,73
ENSG00000119912	IDE	-2,70	0,79
ENSG00000186399	GOLGA8R	-2,70	0,70
ENSG00000204936	CD177	-2,70	0,63
ENSG00000141627	DYM	-2,69	0,80
ENSG00000101040	ZMYND8	-2,69	0,69
ENSG00000160716	CHRNB2	-2,69	0,85
ENSG00000178896	EXOSC4	-2,69	0,68
ENSG00000132694	ARHGEF11	-2,69	0,74
ENSG00000253797	UTP14C	-2,69	0,76
ENSG00000135070	ISCA1	-2,69	0,84
ENSG00000064687	ABCA7	-2,69	0,75
ENSG00000242419	PCDHGC4	-2,69	0,70
ENSG00000100034	PPM1F	-2,69	0,81
ENSG00000121858	TNFSF10	-2,69	0,53
ENSG00000175857	GAPT	-2,69	0,68
ENSG00000139192	TAPBPL	-2,69	0,73
ENSG00000180386	KRTAP9-7	-2,69	0,81
ENSG00000164405	UQCRQ	-2,69	0,78

ensembl gene	gene symbol	Score(d)	Fold Change
ENSG00000000419	DPM1	-2,68	0,69
ENSG00000185920	PTCH1	-2,68	0,50
ENSG00000163513	TGFBR2	-2,68	0,52
ENSG00000129993	CBFA2T3	-2,68	0,72
ENSG00000137478	FCHSD2	-2,68	0,61
ENSG00000168286	THAP11	-2,68	0,82
ENSG00000204956	PCDHGA1	-2,68	0,59
ENSG00000136709	WDR33	-2,68	0,79
ENSG00000117691	NENF	-2,68	0,69
ENSG00000069943	PIGB	-2,68	0,67
ENSG00000215474	SKOR2	-2,68	0,86
ENSG00000137414	FAM8A1	-2,68	0,76
ENSG00000154429	CCSAP	-2,68	0,63
ENSG00000151632	AKR1C2	-2,67	0,76
ENSG00000126461	SCAF1	-2,67	0,82
ENSG00000186395	KRT10	-2,67	0,63
ENSG00000169962	TAS1R3	-2,67	0,70
ENSG00000125864	BFSP1	-2,67	0,75
ENSG00000018280	SLC11A1	-2,67	0,66
ENSG00000198198	SZT2	-2,67	0,81
ENSG00000137074	APTX	-2,67	0,80
ENSG00000165338	HECTD2	-2,67	0,62
ENSG00000137288	UQCC2	-2,67	0,62
ENSG00000151692	RNF144A	-2,67	0,71
ENSG00000178409	BEND3	-2,67	0,81
ENSG00000171714	ANO5	-2,67	0,52
ENSG00000119715	ESRRB	-2,66	0,76
ENSG00000187736	NHEJ1	-2,66	0,71
ENSG00000254245	PCDHGA3	-2,66	0,56
ENSG00000160917	CPSF4	-2,66	0,74
ENSG00000179776	CDH5	-2,66	0,85
ENSG00000148840	PPRC1	-2,66	0,72
ENSG00000175581	MRPL48	-2,66	0,66
ENSG00000113811	SELK	-2,66	0,77
ENSG00000154930	ACSS1	-2,66	0,67
ENSG00000185532	PRKG1	-2,66	0,34
ENSG00000113328	CCNG1	-2,66	0,75
ENSG00000176953	NFATC2IP	-2,66	0,66
ENSG00000065526	SPEN	-2,66	0,79
ENSG00000178188	SH2B1	-2,66	0,84
ENSG00000198723	C19orf45	-2,65	0,71
ENSG00000173198	CYSLTR1	-2,65	0,53
ENSG00000189050	RNFT1	-2,65	0,75
ENSG00000142149	HUNK	-2,65	0,78
ENSG00000130368	MAS1	-2,65	0,85
ENSG00000189171	S100A13	-2,65	0,78
ENSG00000138670	RASGEF1B	-2,65	0,84
ENSG00000183709	IFNL2	-2,65	0,84
ENSG00000087460	GNAS	-2,65	0,84

ensembl gene	gene symbol	Score(d)	Fold Change
ENSG00000160226	C21orf2	-2,65	0,81
ENSG00000188153	COL4A5	-2,65	0,29
ENSG00000155957	TMBIM4	-2,65	0,79
ENSG00000139173	TMEM117	-2,64	0,75
ENSG00000146674	IGFBP3	-2,64	0,62
ENSG00000155749	ALS2CR12	-2,64	0,69
ENSG00000168530	MYL1	-2,64	0,75
ENSG00000181754	AMIGO1	-2,64	0,79
ENSG00000150054	MPP7	-2,64	0,73
ENSG00000197415	VEPH1	-2,64	0,49
ENSG00000204304	PBX2	-2,64	0,82
ENSG00000137501	SYTL2	-2,64	0,58
ENSG00000114859	CLCN2	-2,64	0,83
ENSG00000075151	EIF4G3	-2,64	0,72
ENSG00000168135	KCNJ4	-2,64	0,86
ENSG00000105516	DBP	-2,63	0,73
ENSG00000164291	ARSK	-2,63	0,69
ENSG00000130513	GDF15	-2,63	0,80
ENSG00000112242	E2F3	-2,63	0,82
ENSG00000129071	MBD4	-2,63	0,78
ENSG00000170615	SLC26A5	-2,63	0,46
ENSG00000135828	RNASEL	-2,63	0,67
ENSG00000106609	TMEM248	-2,63	0,84
ENSG00000239590	OR1J4	-2,63	0,60
ENSG00000135473	PAN2	-2,62	0,72
ENSG00000146094	DOK3	-2,62	0,78
ENSG00000188554	NBR1	-2,62	0,76
ENSG00000164830	OXR1	-2,62	0,77
ENSG00000105793	GTPBP10	-2,62	0,72
ENSG00000174579	MSL2	-2,62	0,78
ENSG00000185404	SP140L	-2,62	0,76
ENSG00000159899	NPR2	-2,62	0,80
ENSG00000103485	QPRT	-2,62	0,58
ENSG00000129636	ITFG1	-2,61	0,73
ENSG00000129472	RAB2B	-2,61	0,75
ENSG00000011454	RABGAP1	-2,61	0,75
ENSG00000112343	TRIM38	-2,61	0,76
ENSG00000119328	FAM206A	-2,61	0,67
ENSG00000239887	C1orf226	-2,61	0,72
ENSG00000176177	ENTHD1	-2,61	0,80
ENSG00000148737	TCF7L2	-2,61	0,64
ENSG00000188107	EYS	-2,61	0,80
ENSG00000144455	SUMF1	-2,61	0,68
ENSG00000141699	FAM134C	-2,61	0,79
ENSG00000188086	PRSS45	-2,61	0,78
ENSG00000136854	STXBP1	-2,61	0,63
ENSG00000176533	GNG7	-2,61	0,70
ENSG00000011485	PPP5C	-2,60	0,65
ENSG00000163463	KRTCAP2	-2,60	0,70

ensembl gene	gene symbol	Score(d)	Fold Change
ENSG00000134323	MYCN	-2,60	0,85
ENSG00000104154	SLC30A4	-2,60	0,70
ENSG00000130227	XPO7	-2,60	0,79
ENSG00000250719	RP11-322N21.2	-2,60	0,81
ENSG00000204965	PCDHA5	-2,60	0,86
ENSG00000113742	CPEB4	-2,60	0,70
ENSG00000110042	DTX4	-2,60	0,82
ENSG00000100427	MLC1	-2,60	0,85
ENSG00000251369	ZNF550	-2,59	0,75
ENSG00000140416	TPM1	-2,59	0,65
ENSG00000140474	ULK3	-2,59	0,67
ENSG00000148218	ALAD	-2,59	0,85
ENSG00000196358	NTNG2	-2,59	0,81
ENSG00000183977	PP2D1	-2,59	0,81
ENSG00000103089	FA2H	-2,59	0,56
ENSG00000132953	XPO4	-2,58	0,74
ENSG00000136731	UGGT1	-2,58	0,81
ENSG00000182489	XKRX	-2,58	0,46
ENSG00000130234	ACE2	-2,58	0,79
ENSG00000204267	TAP2	-2,58	0,71
ENSG00000168229	PTGDR	-2,58	0,52
ENSG00000187486	KCNJ11	-2,58	0,80
ENSG00000172232	AZU1	-2,58	0,78
ENSG00000278845	MRPL45	-2,58	0,70
ENSG00000196228	SULT1C3	-2,58	0,89
ENSG00000078687	TNRC6C	-2,58	0,69
ENSG00000132170	PPARG	-2,58	0,70
ENSG00000111834	RSPH4A	-2,58	0,69
ENSG00000107104	KANK1	-2,58	0,56
ENSG00000184305	CCSER1	-2,58	0,48
ENSG00000185608	MRPL40	-2,58	0,74
ENSG00000125337	KIF25	-2,58	0,84
ENSG00000135709	KIAA0513	-2,57	0,64
ENSG00000159915	ZNF233	-2,57	0,62
ENSG00000005801	ZNF195	-2,57	0,78
ENSG00000166930	MS4A5	-2,57	0,84
ENSG00000169047	IRS1	-2,57	0,62
ENSG00000145916	RMND5B	-2,57	0,79
ENSG00000093009	CDC45	-2,57	0,79
ENSG00000156162	DPY19L4	-2,57	0,70
ENSG00000133983	COX16	-2,57	0,78
ENSG00000148248	SURF4	-2,56	0,84
ENSG00000101363	MANBAL	-2,56	0,78
ENSG00000168936	TMEM129	-2,56	0,84
ENSG00000188396	TCTEX1D4	-2,56	0,88
ENSG00000171643	S100Z	-2,56	0,55
ENSG00000169519	METTL15	-2,56	0,65
ENSG00000076928	ARHGEF1	-2,56	0,74
ENSG00000214686	IQCF6	-2,56	0,90

ensembl gene	gene symbol	Score(d)	Fold Change
ENSG00000110448	CD5	-2,56	0,83
ENSG00000164035	EMCN	-2,56	0,74
ENSG00000104324	CPQ	-2,56	0,62
ENSG00000175344	CHRNA7	-2,56	0,74
ENSG00000151208	DLG5	-2,56	0,72
ENSG00000155085	AK9	-2,56	0,69
ENSG00000175826	CTDNEP1	-2,56	0,82
ENSG00000068137	PLEKHH3	-2,55	0,72
ENSG00000164258	NDUFS4	-2,55	0,67
ENSG00000148798	INA	-2,55	0,70
ENSG00000080200	CRYBG3	-2,55	0,61
ENSG00000139546	TARBP2	-2,55	0,80
ENSG00000138639	ARHGAP24	-2,55	0,67
ENSG00000176720	BOK	-2,55	0,87
ENSG00000211898	IGHD	-2,55	0,50
ENSG00000113593	PPWD1	-2,55	0,82
ENSG00000163220	S100A9	-2,55	0,54
ENSG00000169902	TPST1	-2,55	0,63
ENSG00000030419	IKZF2	-2,54	0,73
ENSG00000167656	LY6D	-2,54	0,81
ENSG00000124177	CHD6	-2,54	0,71
ENSG00000106571	GLI3	-2,54	0,57
ENSG00000188211	NCR3LG1	-2,54	0,60
ENSG00000113300	CNOT6	-2,54	0,81
ENSG00000150977	RILPL2	-2,54	0,69
ENSG00000172086	KRCC1	-2,54	0,69
ENSG00000118520	ARG1	-2,54	0,71
ENSG00000137944	KYAT3	-2,54	0,74
ENSG00000128567	PODXL	-2,54	0,79
ENSG00000082213	C5orf22	-2,54	0,82
ENSG00000136114	THSD1	-2,53	0,73
ENSG00000123268	ATF1	-2,53	0,81
ENSG00000157150	TIMP4	-2,53	0,87
ENSG00000136895	GARNL3	-2,53	0,66
ENSG00000113048	MRPS27	-2,53	0,71
ENSG00000270024	C8orf44-SGK3	-2,53	0,57
ENSG00000177042	TMEM80	-2,53	0,78
ENSG00000126091	ST3GAL3	-2,52	0,77
ENSG00000103091	WDR59	-2,52	0,80
ENSG00000105677	TMEM147	-2,52	0,71
ENSG00000164244	PRRC1	-2,52	0,83
ENSG00000214128	TMEM213	-2,52	0,78
ENSG00000137770	CTDSPL2	-2,52	0,77
ENSG00000196781	TLE1	-2,52	0,49
ENSG00000173715	C11orf80	-2,52	0,70
ENSG00000112659	CUL9	-2,52	0,84
ENSG00000154743	TSEN2	-2,52	0,71
ENSG00000109861	CTSC	-2,52	0,59
ENSG00000163430	FSTL1	-2,52	0,75

ensembl gene	gene symbol	Score(d)	Fold Change
ENSG00000149091	DGKZ	-2,52	0,79
ENSG00000115756	HPCAL1	-2,52	0,70
ENSG00000134824	FADS2	-2,52	0,68
ENSG00000182670	TTC3	-2,52	0,78
ENSG00000271303	SRXN1	-2,52	0,82
ENSG00000184347	SLIT3	-2,52	0,82
ENSG00000146802	TMEM168	-2,52	0,66
ENSG00000124102	PI3	-2,52	0,84
ENSG00000171121	KCNMB3	-2,52	0,71
ENSG00000105011	ASF1B	-2,52	0,66
ENSG00000108244	KRT23	-2,52	0,79
ENSG00000128052	KDR	-2,51	0,80
ENSG00000254656	RTL1	-2,51	0,86
ENSG00000171067	C11orf24	-2,51	0,78
ENSG00000105438	KDELR1	-2,51	0,81
ENSG00000221994	ZNF630	-2,51	0,70
ENSG00000006831	ADIPOR2	-2,51	0,60
ENSG00000130299	GTPBP3	-2,51	0,85
ENSG00000139163	ETNK1	-2,51	0,75
ENSG00000239839	DEFA3	-2,51	0,43
ENSG00000223573	TINCR	-2,51	0,76
ENSG00000110975	SYT10	-2,51	0,74
ENSG00000187626	ZKSCAN4	-2,51	0,83
ENSG00000158717	RNF166	-2,51	0,74
ENSG00000164073	MFSD8	-2,50	0,77
ENSG00000076351	SLC46A1	-2,50	0,76
ENSG00000168101	NUDT16L1	-2,50	0,88
ENSG00000136770	DNAJC1	-2,50	0,75
ENSG00000165997	ARL5B	-2,50	0,77
ENSG00000114923	SLC4A3	-2,50	0,80
ENSG00000185518	SV2B	-2,50	0,87
ENSG00000130032	PRRG3	-2,49	0,77
ENSG00000163993	S100P	-2,49	0,70
ENSG00000163687	DNASE1L3	-2,49	0,76
ENSG00000076770	MBNL3	-2,49	0,61
ENSG00000130734	ATG4D	-2,49	0,84
ENSG00000028310	BRD9	-2,49	0,73
ENSG00000169740	ZNF32	-2,49	0,74
ENSG00000168916	ZNF608	-2,49	0,54
ENSG00000101493	ZNF516	-2,49	0,76
ENSG00000088970	KIZ	-2,49	0,78
ENSG00000148346	LCN2	-2,49	0,69
ENSG00000109927	TECTA	-2,49	0,64
ENSG00000136874	STX17	-2,49	0,79
ENSG00000084764	MAPRE3	-2,49	0,80
ENSG00000169684	CHRNA5	-2,49	0,54
ENSG00000142694	EVA1B	-2,49	0,77
ENSG00000167700	MFSD3	-2,49	0,73
ENSG00000130649	CYP2E1	-2,49	0,78

ensembl gene	gene symbol	Score(d)	Fold Change
ENSG00000169282	KCNAB1	-2,49	0,82
ENSG00000144642	RBMS3	-2,49	0,55
ENSG00000164465	DCBLD1	-2,48	0,68
ENSG00000130812	ANGPTL6	-2,48	0,87
ENSG00000168298	HIST1H1E	-2,48	0,74
ENSG00000197044	ZNF441	-2,48	0,83
ENSG00000159239	C2orf81	-2,48	0,85
ENSG00000090581	GNPTG	-2,48	0,69
ENSG00000268182	SMIM17	-2,48	0,87
ENSG00000119699	TGFB3	-2,47	0,81
ENSG00000166716	ZNF592	-2,47	0,78
ENSG00000186510	CLCNKA	-2,47	0,69
ENSG00000137103	TMEM8B	-2,47	0,81
ENSG00000157741	UBN2	-2,47	0,74
ENSG00000237489	LINC00959	-2,47	0,81
ENSG00000196557	CACNA1H	-2,47	0,79
ENSG00000204628	RACK1	-2,47	0,87
ENSG00000171634	BPTF	-2,47	0,79
ENSG00000013561	RNF14	-2,47	0,72
ENSG00000155100	OTUD6B	-2,47	0,75
ENSG00000162736	NCSTN	-2,47	0,79
ENSG00000136383	ALPK3	-2,46	0,62
ENSG00000059588	TARBP1	-2,46	0,65
ENSG00000172497	ACOT12	-2,46	0,73
ENSG00000064012	CASP8	-2,46	0,68
ENSG00000198690	FAN1	-2,46	0,79
ENSG00000151893	CACUL1	-2,46	0,77
ENSG00000161955	TNFSF13	-2,46	0,47
ENSG00000183474	GTF2H2C	-2,46	0,70
ENSG00000127720	METTL25	-2,46	0,72
ENSG00000165138	ANKS6	-2,46	0,75
ENSG00000232070	TMEM253	-2,46	0,89
ENSG00000146276	GABRR1	-2,46	0,79
ENSG00000203780	FANK1	-2,46	0,85
ENSG00000105369	CD79A	-2,46	0,39
ENSG00000174500	GCSAM	-2,46	0,42
ENSG00000104140	RHOV	-2,46	0,88
ENSG00000198336	MYL4	-2,46	0,83
ENSG00000155893	PXYLP1	-2,45	0,73
ENSG00000135999	EPC2	-2,45	0,76
ENSG00000187961	KLHL17	-2,45	0,87
ENSG00000169031	COL4A3	-2,45	0,67
ENSG00000111816	FRK	-2,45	0,56
ENSG00000087095	NLK	-2,45	0,70
ENSG00000165325	CCDC67	-2,45	0,86
ENSG00000135097	MSI1	-2,45	0,86
ENSG00000165632	TAF3	-2,45	0,84
ENSG00000144857	BOC	-2,45	0,53
ENSG00000189058	APOD	-2,45	0,61

ensembl gene	gene symbol	Score(d)	Fold Change
ENSG00000183662	FAM19A1	-2,45	0,82
ENSG00000127588	GNG13	-2,45	0,89
ENSG00000115825	PRKD3	-2,45	0,46
ENSG00000135413	LACRT	-2,45	0,89
ENSG00000151650	VENTX	-2,45	0,86
ENSG00000063176	SPHK2	-2,45	0,85
ENSG00000253873	PCDHGA11	-2,45	0,66
ENSG00000176472	ZNF575	-2,44	0,83
ENSG00000213694	S1PR3	-2,44	0,78
ENSG00000177200	CHD9	-2,44	0,74
ENSG00000121314	TAS2R8	-2,44	0,80
ENSG00000146576	C7orf26	-2,44	0,79
ENSG00000177302	TOP3A	-2,44	0,80
ENSG00000152291	TGOLN2	-2,44	0,84
ENSG00000149929	HIRIP3	-2,44	0,82
ENSG00000182985	CADM1	-2,44	0,30
ENSG00000146966	DENND2A	-2,44	0,85
ENSG00000173376	NDNF	-2,43	0,49
ENSG00000173757	STAT5B	-2,43	0,63
ENSG00000105443	CYTH2	-2,43	0,79
ENSG00000226174	TEX22	-2,43	0,85
ENSG00000102837	OLFM4	-2,43	0,48
ENSG00000138073	PREB	-2,43	0,68
ENSG00000178761	FAM219B	-2,43	0,78
ENSG00000136247	ZDHHC4	-2,43	0,85
ENSG00000166839	ANKDD1A	-2,43	0,82
ENSG00000159753	CARMIL2	-2,43	0,75
ENSG00000040341	STAU2	-2,43	0,79
ENSG00000119977	TCTN3	-2,42	0,74
ENSG00000175063	UBE2C	-2,42	0,79
ENSG00000241360	PDXP	-2,42	0,85
ENSG00000144868	TMEM108	-2,42	0,52
ENSG00000166352	C11orf74	-2,42	0,63
ENSG00000158077	NLRP14	-2,42	0,61
ENSG00000272617	COG8	-2,42	0,69
ENSG00000106333	PCOLCE	-2,42	0,71
ENSG00000128654	MTX2	-2,42	0,78
ENSG00000107562	CXCL12	-2,42	0,43
ENSG00000103994	ZNF106	-2,42	0,68
ENSG00000110492	MDK	-2,42	0,53
ENSG00000164086	DUSP7	-2,42	0,71
ENSG00000103313	MEFV	-2,42	0,78
ENSG00000140943	MBTPS1	-2,41	0,78
ENSG00000074181	NOTCH3	-2,41	0,89
ENSG00000127311	HELB	-2,41	0,68
ENSG00000184992	BRI3BP	-2,41	0,79
ENSG00000256771	ZNF253	-2,41	0,71
ENSG00000047346	FAM214A	-2,41	0,73
ENSG00000198121	LPAR1	-2,41	0,62

ensembl gene	gene symbol	Score(d)	Fold Change
ENSG00000153291	SLC25A27	-2,41	0,48
ENSG00000108231	LGI1	-2,41	0,88
ENSG00000162373	BEND5	-2,41	0,75
ENSG00000182551	ADI1	-2,41	0,78
ENSG00000166263	STXBP4	-2,41	0,68
ENSG00000149403	GRIK4	-2,40	0,55
ENSG00000173409	ARV1	-2,40	0,74
ENSG00000136197	C7orf25	-2,40	0,66
ENSG00000176732	PFN4	-2,40	0,83
ENSG00000167968	DNASE1L2	-2,40	0,88
ENSG00000072818	ACAP1	-2,40	0,65
ENSG00000129194	SOX15	-2,40	0,86
ENSG00000181817	LSM10	-2,40	0,79
ENSG00000126804	ZBTB1	-2,40	0,85
ENSG00000130479	MAP1S	-2,40	0,81
ENSG00000143458	GABPB2	-2,40	0,69
ENSG00000197165	SULT1A2	-2,40	0,82
ENSG00000141556	TBCD	-2,39	0,77
ENSG00000125844	RRBP1	-2,39	0,68
ENSG00000136237	RAPGEF5	-2,39	0,47
ENSG00000113194	FAF2	-2,39	0,82
ENSG00000188735	TMEM120B	-2,39	0,80
ENSG00000110719	TCIRG1	-2,39	0,76
ENSG00000171703	TCEA2	-2,39	0,77
ENSG00000177380	PPFIA3	-2,39	0,75
ENSG00000164880	INTS1	-2,39	0,79
ENSG00000149177	PTPRJ	-2,39	0,58
ENSG00000172116	CD8B	-2,39	0,74
ENSG00000089692	LAG3	-2,39	0,81
ENSG00000197706	OR6C74	-2,39	0,86
ENSG00000144028	SNRNP200	-2,39	0,85
ENSG00000164754	RAD21	-2,39	0,84
ENSG00000080166	DCT	-2,39	0,72
ENSG00000223609	HBD	-2,38	0,69
ENSG00000181991	MRPS11	-2,38	0,73
ENSG00000160963	COL26A1	-2,38	0,90
ENSG00000100711	ZFYVE21	-2,38	0,78
ENSG00000065427	KARS	-2,38	0,79
ENSG00000007129	CEACAM21	-2,38	0,76
ENSG00000138079	SLC3A1	-2,38	0,79
ENSG00000063046	EIF4B	-2,38	0,82
ENSG00000184012	TMPRSS2	-2,38	0,75
ENSG00000197062	ZSCAN26	-2,38	0,78
ENSG00000106638	TBL2	-2,38	0,81
ENSG00000186591	UBE2H	-2,38	0,80
ENSG00000170745	KCNS3	-2,38	0,51
ENSG00000176641	RNF152	-2,38	0,85
ENSG00000058866	DGKG	-2,38	0,66
ENSG00000150681	RGS18	-2,38	0,64

ensembl gene	gene symbol	Score(d)	Fold Change
ENSG00000105287	PRKD2	-2,38	0,68
ENSG00000144468	RHBDD1	-2,38	0,75
ENSG00000089199	CHGB	-2,38	0,81
ENSG00000138768	USO1	-2,37	0,78
ENSG00000145723	GIN1	-2,37	0,62
ENSG00000164694	FNDC1	-2,37	0,76
ENSG00000134152	KATNBL1	-2,37	0,57
ENSG00000118418	HMGN3	-2,37	0,80
ENSG00000230778	ANKRD63	-2,37	0,88
ENSG00000135299	ANKRD6	-2,37	0,69
ENSG00000070269	TMEM260	-2,37	0,76
ENSG00000124602	UNC5CL	-2,37	0,87
ENSG00000152443	ZNF776	-2,37	0,76
ENSG00000184786	TCTE3	-2,37	0,74
ENSG00000135046	ANXA1	-2,37	0,39
ENSG00000166987	MBD6	-2,36	0,77
ENSG00000138613	APH1B	-2,36	0,66
ENSG00000124772	CPNE5	-2,36	0,64
ENSG00000175283	DOLK	-2,36	0,80
ENSG00000215545	DEFB116	-2,36	0,87
ENSG00000099377	HSD3B7	-2,36	0,86
ENSG00000134061	CD180	-2,36	0,48
ENSG00000161921	CXCL16	-2,36	0,61
ENSG00000082074	FYB	-2,36	0,61
ENSG00000107554	DNMBP	-2,36	0,65
ENSG00000253305	PCDHGB6	-2,36	0,61
ENSG00000143512	HHIPL2	-2,36	0,83
ENSG00000174547	MRPL11	-2,36	0,76
ENSG00000111536	IL26	-2,36	0,78
ENSG00000104093	DMXL2	-2,36	0,55
ENSG00000182700	IGIP	-2,36	0,79
ENSG00000187118	CMC1	-2,35	0,76
ENSG00000163536	SERPINI1	-2,35	0,48
ENSG00000101624	CEP76	-2,35	0,76
ENSG00000172987	HPSE2	-2,35	0,87
ENSG00000170291	ELP5	-2,35	0,79
ENSG00000044090	CUL7	-2,35	0,82
ENSG00000110074	FOXRED1	-2,35	0,80
ENSG00000187123	LYPD6	-2,35	0,87
ENSG00000135362	PRR5L	-2,35	0,80
ENSG00000135637	CCDC142	-2,35	0,79
ENSG00000198128	OR2L3	-2,35	0,85
ENSG00000170545	SMAGP	-2,35	0,60
ENSG00000126561	STAT5A	-2,35	0,60
ENSG00000128272	ATF4	-2,35	0,87
ENSG00000076067	RBMS2	-2,35	0,73
ENSG00000169435	RASSF6	-2,35	0,54
ENSG00000139133	ALG10	-2,35	0,71
ENSG00000262576	PCDHGA4	-2,34	0,71

ensembl gene	gene symbol	Score(d)	Fold Change
ENSG00000169641	LUZP1	-2,34	0,72
ENSG00000178690	DYNAP	-2,34	0,85
ENSG00000134917	ADAMTS8	-2,34	0,82
ENSG00000154589	LY96	-2,34	0,61
ENSG00000154642	C21orf91	-2,34	0,72
ENSG00000145284	SCD5	-2,34	0,66
ENSG00000152683	SLC30A6	-2,34	0,77
ENSG00000178878	APOLD1	-2,34	0,82
ENSG00000117461	PIK3R3	-2,34	0,62
ENSG00000103569	AQP9	-2,34	0,57
ENSG00000112378	PERP	-2,34	0,68
ENSG00000160877	NACC1	-2,34	0,80
ENSG00000158458	NRG2	-2,34	0,71
ENSG00000120328	PCDHB12	-2,34	0,69
ENSG00000145700	ANKRD31	-2,33	0,68
ENSG00000183049	CAMK1D	-2,33	0,62
ENSG00000101126	ADNP	-2,33	0,85
ENSG00000215790	SLC35E2	-2,33	0,73
ENSG00000189362	NEMP2	-2,33	0,74
ENSG00000156009	MAGEA8	-2,33	0,87
ENSG00000249242	TMEM150C	-2,33	0,80
ENSG00000181035	SLC25A42	-2,33	0,74
ENSG00000171853	TRAPPC12	-2,33	0,72
ENSG00000114745	GORASP1	-2,33	0,78
ENSG00000214891	TRIM64C	-2,33	0,83
ENSG00000186409	CCDC30	-2,33	0,79
ENSG00000171236	LRG1	-2,33	0,75
ENSG00000154309	DISP1	-2,33	0,74
ENSG00000167642	SPINT2	-2,33	0,49
ENSG00000138193	PLCE1	-2,33	0,91
ENSG00000112419	PHACTR2	-2,33	0,59
ENSG00000118518	RNF146	-2,33	0,78
ENSG00000026950	BTN3A1	-2,32	0,66
ENSG00000197363	ZNF517	-2,32	0,86
ENSG00000171444	MCC	-2,32	0,63
ENSG00000153162	BMP6	-2,32	0,67
ENSG00000100084	HIRA	-2,32	0,67
ENSG00000213760	ATP6V1G2	-2,32	0,82
ENSG00000127074	RGS13	-2,32	0,31
ENSG00000140961	OSGIN1	-2,32	0,86
ENSG00000125449	ARMC7	-2,32	0,81
ENSG00000267561	RP5-1052I5.2	-2,32	0,71
ENSG00000182481	KPNA2	-2,32	0,75
ENSG00000107829	FBXW4	-2,32	0,76
ENSG00000141965	FEM1A	-2,32	0,80
ENSG00000117472	TSPAN1	-2,32	0,74
ENSG00000041353	RAB27B	-2,32	0,74
ENSG00000088766	CRLS1	-2,32	0,75
ENSG00000163882	POLR2H	-2,32	0,72

ensembl gene	gene symbol	Score(d)	Fold Change
ENSG00000179388	EGR3	-2,32	0,74
ENSG00000050438	SLC4A8	-2,32	0,82
ENSG00000135480	KRT7	-2,31	0,74
ENSG00000167081	PBX3	-2,31	0,71
ENSG00000131089	ARHGEF9	-2,31	0,77
ENSG00000122705	CLTA	-2,31	0,78
ENSG00000162227	TAF6L	-2,31	0,79
ENSG00000169570	DTWD2	-2,31	0,78
ENSG00000264364	DYNLL2	-2,31	0,78
ENSG00000121068	TBX2	-2,31	0,85
ENSG00000132466	ANKRD17	-2,31	0,84
ENSG00000162980	ARL5A	-2,31	0,71
ENSG00000274750	HIST1H3E	-2,31	0,56
ENSG00000139826	ABHD13	-2,31	0,80
ENSG00000164080	RAD54L2	-2,31	0,85
ENSG00000165186	PTCHD1	-2,31	0,81
ENSG00000186431	FCAR	-2,31	0,65
ENSG00000153790	C7orf31	-2,31	0,77
ENSG00000172199	OR8U1	-2,31	0,87
ENSG00000140398	NEIL1	-2,30	0,60
ENSG00000182896	TMEM95	-2,30	0,89
ENSG00000163121	NEURL3	-2,30	0,90
ENSG00000186812	ZNF397	-2,30	0,75
ENSG00000181458	TMEM45A	-2,30	0,70
ENSG00000174928	C3orf33	-2,30	0,82
ENSG00000198088	NUP62CL	-2,30	0,70
ENSG00000172345	STARD5	-2,30	0,66
ENSG00000186496	ZNF396	-2,30	0,84
ENSG00000163539	CLASP2	-2,30	0,79
ENSG00000119408	NEK6	-2,30	0,52
ENSG00000006025	OSBPL7	-2,30	0,85
ENSG00000166452	AKIP1	-2,30	0,75
ENSG00000165699	TSC1	-2,30	0,85
ENSG00000169760	NLGN1	-2,30	0,70
ENSG00000258405	ZNF578	-2,30	0,81
ENSG00000172201	ID4	-2,30	0,85
ENSG00000075420	FNDC3B	-2,29	0,77
ENSG00000106785	TRIM14	-2,29	0,87
ENSG00000155974	GRIP1	-2,29	0,50
ENSG00000121897	LIAS	-2,29	0,74
ENSG00000122778	KIAA1549	-2,29	0,67
ENSG00000108984	MAP2K6	-2,29	0,60
ENSG00000063245	EPN1	-2,29	0,83
ENSG00000179023	KLHDC7A	-2,29	0,88
ENSG00000144840	RABL3	-2,29	0,77
ENSG00000135547	HEY2	-2,29	0,49
ENSG00000033800	PIAS1	-2,29	0,81
ENSG00000170234	PWWP2A	-2,29	0,83
ENSG00000115556	PLCD4	-2,29	0,72

ensembl gene	gene symbol	Score(d)	Fold Change
ENSG00000270011	ZNF559-ZNF177	-2,29	0,61
ENSG00000273749	CYFIP1	-2,29	0,69
ENSG00000114735	HEMK1	-2,29	0,85
ENSG00000122986	HVCN1	-2,29	0,66
ENSG00000169857	AVEN	-2,29	0,75
ENSG00000158773	USF1	-2,28	0,71
ENSG00000043143	JADE2	-2,28	0,73
ENSG00000120913	PDLIM2	-2,28	0,78
ENSG00000187650	VMAC	-2,28	0,81
ENSG00000166794	PPIB	-2,28	0,91
ENSG00000147082	CCNB3	-2,28	0,69
ENSG00000182685	BRICD5	-2,28	0,89
ENSG00000111291	GPRC5D	-2,28	0,56
ENSG00000174059	CD34	-2,28	0,84
ENSG00000129151	BBOX1	-2,27	0,53
ENSG00000144357	UBR3	-2,27	0,82
ENSG00000244005	NFS1	-2,27	0,70
ENSG00000143006	DMRTB1	-2,27	0,82
ENSG00000188573	FBLL1	-2,27	0,89
ENSG00000166181	API5	-2,27	0,84
ENSG00000204257	HLA-DMA	-2,27	0,60
ENSG00000089597	GANAB	-2,27	0,84
ENSG00000204572	KRTAP5-10	-2,27	0,87
ENSG00000136521	NDUFB5	-2,27	0,67
ENSG00000269858	EGLN2	-2,26	0,66
ENSG00000095059	DHPS	-2,26	0,72
ENSG00000112658	SRF	-2,26	0,78
ENSG00000113319	RASGRF2	-2,26	0,89
ENSG00000158079	PTPDC1	-2,26	0,74
ENSG00000185880	TRIM69	-2,26	0,56
ENSG00000167380	ZNF226	-2,26	0,74
ENSG00000197912	SPG7	-2,26	0,81
ENSG00000183631	PRR32	-2,26	0,89
ENSG00000215612	HMX1	-2,26	0,90
ENSG00000083828	ZNF586	-2,26	0,79
ENSG00000064607	SUGP2	-2,26	0,82
ENSG00000125304	TM9SF2	-2,26	0,81
ENSG00000166927	MS4A7	-2,26	0,64
ENSG00000185775	SPATA31A6	-2,26	0,89
ENSG00000152767	FARP1	-2,26	0,60
ENSG00000051620	HEBP2	-2,26	0,77
ENSG00000055118	KCNH2	-2,26	0,67
ENSG00000203710	CR1	-2,26	0,65
ENSG00000167535	CACNB3	-2,26	0,74
ENSG00000113070	HBEGF	-2,25	0,69
ENSG00000168036	CTNNB1	-2,25	0,76
ENSG00000198018	ENTPD7	-2,25	0,77
ENSG00000197798	FAM118B	-2,25	0,69
ENSG00000176444	CLK2	-2,25	0,76

ensembl gene	gene symbol	Score(d)	Fold Change
ENSG00000107566	ERLIN1	-2,25	0,76
ENSG00000161813	LARP4	-2,25	0,82
ENSG00000243364	EFNA4	-2,25	0,72
ENSG00000096070	BRPF3	-2,25	0,82
ENSG00000214562	NUTM2D	-2,25	0,80
ENSG00000078699	CBFA2T2	-2,25	0,72
ENSG00000106348	IMPDH1	-2,25	0,84
ENSG00000179002	TAS1R2	-2,25	0,82
ENSG00000090382	LYZ	-2,25	0,55
ENSG00000177984	LCN15	-2,25	0,88
ENSG00000164068	RNF123	-2,25	0,78
ENSG00000186265	BTLA	-2,25	0,70
ENSG00000189114	BLOC1S3	-2,25	0,86
ENSG00000072952	MRVI1	-2,25	0,88
ENSG00000111275	ALDH2	-2,25	0,74
ENSG00000162004	CCDC78	-2,24	0,80
ENSG00000167220	HDHD2	-2,24	0,71
ENSG00000159079	C21orf59	-2,24	0,81
ENSG00000182798	MAGEB17	-2,24	0,87
ENSG00000172209	GPR22	-2,24	0,86
ENSG00000132704	FCRL2	-2,24	0,51
ENSG00000187954	CYHR1	-2,24	0,89
ENSG00000243264	IGKV2D-29	-2,24	0,58
ENSG00000135469	COQ10A	-2,24	0,70
ENSG00000165730	STOX1	-2,24	0,69
ENSG00000172927	MYEOV	-2,24	0,60
ENSG00000198841	KTI12	-2,24	0,78
ENSG00000196345	ZKSCAN7	-2,24	0,71
ENSG00000006282	SPATA20	-2,23	0,79
ENSG00000060237	WNK1	-2,23	0,73
ENSG00000258227	CLEC5A	-2,23	0,82
ENSG00000089335	ZNF302	-2,23	0,83
ENSG00000137393	RNF144B	-2,23	0,56
ENSG00000140391	TSPAN3	-2,23	0,81
ENSG00000081853	PCDHGA2	-2,23	0,72
ENSG00000139651	ZNF740	-2,23	0,78
ENSG00000119508	NR4A3	-2,23	0,58
ENSG00000197381	ADARB1	-2,23	0,68
ENSG00000189339	SLC35E2B	-2,23	0,73
ENSG00000196427	NBPF4	-2,23	0,73
ENSG00000123836	PFKFB2	-2,23	0,66
ENSG00000007866	TEAD3	-2,23	0,82
ENSG00000204414	CSHL1	-2,23	0,88
ENSG00000175928	LRRN1	-2,23	0,71
ENSG00000151135	TMEM263	-2,23	0,76
ENSG00000039600	SOX30	-2,23	0,82
ENSG00000138336	TET1	-2,22	0,65
ENSG00000086548	CEACAM6	-2,22	0,61
ENSG00000181690	PLAG1	-2,22	0,41

ensembl gene	gene symbol	Score(d)	Fold Change
ENSG00000092009	CMA1	-2,22	0,91
ENSG00000180855	ZNF443	-2,22	0,72
ENSG00000108691	CCL2	-2,22	0,41
ENSG00000137040	RANBP6	-2,22	0,76
ENSG00000129968	ABHD17A	-2,22	0,85
ENSG00000198298	ZNF485	-2,22	0,86
ENSG00000154553	PDLIM3	-2,22	0,53
ENSG00000182871	COL18A1	-2,22	0,76
ENSG00000150556	LYPD6B	-2,21	0,57
ENSG00000196834	POTEI	-2,21	0,78
ENSG00000119638	NEK9	-2,21	0,80
ENSG00000205038	PKHD1L1	-2,21	0,65
ENSG00000239704	CDRT4	-2,21	0,64
ENSG00000134574	DDB2	-2,21	0,75
ENSG00000095066	HOOK2	-2,21	0,72
ENSG00000138430	OLA1	-2,21	0,79
ENSG00000111863	ADTRP	-2,21	0,53
ENSG00000169908	TM4SF1	-2,21	0,73
ENSG00000149428	HYOU1	-2,21	0,84
ENSG00000123901	GPR83	-2,21	0,86
ENSG00000129009	ISLR	-2,21	0,47
ENSG00000134452	FBXO18	-2,21	0,76
ENSG00000125245	GPR18	-2,20	0,84
ENSG00000157423	HYDIN	-2,20	0,70
ENSG00000172663	TMEM134	-2,20	0,85
ENSG00000157343	ARMC12	-2,20	0,82
ENSG00000130300	PLVAP	-2,20	0,85
ENSG00000166262	FAM227B	-2,20	0,73
ENSG00000184761	AC013269.5	-2,20	0,84
ENSG00000151304	SRFBP1	-2,20	0,77
ENSG00000103222	ABCC1	-2,20	0,70
ENSG00000276966	HIST1H4E	-2,20	0,55
ENSG00000169764	UGP2	-2,20	0,79
ENSG00000165682	CLEC1B	-2,20	0,79
ENSG00000157637	SLC38A10	-2,20	0,78
ENSG00000101307	SIRPB1	-2,20	0,61
ENSG00000152455	SUV39H2	-2,20	0,71
ENSG00000158445	KCNB1	-2,20	0,86
ENSG00000100504	PYGL	-2,20	0,64
ENSG00000106304	SPAM1	-2,19	0,90
ENSG00000137502	RAB30	-2,19	0,70
ENSG00000100678	SLC8A3	-2,19	0,66
ENSG00000175048	ZDHHC14	-2,19	0,58
ENSG00000131724	IL13RA1	-2,19	0,58
ENSG00000253953	PCDHGB4	-2,19	0,68
ENSG00000102383	ZDHHC15	-2,19	0,56
ENSG00000121381	TAS2R9	-2,19	0,70
ENSG00000170325	PRDM10	-2,19	0,83
ENSG00000009709	PAX7	-2,19	0,88

ensembl gene	gene symbol	Score(d)	Fold Change
ENSG00000011465	DCN	-2,18	0,83
ENSG00000060642	PIGV	-2,18	0,77
ENSG00000086598	TMED2	-2,18	0,85
ENSG00000168802	CHTF8	-2,18	0,84
ENSG00000205078	SYCE1L	-2,18	0,82
ENSG00000226887	ERVMER34-1	-2,18	0,76
ENSG00000154380	ENAH	-2,18	0,57
ENSG00000166803	KIAA0101	-2,18	0,75
ENSG00000198283	OR5B21	-2,18	0,72
ENSG00000020426	MNAT1	-2,18	0,76
ENSG00000163202	LCE3D	-2,18	0,87
ENSG00000119812	FAM98A	-2,18	0,78
ENSG00000070761	CFAP20	-2,18	0,78
ENSG00000159461	AMFR	-2,18	0,77
ENSG00000198131	ZNF544	-2,18	0,58
ENSG00000183044	ABAT	-2,17	0,82
ENSG00000175040	CHST2	-2,17	0,61
ENSG00000121481	RNF2	-2,17	0,74
ENSG00000130158	DOCK6	-2,17	0,80
ENSG00000162086	ZNF75A	-2,17	0,76
ENSG00000165899	OTOGL	-2,17	0,62
ENSG00000185386	MAPK11	-2,17	0,77
ENSG00000205808	PLPP6	-2,17	0,84
ENSG00000176563	CNTD1	-2,17	0,89
ENSG00000119547	ONECUT2	-2,17	0,88
ENSG00000131779	PEX11B	-2,17	0,73
ENSG00000092607	TBX15	-2,17	0,66
ENSG00000196584	XRCC2	-2,17	0,71
ENSG00000106355	LSM5	-2,17	0,79
ENSG00000186184	POLR1D	-2,17	0,79
ENSG00000113448	PDE4D	-2,17	0,48
ENSG00000272804	KRTAP10-7	-2,17	0,86
ENSG00000173530	TNFRSF10D	-2,17	0,69
ENSG00000140950	TLDC1	-2,16	0,87
ENSG00000070182	SPTB	-2,16	0,83
ENSG00000198369	SPRED2	-2,16	0,76
ENSG00000197162	ZNF785	-2,16	0,73
ENSG00000134480	CCNH	-2,16	0,79
ENSG00000156869	FRRS1	-2,16	0,70
ENSG00000184709	LRRC26	-2,16	0,77
ENSG00000174607	UGT8	-2,16	0,49
ENSG00000112787	FBRSL1	-2,16	0,82
ENSG00000120324	PCDHB10	-2,16	0,71
ENSG00000166595	FAM96B	-2,16	0,81
ENSG00000188089	PLA2G4E	-2,16	0,83
ENSG00000170209	ANKK1	-2,16	0,82
ENSG00000206102	KRTAP19-8	-2,16	0,61
ENSG00000185504	FAAP100	-2,15	0,83
ENSG00000164764	SBSPON	-2,15	0,89

ensembl gene	gene symbol	Score(d)	Fold Change
ENSG00000198863	RUNDC1	-2,15	0,81
ENSG00000142541	RPL13A	-2,15	0,72
ENSG00000114503	NCBP2	-2,15	0,80
ENSG00000160323	ADAMTS13	-2,15	0,91
ENSG00000204348	DXO	-2,15	0,79
ENSG00000166326	TRIM44	-2,15	0,82
ENSG00000133063	CHIT1	-2,15	0,79
ENSG00000108599	AKAP10	-2,15	0,83
ENSG00000186912	P2RY4	-2,15	0,87
ENSG00000152229	PSTPIP2	-2,15	0,62
ENSG00000107338	SHB	-2,15	0,83
ENSG00000152380	FAM151B	-2,15	0,69
ENSG00000214113	LYRM4	-2,15	0,63
ENSG00000175899	A2M	-2,15	0,66
ENSG00000066294	CD84	-2,15	0,65
ENSG00000205758	CRYZL1	-2,15	0,79
ENSG00000136156	ITM2B	-2,15	0,79
ENSG00000090534	THPO	-2,15	0,78
ENSG00000203782	LOR	-2,15	0,91
ENSG00000179564	LSMEM2	-2,15	0,89
ENSG00000157303	SUSD3	-2,14	0,72
ENSG00000116191	RALGPS2	-2,14	0,73
ENSG00000234465	PINLYP	-2,14	0,80
ENSG00000168509	HFE2	-2,14	0,86
ENSG00000214013	GANC	-2,14	0,74
ENSG00000133083	DCLK1	-2,14	0,57
ENSG00000156042	CFAP70	-2,14	0,82
ENSG00000188389	PDCD1	-2,14	0,88
ENSG00000166377	ATP9B	-2,14	0,79
ENSG00000160551	TAOK1	-2,14	0,82
ENSG00000111537	IFNG	-2,14	0,72
ENSG00000198326	TMEM239	-2,14	0,87
ENSG00000159314	ARHGAP27	-2,14	0,83
ENSG00000137077	CCL21	-2,14	0,91
ENSG00000129128	SPCS3	-2,14	0,84
ENSG00000104884	ERCC2	-2,14	0,80
ENSG00000186790	FOXE3	-2,14	0,91
ENSG00000162994	CLHC1	-2,14	0,75
ENSG00000184988	TMEM106A	-2,14	0,78
ENSG00000158966	CACHD1	-2,14	0,69
ENSG00000130508	PXDN	-2,13	0,42
ENSG00000169085	C8orf46	-2,13	0,77
ENSG00000185340	GAS2L1	-2,13	0,86
ENSG00000213339	QTRT1	-2,13	0,79
ENSG00000124469	CEACAM8	-2,13	0,53
ENSG00000117013	KCNQ4	-2,13	0,88
ENSG00000145087	STXBP5L	-2,13	0,61
ENSG00000101825	MXRA5	-2,13	0,88
ENSG00000148090	AUH	-2,13	0,85

ensembl gene	gene symbol	Score(d)	Fold Change
ENSG00000003436	TFPI	-2,13	0,60
ENSG00000133460	SLC2A11	-2,13	0,81
ENSG00000112237	CCNC	-2,13	0,77
ENSG00000155755	TMEM237	-2,13	0,68
ENSG00000259431	THTPA	-2,13	0,77
ENSG00000183117	CSMD1	-2,13	0,91
ENSG00000198000	NOL8	-2,13	0,82
ENSG00000205981	DNAJC19	-2,12	0,75
ENSG00000113845	TIMMDC1	-2,12	0,73
ENSG00000242715	CCDC169	-2,12	0,81
ENSG00000005884	ITGA3	-2,12	0,81
ENSG00000063241	ISOC2	-2,12	0,78
ENSG00000204351	SKIV2L	-2,12	0,84
ENSG00000106799	TGFBR1	-2,12	0,74
ENSG00000182768	NGRN	-2,12	0,84
ENSG00000181752	OR8K5	-2,12	0,89
ENSG00000213654	GPSM3	-2,12	0,78
ENSG00000171017	LRRC8E	-2,12	0,88
ENSG00000108684	ASIC2	-2,12	0,83
ENSG00000142405	NLRP12	-2,12	0,84
ENSG00000164756	SLC30A8	-2,12	0,81
ENSG00000188186	LAMTOR4	-2,12	0,81
ENSG00000147443	DOK2	-2,12	0,75
ENSG00000149256	TENM4	-2,11	0,73
ENSG00000140526	ABHD2	-2,11	0,75
ENSG00000141200	KIF2B	-2,11	0,91
ENSG00000108846	ABCC3	-2,11	0,81
ENSG00000155275	TRMT44	-2,11	0,83
ENSG00000173406	DAB1	-2,11	0,85
ENSG00000165810	BTNL9	-2,11	0,87
ENSG00000160679	CHTOP	-2,11	0,77
ENSG00000260325	HSPB9	-2,11	0,90
ENSG00000236383	LINC00854	-2,11	0,83
ENSG00000166886	NAB2	-2,11	0,82
ENSG00000167766	ZNF83	-2,11	0,51
ENSG00000144278	GALNT13	-2,11	0,67
ENSG00000258223	PRSS58	-2,11	0,90
ENSG00000113391	FAM172A	-2,11	0,81
ENSG00000117971	CHRNB4	-2,11	0,81
ENSG00000134882	UBAC2	-2,10	0,76
ENSG00000148824	MTG1	-2,10	0,80
ENSG00000204120	GIGYF2	-2,10	0,86
ENSG00000196159	FAT4	-2,10	0,59
ENSG00000187987	ZSCAN23	-2,10	0,83
ENSG00000163803	PLB1	-2,10	0,79
ENSG00000177947	ODF3	-2,10	0,88
ENSG00000119929	CUTC	-2,10	0,85
ENSG00000136478	TEX2	-2,10	0,79
ENSG00000166002	SMCO4	-2,10	0,79

ensembl gene	gene symbol	Score(d)	Fold Change
ENSG00000140285	FGF7	-2,10	0,76
ENSG00000143322	ABL2	-2,10	0,80
ENSG00000108557	RAI1	-2,10	0,85
ENSG00000159761	C16orf86	-2,10	0,84
ENSG00000139974	SLC38A6	-2,10	0,73
ENSG00000110435	PDHX	-2,10	0,82
ENSG00000137265	IRF4	-2,10	0,75
ENSG00000182600	C2orf82	-2,10	0,85
ENSG00000023516	AKAP11	-2,09	0,75
ENSG00000137078	SIT1	-2,09	0,69
ENSG00000165323	FAT3	-2,09	0,86
ENSG00000111707	SUDS3	-2,09	0,80
ENSG00000136881	BAAT	-2,09	0,87
ENSG00000085998	POMGNT1	-2,09	0,77
ENSG00000162592	CCDC27	-2,09	0,87
ENSG00000197296	FITM2	-2,09	0,73
ENSG00000124164	VAPB	-2,09	0,80
ENSG00000105852	PON3	-2,09	0,67
ENSG00000078061	ARAF	-2,09	0,86
ENSG00000131634	TMEM204	-2,09	0,87
ENSG00000198752	CDC42BPB	-2,09	0,83
ENSG00000169116	PARM1	-2,09	0,58
ENSG00000180592	SKIDA1	-2,09	0,88
ENSG00000164252	AGGF1	-2,09	0,83
ENSG00000153789	FAM92B	-2,08	0,82
ENSG00000102057	KCND1	-2,08	0,84
ENSG00000171914	TLN2	-2,08	0,77
ENSG00000188060	RAB42	-2,08	0,82
ENSG00000137571	SLCO5A1	-2,08	0,58
ENSG00000165059	PRKACG	-2,08	0,86
ENSG00000122783	C7orf49	-2,08	0,83
ENSG00000111640	GAPDH	-2,08	0,81
ENSG00000119335	SET	-2,08	0,79
ENSG00000198722	UNC13B	-2,08	0,74
ENSG00000170734	POLH	-2,08	0,67
ENSG00000168411	RFWD3	-2,08	0,79
ENSG00000198856	OSTC	-2,08	0,83
ENSG00000168081	PNOC	-2,08	0,71
ENSG00000085063	CD59	-2,08	0,72
ENSG00000165115	KIF27	-2,07	0,82
ENSG00000180370	PAK2	-2,07	0,58
ENSG00000154639	CXADR	-2,07	0,57
ENSG00000127564	PKMYT1	-2,07	0,79
ENSG00000116584	ARHGEF2	-2,07	0,62
ENSG00000151348	EXT2	-2,07	0,79
ENSG00000135451	TROAP	-2,07	0,79
ENSG00000119899	SLC17A5	-2,07	0,76
ENSG00000062370	ZNF112	-2,07	0,73
ENSG00000177469	PTRF	-2,07	0,88

ensembl gene	gene symbol	Score(d)	Fold Change
ENSG00000094963	FMO2	-2,07	0,85
ENSG00000183808	RBM12B	-2,07	0,77
ENSG00000112578	BYSL	-2,07	0,78
ENSG00000223865	HLA-DPB1	-2,07	0,65
ENSG00000164070	HSPA4L	-2,07	0,53
ENSG00000160007	ARHGAP35	-2,07	0,81
ENSG00000121377	TAS2R7	-2,07	0,89
ENSG00000178026	LRRC75B	-2,07	0,86
ENSG00000116044	NFE2L2	-2,07	0,82
ENSG00000179562	GCC1	-2,07	0,86
ENSG00000117475	BLZF1	-2,07	0,71
ENSG00000102780	DGKH	-2,06	0,71
ENSG00000103168	TAF1C	-2,06	0,87
ENSG00000108641	B9D1	-2,06	0,73
ENSG00000162692	VCAM1	-2,06	0,66
ENSG00000105401	CDC37	-2,06	0,88
ENSG00000172296	SPTLC3	-2,06	0,76
ENSG00000183313	OR52L1	-2,06	0,75
ENSG00000112699	GMDS	-2,06	0,76
ENSG00000136100	VPS36	-2,06	0,78
ENSG00000184611	KCNH7	-2,06	0,86
ENSG00000144559	TAMM41	-2,06	0,78
ENSG00000147573	TRIM55	-2,06	0,63
ENSG00000147576	ADHFE1	-2,06	0,75
ENSG00000103707	MTFMT	-2,06	0,73
ENSG00000109065	NAT9	-2,06	0,81
ENSG00000182636	NDN	-2,06	0,81
ENSG00000169442	CD52	-2,06	0,79
ENSG00000106012	IQCE	-2,06	0,79
ENSG00000122786	CALD1	-2,06	0,67
ENSG00000174238	PITPNA	-2,06	0,83
ENSG00000183856	IQGAP3	-2,06	0,84
ENSG00000132026	RTBDN	-2,06	0,87
ENSG00000106617	PRKAG2	-2,06	0,82
ENSG00000108001	EBF3	-2,06	0,84
ENSG00000076826	CAMSAP3	-2,06	0,76
ENSG00000119630	PGF	-2,06	0,85
ENSG00000133835	HSD17B4	-2,06	0,70
ENSG00000165181	C9orf84	-2,06	0,90
ENSG00000205250	E2F4	-2,06	0,85
ENSG00000138472	GUCA1C	-2,05	0,77
ENSG00000075643	MOCOS	-2,05	0,48
ENSG00000118402	ELOVL4	-2,05	0,48
ENSG00000115525	ST3GAL5	-2,05	0,67
ENSG00000130741	EIF2S3	-2,05	0,79
ENSG00000155465	SLC7A7	-2,05	0,68
ENSG00000108590	MED31	-2,05	0,74
ENSG00000114902	SPCS1	-2,05	0,89
ENSG00000075624	ACTB	-2,05	0,71

ensembl gene	gene symbol	Score(d)	Fold Change
ENSG00000250305	KIAA1456	-2,05	0,61
ENSG00000165509	MAGEC3	-2,05	0,58
ENSG00000186115	CYP4F2	-2,05	0,77
ENSG00000116017	ARID3A	-2,05	0,78
ENSG00000168016	TRANK1	-2,05	0,65
ENSG00000168646	AXIN2	-2,05	0,76
ENSG00000172264	MACROD2	-2,05	0,75
ENSG00000111669	TPI1	-2,05	0,85
ENSG00000130830	MPP1	-2,05	0,73
ENSG00000236320	SLFN14	-2,04	0,86
ENSG00000166199	ALKBH3	-2,04	0,84
ENSG00000172159	FRMD3	-2,04	0,85
ENSG00000198829	SUCNR1	-2,04	0,87
ENSG00000138061	CYP1B1	-2,04	0,83
ENSG00000214050	FBXO16	-2,04	0,80
ENSG00000185483	ROR1	-2,04	0,70
ENSG00000183833	MAATS1	-2,04	0,55
ENSG00000172273	HINFP	-2,04	0,78
ENSG00000162843	WDR64	-2,04	0,83
ENSG00000269343	ZNF587B	-2,04	0,83
ENSG00000158623	COPG2	-2,04	0,80
ENSG00000146350	TBC1D32	-2,04	0,62
ENSG00000147454	SLC25A37	-2,04	0,76
ENSG00000070770	CSNK2A2	-2,04	0,83
ENSG00000145012	LPP	-2,04	0,73
ENSG00000184454	NCMAP	-2,04	0,62
ENSG00000185130	HIST1H2BL	-2,04	0,69
ENSG00000099250	NRP1	-2,04	0,60
ENSG00000155966	AFF2	-2,04	0,56
ENSG00000104723	TUSC3	-2,04	0,63
ENSG00000163534	FCRL1	-2,04	0,62
ENSG00000177839	PCDHB9	-2,04	0,77
ENSG00000196670	ZFP62	-2,04	0,78
ENSG00000265190	ANXA8	-2,04	0,85
ENSG00000122254	HS3ST2	-2,04	0,82
ENSG00000163932	PRKCD	-2,04	0,76
ENSG00000143457	GOLPH3L	-2,04	0,66
ENSG00000162775	RBM15	-2,04	0,79
ENSG00000166451	CENPN	-2,04	0,69
ENSG00000137135	ARHGEF39	-2,04	0,81
ENSG00000002726	AOC1	-2,04	0,70
ENSG00000079739	PGM1	-2,03	0,78
ENSG00000170035	UBE2E3	-2,03	0,68
ENSG00000197037	ZSCAN25	-2,03	0,82
ENSG00000105204	DYRK1B	-2,03	0,82
ENSG00000165246	NLGN4Y	-2,03	0,52
ENSG00000180806	HOXC9	-2,03	0,89
ENSG00000162598	C1orf87	-2,03	0,80
ENSG00000124786	SLC35B3	-2,03	0,79

ensembl gene	gene symbol	Score(d)	Fold Change
ENSG00000125207	PIWIL1	-2,03	0,86
ENSG00000134201	GSTM5	-2,03	0,82
ENSG00000167515	TRAPPC2L	-2,03	0,78
ENSG00000153250	RBMS1	-2,03	0,75
ENSG00000165494	PCF11	-2,03	0,81
ENSG00000275066	SYNRG	-2,03	0,79
ENSG00000159784	FAM131B	-2,03	0,85
ENSG00000174469	CNTNAP2	-2,03	0,84
ENSG00000149084	HSD17B12	-2,03	0,82
ENSG00000165131	C7orf34	-2,03	0,88
ENSG00000279906	AP002962.1	-2,03	0,89
ENSG00000096654	ZNF184	-2,03	0,78
ENSG00000134905	CARS2	-2,03	0,84
ENSG00000105889	STEAP1B	-2,03	0,72
ENSG00000139718	SETD1B	-2,02	0,81
ENSG00000165695	AK8	-2,02	0,82
ENSG00000172365	OR5B2	-2,02	0,87
ENSG00000129103	SUMF2	-2,02	0,80
ENSG00000124074	ENKD1	-2,02	0,86
ENSG00000181143	MUC16	-2,02	0,91
ENSG00000174292	TNK1	-2,02	0,88
ENSG00000108784	NAGLU	-2,02	0,66
ENSG00000187951	ARHGAP11B	-2,02	0,70
ENSG00000117394	SLC2A1	-2,02	0,80
ENSG00000110057	UNC93B1	-2,02	0,76
ENSG00000128383	APOBEC3A	-2,02	0,68
ENSG00000181481	RNF135	-2,02	0,84
ENSG00000138738	PRDM5	-2,02	0,53
ENSG00000167740	CYB5D2	-2,02	0,81
ENSG00000176209	SMIM19	-2,02	0,76
ENSG00000081087	OSTM1	-2,02	0,76
ENSG00000131142	CCL25	-2,02	0,85
ENSG00000157111	TMEM171	-2,02	0,78
ENSG00000118849	RARRES1	-2,02	0,88
ENSG00000033627	ATP6V0A1	-2,02	0,74
ENSG00000105136	ZNF419	-2,02	0,78
ENSG00000119392	GLE1	-2,02	0,84
ENSG00000064270	ATP2C2	-2,02	0,84
ENSG00000140379	BCL2A1	-2,02	0,71
ENSG00000010932	FMO1	-2,02	0,81
ENSG00000127920	GNG11	-2,01	0,69
ENSG00000160208	RRP1B	-2,01	0,84
ENSG00000203661	OR2T5	-2,01	0,85
ENSG00000197465	GYPE	-2,01	0,73
ENSG00000162711	NLRP3	-2,01	0,84
ENSG00000179930	ZNF648	-2,01	0,90
ENSG00000169738	DCXR	-2,01	0,77
ENSG00000112576	CCND3	-2,01	0,72
ENSG00000179869	ABCA13	-2,01	0,82

Supplementary Table S3. List of 628 specific differentially expressed coding genes in 7 t(11;14)-pPCL compared to 12 t(11;14)-MM cases. Transcripts are ordered according to SAM (d) score.

ensembl gene	gene symbol	Score(d)	Fold Change
ENSG00000078043	PIAS2	4,4	1,98
ENSG00000165280	VCP	4,21	1,44
ENSG00000196705	ZNF431	3,92	1,72
ENSG00000088320	REM1	3,8	1,31
ENSG00000197647	ZNF433	3,61	4,22
ENSG00000177432	NAP1L5	3,53	1,44
ENSG00000166479	TMX3	3,53	1,69
ENSG00000099624	ATP5D	3,41	1,59
ENSG00000155622	XAGE2	3,39	1,36
ENSG00000154518	ATP5G3	3,31	1,76
ENSG00000103489	XYLT1	3,28	4,55
ENSG00000197969	VPS13A	3,27	1,62
ENSG00000078596	ITM2A	3,26	2,02
ENSG00000205189	ZBTB10	3,26	3,05
ENSG00000255302	EID1	3,24	1,57
ENSG00000169567	HINT1	3,23	1,5
ENSG00000104894	CD37	3,22	2,87
ENSG00000266412	NCOA4	3,22	2,15
ENSG00000005243	COPZ2	3,2	2,59
ENSG00000214194	LINC00998	3,19	1,82
ENSG00000116183	PAPPA2	3,19	1,32
ENSG00000214954	LRRC69	3,15	1,59
ENSG00000123144	C19orf43	3,14	1,7
ENSG00000046774	MAGEC2	3,12	1,53
ENSG00000204387	C6orf48	3,11	1,85
ENSG00000157322	CLEC18A	3,1	1,36
ENSG00000115170	ACVR1	3,08	1,8
ENSG00000031823	RANBP3	3,07	1,24
ENSG00000196660	SLC30A10	3,04	1,25
ENSG00000113758	DBN1	3,03	2,01
ENSG00000101670	LIPG	3	1,8
ENSG00000168539	CHRM1	2,98	1,28
ENSG00000183747	ACSM2A	2,97	1,61
ENSG00000171987	C11orf40	2,97	1,22
ENSG00000158715	SLC45A3	2,95	1,4
ENSG00000109193	SULT1E1	2,94	1,39
ENSG00000164172	MOCS2	2,93	1,99
ENSG00000137100	DCTN3	2,92	1,82
ENSG00000186480	INSIG1	2,92	2,06
ENSG00000184162	NR2C2AP	2,92	1,5
ENSG00000187231	SESTD1	2,92	4,43
ENSG00000163898	LIPH	2,91	3,62
ENSG00000156697	UTP14A	2,89	1,4
ENSG00000171130	ATP6V0E2	2,86	1,51
ENSG00000261341	CTD-2568A17.1	2,86	1,44

ensembl gene	gene symbol	Score(d)	Fold Change
ENSG00000129654	FOXJ1	2,85	1,46
ENSG00000176294	OR4N2	2,85	2,07
ENSG00000101911	PRPS2	2,84	1,91
ENSG00000178498	DTX3	2,83	1,77
ENSG00000102195	GPR50	2,83	1,3
ENSG00000075413	MARK3	2,83	1,31
ENSG00000159261	CLDN14	2,79	2,06
ENSG00000168724	DNAJC21	2,79	1,33
ENSG00000081014	AP4E1	2,78	1,42
ENSG00000117984	CTSD	2,77	1,41
ENSG00000137824	RMDN3	2,77	1,31
ENSG00000165164	CFAP47	2,72	1,77
ENSG00000062524	LTK	2,72	1,91
ENSG00000171295	ZNF440	2,72	1,42
ENSG00000283526	RP11-40A7.2	2,69	1,31
ENSG00000159579	RSPRY1	2,69	1,58
ENSG00000197790	OR52M1	2,68	1,33
ENSG00000164647	STEAP1	2,66	1,64
ENSG00000033050	ABCF2	2,64	1,43
ENSG00000204520	MICA	2,64	1,56
ENSG00000148335	NTMT1	2,64	1,43
ENSG00000021355	SERPINB1	2,64	1,53
ENSG00000150456	EEF1AKMT1	2,63	1,34
ENSG00000185085	INTS5	2,63	1,48
ENSG00000157800	SLC37A3	2,63	2,05
ENSG00000105290	APLP1	2,62	1,23
ENSG00000138028	CGREF1	2,62	1,36
ENSG00000132286	TIMM10B	2,62	1,47
ENSG00000134982	APC	2,61	1,58
ENSG00000136717	BIN1	2,61	1,75
ENSG00000152223	EPG5	2,61	1,31
ENSG00000180573	HIST1H2AC	2,6	2,31
ENSG00000179055	OR13D1	2,6	1,4
ENSG00000183048	SLC25A10	2,6	1,3
ENSG00000133020	MYH8	2,59	1,39
ENSG00000115758	ODC1	2,59	1,41
ENSG00000135976	ANKRD36	2,58	1,96
ENSG00000141161	UNC45B	2,58	1,17
ENSG00000130755	GMFG	2,56	1,75
ENSG00000090266	NDUFB2	2,56	1,43
ENSG00000099785	MARCHF2	2,55	1,36
ENSG00000137831	UACA	2,55	1,64
ENSG00000147869	CER1	2,54	1,26
ENSG00000166869	CHP2	2,54	1,28
ENSG00000174137	FAM53A	2,54	1,27
ENSG00000048162	NOP16	2,54	1,98
ENSG00000128040	SPINK2	2,54	1,37
ENSG00000132467	UTP3	2,54	1,64
ENSG00000221878	PSG7	2,52	1,31

ensembl gene	gene symbol	Score(d)	Fold Change
ENSG00000139194	RBP5	2,52	1,27
ENSG00000167210	LOXHD1	2,51	1,2
ENSG00000163069	SGCB	2,51	3,64
ENSG00000148835	TAF5	2,51	1,44
ENSG00000127580	WDR24	2,51	1,26
ENSG00000008277	ADAM22	2,5	2,28
ENSG00000183644	C11orf88	2,5	1,28
ENSG00000242110	AMACR	2,49	1,45
ENSG00000104818	CGB2	2,49	1,33
ENSG00000165259	HDX	2,49	1,57
ENSG00000127530	OR7C1	2,49	1,36
ENSG00000171954	CYP4F22	2,48	1,18
ENSG00000086288	NME8	2,48	1,65
ENSG00000085741	WNT11	2,48	1,46
ENSG00000116783	TNNI3K	2,47	1,84
ENSG00000120215	MLANA	2,45	1,21
ENSG00000111046	MYF6	2,45	2,31
ENSG00000237515	SHISA9	2,45	1,3
ENSG00000067248	DHX29	2,44	1,38
ENSG00000181218	HIST3H2A	2,44	2,22
ENSG00000135972	MRPS9	2,44	1,5
ENSG00000105982	RNF32	2,44	1,71
ENSG00000167112	TRUB2	2,44	1,54
ENSG00000170128	GPR25	2,43	1,28
ENSG00000069424	KCNAB2	2,43	1,41
ENSG00000173464	RNASE11	2,43	1,96
ENSG00000118707	TGIF2	2,43	1,6
ENSG00000132846	ZBED3	2,43	1,57
ENSG00000169594	BNC1	2,42	1,19
ENSG00000181215	C4orf50	2,42	1,2
ENSG00000283697	RP5-937E21.8	2,42	1,32
ENSG00000146755	TRIM50	2,42	1,39
ENSG00000188761	BCL2L15	2,41	1,62
ENSG00000197603	C5orf42	2,41	1,57
ENSG00000163749	CCDC158	2,41	1,82
ENSG00000158315	RHBDL2	2,41	1,28
ENSG00000120725	SIL1	2,41	1,59
ENSG00000006042	TMEM98	2,41	1,43
ENSG00000213973	ZNF99	2,41	1,29
ENSG00000113318	MSH3	2,4	1,32
ENSG00000196368	NUDT11	2,4	1,63
ENSG00000166743	ACSM1	2,39	1,43
ENSG00000140471	LINS1	2,39	1,48
ENSG00000166557	TMED3	2,39	1,69
ENSG00000174963	ZIC4	2,39	1,24
ENSG00000197410	DCHS2	2,38	1,27
ENSG00000213160	KLHL23	2,38	1,96
ENSG00000105085	MED26	2,38	1,67
ENSG00000029725	RABEP1	2,38	1,63

ensembl gene	gene symbol	Score(d)	Fold Change
ENSG00000214842	RAD51AP2	2,38	1,14
ENSG00000149050	ZNF214	2,38	1,27
ENSG00000086289	EPDR1	2,37	2,01
ENSG00000082516	GEMIN5	2,37	1,7
ENSG00000113580	NR3C1	2,37	1,54
ENSG00000138315	OIT3	2,37	1,3
ENSG00000241241	KRTAP4-16	2,36	1,54
ENSG00000125841	NRSN2	2,36	1,59
ENSG00000241128	OR14A2	2,36	1,46
ENSG00000176406	RIMS2	2,36	3,4
ENSG00000166582	CENPV	2,35	1,7
ENSG00000166411	IDH3A	2,35	1,35
ENSG00000186329	TMEM212	2,35	1,46
ENSG00000173335	CST9	2,34	1,21
ENSG00000196890	HIST3H2BB	2,34	1,68
ENSG00000109805	NCAPG	2,34	2,12
ENSG00000168143	FAM83B	2,33	1,23
ENSG00000069764	PLA2G10	2,33	2,1
ENSG00000227500	SCAMP4	2,33	1,48
ENSG00000172031	EPHX4	2,32	1,43
ENSG00000108479	GALK1	2,32	1,21
ENSG00000169856	ONECUT1	2,31	1,28
ENSG00000004975	DVL2	-2,14	0,75
ENSG00000165457	FOLR2	-2,14	0,65
ENSG00000140678	ITGAX	-2,14	0,61
ENSG00000126773	PCNX4	-2,14	0,59
ENSG00000179902	C1orf194	-2,15	0,83
ENSG00000105479	CCDC114	-2,15	0,75
ENSG00000196505	GDAP2	-2,15	0,71
ENSG00000197584	KCNMB2	-2,15	0,36
ENSG00000104643	MTMR9	-2,15	0,69
ENSG00000071909	MYO3B	-2,15	0,38
ENSG00000116690	PRG4	-2,15	0,72
ENSG00000170989	S1PR1	-2,15	0,41
ENSG00000160055	TMEM234	-2,15	0,76
ENSG00000198894	CIPC	-2,16	0,73
ENSG00000126456	IRF3	-2,16	0,69
ENSG00000143353	LYPLAL1	-2,16	0,62
ENSG00000132688	NES	-2,16	0,43
ENSG00000112033	PPARD	-2,16	0,74
ENSG00000103769	RAB11A	-2,16	0,73
ENSG00000215343	ZNF705D	-2,16	0,8
ENSG00000150967	ABCB9	-2,17	0,6
ENSG00000001629	ANKIB1	-2,17	0,67
ENSG00000196372	ASB13	-2,17	0,7
ENSG00000168772	CXXC4	-2,17	0,4
ENSG00000178852	EFCAB13	-2,17	0,63
ENSG00000082482	KCNK2	-2,17	0,81
ENSG00000197879	MYO1C	-2,17	0,65

ensembl gene	gene symbol	Score(d)	Fold Change
ENSG00000197428	OR51D1	-2,17	0,72
ENSG00000131788	PIAS3	-2,17	0,74
ENSG00000132424	PNISR	-2,17	0,77
ENSG00000168447	SCNN1B	-2,17	0,42
ENSG00000078269	SYNJ2	-2,17	0,75
ENSG00000204186	ZDBF2	-2,17	0,62
ENSG00000100246	DNAL4	-2,18	0,65
ENSG00000100292	HMOX1	-2,18	0,51
ENSG00000149243	KLHL35	-2,18	0,68
ENSG00000184384	MAML2	-2,18	0,57
ENSG00000156738	MS4A1	-2,18	0,15
ENSG00000100330	MTMR3	-2,18	0,64
ENSG00000177479	ARIH2	-2,19	0,78
ENSG00000162819	BROX	-2,19	0,76
ENSG00000159189	C1QC	-2,19	0,48
ENSG00000108091	CCDC6	-2,19	0,48
ENSG00000143776	CDC42BPA	-2,19	0,7
ENSG00000165959	CLMN	-2,19	0,61
ENSG00000152207	CYSLTR2	-2,19	0,42
ENSG00000174989	FBXW8	-2,19	0,59
ENSG00000169683	LRRC45	-2,19	0,71
ENSG00000103150	MLYCD	-2,19	0,71
ENSG00000141905	NFIC	-2,19	0,77
ENSG00000046651	OFD1	-2,19	0,72
ENSG00000135093	USP30	-2,19	0,7
ENSG00000152213	ARL11	-2,2	0,66
ENSG00000139624	CERS5	-2,2	0,71
ENSG00000064961	HMG20B	-2,2	0,71
ENSG00000123411	IKZF4	-2,2	0,65
ENSG00000145414	NAF1	-2,2	0,72
ENSG00000171649	ZIK1	-2,2	0,57
ENSG00000268089	GABRQ	-2,21	0,84
ENSG00000154478	GPR26	-2,21	0,84
ENSG00000145979	TBC1D7	-2,21	0,62
ENSG00000169905	TOR1AIP2	-2,21	0,75
ENSG00000178764	ZHX2	-2,21	0,72
ENSG00000113441	LNPEP	-2,22	0,54
ENSG00000100023	PPIL2	-2,22	0,73
ENSG00000132716	DCAF8	-2,23	0,65
ENSG00000148180	GSN	-2,23	0,59
ENSG00000196576	PLXNB2	-2,23	0,57
ENSG00000157985	AGAP1	-2,24	0,5
ENSG00000166825	ANPEP	-2,24	0,59
ENSG00000145982	FARS2	-2,24	0,63
ENSG00000178719	GRINA	-2,24	0,67
ENSG00000140443	IGF1R	-2,24	0,55
ENSG00000187193	MT1X	-2,24	0,4
ENSG00000100228	RAB36	-2,24	0,48
ENSG00000059804	SLC2A3	-2,24	0,49

ensembl gene	gene symbol	Score(d)	Fold Change
ENSG00000176994	SMCR8	-2,24	0,73
ENSG00000178917	ZNF852	-2,24	0,71
ENSG00000166669	ATF7IP2	-2,25	0,4
ENSG00000043039	BARX2	-2,25	0,57
ENSG00000164597	COG5	-2,25	0,67
ENSG00000166503	RP11-382A20.3	-2,25	0,81
ENSG00000139874	SSTR1	-2,25	0,79
ENSG00000197782	ZNF780A	-2,25	0,64
ENSG00000182183	FAM159A	-2,26	0,53
ENSG00000108306	FBXL20	-2,26	0,64
ENSG00000168310	IRF2	-2,26	0,52
ENSG00000116962	NID1	-2,26	0,77
ENSG00000143811	PYCR2	-2,26	0,59
ENSG00000157216	SSBP3	-2,26	0,7
ENSG00000136295	TTYH3	-2,26	0,71
ENSG00000035403	VCL	-2,26	0,62
ENSG00000173210	ABLIM3	-2,27	0,78
ENSG00000158435	CNOT11	-2,27	0,81
ENSG00000175592	FOSL1	-2,27	0,79
ENSG00000138944	KIAA1644	-2,27	0,76
ENSG00000213626	LBH	-2,27	0,68
ENSG00000156463	SH3RF2	-2,27	0,6
ENSG00000111348	ARHGDIB	-2,28	0,73
ENSG00000167861	HID1	-2,28	0,59
ENSG00000175536	LIPT2	-2,28	0,74
ENSG00000131368	MRPS25	-2,28	0,75
ENSG00000122728	TAF1L	-2,28	0,79
ENSG00000154945	ANKRD40	-2,29	0,74
ENSG00000073598	FNDC8	-2,29	0,88
ENSG00000135423	GLS2	-2,29	0,75
ENSG00000163875	MEAF6	-2,29	0,74
ENSG00000100600	LGMN	-2,3	0,41
ENSG00000162148	PPP1R32	-2,3	0,81
ENSG00000079841	RIMS1	-2,3	0,39
ENSG00000137210	TMEM14B	-2,3	0,7
ENSG00000158201	ABHD3	-2,31	0,68
ENSG00000099814	CEP170B	-2,31	0,69
ENSG00000112679	DUSP22	-2,31	0,6
ENSG00000163545	NUAK2	-2,31	0,52
ENSG00000115020	PIKFYVE	-2,31	0,65
ENSG00000100218	RSPH14	-2,31	0,66
ENSG00000162722	TRIM58	-2,31	0,78
ENSG00000115446	UNC50	-2,31	0,73
ENSG00000166432	ZMAT1	-2,31	0,46
ENSG00000143621	ILF2	-2,32	0,79
ENSG00000073169	SELO	-2,32	0,76
ENSG00000185669	SNAI3	-2,32	0,75
ENSG00000173890	GPR160	-2,33	0,53
ENSG00000105650	PDE4C	-2,33	0,78

ensembl gene	gene symbol	Score(d)	Fold Change
ENSG00000130024	PHF10	-2,33	0,71
ENSG00000149346	SLX4IP	-2,33	0,58
ENSG00000179832	MROH1	-2,34	0,77
ENSG00000169439	SDC2	-2,34	0,26
ENSG00000125835	SNRPB	-2,34	0,62
ENSG00000179085	DPM3	-2,35	0,63
ENSG00000180447	GAS1	-2,35	0,67
ENSG00000164654	MIOS	-2,35	0,57
ENSG00000163877	SNIP1	-2,35	0,62
ENSG00000137801	THBS1	-2,35	0,36
ENSG00000089063	TMEM230	-2,35	0,76
ENSG00000127837	AAMP	-2,36	0,74
ENSG00000106077	ABHD11	-2,36	0,67
ENSG00000013016	EHD3	-2,36	0,4
ENSG00000177138	FAM9B	-2,36	0,77
ENSG00000196126	HLA-DRB1	-2,36	0,34
ENSG00000174943	KCTD13	-2,36	0,78
ENSG00000162511	LAPTM5	-2,36	0,28
ENSG00000180658	OR2A4	-2,36	0,75
ENSG00000204531	POU5F1	-2,36	0,43
ENSG00000088179	PTPN4	-2,36	0,66
ENSG00000187147	RNF220	-2,37	0,75
ENSG00000116497	S100PBP	-2,37	0,61
ENSG00000129657	SEC14L1	-2,37	0,62
ENSG00000072657	TRHDE	-2,37	0,63
ENSG00000135686	KLHL36	-2,38	0,51
ENSG00000063322	MED29	-2,38	0,8
ENSG00000090402	SI	-2,38	0,59
ENSG00000002745	WNT16	-2,38	0,61
ENSG00000178665	ZNF713	-2,38	0,46
ENSG00000260314	MRC1	-2,39	0,7
ENSG00000078177	N4BP2	-2,39	0,63
ENSG00000225973	PIGBOS1	-2,39	0,76
ENSG00000154822	PLCL2	-2,39	0,5
ENSG00000040487	PQLC2	-2,39	0,68
ENSG00000125409	TEKT3	-2,39	0,67
ENSG00000204815	TTC25	-2,39	0,78
ENSG00000130208	APOC1	-2,4	0,58
ENSG00000228439	TSTD3	-2,4	0,63
ENSG00000172466	ZNF24	-2,4	0,72
ENSG00000197070	ARRDC1	-2,41	0,69
ENSG00000178585	CTNNBIP1	-2,41	0,82
ENSG00000115866	DARS	-2,41	0,61
ENSG00000185112	FAM43A	-2,41	0,54
ENSG00000162729	IGSF8	-2,41	0,67
ENSG00000167419	LPO	-2,41	0,63
ENSG00000161664	ASB16	-2,42	0,72
ENSG00000112303	VNN2	-2,42	0,38
ENSG00000185278	ZBTB37	-2,42	0,67

ensembl gene	gene symbol	Score(d)	Fold Change
ENSG00000196569	LAMA2	-2,43	0,28
ENSG00000089022	MAPKAPK5	-2,43	0,76
ENSG00000134744	ZCCHC11	-2,43	0,64
ENSG00000187474	FPR3	-2,44	0,64
ENSG00000213015	ZNF580	-2,44	0,82
ENSG00000221869	CEBPD	-2,45	0,71
ENSG00000133878	DUSP26	-2,45	0,43
ENSG00000100578	KIAA0586	-2,45	0,64
ENSG00000091106	NLRC4	-2,45	0,79
ENSG00000091490	SEL1L3	-2,45	0,56
ENSG00000116525	TRIM62	-2,45	0,64
ENSG00000187815	ZFP69	-2,45	0,65
ENSG00000119725	ZNF410	-2,45	0,43
ENSG00000138175	ARL3	-2,46	0,64
ENSG00000173065	FAM222B	-2,46	0,68
ENSG00000010626	LRRC23	-2,46	0,68
ENSG00000160058	BSDC1	-2,47	0,66
ENSG00000180340	FZD2	-2,47	0,78
ENSG00000107147	KCNT1	-2,47	0,67
ENSG00000185900	POMK	-2,47	0,54
ENSG00000149212	SESN3	-2,47	0,66
ENSG00000106868	SUSD1	-2,47	0,42
ENSG00000116918	TSNAX	-2,47	0,36
ENSG00000163795	ZNF513	-2,47	0,77
ENSG00000104765	BNIP3L	-2,48	0,71
ENSG00000167346	MMP26	-2,48	0,77
ENSG00000188021	UBQLN2	-2,48	0,65
ENSG00000158195	WASF2	-2,48	0,68
ENSG00000100299	ARSA	-2,49	0,59
ENSG00000168763	CNNM3	-2,49	0,73
ENSG00000111652	COPS7A	-2,49	0,65
ENSG00000118260	CREB1	-2,49	0,7
ENSG00000147654	EBAG9	-2,5	0,74
ENSG00000046647	GEMIN8	-2,5	0,65
ENSG00000133818	RRAS2	-2,5	0,2
ENSG00000053770	AP5M1	-2,51	0,71
ENSG00000051523	CYBA	-2,51	0,64
ENSG00000108950	FAM20A	-2,51	0,71
ENSG00000162735	PEX19	-2,51	0,69
ENSG00000196812	ZSCAN16	-2,51	0,65
ENSG00000132906	CASP9	-2,52	0,77
ENSG00000109220	CHIC2	-2,52	0,61
ENSG00000121316	PLBD1	-2,52	0,37
ENSG00000282218	RP1-179P9.3	-2,52	0,51
ENSG00000143252	SDHC	-2,52	0,62
ENSG00000109133	TMEM33	-2,52	0,71
ENSG00000168556	ING2	-2,53	0,73
ENSG00000109436	TBC1D9	-2,53	0,58
ENSG00000188167	TMPPE	-2,53	0,59

ensembl gene	gene symbol	Score(d)	Fold Change
ENSG00000170634	ACYP2	-2,54	0,75
ENSG00000085224	ATRX	-2,54	0,63
ENSG00000173372	C1QA	-2,54	0,53
ENSG00000144677	CTDSPL	-2,54	0,64
ENSG00000213859	KCTD11	-2,54	0,68
ENSG00000090975	PITPNM2	-2,54	0,7
ENSG00000136059	VILL	-2,54	0,76
ENSG00000178562	CD28	-2,55	0,22
ENSG00000133114	GPALPP1	-2,55	0,68
ENSG00000163884	KLF15	-2,55	0,65
ENSG00000075914	EXOSC7	-2,56	0,69
ENSG00000237541	HLA-DQA2	-2,56	0,75
ENSG00000087253	LPCAT2	-2,56	0,37
ENSG00000138593	SECISBP2L	-2,56	0,56
ENSG00000175137	SH3BP5L	-2,56	0,57
ENSG00000135605	TEC	-2,56	0,55
ENSG00000155659	VSIG4	-2,56	0,63
ENSG00000167363	FN3K	-2,57	0,29
ENSG00000011405	PIK3C2A	-2,57	0,63
ENSG00000109099	PMP22	-2,57	0,6
ENSG00000149634	SPATA25	-2,57	0,66
ENSG00000215454	KRTAP10-4	-2,58	0,71
ENSG00000107719	PALD1	-2,58	0,52
ENSG00000120594	PLXDC2	-2,58	0,47
ENSG00000184716	SERINC4	-2,58	0,59
ENSG00000163633	C4orf36	-2,59	0,69
ENSG00000257365	FNTB	-2,59	0,56
ENSG00000135525	MAP7	-2,59	0,41
ENSG00000197943	PLCG2	-2,6	0,59
ENSG00000203943	SAMD13	-2,6	0,54
ENSG00000120088	CRHR1	-2,61	0,57
ENSG00000100105	PATZ1	-2,61	0,67
ENSG00000174695	TMEM167A	-2,61	0,71
ENSG00000184867	ARMCX2	-2,62	0,49
ENSG00000179941	BBS10	-2,62	0,63
ENSG00000163528	CHCHD4	-2,62	0,74
ENSG00000213416	KRTAP4-12	-2,62	0,72
ENSG00000197629	MPEG1	-2,62	0,37
ENSG00000166428	PLD4	-2,62	0,28
ENSG00000009413	REV3L	-2,62	0,63
ENSG00000089775	ZBTB25	-2,62	0,72
ENSG00000164048	ZNF589	-2,62	0,61
ENSG00000126759	CFP	-2,63	0,74
ENSG00000118496	FBXO30	-2,63	0,64
ENSG00000280165	PCDH20	-2,63	0,5
ENSG00000052126	PLEKHA5	-2,63	0,38
ENSG00000013392	RWDD2A	-2,63	0,67
ENSG00000143183	TMCO1	-2,63	0,68
ENSG00000250254	PTTG2	-2,64	0,66

ensembl gene	gene symbol	Score(d)	Fold Change
ENSG00000104450	SPAG1	-2,64	0,57
ENSG00000116194	ANGPTL1	-2,65	0,59
ENSG00000106080	FKBP14	-2,65	0,62
ENSG00000152240	HAUS1	-2,65	0,53
ENSG00000047597	XK	-2,65	0,63
ENSG00000180660	MAB21L1	-2,66	0,5
ENSG00000168026	TTC21A	-2,66	0,61
ENSG00000111666	CHPT1	-2,67	0,45
ENSG00000148730	EIF4EBP2	-2,67	0,69
ENSG00000158711	ELK4	-2,67	0,76
ENSG00000143614	GATAD2B	-2,67	0,73
ENSG00000144401	METTL21A	-2,67	0,54
ENSG00000156467	UQCRB	-2,67	0,7
ENSG00000153048	CARHSP1	-2,68	0,67
ENSG00000187239	FNBP1	-2,68	0,17
ENSG00000178922	HYI	-2,68	0,59
ENSG00000197646	PDCD1LG2	-2,68	0,44
ENSG00000164548	TRA2A	-2,68	0,75
ENSG00000171606	ZNF274	-2,68	0,65
ENSG00000184640	SEPTIN9	-2,68	0,56
ENSG00000101938	CHRDL1	-2,69	0,61
ENSG00000144445	KANSL1L	-2,69	0,44
ENSG00000072042	RDH11	-2,69	0,65
ENSG00000038427	VCAN	-2,69	0,57
ENSG00000135974	C2orf49	-2,7	0,66
ENSG00000135678	CPM	-2,7	0,34
ENSG00000118922	KLF12	-2,7	0,45
ENSG00000175482	POLD4	-2,7	0,56
ENSG00000144792	ZNF660	-2,7	0,32
ENSG00000090659	CD209	-2,71	0,55
ENSG00000196092	PAX5	-2,71	0,51
ENSG00000138777	PPA2	-2,71	0,56
ENSG00000258644	SYNJ2BP-COX16	-2,71	0,51
ENSG00000134717	BTF3L4	-2,72	0,63
ENSG00000011600	TYROBP	-2,72	0,54
ENSG00000111254	AKAP3	-2,73	0,65
ENSG00000111642	CHD4	-2,74	0,77
ENSG00000132199	ENOSF1	-2,74	0,55
ENSG00000185442	FAM174B	-2,74	0,5
ENSG00000158869	FCER1G	-2,74	0,38
ENSG00000182255	KCNA4	-2,74	0,59
ENSG00000112394	SLC16A10	-2,74	0,59
ENSG00000049618	ARID1B	-2,75	0,66
ENSG00000149418	ST14	-2,75	0,31
ENSG00000143258	USP21	-2,75	0,65
ENSG00000185101	ANO9	-2,76	0,58
ENSG00000100632	ERH	-2,76	0,53
ENSG00000143442	POGZ	-2,76	0,66
ENSG00000004468	CD38	-2,77	0,68

ensembl gene	gene symbol	Score(d)	Fold Change
ENSG00000205593	DENND6B	-2,77	0,43
ENSG00000215251	FASTKD5	-2,77	0,62
ENSG00000142599	RERE	-2,77	0,49
ENSG00000139971	C14orf37	-2,78	0,58
ENSG00000116285	ERRFI1	-2,78	0,44
ENSG00000187210	GCNT1	-2,78	0,55
ENSG00000134375	TIMM17A	-2,78	0,54
ENSG00000196998	WDR45	-2,78	0,69
ENSG00000130787	HIP1R	-2,79	0,69
ENSG00000169403	PTAFR	-2,79	0,64
ENSG00000166833	NAV2	-2,8	0,43
ENSG00000095397	WHRN	-2,8	0,4
ENSG00000135338	LCA5	-2,81	0,39
ENSG00000203737	GPR52	-2,83	0,48
ENSG00000170962	PDGFD	-2,83	0,53
ENSG00000117533	VAMP4	-2,83	0,57
ENSG00000160345	C9orf116	-2,84	0,44
ENSG00000185477	GPRIN3	-2,84	0,26
ENSG00000136161	RCBTB2	-2,84	0,49
ENSG00000143013	LMO4	-2,85	0,63
ENSG00000164920	OSR2	-2,85	0,6
ENSG00000182185	RAD51B	-2,85	0,5
ENSG00000187676	B3GLCT	-2,86	0,47
ENSG00000133704	IPO8	-2,87	0,47
ENSG00000135048	TMEM2	-2,87	0,29
ENSG00000135144	DTX1	-2,88	0,38
ENSG00000186951	PPARA	-2,88	0,64
ENSG00000197249	SERPINA1	-2,89	0,47
ENSG00000078140	UBE2K	-2,89	0,65
ENSG00000100445	SDR39U1	-2,91	0,7
ENSG00000168038	ULK4	-2,91	0,51
ENSG00000006740	ARHGAP44	-2,92	0,29
ENSG00000147144	CCDC120	-2,92	0,51
ENSG00000179979	CRIPAK	-2,92	0,6
ENSG00000174206	C12orf66	-2,93	0,71
ENSG00000175470	PPP2R2D	-2,93	0,67
ENSG00000196405	EVL	-2,95	0,51
ENSG00000147316	MCPH1	-2,95	0,66
ENSG00000242114	MTFP1	-2,95	0,55
ENSG00000177732	SOX12	-2,95	0,74
ENSG00000189241	TSPYL1	-2,95	0,71
ENSG00000147408	CSGALNACT1	-2,96	0,37
ENSG00000101384	JAG1	-2,96	0,42
ENSG00000125952	MAX	-2,96	0,68
ENSG00000184905	TCEAL2	-2,96	0,69
ENSG00000090316	MAEA	-2,97	0,73
ENSG00000170525	PFKFB3	-2,97	0,36
ENSG00000102710	SUPT20H	-2,97	0,57
ENSG00000167165	UGT1A6	-2,97	0,62

ensembl gene	gene symbol	Score(d)	Fold Change
ENSG00000118579	MED28	-2,98	0,62
ENSG00000060749	QSER1	-2,98	0,71
ENSG00000124557	BTN1A1	-2,99	0,38
ENSG00000178568	ERBB4	-2,99	0,26
ENSG00000173369	C1QB	-3	0,34
ENSG00000152904	GGPS1	-3	0,61
ENSG00000169499	PLEKHA2	-3	0,54
ENSG00000119685	TTLL5	-3	0,61
ENSG00000127507	ADGRE2	-3,02	0,52
ENSG00000164603	BMT2	-3,02	0,53
ENSG00000160856	FCRL3	-3,02	0,36
ENSG00000116574	RHOU	-3,03	0,63
ENSG00000182578	CSF1R	-3,04	0,52
ENSG00000175471	MCTP1	-3,04	0,54
ENSG00000173209	AHSA2	-3,05	0,67
ENSG00000141441	GAREM1	-3,05	0,22
ENSG00000144560	VGLL4	-3,05	0,51
ENSG00000143603	KCNN3	-3,07	0,43
ENSG00000271605	MILR1	-3,07	0,29
ENSG00000136643	RPS6KC1	-3,09	0,45
ENSG00000065970	FOXJ2	-3,1	0,6
ENSG00000169213	RAB3B	-3,1	0,33
ENSG00000183291	SELENOF	-3,11	0,71
ENSG00000145246	ATP10D	-3,11	0,47
ENSG00000221916	C19orf73	-3,11	0,56
ENSG00000170949	ZNF160	-3,13	0,66
ENSG00000114423	CBLB	-3,14	0,51
ENSG00000101224	CDC25B	-3,14	0,53
ENSG00000110077	MS4A6A	-3,14	0,29
ENSG00000011295	TTC19	-3,14	0,52
ENSG00000137486	ARRB1	-3,15	0,42
ENSG00000125753	VASP	-3,16	0,56
ENSG00000170166	HOXD4	-3,19	0,64
ENSG00000262180	OCLM	-3,19	0,59
ENSG00000174574	AKIRIN1	-3,2	0,69
ENSG00000104529	EEF1D	-3,2	0,7
ENSG00000140525	FANCI	-3,2	0,56
ENSG00000146701	MDH2	-3,21	0,59
ENSG00000155903	RASA2	-3,21	0,51
ENSG00000104671	DCTN6	-3,22	0,69
ENSG00000160255	ITGB2	-3,22	0,53
ENSG00000110079	MS4A4A	-3,23	0,33
ENSG00000166068	SPRED1	-3,23	0,22
ENSG00000122417	ODF2L	-3,24	0,45
ENSG00000125818	PSMF1	-3,25	0,73
ENSG00000139289	PHLDA1	-3,26	0,26
ENSG00000187116	LILRA5	-3,27	0,56
ENSG00000198799	LRIG2	-3,3	0,63
ENSG00000159958	TNFRSF13C	-3,3	0,32

ensembl gene	gene symbol	Score(d)	Fold Change
ENSG00000213020	ZNF611	-3,31	0,47
ENSG00000196712	NF1	-3,32	0,51
ENSG00000198081	ZBTB14	-3,32	0,51
ENSG00000196236	XPNPEP3	-3,33	0,61
ENSG00000173166	RAPH1	-3,34	0,18
ENSG00000129538	RNASE1	-3,35	0,27
ENSG00000140022	STON2	-3,35	0,38
ENSG00000139687	RB1	-3,36	0,39
ENSG00000219626	FAM228B	-3,38	0,58
ENSG00000068971	PPP2R5B	-3,38	0,62
ENSG00000205497	OR51A4	-3,42	0,52
ENSG00000116668	SWT1	-3,43	0,48
ENSG00000100564	PIGH	-3,49	0,65
ENSG00000165028	NIPSNAP3B	-3,52	0,33
ENSG00000181631	P2RY13	-3,52	0,39
ENSG00000132003	ZSWIM4	-3,52	0,57
ENSG00000107669	ATE1	-3,53	0,66
ENSG00000136869	TLR4	-3,53	0,49
ENSG00000181381	DDX60L	-3,55	0,46
ENSG00000157869	RAB28	-3,58	0,55
ENSG00000117020	AKT3	-3,61	0,21
ENSG00000233822	HIST1H2BN	-3,62	0,53
ENSG00000134243	SORT1	-3,62	0,24
ENSG00000205659	LIN52	-3,72	0,53
ENSG00000133800	LYVE1	-3,75	0,22
ENSG00000072121	ZFYVE26	-3,76	0,65
ENSG00000160094	ZNF362	-3,76	0,66
ENSG00000119616	FCF1	-3,79	0,63
ENSG00000254598	CSNK2A3	-3,83	0,55
ENSG00000128218	VPREB3	-3,83	0,12
ENSG00000241058	NSUN6	-3,9	0,51
ENSG00000008226	DLEC1	-3,92	0,51
ENSG00000144791	LIMD1	-3,96	0,43
ENSG00000136715	SAP130	-3,97	0,46
ENSG00000078747	ITCH	-4,01	0,78
ENSG00000131100	ATP6V1E1	-4,19	0,52
ENSG00000197980	LEKR1	-4,25	0,54
ENSG00000139318	DUSP6	-4,45	0,27
ENSG00000006468	ETV1	-4,46	0,07
ENSG00000119688	ABCD4	-4,6	0,58
ENSG00000119669	IRF2BPL	-4,73	0,55
ENSG00000119711	ALDH6A1	-4,84	0,39
ENSG00000119650	IFT43	-4,85	0,55
ENSG00000139132	FGD4	-5,26	0,19

Supplementary Table S4. List of the significantly enriched Annotation Clusters for the 628 differentially expressed protein coding genes (Enrichment Score >1.3) by DAVID 6.8 Bioinformatics tool.

	GO Term	Count	P-Value	Fold Enrichment	Enrichment Score	Genes
**Annotation Cluster 1**	**GO:0044212~transcription regulatory region DNA binding**	40	0,005	1,560	**2,208**	RB1, CEBPD, MAX, CHD4, ARRB1, NR3C1, BARX2, IKZF4, ELK4, WNT11, ERBB4, KANSL1L, PATZ1, ZIK1, OSR2, ZNF660, KLF12, ZBTB14, LMO4, SNAI3, FOXJ2, FOXJ1, ETV1, SOX12, PAX5, KLF15, POU5F1, GATAD2B, FOSL1, CREB1, IRF3, NFIC, IRF2, MYF6, ZNF713, ZNF513, TAF5, PPARA, SSBP3, ZNF431
**Annotation Cluster 2**	**GO:0043167~ion binding**	155	0,012	1,17	**1,883**	ZBTB25, ACSM1, CBLB, MT1X, NR3C1, IKZF4, ZDBF2, NUDT11, RIMS2, RIMS1, ZFYVE26, ANPEP, FNTB, POGZ, CHP2, ACVR1, CSGALNACT1, OSR2, ZNF440, ARSA, PIAS3, ZNF705D, LMO4, ZNF160, APLP1, ZBTB37, EPDR1, ZNF99, SDHC, CYBA, TNNI3K, PIAS2, PPA2, PLA2G10, ZNF713, STEAP1, ZNF433, ZNF431, ZNF274, MTMR3, ARL3, DTX1, AGAP1, LPO, DTX3, ZNF24, CYP4F22, NID1, TRHDE, NUAK2, ZMAT1, DNAJC21, HMOX1, PATZ1, ZSCAN16, ZBED3, ZNF660, JAG1, ZBTB14, PHF10, SNAI3, PCDH20, ZBTB10, LNPEP, FBXO30, TIMM10B, ZFP69, EHD3, SESTD1, DCHS2, CD209, NF1, ZNF410, RNF220, RERE, CPM, ZCCHC11, MCTP1, BNC1, FKBP14, ITGB2, ACSM2A, CHD4, LIMD1, WDR45, FARS2, IRF2BPL, ADGRE2, ING2, MMP26, ENOSF1, REV3L, ITGAX, ARIH2, OIT3, IDH3A, RSPRY1, ZIK1, KLF12, ZNF362, ZHX2, TRIM62, GGPS1, PDE4C, ATRX, CTDSPL, TBC1D9, KLF15, VCAN, PITPNM2, RASA2, TRIM58, RHOU, ZNF513, ZNF780A, PPARA, PAPPA2, PPARD, PRPS2, RNF32, TRIM50, ATP10D, KCNA4, NLRC4, THBS1, CXXC4, FGD4, ABLIM3, ZNF589, ZSWIM4, UGT1A6, TSNAX, CGREF1, GSN, ANKIB1, ZNF580, USP21, SUSD1, HIP1R, PLEKHA5, ZIC4, CDC42BPA, GATAD2B, TMPPE, SH3RF2, PIKFYVE, TEC, XPNPEP3, APOC1, LPCAT2, CHPT1, ZNF611, FOLR2, ZNF214, ZNF852
**Annotation Cluster 3**	**GO:1903214~regulation of protein targeting to mitochondrion**	10	0,004	3,210	**1,859**	BNIP3L, GPR26, ABLIM3, GLS2, ITGAX, ARIH2, SNIP1, RHOU, KCNN3, ZBED3
**Annotation Cluster 4**	**GO:0001228~transcriptional activator activity, RNA polymerase II transcription regulatory region sequence-specific binding**	19	0,009	1,935	**1,731**	OSR2, CEBPD, ONECUT1, FOXJ2, FOXJ1, ETV1, SOX12, PAX5, NR3C1, KLF15, BARX2, FOSL1, CREB1, NFIC, IRF2, MYF6, PATZ1, PPARA, SSBP3

	GO Term	Count	P-Value	Fold Enrichment	Enrichment Score	Genes
**Annotation Cluster 5**	**GO:0042089~cytokine biosynthetic process**	9	0,016	2,755	**1,655**	ERRFI1, IRF3, PTAFR, CD28, HMOX1, FOXJ1, TNFRSF13C, THBS1, TLR4
**Annotation Cluster 6**	**GO:0000978~RNA polymerase II core promoter proximal region sequence-specific DNA binding**	20	0,011	1,865	**1,521**	CEBPD, MAX, SNAI3, FOXJ2, FOXJ1, ETV1, CHD4, PAX5, NR3C1, GATAD2B, FOSL1, CREB1, IRF3, NFIC, MYF6, ZNF713, PATZ1, PPARA, SSBP3, ZNF431
**Annotation Cluster 7**	**GO:0048002~antigen processing and presentation of peptide antigen**	12	0,027	2,114	**1,469**	DCTN6, FCER1G, CD209, DCTN3, LNPEP, CYBA, ABCB9, PSMF1, CTSD, HLA-DQA2, HLA-DRB1, LGMN
**Annotation Cluster 8**	**GO:0090316~positive regulation of intracellular protein transport**	16	0,016	1,970	**1,329**	GPR26, CHRM1, RANBP3, GLS2, POU5F1, MYO1C, ABLIM3, ERBB4, ITGAX, CHP2, ARIH2, SNIP1, RHOU, KCNN3, TLR4, ZBED3

Supplementary Table S5. List of positively (red) or negatively (blue) enriched gene sets in pPCL with t(11;14) compared to MM cases with t(11;14), by GSEA analysis on pre-ranked 628 protein coding gene list. Gene sets (version 7.1) are selected according to nominal p-value (<0.05), and ordered according to Normalized Enrichment Score (NES) in each gene set collection. Size, NES, Nominal p-value, and FDR q-value are reported for each gene set.

NAME	SIZE	NES	NOM p-val	FDR q-val
**HALLMARK**				
HALLMARK_IL2_STAT5_SIGNALING	9	1,741	0,043	0,119
HALLMARK_ANDROGEN_RESPONSE	5	1,576	0,026	0,177
**REACTOME**				
REACTOME_CELL_CYCLE_MITOTIC	15	-2,346	0,000	0,000
REACTOME_CELL_CYCLE	16	-2,199	0,000	0,020
REACTOME_DISEASES_OF_SIGNAL_TRANSDUCTION_BY_GROWTH_FACTOR_RECEPTORS_AND_SECOND_MESSENGERS	17	-1,973	0,016	0,032
**KEGG**				
**C2-Gene Perturbations gene sets selected for "Multiple Myeloma" term**				
MOREAUX_MULTIPLE_MYELOMA_BY_TACI_UP	17	-2,1089	0	0.094
**Gene Ontology (GO): Biological Process (BP), Cellular Component (CC), molecular function (MF) terms**				
GOBP_NEGATIVE_REGULATION_OF_INTRACELLULAR_SIGNAL_TRANSDUCTION	28	-2,646	0,000	0.0
GOBP_POSITIVE_REGULATION_OF_IMMUNE_SYSTEM_PROCESS	46	-2,506	0,000	0.008
GOBP_MAPK_CASCADE	29	-2,342	0,000	0.059
GOBP_REGULATION_OF_PHOSPHORUS_METABOLIC_PROCESS	49	-2,340	0,000	0.047
GOBP_REGULATION_OF_INTRACELLULAR_SIGNAL_TRANSDUCTION	61	-2,326	0,000	0.041
GOBP_NEGATIVE_REGULATION_OF_CELL_DIFFERENTIATION	20	-2,325	0,000	0.035
GOBP_REGULATION_OF_IMMUNE_SYSTEM_PROCESS	54	-2,276	0,000	0.035
GOBP_NEGATIVE_REGULATION_OF_DEVELOPMENTAL_PROCESS	29	-2,275	0,000	0.031
GOBP_POSITIVE_REGULATION_OF_IMMUNE_RESPONSE	31	-2,265	0,000	0.032
GOBP_REGULATION_OF_CELL_ACTIVATION	27	-2,173	0,000	0.043
GOBP_ACTIVATION_OF_IMMUNE_RESPONSE	27	-2,115	0,000	0.060
GOBP_REGULATION_OF_RESPONSE_TO_EXTERNAL_STIMULUS	32	-2,109	0,000	0.057
GOBP_IMMUNE_RESPONSE_REGULATING_SIGNALING_PATHWAY	20	-2,100	0,000	0.055
GOBP_REGULATION_OF_MAPK_CASCADE	26	-2,064	0,000	0.066
GOBP_POSITIVE_REGULATION_OF_PHOSPHORUS_METABOLIC_PROCESS	28	-2,012	0,016	0.081
GOBP_REGULATION_OF_CELL_CELL_ADHESION	15	-1,968	0,000	0.102
GOBP_REGULATION_OF_PROTEIN_PHOSPHORYLATION	35	-1,965	0,000	0.101
GOBP_REGULATION_OF_TRANSFERASE_ACTIVITY	31	-1,960	0,000	0.101
GOBP_REGULATION_OF_ANATOMICAL_STRUCTURE_MORPHOGENESIS	26	-1,925	0,015	0.119
GOBP_REGULATION_OF_PROTEIN_KINASE_ACTIVITY	24	-1,913	0,000	0.125
GOBP_REGULATION_OF_LYMPHOCYTE_ACTIVATION	16	-1,908	0,000	0.126
GOBP_DEFENSE_RESPONSE	52	-1,903	0,014	0.124
GOBP_LEUKOCYTE_CELL_CELL_ADHESION	15	-1,901	0,018	0.124
GOBP_REGULATION_OF_IMMUNE_RESPONSE	35	-1,897	0,000	0.122

NAME	SIZE	NES	NOM p-val	FDR q-val
GOBP_POSITIVE_REGULATION_OF_CELL_POPULATION_PROLIFERATION	31	-1,895	0,000	0.121
GOBP_NEGATIVE_REGULATION_OF_RESPONSE_TO_STIMULUS	61	-1,861	0,015	0.150
GOBP_POSITIVE_REGULATION_OF_RESPONSE_TO_EXTERNAL_STIMULUS	19	-1,854	0,020	0.147
GOBP_NEURON_DIFFERENTIATION	44	-1,836	0,000	0.152
GOBP_REGULATION_OF_DEFENSE_RESPONSE	22	-1,809	0,019	0.174
GOCC_MICROTUBULE_ORGANIZING_CENTER	25	-1,806	0,000	0.171
GOBP_NEUROGENESIS	53	-1,797	0,000	0.177
GOBP_DEFENSE_RESPONSE_TO_OTHER_ORGANISM	34	-1,796	0,017	0.176
GOBP_LEUKOCYTE_PROLIFERATION	15	-1,771	0,030	0.186
GOBP_POSITIVE_REGULATION_OF_INTRACELLULAR_SIGNAL_TRANSDUCTION	32	-1,767	0,016	0.181
GOBP_REGULATION_OF_RESPONSE_TO_STRESS	43	-1,766	0,016	0.178
GOBP_NEGATIVE_REGULATION_OF_CATALYTIC_ACTIVITY	26	-1,759	0,015	0.176
GOBP_POSITIVE_REGULATION_OF_PROTEIN_MODIFICATION_PROCESS	27	-1,751	0,033	0.177
GOBP_INNATE_IMMUNE_RESPONSE	31	-1,750	0,015	0.175
GOBP_ANATOMICAL_STRUCTURE_HOMEOSTASIS	16	-1,747	0,032	0.177
GOBP_CYTOKINE_PRODUCTION	28	-1,745	0,031	0.175
GOBP_PROTEIN_PHOSPHORYLATION	48	-1,737	0,000	0.179
GOBP_POSITIVE_REGULATION_OF_TRANSFERASE_ACTIVITY	19	-1,732	0,034	0.180
GOBP_CELL_MIGRATION	56	-1,731	0,030	0.178
GOBP_RESPONSE_TO_CYTOKINE	33	-1,730	0,000	0.176
GOBP_NEURON_DEVELOPMENT	32	-1,725	0,034	0.180
GOBP_CELL_MORPHOGENESIS	28	-1,718	0,016	0.179
GOBP_INFLAMMATORY_RESPONSE	26	-1,713	0,031	0.181
GOMF_PHOSPHOLIPID_BINDING	24	-1,702	0,033	0.191
GOBP_POSITIVE_REGULATION_OF_CELL_DEATH	26	-1,671	0,029	0.214
GOBP_REGULATION_OF_VESICLE_MEDIATED_TRANSPORT	21	-1,665	0,016	0.215
GOCC_CENTROSOME	17	-1,655	0,050	0.221
GOCC_CELL_LEADING_EDGE	19	-1,653	0,046	0.221
GOBP_CELL_CELL_ADHESION	24	-1,652	0,000	0.219
GOBP_CIRCULATORY_SYSTEM_DEVELOPMENT	38	-1,626	0,000	0.232
GOBP_REGULATION_OF_RESPONSE_TO_BIOTIC_STIMULUS	15	-1,621	0,049	0.231
GOBP_POSITIVE_REGULATION_OF_PROTEIN_PHOSPHORYLATION	17	-1,603	0,036	0.240
GOBP_POSITIVE_REGULATION_OF_MAPK_CASCADE	19	-1,597	0,016	0.24
GOMF_ENZYME_BINDING	70	-1,591	0,043	0.24
GOBP_CHEMICAL_HOMEOSTASIS	29	2,162	0,000	0.0

Supplementary Table S6. Selected gene sets (shown in Figure 2) obtained using Gene Set Enrichment Analysis on the list of 628 DE protein-coding genes that were ranked based on fold change values (NES >1.5 or < -1.5; p-value <0.05, NES: Normalized Enrichment Score).

NAME	SIZE	NES	NOM p-val	gene list*
**GOBP_REGULATION_OF_CELL_CELL_ADHESION**	15	-1.96	0.000	FOXJ1, HLA-DRB1, LAPTM5, **CD28, VSIG4, PDCD1LG2, CD209, PTAFR, DTX1, PPARA, SOX12, JAG1, CBLB, ITGB2, TNFRSF13C**
**GOBP_REGULATION_OF_RESPONSE_TO_EXTERNAL_STIMULUS**	32	-2.10	0.0000	MICA, S1PR1, PPARD, IRF3, LGMN, **THBS1, HLA-DRB1, SEC14L1, ZNF580, NLRC4, MMP26, CYBA, CD28, VSIG4, FNTB, PLCG2, CD209, TYROBP, FCER1G, PDGFD, PPARA, UBE2K, MCPH1, CSF1R, AKIRIN1, PSMF1, LILRA5, LRIG2, RB1, ZSWIM4, TLR4, ITCH**
**GOBP_NEGATIVE_REGULATION_OF_CELL_DIFFERENTIATIONE**	20	-2.32	0.0000	INSIG1, FOXJ1, TMEM98, PPARD, PIAS3, C1QC, ZHX2, TRIM62, **DTX1, PPARA, JAG1, MED28, FOXJ2, AKIRIN1, SPRED1, NF1, RB1, TLR4, SORT1, LIMD1**
**REACTOME_CELL_CYCLE_MITOTIC**	15	-2.34	0.0000	DCTN3, NCAPG, OFD1, HAUS1, POLD4, **PPP2R2D, MCPH1, MAX, CDC25B, PSMF1, PHLDA1, RB1, PPP2R5B, AKT3, LIN52**
**HALLMARK_IL2_STAT5_SIGNALING**	9	1.74	0.0425	**EEF1AKMT1, APLP1, ODC1, AMACR, MYO1C, IKZF4, SLC2A3, IGF1R**, PHLDA1
**MOREAUX_MULTIPLE_MYELOMA_BY_TACI_UP**	17	-2.11	0.0000	REM1, IKZF4, LBH, CEP170B, KIAA0586, PLEKHA5, HYI, **ARHGAP44, SOX12, TCEAL2, BTN1A1, ARRB1, VASP, NF1, RNASE1, ZNF362, ETV1**

* Core Enrichment genes are shown in bold

Supplementary Table S7. List of 31 differentially expressed lncRNAs in 15 pPCL compared to 50 MM cases, by SAM analysis at q-value=0. Transcripts are ordered according to SAM (d) score.

ensembl gene*	lncRNA	Score(d)	Fold Change
ENSG00000282033	RP11-506F3.1	5,79	1,81
ENSG00000235531	MSC-AS1	5,23	3,11
ENSG00000248773	RP11-231L11.3	4,02	2,65
ENSG00000259287	AC010809.1	-4,87	0,36
ENSG00000229228	LINC00582	-4,74	0,25
ENSG00000236901	MIR600HG	-4,57	0,38
ENSG00000248980	AC019163.1	-4,24	0,62
ENSG00000259291	RP11-617F23.1	-4,14	0,44
ENSG00000268751	SCGB1B2P	-3,82	0,62
ENSG00000243276	RP11-384F7.1	-3,62	0,43
ENSG00000247157	LINC01252	-3,45	0,43
ENSG00000232884	AF127936.3	-3,42	0,25
ENSG00000259408	RP11-3D4.3	-3,39	0,61
ENSG00000244513	CTD-2013N24.2	-3,33	0,63
ENSG00000241525	AC108004.3	-3,24	0,51
ENSG00000236051	MYCBP2-AS1	-3,23	0,52
ENSG00000255183	RP11-720D4.3	-3,23	0,26
ENSG00000253686	LINC01484	-3,20	0,33
ENSG00000223381	RP11-655H13.2	-3,20	0,80
ENSG00000257151	PWAR6	-3,20	0,48
ENSG00000224626	AC106053.1	-3,18	0,52
ENSG00000276759	RP11-80I3.1	-3,18	0,25
ENSG00000263327	TAPT1-AS1	-3,15	0,38
ENSG00000247774	PCED1B-AS1	-3,14	0,28
ENSG00000237513	RP11-325F22.2	-3,14	0,42
ENSG00000225218	AP001628.6	-3,11	0,69
ENSG00000258474	RP11-187E13.1	-3,10	0,80
ENSG00000253315	CTB-11I22.2	-3,00	0,77
ENSG00000255310	AF131215.2	-2,99	0,50
ENSG00000249096	LINC02362	-2,94	0,34
ENSG00000235127	AC068286.1	-2,93	0,72

* Red colored ensembl genes are those in common with the 38-list of differentially expressed lncRNAs between MM and pPCL with t(11;14).

Supplementary Table S8. Ensembl Annotation (GRCh38.p13) of top 33 DE lncRNAs differentially expressed lncRNAs in 7 t(11;14)-pPCL compared to 12 t(11;14)-MM cases, by SAM analysis at q-value=0. Transcripts are ordered according to SAM (d) score.

Gene stable ID	Gene name	Score(d)	Fold Change	Description	Chr.	start (bp)	end (bp)	Strand
ENSG00000197989	**SNHG12**	5,19	2,178365	small nucleolar RNA host gene 12 [Source:HGNC Symbol;Acc:HGNC:30062]	1	28578538	28583132	-1
ENSG00000227912	**AL441943.2**	4,76	1,682956	novel transcript (FLJ31539)	10	2150480	2169460	1
ENSG00000245910	**SNHG6**	4,63	6,527392	small nucleolar RNA host gene 6 [Source:HGNC Symbol;Acc:HGNC:32965]	8	66921684	66926398	-1
ENSG00000253524	**AC124290.2**	-3,63	0,184553	novel transcript	8	36004316	36095046	-1
ENSG00000177406	**NINJ2-AS1**	-3,64	0,545158	NINJ2 antisense RNA 1 [Source:HGNC Symbol;Acc:HGNC:40405]	12	630858	664196	1
ENSG00000258757	**AL133453.1**	-3,68	0,465076	novel transcript, antisense to ERO1L	14	52640839	52641566	1
ENSG00000272189	**AL024508.1**	-3,71	0,695147	novel transcript, antisense to MAP7	6	136550661	136552554	1
ENSG00000234684	**SDCBP2-AS1**	-3,73	0,544211	SDCBP2 antisense RNA 1 [Source:HGNC Symbol;Acc:HGNC:44314]	20	1325405	1378735	1
ENSG00000224220	**DTNB-AS1**	-3,73	0,243588	DTNB antisense RNA 1 [Source:HGNC Symbol;Acc:HGNC:40198]	2	25421117	25427643	1
ENSG00000225655	**BX255923.1**	-3,78	0,556919	novel transcript	9	41073710	41076392	1
ENSG00000270069	**MIR222HG**	-3,79	0,117648	miR222/221 cluster host gene [Source:HGNC Symbol;Acc:HGNC:49555]	X	45745211	45770274	-1
ENSG00000240875	**LINC00886**	-3,79	0,44795	long intergenic non-protein coding RNA 886 [Source:HGNC Symbol;Acc:HGNC:48572]	3	156747343	156817062	-1
ENSG00000236144	**TMEM147-AS1**	-3,85	0,437088	TMEM147 antisense RNA 1 [Source:HGNC Symbol;Acc:HGNC:51273]	19	35540738	35546029	-1
ENSG00000257759	**AL357153.2**	-3,86	0,430814	novel transcript	14	70425812	70547464	-1
ENSG00000229502	**AL391863.1**	-3,86	0,404444	novel transcript	6	157872571	157875210	-1
ENSG00000245534	**RORA-AS1**	-3,93	0,439143	RORA antisense RNA 1 [Source:HGNC Symbol;Acc:HGNC:51410]	15	60479152	60630637	1
ENSG00000247081	**BAALC-AS1**	-3,95	0,514999	BAALC antisense RNA 1 [Source:HGNC Symbol;Acc:HGNC:50461]	8	103153394	103298772	-1
ENSG00000253948	**VPS13B-DT**	-3,99	0,390776	VPS13B divergent transcript [Source:HGNC Symbol;Acc:HGNC:54375]	8	98958277	99013743	-1
ENSG00000269391	**AC010139.1**	-4,09	0,536944	novel transcript	3	16524800	16531807	1
ENSG00000234361	**AL391863.2**	-4,09	0,410476	novel transcript	6	157829143	157830573	-1
ENSG00000232613	**LINC02576**	-4,12	0,10995	long intergenic non-protein coding RNA 2576 [Source:HGNC Symbol;Acc:HGNC:53748]	2	65030727	65053017	1
ENSG00000255949	**RPS6KB2-AS1**	-4,14	0,562092	RPS6KB2 antisense RNA 1 [Source:HGNC Symbol;Acc:HGNC:53744]	11	67431367	67435399	-1
ENSG00000223642	**AC008277.1**	-4,17	0,499357	novel transcript, antisense to BAZ2B	2	159386367	159404636	1
ENSG00000179406	**LINC00174**	-4,18	0,443413	long intergenic non-protein coding RNA 174 [Source:HGNC Symbol;Acc:HGNC:27788]	7	66376044	66493566	-1
ENSG00000247092	**SNHG10**	-4,18	0,480974	small nucleolar RNA host gene 10 [Source:HGNC Symbol;Acc:HGNC:27510]	14	95521943	95534889	-1
ENSG00000253686	**LINC01484**	-4,19	0,095893	long intergenic non-protein coding RNA 1484 [Source:HGNC Symbol;Acc:HGNC:51136]	5	173707614	173746279	-1

Gene stable ID	Gene name	Score(d)	Fold Change	Description	Chr.	start (bp)	end (bp)	Strand
ENSG00000257453	**AC011611.3**	-4,29	0,33742	novel transcript, antisense to PHLDA1	12	76030494	76031378	1
ENSG00000224972	**AL513412.1**	-4,32	0,511749	novel transcript	9	6902670	6978859	1
ENSG00000236723	**AL606760.2**	-4,38	0,550533	novel transcript, antisense to CPT2	1	53209783	53213775	-1
ENSG00000282851	**BISPR**	-4,67	0,476566	BST2 interferon stimulated positive regulator [Source:HGNC Symbol;Acc:HGNC:51290]	19	17405686	17419324	1
ENSG00000251323	**LINC02728**	-4,86	0,402352	long intergenic non-protein coding RNA 2728 [Source:HGNC Symbol;Acc:HGNC:54245]	11	78423982	78429836	-1
ENSG00000232455	**LARS2-AS1**	-4,88	0,518806	LARS2 antisense RNA 1 [Source:HGNC Symbol;Acc:HGNC:40796]	3	45483974	45509545	-1
ENSG00000251364	**AC107884.1**	-4,95	0,318859	novel transcript, antisense to OLFML1	11	7418826	7513644	-1

Supplementary Table S9. Results of Pearson's correlation between the expression levels of the 33 lncRNAs and the overlapping/nearby transcripts globally profiled in 774 MM CoMMpass cohort. In bold, those lncRNA-gene pairs with at least 0.5 significant Pearson's correlation. BH adjustment was applied.

lncRNA Gene ID	lncRNA Gene Name	overlapping/nearby Gene ID	overlapping/ nearby Gene Name	Pearson's Correlation#	biotype*
ENSG00000177406	NINJ2-AS1	ENSG00000171840	NINJ2	r=0.24, q-value =3.6E-11	PC
		ENSG00000256020	AC006205.2	r=0.05, q-value =0.132	lncRNA
		ENSG00000238370	RNU7-103P	r=0.009, q-value =0.8	snRNA
		ENSG00000255825	AC006205.1	r=0.098, q-value =0.009	lncRNA
		ENSG00000256672	LINC02455	r=0.20, q-value =1.52E-8	lncRNA
ENSG00000179406	**LINC00174**	ENSG00000169902	TPST1	r=0.03, q-value =0.492	PC
		ENSG00000252126	RNU6-313P	r=0.01, q-value =0.69	snRNA
		ENSG00000251451	**GTF2IP9**	r=0.82, q-value =4.5E-191	TUP
		ENSG00000231234	**SKP1P1**	r=0.51, q-value =6.8E-53	TPP
		ENSG00000237310	GS1-124K5.4	r=0.35, q-value =4.9E-24	lncRNA
ENSG00000197989	**SNHG12**	ENSG00000180198	RCC1	r=0.19, q-value =1.5E-07	PC
		ENSG00000180098	TRNAU1AP	r=0.3, q-value =4.4E-17	PC
		ENSG00000221539	**SNORD99**	r=0.51, q-value =3.9E-52	snoRNA
		ENSG00000120656	TAF12	r=0.19, q-value =1.5E-07	PC
		ENSG00000188060	RAB42	r=0.13, q-value =0.0002	PC
ENSG00000223642	**AC008277.1**	ENSG00000123636	**BAZ2B**	r=0.53, q-value =1.4E-57	PC
ENSG00000224220	**DTNB-AS1**	ENSG00000235072	ARNILA	r=0.10, q-value =0.0028	lncRNA
		ENSG00000138101	**DTNB**	r=0.89, q-value =4.3E-272	PC
ENSG00000224972	AL513412.1	ENSG00000107077	KDM4C	r=0.30, q-value =3.5E-18	PC
ENSG00000225655	BX255923.1	ENSG00000276291	FRG1HP	not present	TUP
		ENSG00000277778	PGM5P2	not present	TUP
ENSG00000227912	AL441943.2	ENSG00000231326	LINC02662	r=-0.004, q-value =0.9	lncRNA
		ENSG00000212156	RNU6-576P	r=-0.032, q-value =0.57	snRNA
ENSG00000229502	**AL391863.1**	ENSG00000130340	**SNX9**	r=0.65, q-value =3.5E-95	PC
		ENSG00000234361	**AL391863.2**	r=0.65, q-value =7.0E-93	lncRNA
		ENSG00000236324	**SNX9-AS1**	r=0.86, q-value =8.7E-230	lncRNA

lncRNA Gene ID	lncRNA Gene Name	overlapping/nearby Gene ID	overlapping/ nearby Gene Name	Pearson's Correlation#	biotype*
ENSG00000234361	**AL391863.2**	ENSG00000220867	**HSPE1P26**	r=0.80, q-value =4.8E-165	PP
		ENSG00000130340	**SNX9**	r=0.58, q-value =2.4E-71	PC
		ENSG00000229502	**AL391863.1**	r=0.65, q-value =7.0E-93	lncRNA
ENSG00000232613	LINC02576	ENSG00000237638	LINC02245	r=046, q-value =6.0E-42	lncRNA
		ENSG00000115902	SLC1A4	r=0.36, q-value =2.4E-24	PC
		ENSG00000252892	RNU6-548P	r=0.33, q-value =6.0E-21	snRNA
		ENSG00000011523	CEP68	r=0.23, q-value =1.8E-10	PC
		ENSG00000138069	RAB1A	r=0.04, q-value =0.27	PC
ENSG00000232455	LARS2-AS1	ENSG00000011376	LARS2	r=0.27, q-value =1.7E-14	PC
		ENSG00000144791	LIMD1	r=0.37, q-value =1.0E-18	PC
ENSG00000234684	**SDCBP2-AS1**	ENSG00000244588	RAD21L1	r=0.08, q-value =0.021	PC
		ENSG00000101298	SNPH	r=0.27, q-value =1.5E-14	PC
		ENSG00000125775	SDCBP2	r=- 0.19, q-value =0.0011	PC
		ENSG00000274322	AL136531.2	not present	PC
		ENSG00000229728	**AL136531.1**	r=0.72, q-value =1.9E-123	lncRNA
		ENSG00000088832	**FKBP1A**	r=0.56, q-value =1.8E-64	PC
		ENSG00000284436	MIR6869	not present	miRNA
ENSG00000236144	TMEM147-AS1	ENSG00000188508	KRTDAP	r=0.05, q-value =0.19	PC
		ENSG00000161249	DMKN	r=0.08, q-value =0.035	PC
		ENSG00000189001	SBSN	r=0.23, q-value =1.3E-10	PC
		ENSG00000105679	GAPDHS	r=0.25, q-value =1.4E-12	PC
		ENSG00000105677	TMEM147	r=0.30, q-value =5.9E-17	PC
		ENSG00000105675	ATP4A	r=0.15, q-value =4.7E-5	PC
		ENSG00000283907	AD000090.1	not present	lncRNA
		ENSG00000283758	PMIS2	not present	PC
ENSG00000236723	AL606760.2	ENSG00000157184	CPT2	r=- 0.23, q-value =2.1E-07	PC
		ENSG00000162384	CZIB	r=0.007, q-value =0.85	PC
		ENSG00000259818	CZIB-DT	r=- 0.03, q-value =0.50	lncRNA
		ENSG00000162385	MAGOH	r=0.08, q-value =0.06	PC
		ENSG00000226754	RP5-1024G6.5	r=- 0.007, q-value =0.85	lncRNA

lncRNA Gene ID	lncRNA Gene Name	overlapping/nearby Gene ID	overlapping/ nearby Gene Name	Pearson's Correlation#	biotype*
ENSG00000240875	**LINC00886**	ENSG00000157193	LRP8	r=- 0.12, q-value =0.0011	PC
		ENSG00000163659	TIPARP	r=0.11, q-value =0.0018	PC
		ENSG00000174912	**METTL15P1**	r=0.59, q-value =4.5E-75	PP
		ENSG00000230457	PA2G4P4	r=0.32, q-value =1.1E-15	PP
		ENSG00000197980	LEKR1	r=0.40, q-value =3.2E-17	PC
ENSG00000245534	**RORA-AS1**	ENSG00000128915	**ICE2**	r=0.51, q-value =1.5E-38	PC
		ENSG00000069667	RORA	r=0.02, q-value =0.52	PC
		ENSG00000259274	**AC107241.1**	r=0.59, q-value =1.0E-73	lncRNA
		ENSG00000259513	CYCSP38	r=0.41, q-value =4.4E-29	TPP
		ENSG00000273958	AC009560.1	not present	lncRNA
		ENSG00000286457	AC009560.3	not present	lncRNA
		ENSG00000287508	AC009560.4	not present	lncRNA
ENSG00000245910	SNHG6	ENSG00000178460	MCMDC2	r=0.19, q-value =1.4E-07	PC
		ENSG00000261787	TCF24	r=0.017, q-value =0.63	PC
		ENSG00000178125	PPP1R42	r=- 0.02, q-value =0.63	PC
ENSG00000247081	**BAALC-AS1**	ENSG00000250929	**LINC01181**	r=0.55, q-value =4.9E-61	lncRNA
		ENSG00000236939	BAALC-AS2	r=0.22, q-value =8.9E-10	lncRNA
		ENSG00000164929	BAALC	r=- 0.001, q-value =0.98	PC
		ENSG00000265657	MIR3151	r=0.38, q-value =1.2E-28	miRNA
		ENSG00000253851	AC025370.1	r=0.07, q-value =0.065	lncRNA
		ENSG00000164930	**FZD6**	r=0.84, q-value =1.5E-209	PC
		ENSG00000286337	AC025370.2	not present	lncRNA
ENSG00000247092	**SNHG10**	ENSG00000176438	SYNE3	r=0.02, q-value =0.49	PC
		ENSG00000258572	AL133467.1	r=0.17, q-value =1.7E-06	lncRNA
		ENSG00000252481	**SCARNA13**	r=0.59, q-value =4.8E-74	scaRNA
		ENSG00000182512	GLRX5	r=0.41, q-value =1.4E-32	PC
		ENSG00000258390	LINC02318	r=0.26, q-value =1.4E-10	lncRNA
ENSG00000251323	LINC02728	ENSG00000033327	GAB2	r=0.37, q-value =5.3E-12	PC
		ENSG00000254420	AP003086.1	r=0.32, q-value =1.0E-15	lncRNA
		ENSG00000254649	AP003086.2	r=0.21, q-value =2.9E-09	lncRNA

lncRNA Gene ID	lncRNA Gene Name	overlapping/nearby Gene ID	overlapping/ nearby Gene Name	Pearson's Correlation#	biotype*
ENSG00000251364	**AC107884.1**	ENSG00000137513	NARS2	r=0.42, q-value =1.6E-30	PC
		ENSG00000170743	SYT9	r=0.39, q-value =1.2E-29	PC
		ENSG00000183801	**OLFML1**	r=0.61, q-value =2.2E-79	PC
		ENSG00000166387	PPFIBP2	r=0.49, q-value =3.7E-34	PC
ENSG00000253524	AC124290.2	ENSG00000253452	AC124290.1	r=0.21, q-value =6.3E-09	lncRNA
ENSG00000253686	**LINC01484**	ENSG00000253959	**LINC01863**	r=0.54, q-value =1.0E-44	lncRNA
		ENSG00000253428	**LINC01942**	r=0.75, q-value =4.4E-139	lncRNA
		ENSG00000287003	AC008674.1	not present	lncRNA
		ENSG00000254211	LINC01485	r=0.48, q-value =3.4E-45	lncRNA
ENSG00000253948	VPS13B-DT	ENSG00000104375	STK3	r=0.22, q-value =1.2E-09	PC
		ENSG00000271930	AC016877.3	r=0.05, q-value =0,044	lncRNA
		ENSG00000164920	OSR2	r=- 0.02, q-value =0.54	PC
		ENSG00000229625	AC016877.1	r=0.12, q-value =0,00075	lncRNA
		ENSG00000132549	VPS13B	r=0.28, q-value =3.3E-15	PC
ENSG00000255949	RPS6KB2-AS1	ENSG00000172613	RAD9A	r=0.03, q-value =0.63	PC
		ENSG00000253024	RNU6-1238P	r=- 0.08, q-value =0.18	snRNA
		ENSG00000172531	PPP1CA	r=0.06, q-value =0.32	PC
		ENSG00000175463	TBC1D10C	r=0.04, q-value =0.40	PC
		ENSG00000172508	CARNS1	r=0.02, q-value =0.69	PC
		ENSG00000175634	RPS6KB2	r=0.06, q-value =0.32	PC
		ENSG00000213402	PTPRCAP	r=0.02, q-value =0.63	PC
		ENSG00000172725	CORO1B	r=0.04, q-value =0.40	PC
		ENSG00000175514	GPR152	r=0.01, q-value =0.74	PC
		ENSG00000175544	CABP4	r=0.05, q-value =0.34	PC
		ENSG00000172663	TMEM134	r=0.01, q-value =0.71	PC
		ENSG00000110711	AIP	r=0.02, q-value =0.65	PC
ENSG00000257453	**AC011611.3**	ENSG00000258077	**AC078923.1**	r=0.54, q-value =9.6E-46	lncRNA
		ENSG00000139289	**PHLDA1**	r=0.87, q-value =1.2E-233	PC
		ENSG00000257839	RP11-290L1.2	r=0.38, q-value =1.4E-25	lncRNA
		ENSG00000187109	NAP1L1	r=0.08, q-value =0.014	PC

lncRNA Gene ID	lncRNA Gene Name	overlapping/nearby Gene ID	overlapping/ nearby Gene Name	Pearson's Correlation#	biotype*
ENSG00000257759	AL357153.2	ENSG00000133983	COX16	r=0.21, q-value =5.0E-09	PC
		ENSG00000258644	SYNJ2BP-COX16	r=- 0.007, q-value =0.84	PC
		ENSG00000213463	SYNJ2BP	r=0.48, q-value =9.0E-45	PC
		ENSG00000139985	ADAM21	r=0.28, q-value =4.0E-15	PC
		ENSG00000259158	ADAM20P1	r=0.34, q-value =3.3E-08	TUP
		ENSG00000252263	RNU6-659P	r=0.01, q-value =0.77	snRNA
		ENSG00000134007	ADAM20	r=0.15, q-value =3.3E-09	PC
		ENSG00000133997	MED6	r=0.22, q-value =4.2E-10	PC
		ENSG00000259115	AL357153.3	r=0.02, q-value =0.66	lncRNA
ENSG00000258757	AL133453.1	ENSG00000180998	GPR137C	r=- 0.02, q-value =0.54	PC
		ENSG00000197930	ERO1A	r=- 0.10, q-value =0.0064	PC
ENSG00000269391	AC010139.1	ENSG00000233570	LINC00690	r=0.09, q-value =0.013	lncRNA
ENSG00000270069	**MIR222HG**	ENSG00000204915	MFFP3	r=0.15, q-value =4.8E-05	PP
		ENSG00000207870	**MIR221**	r=0.95, q-value =0.0E-00	miRNA
		ENSG00000207725	MIR222	r=0.35, q-value =6.6E-21	miRNA
		ENSG00000269902	**AC234772.1**	r=0.90, q-value =5.0E-295	lncRNA
ENSG00000272189	**AL024508.1**	ENSG00000135525	**MAP7**	r=0.55, q-value =1.6E-63	PC
		ENSG00000271765	RN7SKP299	r=0.31, q-value =2.4E-18	misc_RNA
		ENSG00000197442	MAP3K5	r=0.19, q-value =1.3E-07	PC
ENSG00000282851	BISPR[#]	ENSG00000130300	PLVAP	-	PC
		ENSG00000269350	AC010463.2	-	lncRNA
		ENSG00000269720	CCDC194	-	PC
		ENSG00000130303	BST2	-	PC
		ENSG00000141971	MVB12A	-	PC
		ENSG00000269481	AC010319.4	-	lncRNA
		ENSG00000269053	AC010319.3	-	lncRNA
		ENSG00000188051	TMEM221	-	PC
		ENSG00000269035	AC010319.2	-	PC
		ENSG00000269736	AC010319.5	-	lncRNA
		ENSG00000171773	NXNL1	-	PC

lncRNA Gene ID	lncRNA Gene Name	overlapping/nearby Gene ID	overlapping/ nearby Gene Name	Pearson's Correlation#	biotype*
		ENSG00000130304	SLC27A1	-	PC

# - No correlation as BIPR is absent in coMMpass matrix; * PC= Protein coding; TUP=transcribed unprocessed pseudogene; PP= processed pseudogene; TPP=transcribed processed pseudogene

Supplementary Table S10. Results of Cox regression multivariate analysis for selected lncRNAs in high versus low expression group, together with significant clinical and molecular variables respectively in PFS and OS, in 497 MM patients of CoMMpass cohort. The number (N) and percentage of MM cases at higher expression levels are reported for each lncRNA. Hazard Ratio (HR) and 95% Confidence Interval (95% CI) are reported for each variable. Global p-value from Log-Rank test is also indicated for each analysis.

Multivariate Cox Analysis				
	OS		***PFS***	
Variable	***P-value***	***HR (95% CI)***	***P-value***	***HR (95% CI)***
**ENSG00000245910_high.level (N=262, 52,7%)**	**1,40E-02**	**1,62 (1,10-2,39)**		
**del1p.CDKN2C**	1,98E-01	1.32 (0,87-2,00)		
**del13q.RB1**	7,30E-02	1.52 (0,96-2,39)		
**HD**	2,15E-01	0,77 (0,51-1,16)		
**TP53.alt.1q.gain.amp**	<0,001	3,58 (1,74-7,40)		
**gain.amp.1q**	1,80E-02	1.63 (1,09-2,44)		
**DIS3mut**	2,94E-01	1.31 (0,79-2,16)		
**t4.14**	6,09E-01	0,87 (0,51-1,49)		
**trx.MAF**	9,50E-01	1.02 (0,53-1,98)		
**ISS I**	<0,001	0,40 (0,23-0,69)		
**ISS III**	4,90E-02	1.51 (1,00-2,27)		
Log-Rank global p-value	p=9,1e-11			
**ENSG00000240875_high.level (N=240, 48%)**	3,02E-01	0,82 (0,56-1,20)	3,64E-01	0,88 (0,68-1,15)
**del1p.CDKN2C**	1,90E-01	1.32 (0,87-2,00)	-	-
**del13q.RB1**	6,10E-02	1.54 (0,98-2,43)	2,90E-02	1.40 (1,03-1,91)
**HD**	1,69E-01	0,75 (0,50-1,13)	3,70E-02	0,74 (0,55-0,98)
**TP53.alt.1q.gain.amp**	1,00E-03	3,29 (1,60-6,77)	1,60E-02	2,13 (1,15-3,94)
**gain.amp.1q**	3,70E-02	1.53 (1,02-2,28)	8,60E-02	1,28 (0,97-1,69)
**DIS3mut**	3,08E-01	1.29 (0,79-2,12)	2,71E-01	1,22 (0.86-1.73)
**t4.14**	2,60E-01	0,74 (0,43-1,25)	8,42E-01	0,96 (0,65-1,42)
**trx.MAF**	9,80E-01	1.01 (0,52-1,95)	9,26E-01	1.02 (0,62-1,69)
**trx.MYC**	-	-	2,80E-02	1.89 (1,07-3,34)
**ISS I**	<0,001	0,40 (0,23-0,69)	2,00E-03	0,58 (0,42-0,82)
**ISS III**	4,90E-02	1.51 (1,00-2,20)	3,10E-02	1.39 (1,03-1,87)
Log-Rank global p-value	p=8,3e-10		p=8,1e-11	
**ENSG00000251323_high.level (N=263, 53%)**			4,36E-01	0,90 (0,68-1,18)
**del13q.RB1**			2,30E-02	1.42 (1.05-1.92)
**HD**			4,10E-02	0,74 (0.56-0.99)
**TP53.alt.1q.gain.amp**			2,10E-02	2,08 (1.12-3.86)
**gain.amp.1q**			1,17E-01	1,25 (0.96-1.66)
**DIS3mut**			2,54E-01	1,23 (0.86-1.75)
**t4.14**			8,05E-01	0,95 (0.64-1.41)
**trx.MAF**			9,78E-01	1,01 (0.61-1.66)
**trx.MYC**			2,10E-02	1,97 (1.11-3,50)
**ISS I**			1,00E-03	0,57 (0.41-0.80)
**ISS III**			3,60E-02	1,38 (1.02-1.86)
Log-Rank global p-value			p=8,96e-11	

Variable	OS		PFS	
	P-value	*HR (95% CI)*	*P-value*	*HR (95% CI)*
**ENSG00000177406_high.level (N=246, 49%)**			8,40E-02	0,79 (0,60-1,03)
**del13q.RB1**			2,10E-02	1.43 (1,06-1,93)
**HD**			1,50E-02	0,69 (0,51-0,93)
**TP53.alt.1q.gain.amp**			2,00E-02	2,08 (1,12-3,85)
**gain.amp.1q**			1,46E-01	1,23 (0,93-1,63)
**DIS3mut**			2,44E-01	1.23 (0,87-1,74)
**t4.14**			8,66E-01	0,97 (0,66-1,43)
**trx.MAF**			9,84E-01	0,99 (0,60-1,64)
**trx.MYC**			5,00E-02	1.77 (1,00-3,15)
**ISS I**			2,00E-03	0,59 (0,42-0,82)
**ISS III**			3,10E-02	1.39 (1,03-1,88)
Log-Rank global p-value			p=3,1e-11	

Supplementary Table S11. Fisher's exact test measuring the correlation between low and high SNHG6 expression level and the occurrence of main IgH translocations (trx), copy number alterations (CNAs) and non-synonymous somatic mutations in recurrently mutated genes in coMMpass MM cases. del(13q) refers to 13q14, 13q34 cytobands or *RB1* locus; 1q gain to 1q21 cytoband; del(1p) to 1p22 or *CDKN2C* locus; del(17p) to 17p13 or *TP53* locus; HD to hyperdiploid condition. The total number of MM samples analyzed for each kind of data is reported. Significant adjusted p-values (<0.05) were in bold.

	**IgH trx (497 MM)**	***SNHG6* low N**	***SNHG6 high* N**	**P-value**
**t(4;14)**	neg	194	234	**0,03**
	pos	41	28	
**MAF.trx**	neg	223	241	0,21
	pos	12	21	
**t(11;14)**	neg	193	202	0,18
	pos	42	60	
**MYC.trx**	neg	229	248	0,17
	pos	6	14	
	**CNA (497 MM)**	***SNHG6* low N**	***SNHG6 high* N**	**P-value**
**1q21 gain**	neg	143	190	**0,007**
	pos	92	72	
**del(13q)**	neg	117	122	0,53
	pos	118	140	
**1p loss**	neg	169	185	0,62
	pos	66	77	
**del(17p)/TP53**	neg	214	227	0,15
	pos	21	35	
**HD**	neg	99	117	0,58
	pos	136	145	
**TP53alt/1q21 gain**	neg	226	252	1
	pos	9	10	
	**Nonsyn. Somatic Mutation (497 MM)**	***SNHG6* low N**	***SNHG6 high* N**	**P-value**
N-RAS	WT	187	193	0,41
	mut	48	69	
K-RAS	WT	181	195	0,8200
	mut	54	67	
DIS3	WT	211	236	1
	mut	24	26	
BRAF	WT	218	243	1
	mut	17	19	
TP53alt	WT	222	235	0,36
	mut	13	27	
FAM46C	WT	213	235	0,9400
	mut	22	27	

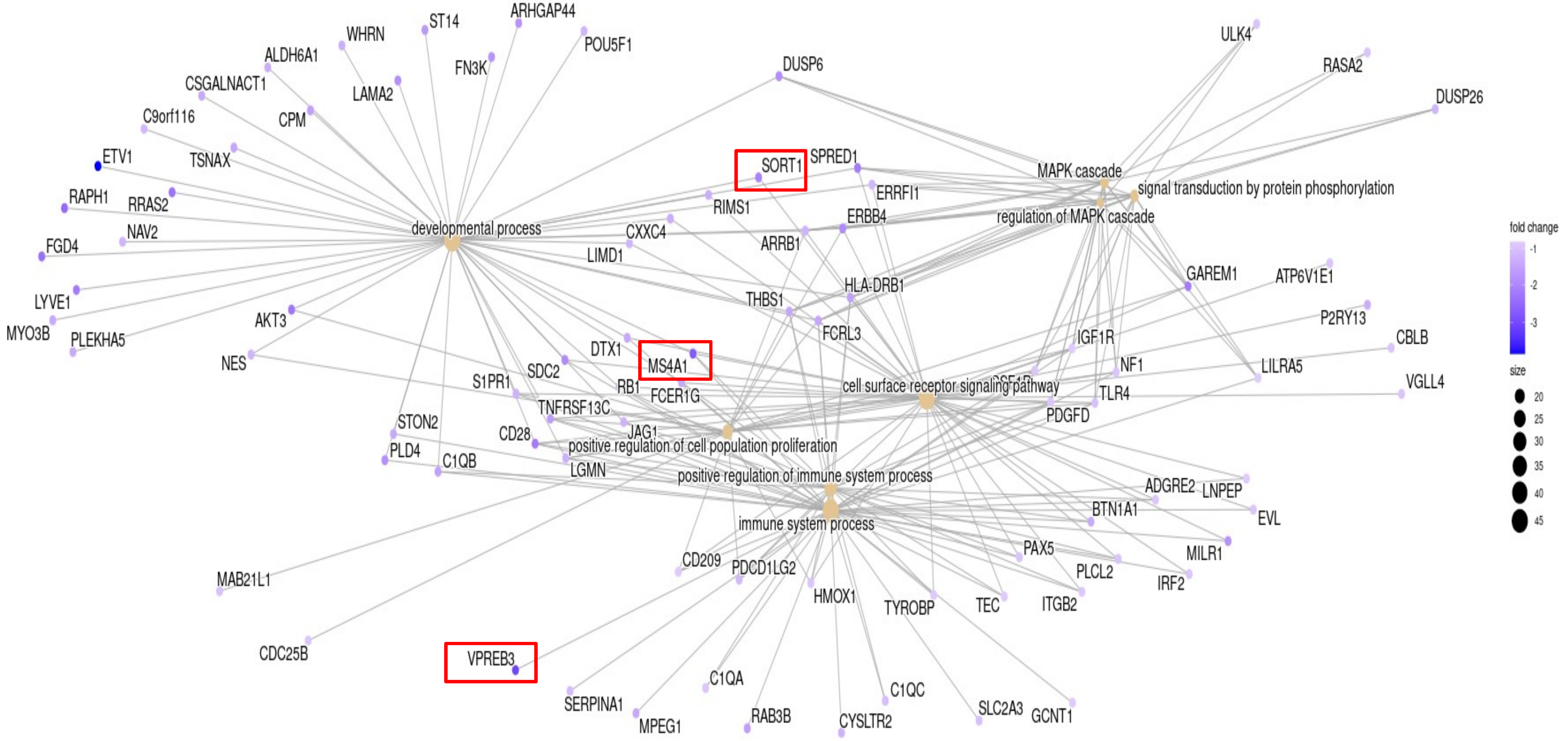

Supplementary Figure S1: Enrichment map on top 8 GSEA gene sets based on GO-BP terms, performed by Cluster Profiler analysis on DE global protein coding gene list. B cell related genes upregulated in venetoclax sensitive MM are marked by a red box.

BCL-2

	N	MM t(11;14)	MM	pPCL t(11;14)
MM t(11;14)	**0.0043**			
MM	**0.035**	**0.043**		
pPCL t(11;14)	0.053	0.14	0.44	
pPCL	0.064	0.09	0.47	0.43

BCL2L1

	N	MM t(11;14)	MM	pPCL t(11;14)
MM t(11;14)	**0.0063**			
MM	0.10	**0.010**		
pPCL t(11;14)	**0.0001**	**0.039**	**0.0000**	
pPCL	**0.045**	0.18	0.17	**0.0083**

BCL2L2

	N	MM t(11;14)	MM	pPCL t(11;14)
MM t(11;14)	0.39			
MM	0.12	0.08		
pPCL t(11;14)	**0.014**	**0.0056**	**0.032**	
pPCL	0.19	0.20	0.43	0.054

MCL1

	N	MM t(11;14)	MM	pPCL t(11;14)
MM t(11;14)	**0.019**			
MM	**0.0027**	0.20		
pPCL t(11;14)	0.14	0.13	**0.026**	
pPCL	**0.0023**	0.11	0.24	**0.019**

BCL2A1

	N	MM t(11;14)	MM	pPCL t(11;14)
MM t(11;14)	0.14			
MM	0.13	0.44		
pPCL t(11;14)	0.27	**0.018**	**0.010**	
pPCL	0.18	0.43	0.47	**0.03**

HRK

	N	MM t(11;14)	MM	pPCL t(11;14)
MM t(11;14)	0.07			
MM	**0.008**	0.1		
pPCL t(11;14)	**0.034**	0.27	0.37	
pPCL	0.11	0.39	0.08	0.21

BAD

	N	MM t(11;14)	MM	pPCL t(11;14)
MM t(11;14)	0.43			
MM	0.172	0.11		
pPCL t(11;14)	0.23	0.23	0.45	
pPCL	0.33	0.36	0.27	0.35

BMF

	N	MM t(11;14)	MM	pPCL t(11;14)
MM t(11;14)	0.30			
MM	**0.04**	**0.03**		
pPCL t(11;14)	0.47	0.29	**0.020**	
pPCL	0.32	0.48	0.06	0.32

PMAIP1

	N	MM t(11;14)	MM	pPCL t(11;14)
MM t(11;14)	0.057			
MM	0.42	**0.0069**		
pPCL t(11;14)	**0.0078**	0.10	**0.0003**	
pPCL	0.25	0.13	0.20	**0.01**

BBC3

	N	MM t(11;14)	MM	pPCL t(11;14)
MM t(11;14)	0.26			
MM	0.33	0.33		
pPCL t(11;14)	0.43	0.29	0.39	
pPCL	0.36	0.36	0.49	0.41

BCL2L11

	N	MM t(11;14)	MM	pPCL t(11;14)
MM t(11;14)	**0.0099**			
MM	0.12	**0.011**		
pPCL t(11;14)	0.07	0.17	0.23	
pPCL	0.12	0.07	0.39	0.35

BID

	N	MM t(11;14)	MM	pPCL t(11;14)
MM t(11;14)	**0.0016**			
MM	**0.0002**	0.33		
pPCL t(11;14)	0.06	0.06	**0.0178**	
pPCL	**0.0102**	0.27	0.14	0.19

BAK1

	N	MM t(11;14)	MM	pPCL t(11;14)
MM t(11;14)	0.20			
MM	**0.0050**	**0.0040**		
pPCL t(11;14)	0.23	0.48	**0.0145**	
pPCL	**0.0129**	**0.026**	0.49	**0.039**

BAX

	N	MM t(11;14)	MM	pPCL t(11;14)
MM t(11;14)	0.39			
MM	0.092	**0.0051**		
pPCL t(11;14)	013	**0.0355**	0.49	
pPCL	0.06	**0.0084**	0.26	0.32

BCL-2/BCL2L1

	N	MM t(11;14)	MM	pPCL t(11;14)
MM t(11;14)	**0.0005**			
MM	0.053	**0.0007**		
pPCL t(11;14)	**0.0001**	0.13	**0.0001**	
pPCL	**0.022**	0.07	0.15	**0.011**

BCL-2/MCL1

	N	MM t(11;14)	MM	pPCL t(11;14)
MM t(11;14)	0.227			
MM	0.386	**0.039**		
pPCL t(11;14)	0.250	0.491	0.08	
pPCL	0.254	**0.033**	0.257	0.055

Supplementary Figure S2: Results of Dunn's test evaluating the differences in the expression of BCL2 gene family in MM and pPCL stratified by the presence of t(11;14), and in normal controls (N). Significant p-value<0.05 is marked red-bold. Expression levels are plotted in Figure 3B-C .

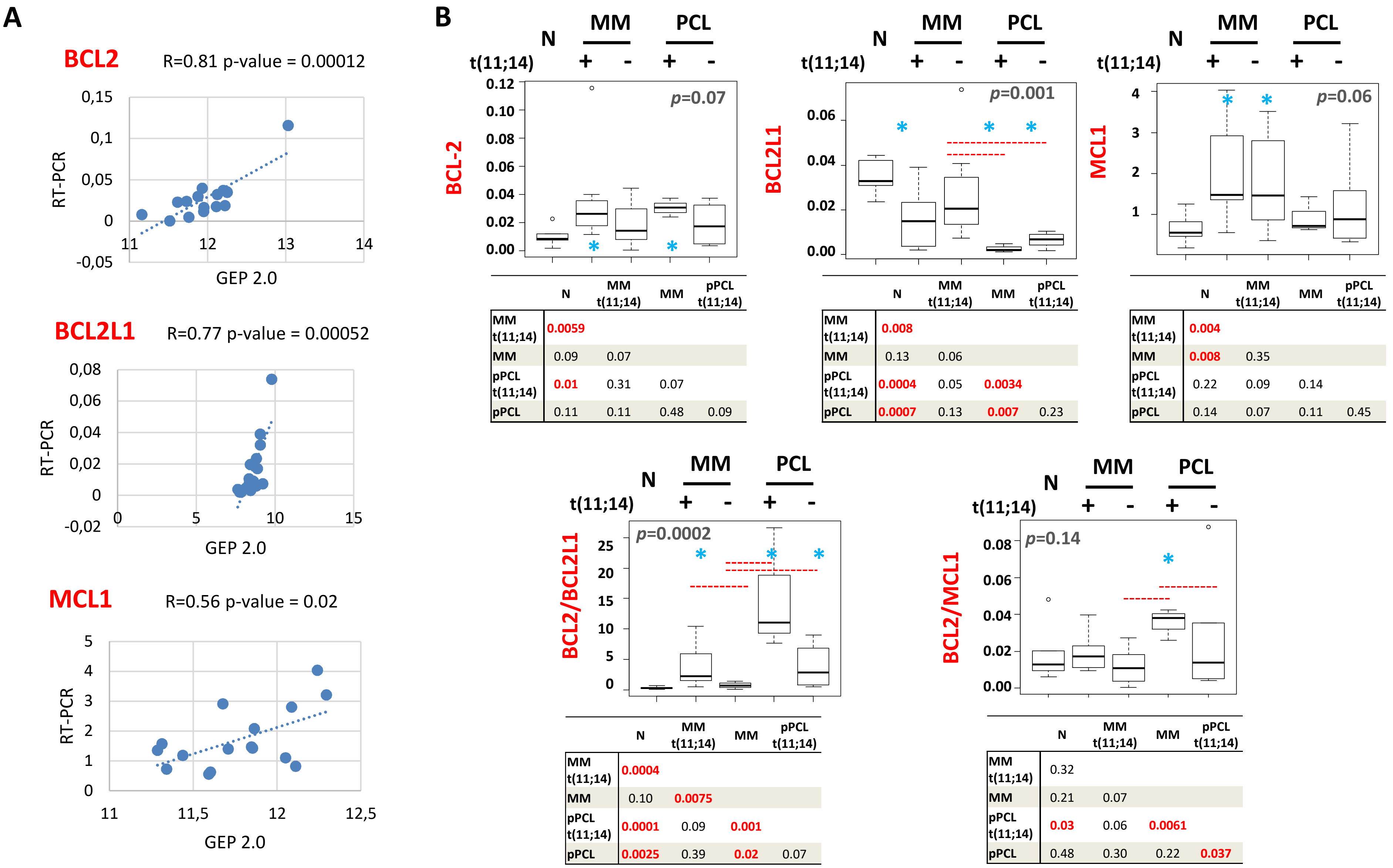

BCL-2

	N	MM t(11;14)	MM	pPCL t(11;14)
MM t(11;14)	**0.0059**			
MM	0.09	0.07		
pPCL t(11;14)	**0.01**	0.31	0.07	
pPCL	0.11	0.11	0.48	0.09

BCL2L1

	N	MM t(11;14)	MM	pPCL t(11;14)
MM t(11;14)	**0.008**			
MM	0.13	0.06		
pPCL t(11;14)	**0.0004**	0.05	**0.0034**	
pPCL	**0.0007**	0.13	**0.007**	0.23

MCL1

	N	MM t(11;14)	MM	pPCL t(11;14)
MM t(11;14)	**0.004**			
MM	**0.008**	0.35		
pPCL t(11;14)	0.22	0.09	0.14	
pPCL	0.14	0.07	0.11	0.45

BCL2/BCL2L1

	N	MM t(11;14)	MM	pPCL t(11;14)
MM t(11;14)	**0.0004**			
MM	0.10	**0.0075**		
pPCL t(11;14)	**0.0001**	0.09	**0.001**	
pPCL	**0.0025**	0.39	**0.02**	0.07

BCL2/MCL1

	N	MM t(11;14)	MM	pPCL t(11;14)
MM t(11;14)	0.32			
MM	0.21	0.07		
pPCL t(11;14)	**0.03**	0.06	**0.0061**	
pPCL	0.48	0.30	0.22	**0.037**

SupplementaryFigure S3: Quantitative RT-PCR validation of BCL2, BCL2L1, and MCL1 expression levels. **(A)**Pearson's correlation coefficient was calculated between GEP data and quantitative RT-PCR results expressed as $2^{-\Delta Ct}$ in 16 samples. **(B)**Box plot representation of the gene expression levels in 34 samples including 6 normal controls (N), 9 MM patients carrying t(11;14), 10 MMs without t(11;14), 3 pPCL patients carrying t(11;14),and 6pPCLs without t(11;14) evaluated by kruskal-Wallis test (p-values are shown for each panel). The expression levels are represented as $2^{-\Delta Ct}$. In each panel, red dashed lines above two groups indicate significant differences in their gene expression level, blue asterisks indicate significant differences between the indicated group and N evaluated by Dunn's test (table under the corresponding box plot, significant p-value<0.05 is marked red-bold).

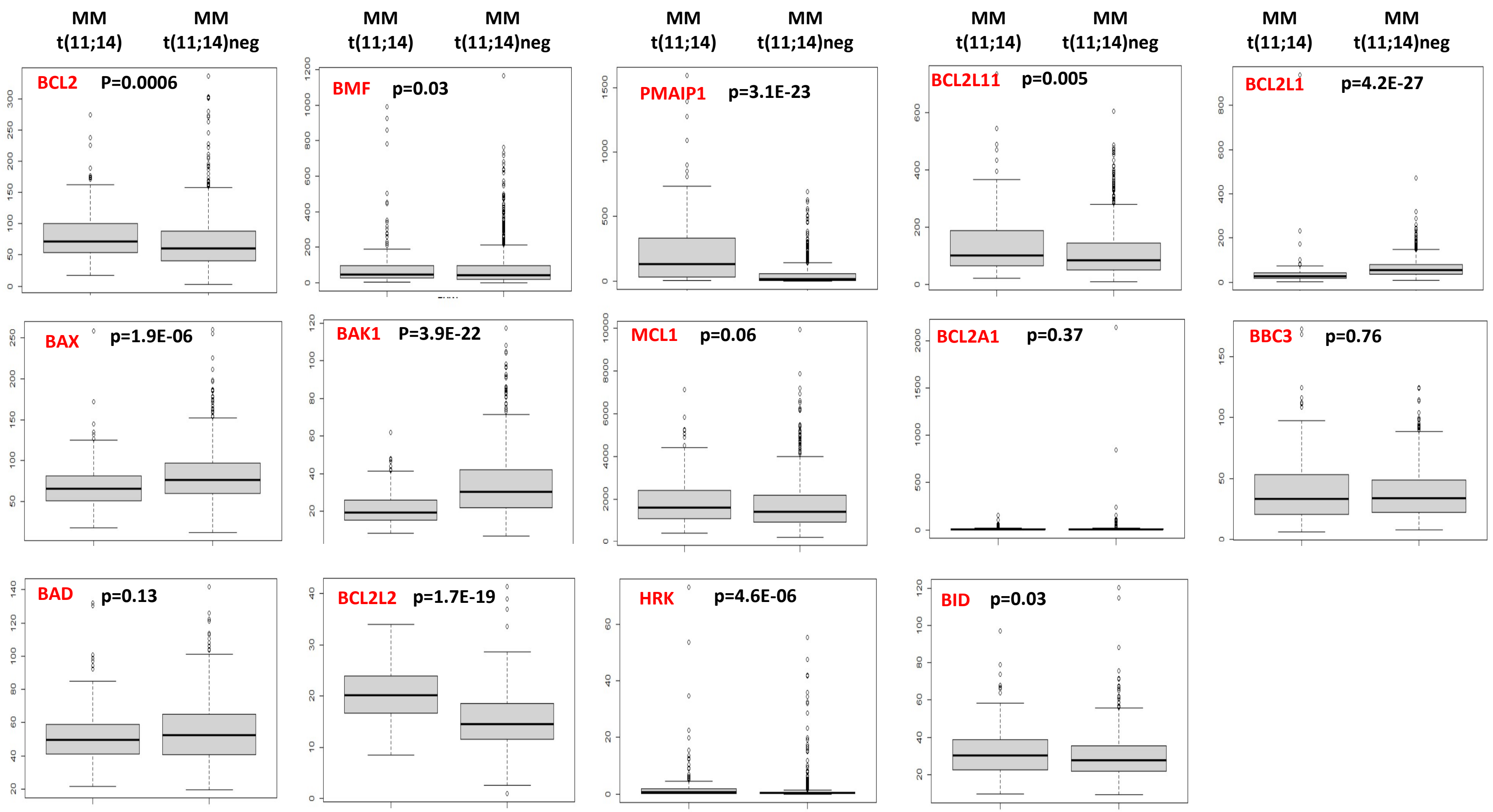

Supplementary Figure S4: Box plot representation of the mRNA expression of BCL2 family genes in 156 MM with t(11;14) and 618 MM without t(11;14) from the CoMMpass database. For each plot BH adjusted p-value is reported.

MS4A1

	N	MM t(11;14)	MM	pPCL t(11;14)
MM t(11;14)	0.49			
MM	**0.011**	**0.0002**		
pPCL t(11;14)	**0.032**	**0.0075**	0.47	
pPCL	**0.040**	**0.0100**	0.37	0.42

VPREB3

	N	MM t(11;14)	MM	pPCL t(11;14)
MM t(11;14)	0.28			
MM	**0.0020**	**0.0000**		
pPCL t(11;14)	**0.0058**	**0.0016**	0.43	
pPCL	**0.0009**	**0.0001**	0.15	0.26

RASGRP2

	N	MM t(11;14)	MM	pPCL t(11;14)
MM t(11;14)	**0.013**			
MM	**0.0006**	0.10		
pPCL t(11;14)	**0.0006**	0.056	0.20	
pPCL	**0.014**	0.44	0.17	0.09

PIK3AP1

	N	MM t(11;14)	MM	pPCL t(11;14)
MM t(11;14)	**0.044**			
MM	**0.0024**	0.06		
pPCL t(11;14)	**0.0004**	**0.0084**	0.059	
pPCL	**0.0003**	**0.0073**	0.055	0.48

CD79A

	N	MM t(11;14)	MM	pPCL t(11;14)
MM t(11;14)	0.5			
MM	**0.0016**	**0.0000**		
pPCL t(11;14)	**0.0040**	**0.0002**	0.38	
pPCL	**0.0004**	**0.0000**	0.10	0.23

STAT5A

	N	MM t(11;14)	MM	pPCL t(11;14)
MM t(11;14)	0.12			
MM	**0.0025**	**0.0070**		
pPCL t(11;14)	**0.0018**	**0.0070**	0.19	
pPCL	**0.0005**	**0.0015**	0.08	0.36

SORT1

	N	MM t(11;14)	MM	pPCL t(11;14)
MM t(11;14)	**0.0014**			
MM	0.44	**0.0000**		
pPCL t(11;14)	0.19	**0.0064**	0.13	
pPCL	0.20	**0.0000**	0.06	**0.021**

BEND5

	N	MM t(11;14)	MM	pPCL t(11;14)
MM t(11;14)	0.44			
MM	**0.0018**	**0.0000**		
pPCL t(11;14)	**0.0013**	**0.0001**	0.18	
pPCL	**0.0015**	**0.0001**	0.23	0.43

IL4R

	N	MM t(11;14)	MM	pPCL t(11;14)
MM t(11;14)	**0.016**			
MM	**0.020**	0.31		
pPCL t(11;14)	**0.0052**	0.21	0.09	
pPCL	**0.049**	0.31	0.43	0.12

REL

	N	MM t(11;14)	MM	pPCL t(11;14)
MM t(11;14)	0.35			
MM	**0.0089**	**0.0009**		
pPCL t(11;14)	**0.033**	**0.024**	0.40	
pPCL	**0.031**	**0.021**	0.39	0.49

CXCR5

	N	MM t(11;14)	MM	pPCL t(11;14)
MM t(11;14)	**0.023**			
MM	**0.0094**	0.39		
pPCL t(11;14)	0.134	0.16	0.09	
pPCL	**0.029**	0.49	0.41	0.18

BATF

	N	MM t(11;14)	MM	pPCL t(11;14)
MM t(11;14)	**0.0023**			
MM	**0.023**	**0.037**		
pPCL t(11;14)	0.32	**0.0024**	**0.033**	
pPCL	**0.028**	0.15	0.37	**0.04**

Supplementary Figure S5: Results of Dunn's test evaluating the differences in the expression of B-cell genes from the signature of venetoclax sensitive myeloma in MM and PCL stratified by the presence of t(11;14), and in normal controls (N). Significant p-value<0.05 is marked red-bold. Expression levels are plotted in Figure 4.

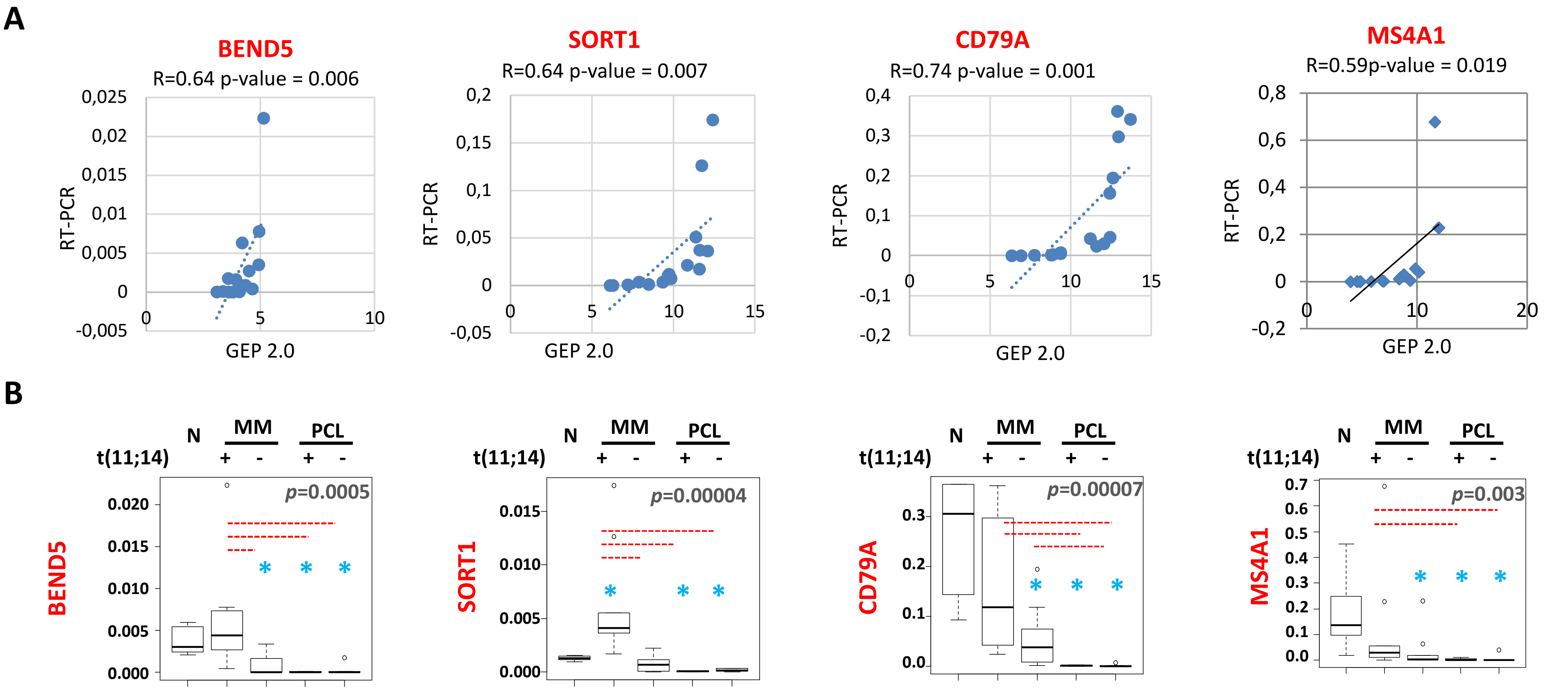

BEND5

	N	MM t(11;14)	MM	pPCL t(11;14)
MM t(11;14)	0.34			
MM	**0.0065**	**0.0006**		
pPCL t(11;14)	**0.013**	**0.0037**	0.32	
pPCL	**0.0054**	**0.0007**	0.35	0.44

SORT1

	N	MM t(11;14)	MM	pPCL t(11;14)
MM t(11;14)	**0.041**			
MM	0.08	**0.0002**		
pPCL t(11;14)	**0.014**	**0.0001**	0.10	
pPCL	**0.017**	**0.0000**	0.16	0.32

CD79A

	N	MM t(11;14)	MM	pPCL t(11;14)
MM t(11;14)	0.13			
MM	**0.009**	0.08		
pPCL t(11;14)	**0.0006**	**0.0052**	0.05	
pPCL	**0.0000**	**0.0003**	**0.011**	0.44

MS4A1

	N	MM t(11;14)	MM	pPCL t(11;14)
MM t(11;14)	0.13			
MM	**0.0056**	0.05		
pPCL t(11;14)	**0.002**	**0.019**	0.16	
pPCL	**0.0007**	**0.008**	0.15	0.43

SupplementaryFigure S6: Quantitative RT-PCR validation of BEND5, SORT1, CD79A, and MS4A1 expression levels **(A)**Pearson's correlation coefficient was calculated between GEP data and quantitative RT-PCR results expressed as $2^{-\Delta Ct}$ in 16 samples. **(B)** Box plot representation of the gene expression levels in 34 samples including 6 normal controls (N), 9 MM patients carrying t(11;14), 10 MMs without t(11;14), 3 pPCL patients carrying t(11;14), and 6 pPCLs without t(11;14) evaluated by kruskal-Wallis test (p-values are shown for each panel). The expression levels are represented as $2^{-\Delta Ct}$. In each panel, red dashed lines above two groups indicate significant differences in their gene expression level, blue asterisks indicate significant differences between the indicated group and N evaluated by Dunn's test (table under the corresponding box plot, significant p-value<0.05 is marked red-bold).

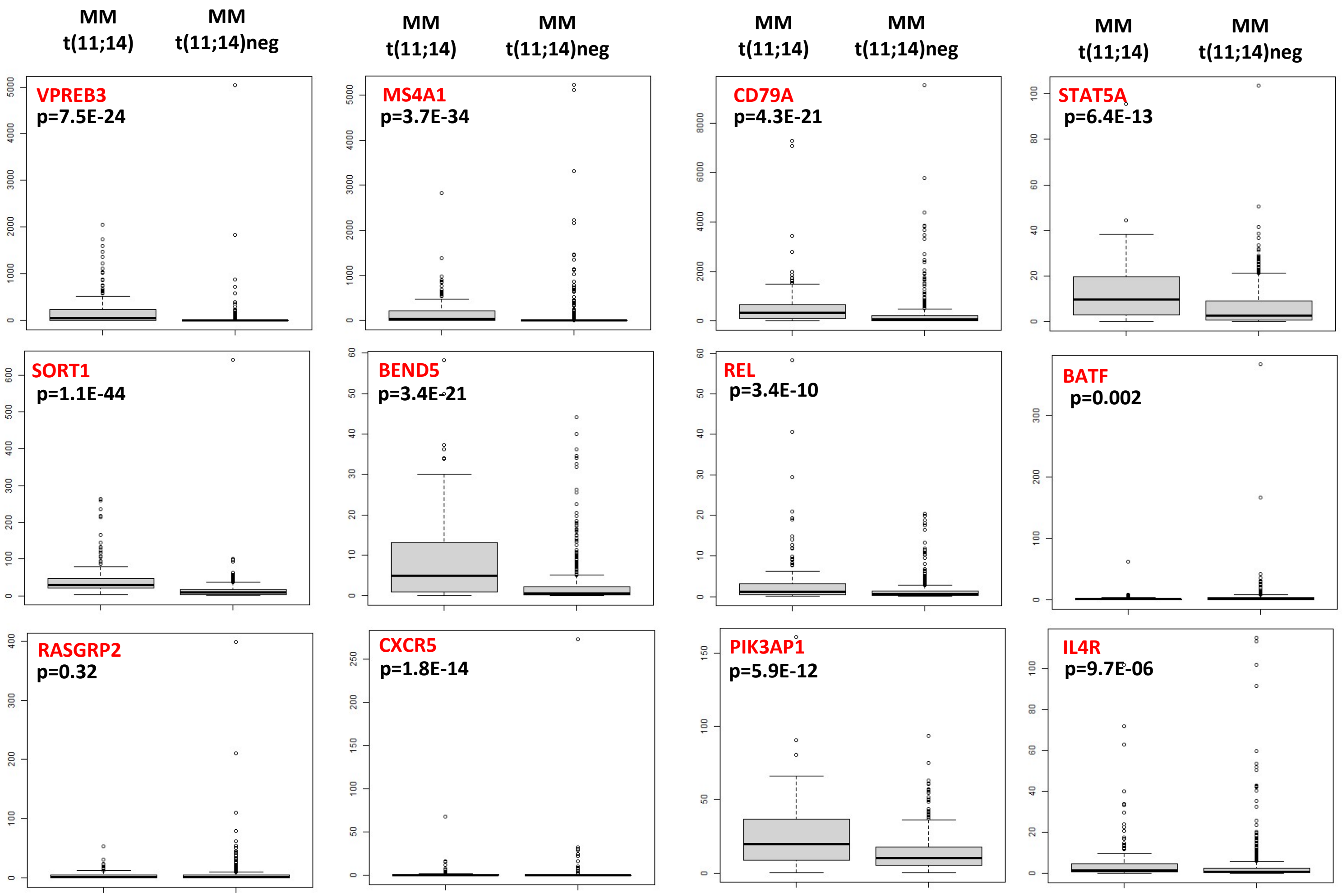

Supplementary Figure S7: Box plot representation of the mRNA expression of the signature of venetoclax sensitive myeloma in 156 MM with t(11;14) and 618 MM without t(11;14) from the CoMMpass database. For each plot BH adjusted p-value is reported.

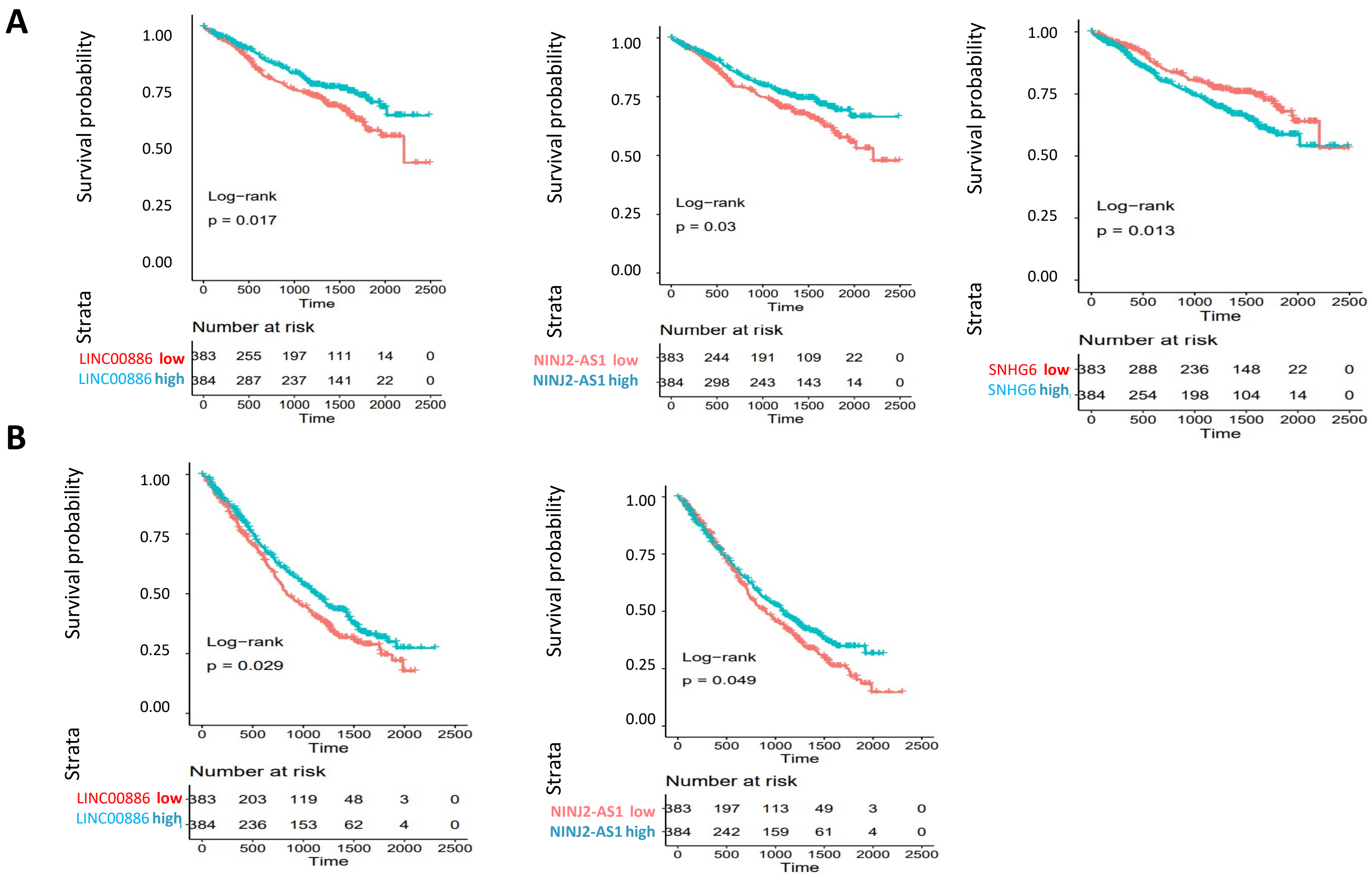

Supplementary Figure S8: Kaplan-Meiersurvivalcurvesin767BM_1MMcasesstratifiedaccordingtotheexpressionlevel ofthespecifiedlncRNAwithrespecttoOS (**A**) and PFS (**B**).

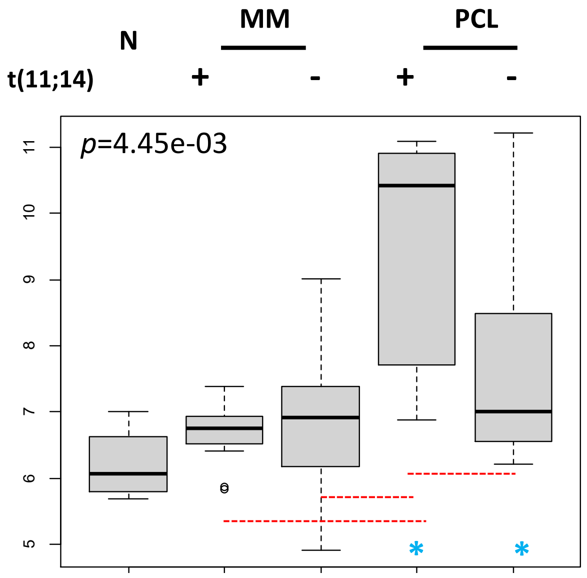

	N	MM t(11;14)	MM	pPCL t(11;14)
**MM t(11;14)**	0.184			
**MM**	0.069	0.215		
**pPCL t(11;14)**	**0.0005**	**0.0006**	**0.0010**	
**pPCL**	**0.0313**	0.086	0.17	**0.039**

Supplementary Figure S9: BoxplotrepresentationofSNHG6 geneexpressionin4normalcontrols (N),12MMpatientscarryingt(11;14),38MMwithoutt(11;14),7pPCLpatientscarryingt(11;14), and8pPCLwithoutt(11;14)evaluatedbyGeneChip®HumanGene2.0STarray(GSE116294).Red dashedlinesbelowtwogroupsindicatesignificantdifferencesintheirgeneexpressionlevel,blue asterisks indicate significant differences between the indicated group and N (Dunn's test, p-value<0.05reportedinthetableontheright).Kruskal-Wallistestp-valueisreported.
